# *PCSK9* genetic variants and risk of type 2 diabetes: a mendelian randomisation study

**DOI:** 10.1016/S2213-8587(16)30396-5

**Published:** 2017-02

**Authors:** Amand F Schmidt, Daniel I Swerdlow, Michael V Holmes, Riyaz S Patel, Zammy Fairhurst-Hunter, Donald M Lyall, Fernando Pires Hartwig, Bernardo Lessa Horta, Elina Hyppönen, Christine Power, Max Moldovan, Erik van Iperen, G Kees Hovingh, Ilja Demuth, Kristina Norman, Elisabeth Steinhagen-Thiessen, Juri Demuth, Lars Bertram, Tian Liu, Stefan Coassin, Johann Willeit, Stefan Kiechl, Karin Willeit, Dan Mason, John Wright, Richard Morris, Goya Wanamethee, Peter Whincup, Yoav Ben-Shlomo, Stela McLachlan, Jackie F Price, Mika Kivimaki, Catherine Welch, Adelaida Sanchez-Galvez, Pedro Marques-Vidal, Andrew Nicolaides, Andrie G Panayiotou, N Charlotte Onland-Moret, Yvonne T van der Schouw, Giuseppe Matullo, Giovanni Fiorito, Simonetta Guarrera, Carlotta Sacerdote, Nicholas J Wareham, Claudia Langenberg, Robert Scott, Jian'an Luan, Martin Bobak, Sofia Malyutina, Andrzej Pająk, Ruzena Kubinova, Abdonas Tamosiunas, Hynek Pikhart, Lise Lotte Nystrup Husemoen, Niels Grarup, Oluf Pedersen, Torben Hansen, Allan Linneberg, Kenneth Starup Simonsen, Jackie Cooper, Steve E Humphries, Murray Brilliant, Terrie Kitchner, Hakon Hakonarson, David S Carrell, Catherine A McCarty, H Lester Kirchner, Eric B Larson, David R Crosslin, Mariza de Andrade, Dan M Roden, Joshua C Denny, Cara Carty, Stephen Hancock, John Attia, Elizabeth Holliday, Martin O'Donnell, Salim Yusuf, Michael Chong, Guillaume Pare, Pim van der Harst, M Abdullah Said, Ruben N Eppinga, Niek Verweij, Harold Snieder, Tim Christen, Dennis O Mook-Kanamori, Stefan Gustafsson, Lars Lind, Erik Ingelsson, Raha Pazoki, Oscar Franco, Albert Hofman, Andre Uitterlinden, Abbas Dehghan, Alexander Teumer, Sebastian Baumeister, Marcus Dörr, Markus M Lerch, Uwe Völker, Henry Völzke, Joey Ward, Jill P Pell, Daniel J Smith, Tom Meade, Anke H Maitland-van der Zee, Ekaterina V Baranova, Robin Young, Ian Ford, Archie Campbell, Sandosh Padmanabhan, Michiel L Bots, Diederick E Grobbee, Philippe Froguel, Dorothée Thuillier, Beverley Balkau, Amélie Bonnefond, Bertrand Cariou, Melissa Smart, Yanchun Bao, Meena Kumari, Anubha Mahajan, Paul M Ridker, Daniel I Chasman, Alex P Reiner, Leslie A Lange, Marylyn D Ritchie, Folkert W Asselbergs, Juan-Pablo Casas, Brendan J Keating, David Preiss, Aroon D Hingorani, Naveed Sattar

**Affiliations:** a Institute of Cardiovascular Science, University College London, UK; b Department of Primary Care & Population Health, University College London, UK; c Department of Epidemiology and Public Health, UCL Institute of Epidemiology and Health Care, University College London, UK; d Centre for Cardiovascular Genetics, University College London, UK; e Farr Institute of Health Informatics Research, UCL Institute of Health Informatics, University College London, UK; f Department of Medicine, Imperial College London, London, UK; g Neuroepidemiology and Ageing Research Unit, Imperial College London, London, UK; h Department of Vascular Surgery, Imperial College London, London, UK; i Department of Genomics of Common Disease, Imperial College London, London, UK; j Faculty of Medicine, and Department of Biostatistics and Epidemiology, MRC-PHE Centre for Environment and Health, School of Public Health, Imperial College London, London, UK; k Clinical Trial Service Unit & Epidemiological Studies Unit (CTSU), Nuffield Department of Population Health, University of Oxford, Oxford, UK; l Medical Research Council Population Health Research Unit at the University of Oxford, University of Oxford, Oxford, UK; m Wellcome Trust Centre for Human Genetics, University of Oxford, Oxford, UK; n The Barts Heart Centre, St Bartholomew's Hospital, London, UK; o Institute of Health & Wellbeing, University of Glasgow, Glasgow, UK; p Robertson Centre for Biostatistics, University of Glasgow, Glasgow, UK; q Institute of Cardiovascular and Medical Sciences, University of Glasgow, Glasgow, UK; r Postgraduate Program in Epidemiology, Federal University of Pelotas, Pelotas, Brazil; s Centre for Population Health Research, Sansom Institute for Health Research, University of South Australia, Adelaide, SA, Australia; t School of Health Sciences, University of South Australia, Adelaide, SA, Australia; u Population, Policy and Practice, UCL Great Ormond Street Institute of Child Health, London, UK; v South Australian Health and Medical Research Institute, Adelaide, SA, Australia; w South Australian Health and Medical Research Institute–EMBL Australia, Adelaide, SA, Australia; x Durrer Center for Cardiovascular Research, Netherlands Heart Institute, Utrecht, Netherlands; y Department of Clinical Epidemiology, Biostatistics and Bioinformatics, Academic Medical Center Amsterdam, Netherlands; z Department of Vascular Medicine, Academic Medical Center Amsterdam, Netherlands; aa Department of Respiratory Medicine, Academic Medical Center Amsterdam, Netherlands; ab Charité Research Group on Geriatrics, Charité–Universitätsmedizin Berlin, Berlin, Germany; ac Institute of Medical and Human Genetics, Charité–Universitätsmedizin Berlin, Berlin, Germany; ad E.CA Economics GmbH, Berlin, Germany; ae Lübeck Interdisciplinary Platform for Genome Analytics (LIGA), Institutes of Neurogenetics and Integrative and Experimental Genomics, University of Lübeck, Lübeck, Germany; af Max Planck Institute for Human Development, Berlin, Germany; ag Max Planck Institute for Molecular Genetics, Berlin, Germany; ah Division of Genetic Epidemiology Innsbruck, Department of Medical Genetics, Molecular and Clinical Pharmacology, Medical University of Innsbruck, Innsbruck, Austria; ai Department of Neurology, Medical University of Innsbruck, Innsbruck, Austria; aj Bradford Institute for Health Research, Bradford Royal Infirmary, Bradford, UK; ak School of Social and Community Medicine, University of Bristol, Bristol, UK; al Population Health Research Institute, St George's, University of London, London, UK; am Centre for Population Health Sciences, Usher Institute of Population Health Sciences and Informatics, University of Edinburgh, Edinburgh, UK; an Institute of Genetics and Molecular Medicine, University of Edinburgh, Edinburgh, UK; ao Internal Medicine Unit, Department of Medicine, Lausanne University Hospital, Lausanne, Switzerland; ap Department of Surgery, Nicosia Medical School, University of Nicosia, Nicosia, Cyprus; aq Cyprus International Institute for Environmental and Public Health, Cyprus University of Technology, Limassol, Cyprus; ar Julius Center for Health Sciences and Primary Care, University Medical Center Utrecht, Utrecht, Netherlands; as Department of Cardiology, Division Heart and Lungs, University Medical Center Utrecht, Utrecht, Netherlands; at Human Genetics Foundation, HuGeF, Turin, Italy; au Department of Medical Sciences, University of Turin, Turin, Italy; av Cancer Epidemiology Unit, San Giovanni Battista Hospital, Turin, Italy; aw Centre for Oncology Prevention, CPO Piemonte, Turin, Italy; ax MRC Epidemiology Unit, Institute of Metabolic Science, University of Cambridge School of Clinical Medicine, Cambridge, UK; ay Novosibirsk State Medical University, Novosibirsk, Russia; az Institute of Internal and Preventive Medicine, Siberian Branch of the Russian Academy of Medical Sciences, Novosibirsk, Russia; ba Jagiellonian University Collegium Medicum, Krakow, Poland; bb National Institute of Public Health, Prague, Czech Republic; bc Lithuanian University of Health Sciences, Kaunas, Lithuania; bd Research Centre for Prevention and Health, Capital Region of Denmark, Denmark; be Novo Nordisk Foundation Center for Basic Metabolic Research, Faculty of Health and Medical Sciences, University of Copenhagen, Copenhagen, Denmark; bf Department of Clinical Experimental Research, Rigshospitalet, Copenhagen, Denmark; bg Department of Clinical Medicine, Faculty of Health and Medical Sciences, University of Copenhagen, Copenhagen, Denmark; bh Center for Human Genetics, Marshfield Clinic Research Foundation, Marshfield, WI, USA; bi Children's Hospital of Philadelphia, Philadelphia, PA, USA; bj Group Health Research Institute in Seattle, WA, USA; bk Essentia Institute of Rural Health, Duluth, MN, USA; bl Center for Health Research, Geisinger Clinic, Danville, PA, USA; bm Group Health Research Institute, Seattle, WA, USA; bn Division of Biomedical Statistics and Informatics, Mayo Clinic, Rochester, MN, USA; bo Departments of Medicine and Pharmacology, Vanderbilt University School of Medicine, Nashville, TN, USA; bp Department of Biomedical Informatics, Vanderbilt University School of Medicine, Nashville, TN, USA; bq George Washington University, Washington, DC, USA; br University of Newcastle, Newcastle, NSW, Australia; bs Population Health Research Institute, Hamilton, ON, Canada; bt Department of Cardiology, University Medical Center Groningen, University of Groningen, Groningen, Netherlands; bu Department of Genetics, University Medical Center Groningen, University of Groningen, Groningen, Netherlands; bv Department of Epidemiology, University Medical Center Groningen, University of Groningen, Groningen, Netherlands; bw Department of Clinical Epidemiology, Leiden University Medical Center, Leiden, Netherlands; bx Molecular Epidemiology, Department of Medical Sciences, Uppsala University, Uppsala, Sweden; by Science for Life Laboratory, Department of Medical Sciences, Uppsala University, Uppsala, Sweden; bz Division of Cardiovascular Medicine, Department of Medicine, Stanford University School of Medicine, Stanford, CA, USA; ca Department of Epidemiology, Erasmus University Medical Center, Rotterdam, Netherlands; cb Department of Internal Medicine, Erasmus University Medical Center, Rotterdam, Netherlands; cc Institute for Community Medicine, University Medicine Greifswald, Greifswald, Germany; cd Department of Internal Medicine B, University Medicine Greifswald, Greifswald, Germany; ce Department of Medicine A, University Medicine Greifswald, Greifswald, Germany; cf Interfaculty Institute of Genetics and Functional Genomics, University Medicine Greifswald, Greifswald, Germany; cg German Centre for Cardiovascular Research (DZHK), Greifswald, Germany; ch Department of Epidemiology and Preventive Medicine, University of Regensburg, Regensburg, Germany; ci Department of Non-Communicable Disease Epidemiology, London School of Hygiene & Tropical Medicine, London, UK; cj Division of Pharmacoepidemiology and Clinical Pharmacology, Utrecht Institute of Pharmaceutical Sciences, Faculty of Science, Utrecht University, Utrecht, Netherlands; ck CNRS UMR 8199, European Genomic Institute for Diabetes (EGID), Institut Pasteur de Lille, University of Lille, Lille, France; cl Renal and Cardiovascular Epidemiology, Centre de Recherche en Epidémiologie et Santé des Populations (CESP), INSERM U1018, Villejuif, France; cm I'institut du Thorax, INSERM, CNRS, University of Nantes, CHU de Nantes, Nantes, France; cn Institute for Social and Economic Research, University of Essex, Colchester, Essex, UK; co Harvard Medical School Center for Cardiovascular Disease Prevention, Brigham and Women's Hospital, Boston, MA, USA; cp Department of Epidemiology, Fred Hutchinson Cancer Research Center, University of Washington, Seattle, WA, USA; cq Anschutz Medical Campus, University of Colorado Denver, Denver, CO, USA; cr Biomedical and Translational Informatics, Geisinger Health System, Danville, PA, USA; cs Department of Biochemistry and Molecular Biology, Huck Institutes of the Life Sciences, Pennsylvania State University, University Park, PA, USA; ct Department of Surgery, University of Pennsylvania, Philadelphia, PA, USA

## Abstract

**Background:**

Statin treatment and variants in the gene encoding HMG-CoA reductase are associated with reductions in both the concentration of LDL cholesterol and the risk of coronary heart disease, but also with modest hyperglycaemia, increased bodyweight, and modestly increased risk of type 2 diabetes, which in no way offsets their substantial benefits. We sought to investigate the associations of LDL cholesterol-lowering *PCSK9* variants with type 2 diabetes and related biomarkers to gauge the likely effects of PCSK9 inhibitors on diabetes risk.

**Methods:**

In this mendelian randomisation study, we used data from cohort studies, randomised controlled trials, case control studies, and genetic consortia to estimate associations of *PCSK9* genetic variants with LDL cholesterol, fasting blood glucose, HbA_1c_, fasting insulin, bodyweight, waist-to-hip ratio, BMI, and risk of type 2 diabetes, using a standardised analysis plan, meta-analyses, and weighted gene-centric scores.

**Findings:**

Data were available for more than 550 000 individuals and 51 623 cases of type 2 diabetes. Combined analyses of four independent *PCSK9* variants (rs11583680, rs11591147, rs2479409, and rs11206510) scaled to 1 mmol/L lower LDL cholesterol showed associations with increased fasting glucose (0·09 mmol/L, 95% CI 0·02 to 0·15), bodyweight (1·03 kg, 0·24 to 1·82), waist-to-hip ratio (0·006, 0·003 to 0·010), and an odds ratio for type diabetes of 1·29 (1·11 to 1·50). Based on the collected data, we did not identify associations with HbA_1c_ (0·03%, −0·01 to 0·08), fasting insulin (0·00%, −0·06 to 0·07), and BMI (0·11 kg/m^2^, −0·09 to 0·30).

**Interpretation:**

*PCSK9* variants associated with lower LDL cholesterol were also associated with circulating higher fasting glucose concentration, bodyweight, and waist-to-hip ratio, and an increased risk of type 2 diabetes. In trials of PCSK9 inhibitor drugs, investigators should carefully assess these safety outcomes and quantify the risks and benefits of PCSK9 inhibitor treatment, as was previously done for statins.

**Funding:**

British Heart Foundation, and University College London Hospitals NHS Foundation Trust (UCLH) National Institute for Health Research (NIHR) Biomedical Research Centre.

## Introduction

The benefit of statins in reducing LDL cholesterol and coronary heart disease (CHD) risk is well established. More recently, and only after completion of numerous randomised controlled trials, was it discovered that statins increase risk of type 2 diabetes,^1^, ^2^ although this effect is modest and greatly outweighed by the benefits of this drug class. Genetic studies based on common variants in the gene encoding the target of statins, HMG-CoA reductase (HMGCR), suggest the effect is mechanism-based (ie, on-target).^3^ Genetic studies assessing the effects of variants in a broader range of genes suggest a more general link between lower LDL cholesterol and higher risk of type 2 diabetes.^4^, ^5^ Consistent with this finding, patients with autosomal dominant familial hypercholesterolaemia caused by mutations in the LDL receptor and apolipoprotein B genes are 50% less likely to be diagnosed with type 2 diabetes compared with their unaffected relatives.^6^

**Research in context**

**Evidence before this study**

We searched PubMed for “pcsk9[All Fields] AND (“antagonists and inhibitors”[Subheading] OR (“antagonists”[All Fields] AND “inhibitors”[All Fields]) OR “antagonists and inhibitors”[All Fields] OR “inhibitors”[All Fields]) AND (“diabetes mellitus”[MeSH Terms] OR (“diabetes”[All Fields] AND “mellitus”[All Fields]) OR “diabetes mellitus”[All Fields])” for articles published up to Oct 8, 2016, to identify studies that assessed treatment with PCSK9 inhibitors or carriage of genetic variants in *PCSK9* in relation to diabetes. This search identified 17 studies, two of which presented novel, yet contrasting findings in relation to genetic variants in *PCSK9* and glycaemic status.

Randomised trials of treatment with statins and carriage of corresponding genetic variants in *HMGCR* that lower LDL cholesterol both show and increase in the risk of type 2 diabetes. More recently, genetic predisposition to lower LDL cholesterol concentrations has been linked to an increased risk of diabetes, suggesting that dysglycaemia might be a consequence of lowering LDL cholesterol in general. Whether lowering of LDL cholesterol by PCSK9 inhibitors results in increased risk of diabetes is currently unknown. Clinical trials of PCSK9 inhibitors to assess their effect on cardiovascular outcomes are ongoing, but reliable evidence for a possible association between PCSK9 inhibition and risk of diabetes could take longer to accrue.

**Added value of this study**

Mendelian randomisation is an established approach that uses randomly allocated variants in the encoding gene to infer mechanism-based efficacy and safety outcomes from pharmacological perturbation of a drug target. We used four genetic variants in *PCSK9* in more than 550 000 individuals (including about 50 000 diabetes cases) and showed that *PCSK9* genetic variants associated with lower LDL cholesterol concentrations were associated with increased concentration of fasting glucose, bodyweight, and risk of diabetes. This finding adds robust new evidence to previous research that identified weak associations of *PCSK9* with risk of diabetes.

**Implications of all the available evidence**

Similar to statin therapy, treatment with PCSK9 inhibitors is likely to increase the risk of diabetes. Patients treated with PCSK9 inhibitors should be carefully monitored for dysglycaemia, including within ongoing and future clinical trials.

Gain-of-function mutations in *PCSK9*, the gene encoding proprotein convertase subtilisin/kexin type 9, also cause familial hypercholesterolaemia,^7^ whereas loss-of-function mutations in the same gene lower LDL cholesterol and protect against CHD.^8^ Consequently, monoclonal antibodies inhibiting PCSK9 have been developed^9^ and are effective in lowering LDL cholesterol by 50–70%,^10^ with preliminary evidence suggesting that this effect might be associated with reduced risk of myocardial infarction and all-cause mortality.^9^ Although large phase 3 trials to assess the effects of PCSK9 monoclonal antibodies on cardiovascular events are underway, conclusive evidence for the specific effect of PCSK9 inhibition on risk of type 2 diabetes from individual randomised controlled trials or meta-analyses might not emerge for some time.

We used the principle of mendelian randomisation as a tool for drug target validation, whereby common variants in a gene that encodes a drug target, through effects on expression or activity, are used to predict the on-target effect of pharmacological modification of the same target.^3^, ^11^, ^12^ We investigated associations of common genetic variants in *PCSK9* with markers of glycaemia, bodyweight, and risk of type 2 diabetes to assess the potential on-target effects of PCSK9 inhibition on these traits. Although results of a recent study provided evidence of an association of a single nucleotide polymorphism (SNP) in *PCSK9* with type 2 diabetes risk,^13^ our aim was to confirm the type 2 diabetes risk-increasing effect of *PCSK9* variation and explore potential biological mechanisms that might explain this effect. To do this we used four SNPs in the *PCSK9* locus collected in 50 studies supplemented with data from large genetic consortia.

## Methods

### Genetic variant selection

We selected four SNPs in or near *PCSK9* on the basis of a strong association with LDL cholesterol, as reported by the Global Lipids Genetics Consortium (GLGC);^14^ low pairwise linkage disequilibrium (r^2^≤0·30) with SNPs within the same and adjacent genes (1000 Genomes CEU data); high prior probability of being a functional variant based on the combined annotation dependent depletion (CADD) score, or the SNP being non-synonymous, or both;^15^ or previous reported associations with CHD.^16^ On the basis of these criteria, we selected the SNPs rs11583680 (minor allele frequency 0·14), rs11591147 (0·01), rs2479409 (0·36), and rs11206510 (0·17; appendix).

### Individual participant-level and summary-level data

Data were analysed from two sources. Participating studies executed a common analysis script on their own data, submitting summary estimates to a central analysis centre at University College London, London, UK. Main effect estimates from the participating studies were then meta-analysed with pooled summary estimates from the public domain data repositories of relevant genetic (genome-wide association study [GWAS]) consortia, but only if the study-level estimates had not previously contributed to consortia results, to prevent double counting. All studies contributing data to these analyses were approved by their local ethics committees.

Data were collected for LDL cholesterol, insulin (fasting and non-fasting), glucose (fasting and non-fasting), HbA_1c_, insulin resistance and secretion via basal homeostatic model assessments (HOMA-IR and HOMA-B), bodyweight, height, BMI, waist-to-hip ratio, and history or incidence of type 2 diabetes.

Publicly available summary-level data were available on blood lipids from the GLGC;^14^ type 2 diabetes-related biomarkers (plasma insulin, glucose, HbA_1c_, HOMA-IR, and HOMA-B) from the Meta-Analyses of Glucose and Insulin-related traits Consortium (MAGIC);^17^, ^18^, ^19^ bodyweight, height, BMI, and waist-to-hip ratio from the Genetic Investigation of Anthropometric Traits consortium (GIANT);^20^, ^21^ and type 2 diabetes from the Diabetes Genetics Replication and Meta-analysis consortium (DIAGRAM)^22^ and Exome chip 80K.^23^ Additionally, cross-sectional data were obtained for adiposity traits and the prevalence of type 2 diabetes from UK Biobank.^24^

### Statistical analyses

In all analyses we assumed an additive allele effect with genotypes coded as 0, 1, and 2, representing the number of minor alleles. We analysed continuous biomarkers using linear regression models; the composite endpoint of prevalent or incident type 2 diabetes was analysed with logistic regression. Study-specific associations were pooled for each SNP by use of the inverse-variance weighted method for fixed-effect and random-effects meta-analysis. We assessed between-study heterogeneity using the Q-test and the *I*^2^ statistic^25^ with a one-sided upper 97·5% CI. Study-specific associations were excluded if the SNP was not in Hardy-Weinberg equilibrium (appendix).

Our approach to SNP selection was designed to prune the number of SNPs at *PCSK9* used in the analysis, without loss of information. We decided a priori to combine the four approximately independent SNPs in a weighted gene-centric score (GS) using the inverse-variance weighted method for fixed and random effects.^26^ The GS provides a more precise estimate of the downstream effects of variation at *PCSK9* by incorporating maximum biological variation. Furthermore, if the four SNP effects are homogeneous (assessed by the heterogeneity measures Q-test and *I*^2^), the GS estimates will be more powerful and precise compared with individual SNPs in isolation. If, however, the SNP effects are heterogeneous (meaning that the *PCSK9* effects are different according to which part of the gene is assessed), the GS method will be less powerful than the individual SNP tests (depending on the degree of heterogeneity). Our aim was to estimate the effect of the *PCSK9* locus as a whole, but SNP-specific estimates are also reported. Other important assumptions of the GS approach are (approximate) independence of the included SNPs (assessed by pairwise linkage disequilibrium (r^2^) and use of multivariable regression models) and the additivity of allele effects. We also investigated whether the association of individual SNPs with diabetes risk was in proportion to the association with LDL cholesterol lowering.

Estimates are presented as mean differences or odds ratios (ORs) with 95% CIs, presented either per LDL cholesterol-decreasing allele or, in the case of GS, per 1 mmol/L (38·67 mg/dL) lower LDL cholesterol. The per 1 mmol/L GS effect estimates were derived by multiplying point estimates and their variances by the multiplicative inverse of the estimated SNP-LDL cholesterol effects. Similar to most genetic studies, missing data were excluded in an available case manner, assuming a missing-completely-at-random mechanism.^27^, ^28^ To avoid potential bias due to population stratification and non-modelled ancestry interactions, analyses excluded individuals of non-European ancestry. Differences in ancestry can be a potential source of confounding bias (ie, population stratification bias) when environment is related to both the genes and the outcome of interest. Analyses were done with the statistical programme R (version 3.3.0).

### Sensitivity analyses

We assumed that the allele effects were additive, which we assessed in available individual participant data by comparing an additive model to a non-additive model (allowing for dominance or recessiveness) using a likelihood ratio test (meta-analysed by Fisher's method).^29^ Because measurement error might be larger in prevalent cases (ascertained, for example, from hospital records) we did a further sensitivity analysis in which we separately analysed incident and prevalent type 2 diabetes. This sensitivity analysis was done not because we expect the true associations of *PCSK9* to be different with respect to prevalent and incident case status, but merely reflected a quality-control check. Although SNPs were selected to be independent, there was some degree of residual dependency (appendix; maximum r^2^ 0·26). To explore the effect of this residual correlation between the four study SNPs (appendix), we compared results from a multivariable analysis (including the four SNPs in the same model) in studies with individual participant data (correcting for this correlation) to pairwise results (ignoring any between-SNP correlation) based on the same data.

### Role of the funding source

The funder of the study had no role in study design, data collection, data analysis, data interpretation, or writing of the report. The corresponding author (AFS) had full access to all the data in the study and shared final responsibility for the decision to submit for publication with all authors.

## Results

50 studies shared participant-level data from up to 245 942 individuals, which was supplemented by summary effect estimates from data repositories, resulting in a maximum available sample size of 568 448 individuals, including 51 623 cases of incident or prevalent type 2 diabetes. Individual studies were similar with respect to the distribution of biochemical measures (assessed by the median of study-specific means): LDL cholesterol 3·41 mmol/L (IQR 0·39), fasting glucose 5·38 mmol/L (0·58), and HbA_1c_ 5·50% (appendix). Pooled pairwise linkage disequilibrium estimates for the four *PCSK9* SNPs all had r^2^ values less than 0·30 (appendix), confirming that the selected SNPs were in low correlation in the collected data.

**Figure 1:**
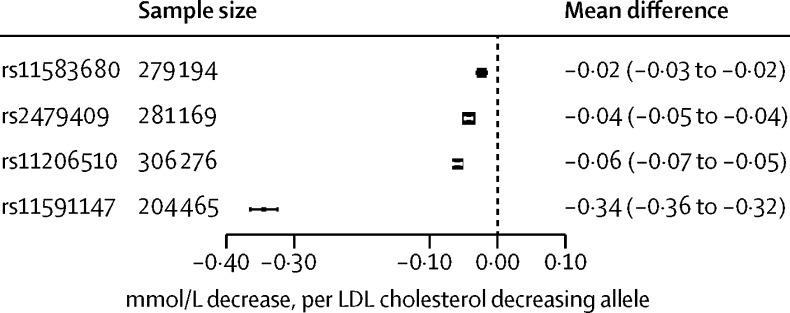
Association of genetic variants in *PCSK9* with circulating LDL cholesterol concentration Effect estimates are presented as mean difference in LDL cholesterol (mmol/L) per LDL cholesterol-lowering allele, with 95% CIs. Results are pooled by use of a fixed-effect model. The size of the black dots representing the point estimates is proportional to the inverse of the variance. Note that results from individual participant data are supplemented by repository data from the Global Lipids Genetics Consortium.

The four *PCSK9* SNPs were associated with reductions in LDL cholesterol ranging from −0·02 mmol/L (95% CI −0·03 to −0·02) for rs11583680 to −0·34 mmol/L (−0·36 to −0·32) for rs11591147 per LDL cholesterol-decreasing allele (figure 1).

**Figure 2:**
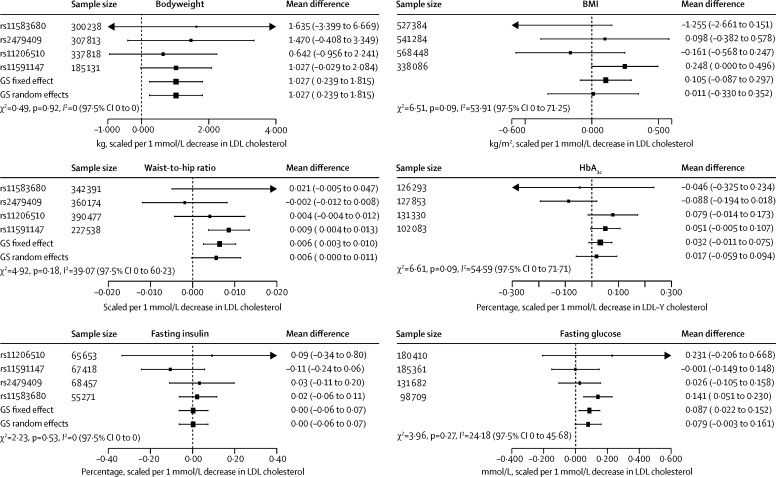
Association of genetic variants in *PCSK9* with glycaemic and anthropometric biomarkers Effect estimates are presented as mean difference with 95% CIs. Associations were scaled to a 1 mmol/L reduction in LDL cholesterol. SNP-specific results are pooled by use of a fixed-effect model; weighted gene-centric score (GS) models combining all four SNP-specific estimates are presented as fixed-effect and random-effects estimates. The size of the black dots representing the point estimates is proportional to the inverse of the variance. Between-SNP heterogeneity was measured as a two-sided Q-test (χ^2^) and an *I*^2^ with one-sided 97·5% CI. Note that results from individual participant data are supplemented by repository data from the Global Lipids Genetics Consortium, the Meta-Analyses of Glucose and Insulin-related traits Consortium, and the Genetic Investigation of Anthropometric Traits consortium.

Figure 2 depicts the associations of the four *PCSK9* SNPs after scaling the SNP effect to 1 mmol/L lower LDL cholesterol. Results of the *PCSK9* GS analysis show that a 1 mmol/L lower LDL cholesterol was associated with an increase in bodyweight of 1·03 kg (95% CI 0·24 to 1·82; and an increase of 0·006 (0·003 to 0·010) in waist-to-hip ratio, but we observed a potentially neutral association with BMI (0·11 kg/m^2^, −0·09 to 0·30). Associations of the *PCSK9* GS with glycaemia measures were 0·09 mmol/L (0·02 to 0·15) higher fasting plasma glucose, HbA_1c_ of 0·03% (−0·01 to 0·08; and for fasting insulin 0·00%, −0·06 to 0·07). SNP-specific forest plots are presented in the appendix. The estimates were similar when corrected for linkage disequilibrium (appendix), and no systematic deviations from an additive model were identified (appendix). Finally, we noted an unanticipated effect on height (mean difference 0·008 m, 0·0008 to 0·015; appendix).

**Figure 3:**
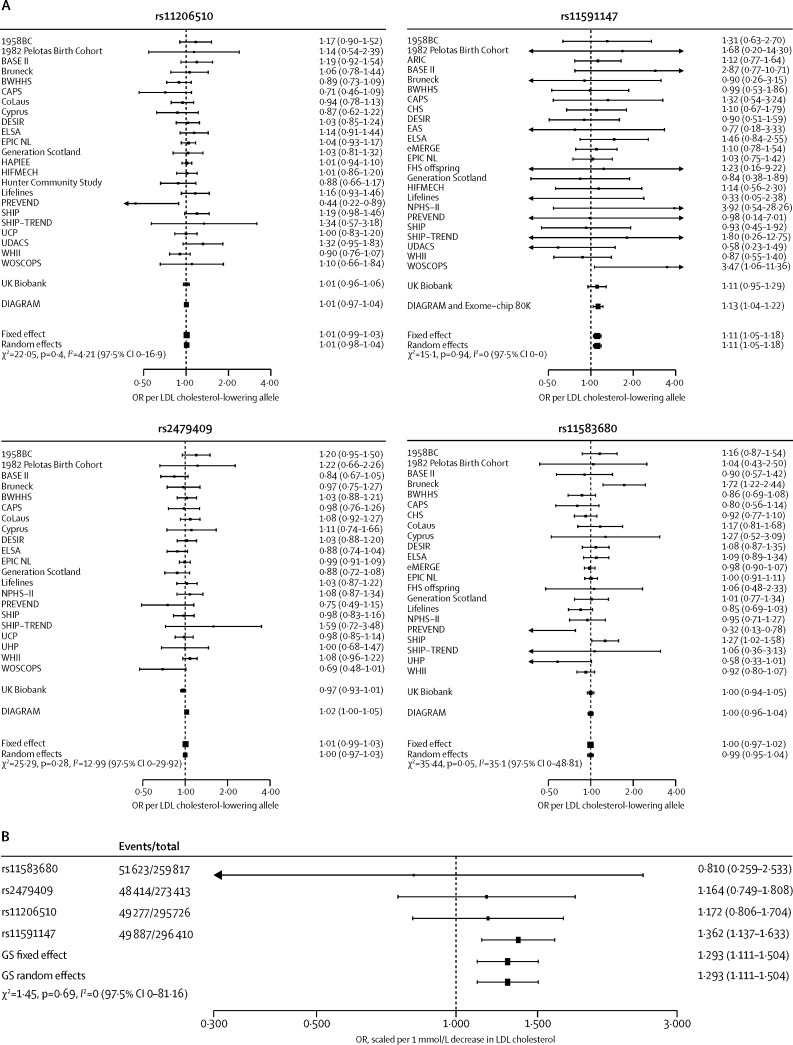
Association of genetic variants in *PCSK9* with risk of type 2 diabetes, individually (A) and as weighted gene-centric score (B) Effect estimates are presented as odds ratios (ORs) for the incidence or prevalence of type 2 diabetes, with 95% CIs. Associations were scaled to a 1 mmol/L reduction in LDL cholesterol. SNP-specific results are pooled by use of a fixed-effect model; weighted gene-centric score (GS) models combining all four SNP-specific estimates are presented as fixed-effect and random-effects estimates. The size of the black dots representing the point estimates is proportional to the inverse of the variance. Between-SNP heterogeneity was measured as a two-sided Q-test (χ^2^) and an *I*^2^ with one-sided 97·5% CI. Results from individual participant data are supplemented by repository data from the Diabetes Genetics Replication and Meta-analysis consortium.

**Figure 4:**
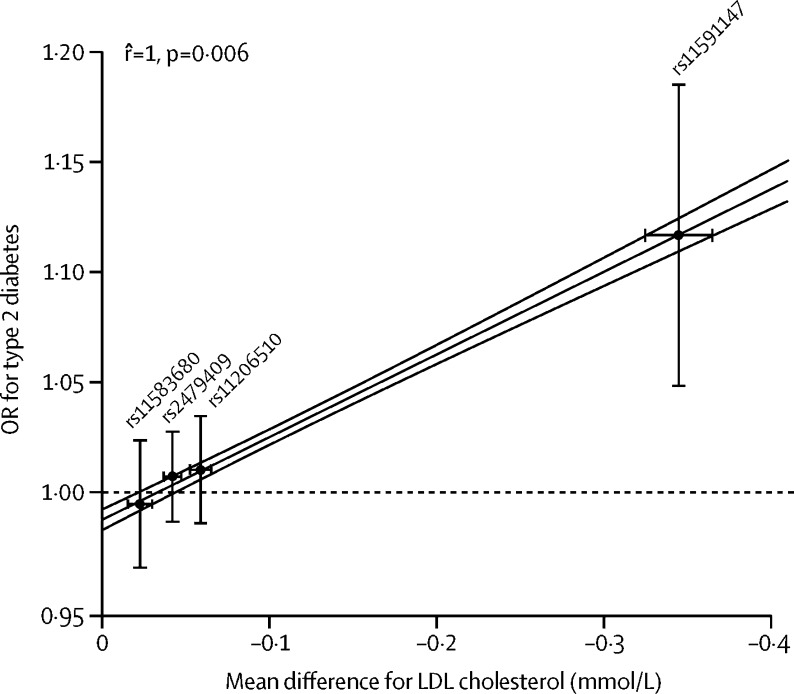
Correlation between *PCSK9* associations with LDL cholesterol concentration and type 2 diabetes Effect estimates are presented as mean difference in LDL cholesterol concentration (mmol/L) and odds ratios (ORs) for the incidence or prevalence of type 2 diabetes, with 95% CIs. Associations are presented per LDL cholesterol-decreasing allele. The Pearson correlation coefficient, regression line (grey), and its 95% CI (red) were calculated by weighting the SNPs for the inverse of the variance in the type 2 diabetes association. Excluding the SNP with the largest effect on LDL cholesterol (rs11591147) resulted in a correlation coefficient of 0·993 and a p value of 0·437.

Figure 3 shows the associations of individual *PCSK9* variants and the GS with risk of type 2 diabetes. Using the *PCSK9* GS, 1 mmol/L lower LDL cholesterol was associated with an increased risk of type 2 diabetes (OR 1·29, 95% CI 1·11 to 1·50). Exploring the *PCSK9* associations with incident (appendix) or prevalent (appendix) type 2 diabetes separately showed directional concordance of this effect (incident type 2 diabetes OR 1·15, 0·76 to 1·72; prevalent type 2 diabetes OR 1·26, 0·88 to 1·80). Associations of individual SNPs with LDL cholesterol and risk of type 2 diabetes showed a dose-response relation (figure 4).

## Discussion

In this mendelian randomisation study, genetic variants in *PCSK9,* used as a proxy for pharmacological inhibition of PCSK9, were associated with lower LDL cholesterol concentration and increased risk of type 2 diabetes. The same variants were also associated with higher fasting glucose, bodyweight, and waist-to-hip ratio, and with directionally concordant but non-significant associations for BMI and HbA_1c_ and a seemingly neutral association for fasting insulin. These results are in agreement with previous findings for variants in the *HMGCR* gene encoding the target of statin drugs, with statins modestly increasing bodyweight and the risk of type 2 diabetes.^3^

When scaled to 1 mmol/L lower LDL cholesterol, the risk for type 2 diabetes based on *HMGCR* variants^13^ was an OR of 1·39 (95% CI 1·12 to 1·73), similar to the corresponding scaled estimate for this *PCSK9* GS (1·29, 1·11 to 1·50), and similar to an estimate based on SNPs affecting LDL cholesterol selected from throughout the genome (1·27, 1·14 to 1·41).^5^ However, effect estimates obtained from mendelian randomisation studies proxy lifetime exposure to natural genetic variation, and might therefore not directly translate to the size of effect of any corresponding pharmacological treatment introduced much later in life and thus for a shorter duration of time.^30^ For example, in a meta-analysis of randomised controlled trials of statin treatment,^31^ the OR for type 2 diabetes was 1·12 (95% CI 1·06 to 1·18).

In the case of statins, the treatment benefit in terms of CHD risk reduction greatly outweighs any potential adverse effect on risk of type 2 diabetes, partly because the size of the risk reduction in CHD is greater than the risk increase in type 2 diabetes, and partly because the absolute risk of CHD in primary prevention populations eligible for statin treatment is greater than the absolute risk of type 2 diabetes.^32^ A similarly precise risk assessment for PCSK9 inhibitors awaits results from larger and longer-term randomised trials. In a recent pooled analysis,^33^ researchers reported that treatment with alirocumab was associated with an OR for type 2 diabetes of 0·89 (95% CI 0·62 to 1·28) compared with placebo, based on 133 type 2 diabetes events.

Variants that affect circulating LDL cholesterol have been reported previously to affect the probability of being prescribed a lipid-lowering drug.^34^ We were unable to account for this effect in the analysis because prescription data for these treatments were often not available, and when they were recorded they were only available for a single follow-up point. For lipid-lowering treatments, one record of treatment does not properly reflect the time-varying therapy received, and adjusting for only a single record when in fact treatment varies over follow-up might increase bias.^35^ Typically, diabetes drug treatments are much less variable over time and correction for this treatment might seem advisable; however, because of the strong correlation between history of type 2 diabetes and use of type 2 diabetes-related drugs, any correction for the latter would essentially correct for prevalent type 2 diabetes as well. Importantly, any effect of lipid-lowering drug therapy would attenuate rather than inflate any associations.

We have previously reported examples of common variants in genes encoding a protein drug target mimicking the on-target effects of pharmacological interventions on biomarkers and disease outcomes in type, direction, and relative size.^3^, ^36^, ^37^ However, such analyses cannot predict off-target effects of treatments. We refer to on-target effects as those that are due to a drug effect on the intended target (in this case PCSK9) and off-target effects as those that might occur because of the drug also binding to an unintended target (in this case, any target other than PCSK9). Although monoclonal antibody therapeutics are often highly specific, perhaps more so than small molecule therapeutics, they retain the potential for off-target effects. Hence, in the presence of off-target effects, results from ongoing randomised controlled trials could differ from the genetic associations reported here.

Our main findings are based on four *PCSK9* SNPs in combination and scaled to 1 mmol/L lower LDL cholesterol. This approach assumes additive effects across the SNPs, an assumption that held well in sensitivity analyses. A potentially unobserved non-additive effect might explain why we identified a genetic association with fasting glucose and a concordant (although non-significant) association with HbA_1c_, whereas fasting insulin seemed unaffected. Conflicting evidence exists about a possible role of *PCSK9* and PCSK9 monoclonal antibodies in disruption of pancreatic islet function.^38^, ^39^ Although concordant with fasting glucose, the HbA_1c_ association was non-significant in the collected data, which might be related to the large amount of heterogeneity between the four SNPs (upper-bound *I*^2^ 72%). Interestingly, the association of the *PCSK9* GS with BMI was smaller than that with bodyweight, which might be (partially) explained by a slightly greater average height among individuals with *PCSK9* variants associated with lower LDL cholesterol concentrations. A further potential reason for the slight discrepancy between the BMI and bodyweight associations could be the greater heterogeneity in the associations of *PCSK9* SNPs with BMI than with weight. Notably, the GS effect estimates were often driven by a large effect of SNP rs11591147; as our dose-response analysis shows (figure 4), the larger influence of this SNP appropriately reflects the proportionally larger LDL cholesterol effect of this SNP. Finally, we did not have access to measures of PCSK9 concentration in this analysis, but others^40^ have shown associations between common and rare *PCSK9* alleles (including some of the same SNPs used here) and circulating PCSK9 concentrations.

Setting aside associations with glycaemia and weight, risk of type 2 diabetes could also be increased because lifelong exposure to genetic variation in *PCSK9* might reduce mortality, making it conceivable that individuals with these variants survive longer and hence have more time to develop type 2 diabetes. However, whether *PCSK9* genotype reduces mortality has not be conclusively shown.^8^, ^41^ Irrespective of the nature of the *PCSK9* association with type 2 diabetes, large randomised trials should determine whether this relation also holds for PCSK9 monoclonal antibodies.

In a recent study,^13^ investigators used a single SNP in *PCSK9* and also reported evidence of an association with type 2 diabetes (OR 1·19, 95% CI 1·02 to 1·38; per 1 mmol/L reduction in LDL cholesterol). In the present study, we incorporated data from four SNPs, instead of a single SNP, in a *PCSK9* gene score with participant data from 50 studies supplemented by large genetic consortia and are able to confirm their results, and also show this increase in type 2 diabetes risk is likely to be related to *PCSK9*-related increases in bodyweight and glucose. Previous studies of LDL cholesterol lowering *HMGCR*^3^ and *NPC1L1*^13^ variants (encoding pharmacological targets of statins and ezetimibe, respectively) and more widely on LDL cholesterol-lowering variants from multiple GWAS-associated loci,^5^ as well as analyses of patients with monogenic hypercholesterolaemia,^6^ have provided evidence of a link between LDL cholesterol and type 2 diabetes, compatible with the findings from the present study. However, it is far from certain that all LDL cholesterol-lowering interventions will increase risk of type 2 diabetes, as not all share the same mechanism of action. The major site of both statins and PCSK9 inhibitors is thought to be the liver, through increased cellular membrane expression of the LDL receptor. The liver is also the site of action of the investigational apolipoprotein B antisense oligonucleotide mipomersen, whereas ezetimibe, the other licensed LDL cholesterol lowering drug, acts in the intestine to limit LDL cholesterol absorption. A potential unifying mechanism might be pancreatic β cell LDL receptor upregulation, increased lipid accumulation, and β cell dysfunction,^6^ but this suggestion will need to be tested experimentally.

In conclusion, genetic variants in *PCSK9* that associate with lower concentrations of LDL cholesterol are also associated with a modestly higher risk of type 2 diabetes and with associated differences in measures of glycaemia and bodyweight. Investigators of ongoing and future randomised controlled trials of PCSK9 inhibitors should carefully monitor changes in metabolic markers, including bodyweight and glycaemia, and the incidence of type 2 diabetes in study participants. Genetic studies of the type used here could be more widely used to interrogate the safety and efficacy of novel drug targets.
