# Supplementary material for: PCSK9 genetic variants and risk of type 2 diabetes: a mendelian randomisation study
Source: Lancet Diabetes Endocrinol. 2017 Feb;5(2):97–105. doi: 10.1016/S2213-8587(16)30396-5 (PMC5266795; doi:10.1016/S2213-8587(16)30396-5)
Supplement: Supplementary appendix [file mmc1.pdf]

THE LANCET
Diabetes & Endocrinology

Supplementary appendix

This appendix formed part of the original submission and has been peer reviewed. We post it as supplied by the authors.

Supplement to: Schmidt AF, Swerdlow DI, Holmes MV, et al. PCSK9 genetic variants and risk of type 2 diabetes: a mendelian randomisation study. *Lancet Diabetes Endocrinol* 2016; published online Nov 28. http://dx.doi.org/10.1016/S2213-8587(16)30396-5.

Supplementary Appendix - contents

	Page
Individual Study Acknowledgements	2
Appendix figures	9
Appendix tables	107

Individual study acknowledgments:

1958BC: Data collection was funded by grant no. G0000934 from the UK Medical Research Council and cell–line creation by Wellcome Trust grant 068545/Z/02. This research used resources provided by the Type 1 Diabetes Genetics Consortium, a collaborative clinical study sponsored by the National Institute of Diabetes and Digestive and Kidney Diseases (NIDDK), National Institute of Allergy and Infectious diseases, National Human Genome Research Institute, National Institute of Child Health and Human Development, and Juvenile Diabetes Research Foundation International (JDRF) and supported by U01DK062418. This study makes use of data generated by the Wellcome Trust Case–Control Consortium. A full list of investigators who contributed to generation of the data is available from the Wellcome Trust Case–Control Consortium website. Funding for the project was provided by the Wellcome Trust under award 076113. Support was provided by the National Institute for Health Research Biomedical Research Centre at Great Ormond Street Hospital for Children NHS Foundation Trust and University College London.

1982 Pelotas Birth Cohort: The 1982 Pelotas Birth Cohort Study is conducted by the Postgraduate Program in Epidemiology at Federal University of Pelotas (UFPel) in collaboration with the Brazilian Public Health Association (ABRASCO). From 2004 to 2013, the Wellcome Trust supported the 1982 Pelotas Birth Cohort Study. Additional funding was granted from The Brazilian National Research Council (CNPq) and Rio Grande do Sul State Research Support Foundation (FAPERGS). Previous phases of the study were supported by The International Development Research Center, World Health Organization, Overseas Development Administration, European Union, National Support Program for Centers of Excellence (PRONEX) and the Brazilian Ministry of Health. Genotyping was supported by the Department of Science and Technology (DECIT, Ministry of Health) and National Fund for Scientific and Technological Development (FNDCT, Ministry of Science and Technology), Funding of Studies and Projects (FINEP, Ministry of Science and Technology, Brazil), and Coordination of Improvement of Higher Education Personnel (CAPES, Ministry of Education, Brazil).

AMC–PAS: The AMC team is indebted to the participants of the AMC–PAS study.

BASE–II: BASE–II has been funded by the German Federal Ministry of Education and Research (BMBF) and has been formally divided into four subprojects: 'Psychology & Project Coordination and Database" (Max Planck Institute for Human Development [MPIB], grant number 16SV5837), 'Survey Methods and Social Science" (German Institute for Economic Research and Socioeconomic Panel [SOEP/DIW], grant number 16 SV5537), Medicine and Geriatrics (Charité—Universitätsmedizin, Berlin [Charité], grant number 16SV5536K), and 'Molecular Genetics" (Max Planck Institute for Molecular Genetics, now University of Lübeck [MPIMG–ULBC], grant number 16SV5538).

BiB study: Core support for Born in Bradford is provided by the Wellcome Trust (WT101597MA) and the National Institute for Health Research's Collaboration for Applied Health Research and Care (CLAHRC) for Yorkshire and Humber.

BRHS: We acknowledge the British Regional Heart Study team for data collection. The British Regional Heart study is supported by a British Heart Foundation grants (RG/08/013/25942) and BHF (RG/13/16/30528). The British Heart Foundation had no role in the design and conduct of the study; collection, management, analysis, and interpretation of the data; preparation, review, or approval of the manuscript; and decision to submit the manuscript for publication.

Bruneck: K.W., S.K., and J.W. were supported by the Translational–Research–Programme grant 'Tyrol Score" funded by the 'Land Tirol" and an excellence initiative (Competence Centers for Excellent Technologies –COMET) of the Austrian Research Promotion Agency FFG: 'Research Center of Excellence in Vascular Ageing —Tyrol, VASCage" (K–Project Nr. 843536)

funded by the BMVIT, BMWFW, the Wirtschaftsagentur Wien and the Standortagentur Tirol.

BWHHS: BWHHS is supported by funding from the British Heart Foundation and the Department of Health Policy Research Programme (England).

CAPS: CaPS was funded by the Medical Research Council and undertaken by the former MRC Epidemiology Unit (South Wales). The DNA bank was established with funding from a MRC project grant. The data archive is maintained by the University of Bristol.

CARe consortium including the ARIC, CARDIA, CHS, CFS, FHS, FHS offspring studies: The IBC array data (also known as Cardiochip or CVDSNP55v1_A from the National Heart, Lung and Blood Institute (NHLBI)Candidate Gene Association Resource (CARe) was downloaded with appropriate permissions from The database of Genotypes and Phenotypes (dbGaP) (www.ncbi.nlm.gov/gap).

CoLaus: The CoLaus study was and is supported by research grants from GlaxoSmithKline, the Faculty of Biology and Medicine of Lausanne, and the Swiss National Science Foundation (grants 33CSCO–122661, 33CS30–139468 and 33CS30–148401).

Cyprus study: The Cyprus study has been supported by grants from the Cyprus Research Promotion Foundation (YGEIA/1104/17 and PENEK_05/04) and the Cyprus Cardiovascular Educational and Research Trust

D.E.S.I.R. study: The D.E.S.I.R. study was supported by Inserm contracts with CNAMTS, Lilly, Novartis Pharma and Sanofi–Aventis, and by Inserm (Réseaux en Santé Publique, Interactions entre les déterminants de la santé, Cohortes Santé TGIR 2008), the Association Diabète Risque Vasculaire, the Fédération Française de Cardiologie, La Fondation de France, Société Francophone du Diabète, ONIVINS, Ardix Medical, Bayer Diagnostics, Becton Dickinson, Cardionics, Merck Santé, Novo Nordisk, Pierre Fabre, Roche and Topcon. The D.E.S.I.R. Study Group is composed of Inserm–U1018 (Paris: B. Balkau, P. Ducimetière, E. Eschwège), Inserm–U367 (Paris: F. Alhenc–Gelas), CHU d'Angers (A. Girault), Bichat Hospital (Paris: F. Fumeron, M. Marre, R. Roussel), CHU de Rennes (F. Bonnet), CNRS UMR–8199 (Lille: A. Bonnefond, P. Froguel), Medical Examination Services (Alençon, Angers, Blois, Caen, Chartres, Chateauroux, Cholet, LeMans, Orléans and Tours), Research Institute for General Medicine (J. Cogneau), the general practitioners of the region and the Cross–Regional Institute for Health (C. Born, E. Caces, M. Cailleau, N. Copin, J.G. Moreau, F. Rakotozafy, J. Tichet, S. Vol).

EAS: The Edinburgh Artery Study is funded by the British Heart Foundation (Programme Grant RG/98002), with Metabochip genotyping funded by a project grant from the Chief Scientist Office of Scotland (Project Grant CZB/4/672).

ELSA: Samples from the ELSA DNA Repository (EDNAR), received support under a grant (AG1764406S1) awarded by the National Institute on Ageing (NIA). ELSA was developed by a team of researchers based at the National Centre for Social Research, University College London and the Institute of Fiscal Studies. The data were collected by the National Centre for Social Research.

eMERGE: Electronic Medical Records and Genomics (eMERGE) Network was initiated and funded by the National Human Genome Research Institute (NHGRI), in conjunction with additional funding from the National Institute of General Medical Sciences (NIGMS) through the following grants: U01-HG-004610 (Group Health Cooperative/University of Washington); U01-HG-004608 (Marshfield Clinic Research Foundation and Vanderbilt University Medical Center); U01-HG-04599 (Mayo Clinic); U01HG004609 (Northwestern University); U01-HG-04603 (Vanderbilt University Medical Center, also serving as the Administrative Coordinating Center); U01HG004438 (CIDR) serving as Genotyping Center.

EPIC NL: The EPIC–NL study was funded by âĂŸEurope against Cancer' Programme of the European Commission (SANCO), Dutch Ministry of Public Health, Welfare and Sports (VWS), Netherlands Cancer Registry (NKR), LK Research Funds, Dutch Prevention Funds, Dutch Cancer Society; ZonMW the Netherlands Organisation for Health Research and Development, World Cancer Research Fund (WCRF) (The Netherlands). Genotyping for the EPIC–NL study was funded by IOP Genomics grant IGE05012 from Netherlands Enterprise Agency (RVO).

EPICOR: The EPICOR study was supported by the Human Genetics Foundation (HuGeF) and Compagnia di San Paolo (to G.M.), Turin, Italy. EPIC–Italy is further supported by a grant from the 'Associazione Italiana per la Ricerca sul Cancro" (AIRC; to C.S.). The EPICOR authors wish to thank all the EPIC–Italy PIs and colleagues for their collaboration.

ET2DS: The Edinburgh Type 2 Diabetes Study is funded by the Medical Research Council (Project Grant G0500877); the Chief Scientist Office of Scotland (Programme Support Grant CZQ/1/38); Pfizer plc (Unrestricted Investigator Led Grant); and Diabetes UK (Clinical Research Fellowship 10/0003985). Research clinics were held at the Wellcome Trust Clinical Research Facility and Princess Alexandra Eye Pavilion in Edinburgh

Fenland: The Fenland Study is funded by the Wellcome Trust and the Medical Research Council (MC_U106179471). We are grateful to all the volunteers for their time and help, and to the General Practitioners and practice staff for assistance with recruitment. We thank the Fenland Study Investigators, Fenland Study Co–ordination team and the Epidemiology Field, Data and Laboratory teams. We further acknowledge support from the Medical research council (MC_UU_12015/1).

Generation Scotland: Generation Scotland received core support from the Chief Scientist Office of the Scottish Government Health Directorates [CZD/16/6] and the Scottish Funding Council [HR03006]. Genotyping was funded by the Medical Research Council and the Wellcome Trust.

HAPIEE: The HAPIEE study was supported by Wellcome Trust 'Determinants of Cardiovascular Diseases in Eastern Europe: Longitudinal follow–up of a multi–centre cohort study" (The HAPIEE Project) (Reference number 081081/Z/06/Z); MacArthur Foundation 'Health and Social Upheaval (a research network)"; and National Institute on Aging 'Health disparities and aging in societies in transition (the HAPIEE study)" (Grant number 1R01 AG23522).

Health 2006: The Health2006 was financially supported by grants from the Velux Foundation; The Danish Medical Research Council, Danish Agency for Science, Technology and Innovation; The Aase and Ejner Danielsens Foundation; ALK–Abello A/S, Hørsholm, Denmark, and Research Centre for Prevention and Health, the Capital Region of Denmark. This work was supported by the Timber Merchant Vilhelm Bang's Foundation, the Danish Heart Foundation (Grant number 07–10–R61–A1754–B838–22392F), and the Health Insurance Foundation (Helsefonden) (Grant number 2012B233). The Novo Nordisk Foundation Center for Basic Metabolic Research is an independent Research Center at the University of Copenhagen partially funded by an unrestricted donation from the Novo Nordisk Foundation (www.metabol.ku.dk).

HIFMECH: The HIFMECH study was supported by the European Commission (BMH4–CT96—0272), the Swedish Medical Research Council, the Swedish Heart—Lung Foundation, INSERM, Université de la Méditerranée (INSERM U626), Foundation pour la Recherche Médicale (FRM), and Programme Hospitalier de Recherche Clinique (PHRC 1996). HIFMECH co–investigators are A.H., S.E.H., Irène Juhan–Vague, Maurizio Margaglione, Giovanni di Minno, John Yudkin, and Elena Tremoli.

Hunter Community Study: The University of Newcastle provided $300 000 from its Strategic

Initiatives Fund, and $600 000 from the Gladys M Brawn Senior Research Fellowship scheme; Vincent Fairfax Family Foundation, a private philanthropic trust, provided $195 000; The Hunter Medical Research Institute provided media support during the initial recruitment of participants; and Dr Anne Crotty, Prof. Rodney Scott and Associate Prof. Levi provided financial support towards freezing costs for the long–term storage of participant blood samples. The authors would like to thank the men and women participating in the HCS as well as all the staff, investigators and collaborators who have supported or been involved in the project to date.

Inter 99: The Inter99 was initiated by Torben Jørgensen (PI), Knut Borch–Johnsen (co–PI), Hans Ibsen and Troels F. Thomsen. The steering committee comprises the former two and Charlotta Pisinger. The study was financially supported by research grants from the Danish Research Council, the Danish Centre for Health Technology Assessment, Novo Nordisk Inc., Research Foundation of Copenhagen County, Ministry of Internal Affairs and Health, the Danish Heart Foundation, the Danish Pharmaceutical Association, the Augustinus Foundation, the Ib Henriksen Foundation, the Becket Foundation, and the Danish Diabetes Association.

INTERHEART and **INTERSTROKE**: Canadian Institutes of Health Research, Heart and Stroke Foundation of Canada, Canadian Stroke Network, Health Research Board Ireland, Swedish Research Council, Swedish Heart and Lung Foundation, The Health & Medical Care Committee of the Regional Executive Board, Region Västra Götaland (Sweden), AstraZeneca, Boehringer Ingelheim (Canada), Pfizer (Canada), MSD, Chest, Heart and Stroke Scotland, and The Stroke Association, with support from The UK Stroke Research Network.

JUPITER: The Justification for the Use of Statins in Prevention: an Intervention Trial Evaluating Rosuvastatin (JUPITER) was supported by AstraZeneca (to PMR). Genetic analysis in the trial population was supported by AstraZeneca (to DIC & PMR).

LifeLines: The LifeLines Cohort Study, and generation and management of GWAS genotype data for the LifeLines Cohort Study is supported by the Netherlands Organization of Scientific Research NWO (grant 175.010.2007.006), the Economic Structure Enhancing Fund (FES) of the Dutch government, the Ministry of Economic Affairs, the Ministry of Education, Culture and Science, the Ministry for Health, Welfare and Sports, the Northern Netherlands Collaboration of Provinces (SNN), the Province of Groningen, University Medical Center Groningen, the University of Groningen, Dutch Kidney Foundation and Dutch Diabetes Research Foundation. The authors wish to acknowledge the services of the Lifelines Cohort Study, the contributing research centers delivering data to Lifelines, and all the study participants.

NEO study: The Netherlands Epidemiology of Obesity (NEO) study: The NEO was designed for extensive phenotyping to investigate pathways that lead to obesity–related diseases. The NEO study is a population–based, prospective cohort study that includes 6,671 individuals aged 45—65 years, with an oversampling of individuals with overweight or obesity. At baseline, information on demography, lifestyle, and medical history have been collected by questionnaires. In addition, samples of 24–h urine, fasting and postprandial blood plasma and serum, and DNA were collected. Genotyping was performed using the Illumina HumanCoreExome chip, which was subsequently imputed to the 1000 genome reference panel. Participants underwent an extensive physical examination, including anthropometry, electrocardiography, spirometry, and measurement of the carotid artery intima–media thickness by ultrasonography. In random subsamples of participants, magnetic resonance imaging of abdominal fat, pulse wave velocity of the aorta, heart, and brain, magnetic resonance spectroscopy of the liver, indirect calorimetry, dual energy X–ray absorptiometry, or accelerometry measurements were performed. The collection of data started in September 2008 and completed at the end of September 2012. Participants are currently being followed for the incidence of obesity–related diseases and mortality.The authors of the NEO study thank all individuals who participated in the Netherlands Epidemiology in Obesity study, all participating general practitioners for inviting eligible participants and all research nurses for collection of the data. We thank the NEO study group, Pat van Beelen, Petra

Noordijk and Ingeborg de Jonge for the coordination, lab and data management of the NEO study. The genotyping in the NEO study was supported by the Centre National de Génotypage (Paris, France), headed by Jean–Francois Deleuze. The NEO study is supported by the participating Departments, the Division and the Board of Directors of the Leiden University Medical Center, and by the Leiden University, Research Profile Area Vascular and Regenerative Medicine.

NPHS–II: The NPHS–II study was supported by the Medical Research Council, the US National Institutes of Health (NIH 33014), and Du Pont Pharma.

PIVUS: This project was supported by Knut and Alice Wallenberg Foundation (Wallenberg Academy Fellow), Swedish Diabetes Foundation (2013–024), Swedish Research Council (2012–1397 and 2015–02907), and Swedish Heart–Lung Foundation (20140422). Computations were performed on resources provided by SNIC through Uppsala Multidisciplinary Center for Advanced Computational Science (UPPMAX) under Project b2011036.

PREVEND: PREVEND genetics is supported by the Dutch Kidney Foundation (Grant E033), the National Institutes of Health (grant 2R01LM010098), The Netherlands organisation for health research and development (NWO–Groot grant 175.010.2007.006, NWO VENI grant 916.761.70, ZonMw grant 90.700.441).

SHIP and **SHIP–Trend** studies: SHIP is part of the Community Medicine Research net of the University of Greifswald, Germany, which is funded by the Federal Ministry of Education and Research (grants no. 01ZZ9603, 01ZZ0103, and 01ZZ0403), the Ministry of Cultural Affairs as well as the Social Ministry of the Federal State of Mecklenburg–West Pomerania, and the network 'Greifswald Approach to Individualized Medicine (GANI_MED)" funded by the Federal Ministry of Education and Research (grant 03IS2061A). Genome–wide data have been supported by the Federal Ministry of Education and Research (grant no. 03ZIK012) and a joint grant from Siemens Healthcare, Erlangen, Germany and the Federal State of Mecklenburg–West Pomerania. The University of Greifswald is a member of the Caché Campus program of the InterSystems GmbH.

TPT: We thank Professor Tom Meade for use of data from the Thrombosis Prevention Trial.

UCP: The UCP studies were funded by the Netherlands Heart Foundation and the Dutch Top Institute Pharma Mondriaan Project.

UDACS: Diabetes UK (BDA: RD01/0001357) supported the creation of UDACS.

UK Biobank This research has been conducted using the UK Biobank resource. The authors are grateful to UK Biobank participants. UK Biobank was established by the Wellcome Trust medical charity, Medical Research Council, Department of Health, Scottish Government, and the Northwest Regional Development Agency. It has also had funding from the Welsh Assembly Government and the British Heart Foundation. Diabetes cases were self-reported, and a minority may have type 1 rather than type 2 diabetes.

UKHLS: UKHLS was funded by the Economic and Social Research Council (RES–586–47–0002). The data were collected by NatCen and the genotyping was conducted by the Wellcome Trust Sanger Institute. Information on how to access the data can be found on the Understanding Society website https://www.understandingsociety.ac.uk/.

UHP: The Utrecht Health Project (UHP) is an ongoing dynamic population study initiated in a newly developed large residential area in Leidsche Rijn, part of the city of Utrecht and made possible by structural grants from the Julius Center.

WGHS: The Women's Genome Health Study (WGHS) was supported by grants from the Na-

tional Heart, Lung, and Blood Institute (HL043851 and HL080467) and the National Cancer Institute (CA047988) to Julie E. Buring, with collaborative scientific support and funding for genotyping provided by Amgen (to PMR & DIC). The WGHS is currently supported by a grant from the NCI (UM1CA182913) to Julie E. Buring and I–Min Lee.

WHII: The WHII study is supported by grants from the Medical Research Council (G0902037; ID85374), British Heart Foundation (RG/07/008/23674), Stroke Association, National Heart Lung and Blood Institute (5RO1 HL036310), National Institute on Aging (5RO1AG13196) Agency for Health Care Policy Research (HS06516), and the John D. and Catherine T. MacArthur Foundation Research Networks on Successful Midlife Development and Socio–economic Status and Health.

WHI: The WHI program is funded by the National Heart, Lung, and Blood Institute, National Institutes of Health, U.S. Department of Health and Human Services through contracts HHSN268201100046C, HHSN268201100001C, HHSN268201100002C, HHSN268201100003C, HHSN268201100004C, and HHSN271201100004C.

Data on lipid traits have been contributed by the **GLGC** investigators and have been downloaded from http://csg.sph.umich.edu//abecasis/index.html. Data on glycemic traits have been contributed by the **MAGIC** investigators and have been downloaded from www.magicinvestigators.org. Data on anthropometric traits have been contributed by the **GIANT** investigators and have been downloaded from https://www.broadinstitute.org/collaboration/giant/index.php/Main_Page. Data on type 2 diabetes have contributed from **DIAGRAM** investigators and Exome chip 80K downloaded from http://www.diagram–consortium.org/index.html and type 2 diabetes knowledge portal (http://www.type2diabetesgenetics.org/)

LifeLines group authors

Behrooz Z Alizadeh (1), H Marike Boezen (1), Lude Franke (2), Pim van der Harst (3), Gerjan Navis (4), Marianne Rots (5), Harold Snieder (1), Morris Swertz (2), Bruce HR Wolffenbuttel (6), Cisca Wijmenga (2).

1. Department of Epidemiology, University of Groningen, University Medical Center Groningen, The Netherlands
2. Department of Genetics, University of Groningen, University Medical Center Groningen, The Netherlands
3. Department of Cardiology, University of Groningen, University Medical Center Groningen, The Netherlands
4. Department of Internal Medicine, Division of Nephrology, University of Groningen, University Medical Center Groningen, The Netherlands
5. Department of Medical Biology, University of Groningen, University Medical Center Groningen, The Netherlands
6. Department of Endocrinology, University of Groningen, University Medical Center Groningen, The Netherlands

UCLEB consortium authors

Borges C (1), Caddidy A (2), Charoen P (1), Chaturvedi N (3), Dale C (1), Drenos F (4), Dudbridge F (2), Engmann J (1), Finan C (1), Garfield V(5), Gaunt T (6), Gentry-Maharaj A (7), Jefferis B (8), Kuh D (9), Lawlor D (6), Mclachlan S (10), Menon U (7), Plagnol V (11), Price A (10), Sofat R (12), Talmud P (4), Tillin T (13), Walker A (4), White J (11), Whittaker J (14), Wong A (9).

1. Institute of Cardiovascular Science, University College London, UK
2. Dept Non-communicable Disease Epidemiology, London School of Hygiene and Tropical Medicine, UK
3. Cardiometabolic Phenotyping Group, Institute of Cardiovascular Science, University College London, UK
4. Centre for Cardiovascular Genetics, Dept. of Medicine, University College London, UK
5. Department of Epidemiology & Public Health, UCL Institute of Epidemiology & Health Care, University College London, UK
6. MRC Integrative Epidemiology Unit, School of Social and Community Medicine, University of Bristol, Bristol, UK
7. Institute for Women's Health, Faculty of Population Health Sciences, University College London, UK
8. Dept Primary Care & Population Health, University College London, UK
9. MRC Unit for Lifelong Health and Ageing, London, UK
10. Centre for Population Health Sciences, The Usher Institute of Population Health Sciences and Informatics, University of Edinburgh, UK
11. University College London Genetics Institute, Department of Genetics, Environment and Evolution, London, UK
12. Centre for Clinical Pharmacology, University College London, London, UK
13. Cardiometabolic Phenotyping Group, Institute of Cardiovascular Science, University College London, UK
14. Genetics Division, Research and Development, GlaxoSmithKline, Harlow, UK

The UCLEB consortium is supported by a British Heart Foundation Programme Grant (RG/10/12/28456).

Appendix Figures

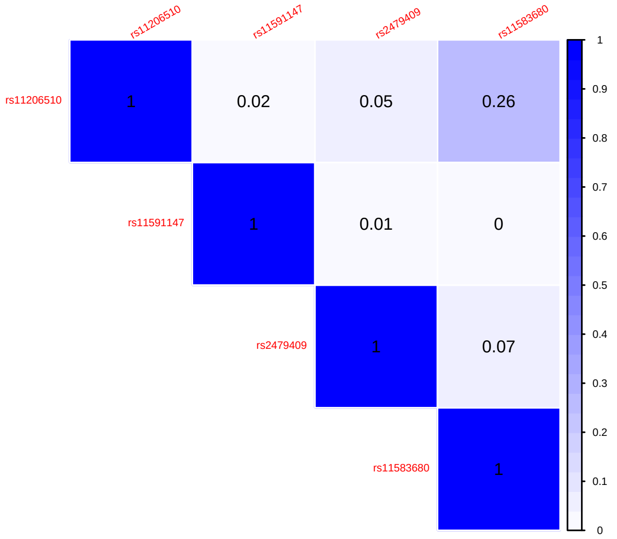

Figure 1: Linkage disequilibrium estimates, as r^2, between the *PCSK9* SNPs. Study specific estimates were pooled based on a random effects model.

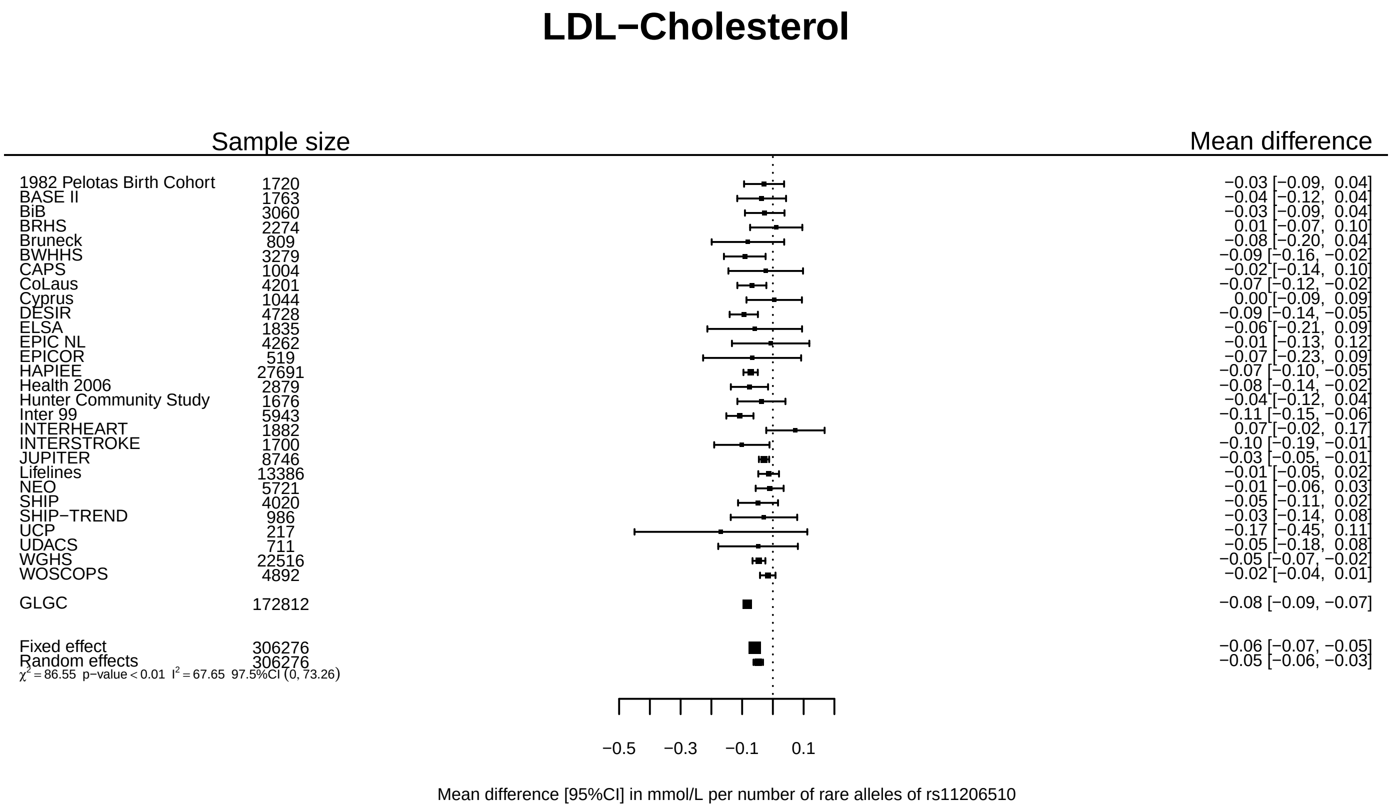

Figure 2: Mean difference of PCSK9 SNP rs11206510 on LDL–C (mmol/L) per number of rare alleles, with 95% confidence interval (CI). Results are pooled using a fixed and random effects model, irrespective of observed between study heterogeneity; measured as a two sided Q–test (χ^2) and the I^2 with one–sided 97.5% CI.

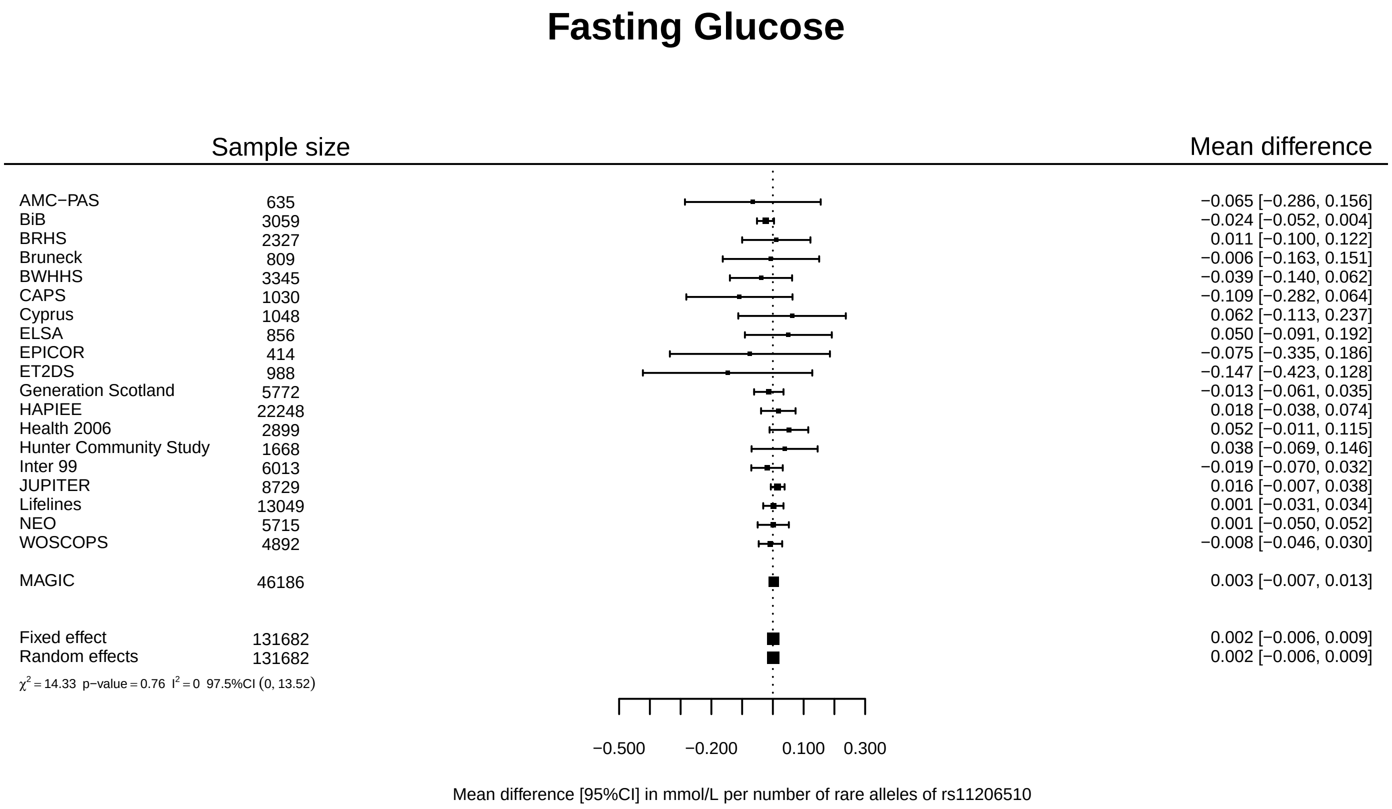

Figure 3: Mean difference of PCSK9 SNP rs11206510 on fasting glucose (mmol/L) per number of rare alleles, with 95% confidence interval (CI). Results are pooled using a fixed and random effects model, irrespective of observed between study heterogeneity; measured as a two sided Q–test (χ^2) and the I^2 with one–sided 97.5% CI.

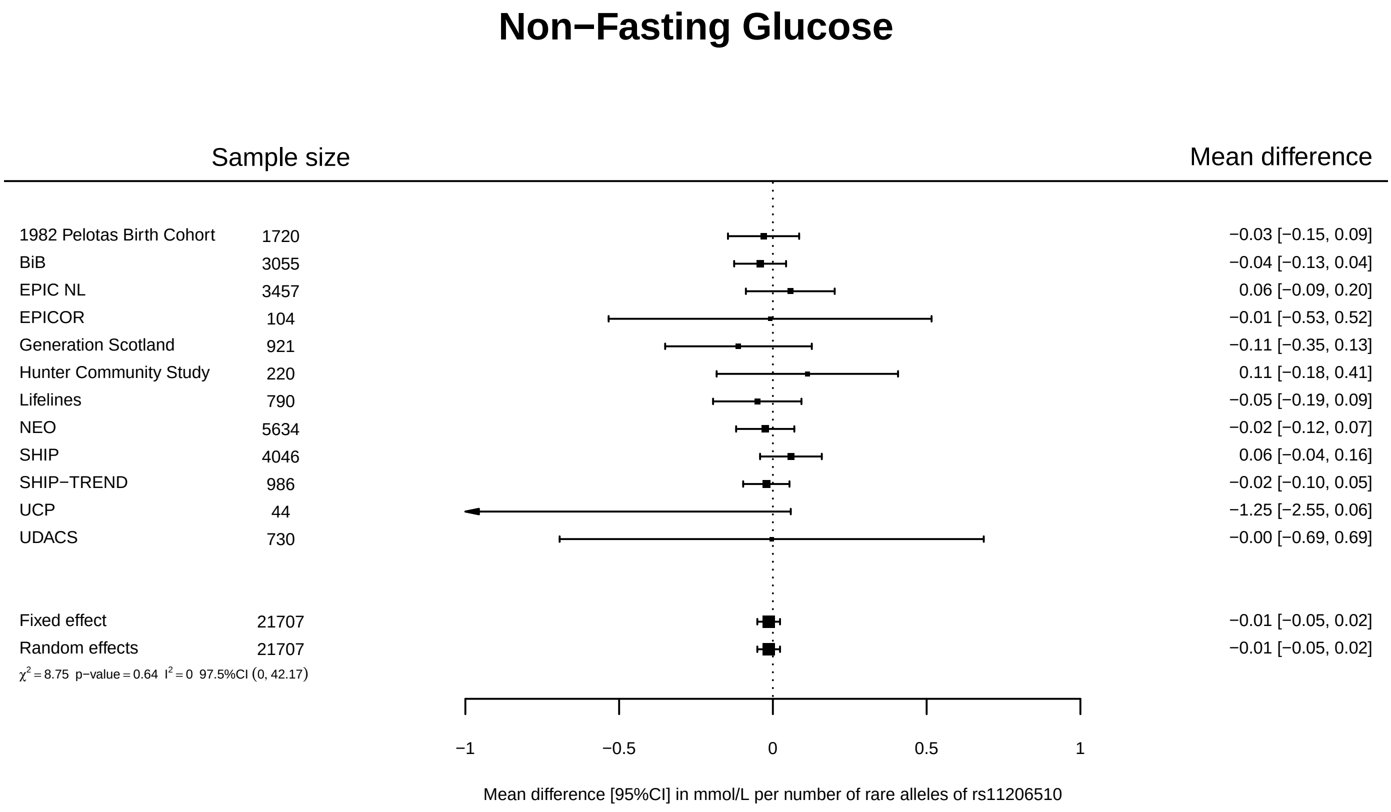

Figure 4: Mean difference of PCSK9 SNP rs11206510 on non–fasting glucose (mmol/L) per number of rare alleles, with 95% confidence interval (CI). Results are pooled using a fixed and random effects model, irrespective of observed between study heterogeneity; measured as a two sided Q–test (χ^2) and the I^2 with one–sided 97.5% CI.

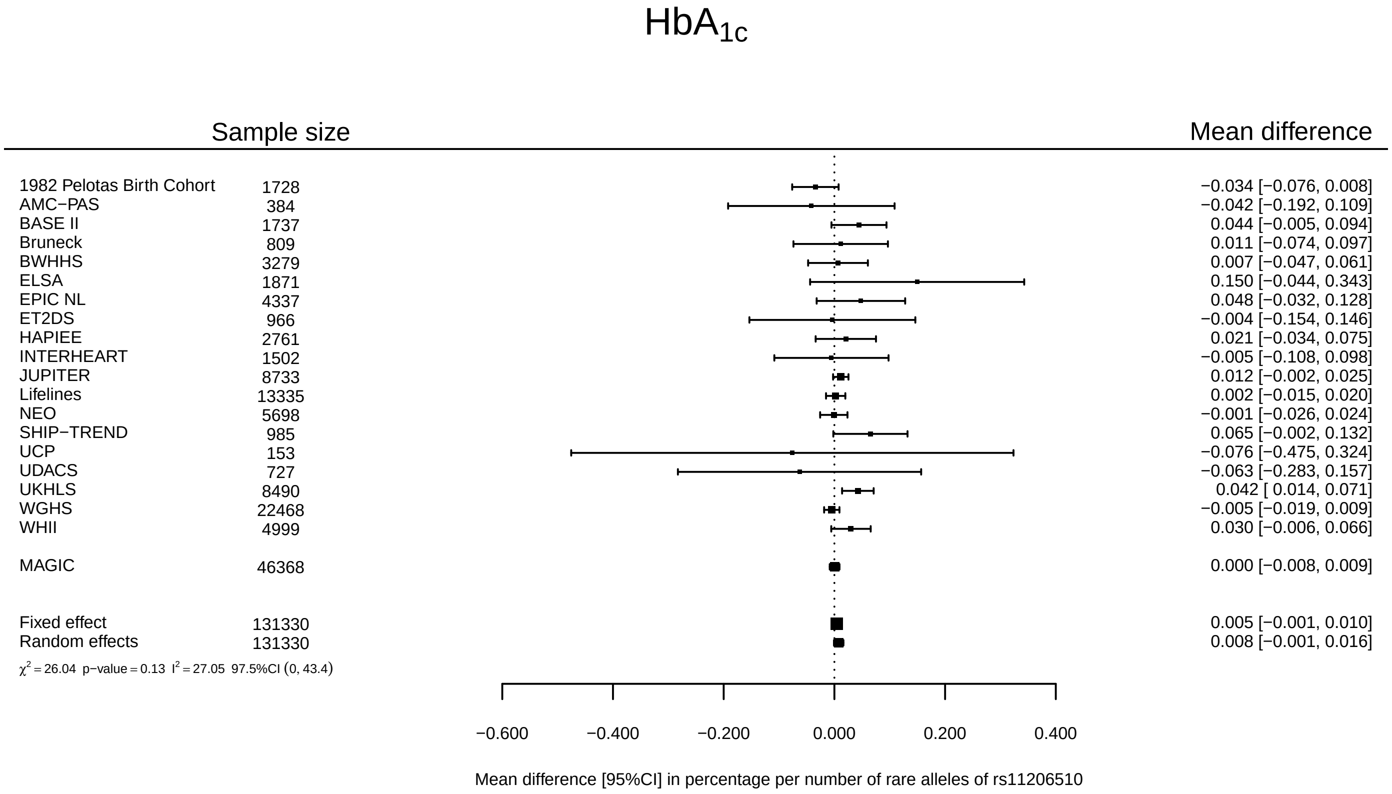

Figure 5: Mean difference of PCSK9 SNP rs11206510 on HbA_{1c} (percentage) per number of rare alleles, with 95% confidence interval (CI). Results are pooled using a fixed and random effects model, irrespective of observed between study heterogeneity; measured as a two sided Q–test (χ^2) and the I^2 with one–sided 97.5% CI.

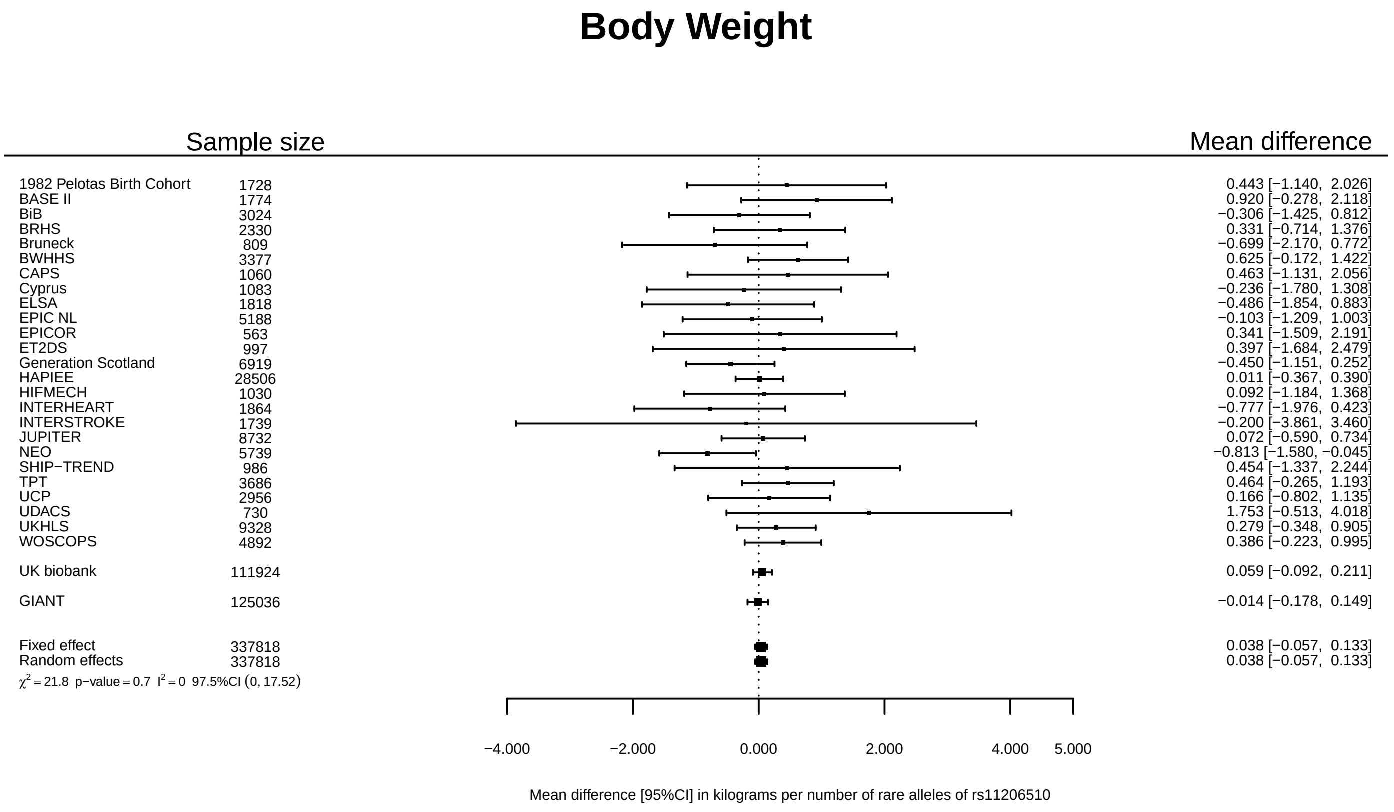

Figure 6: Mean difference of PCSK9 SNP rs11206510 on body weight (kilogram) per number of rare alleles, with 95% confidence interval (CI). Results are pooled using a fixed and random effects model, irrespective of observed between study heterogeneity; measured as a two sided Q–test (χ^2) and the I^2 with one–sided 97.5% CI.

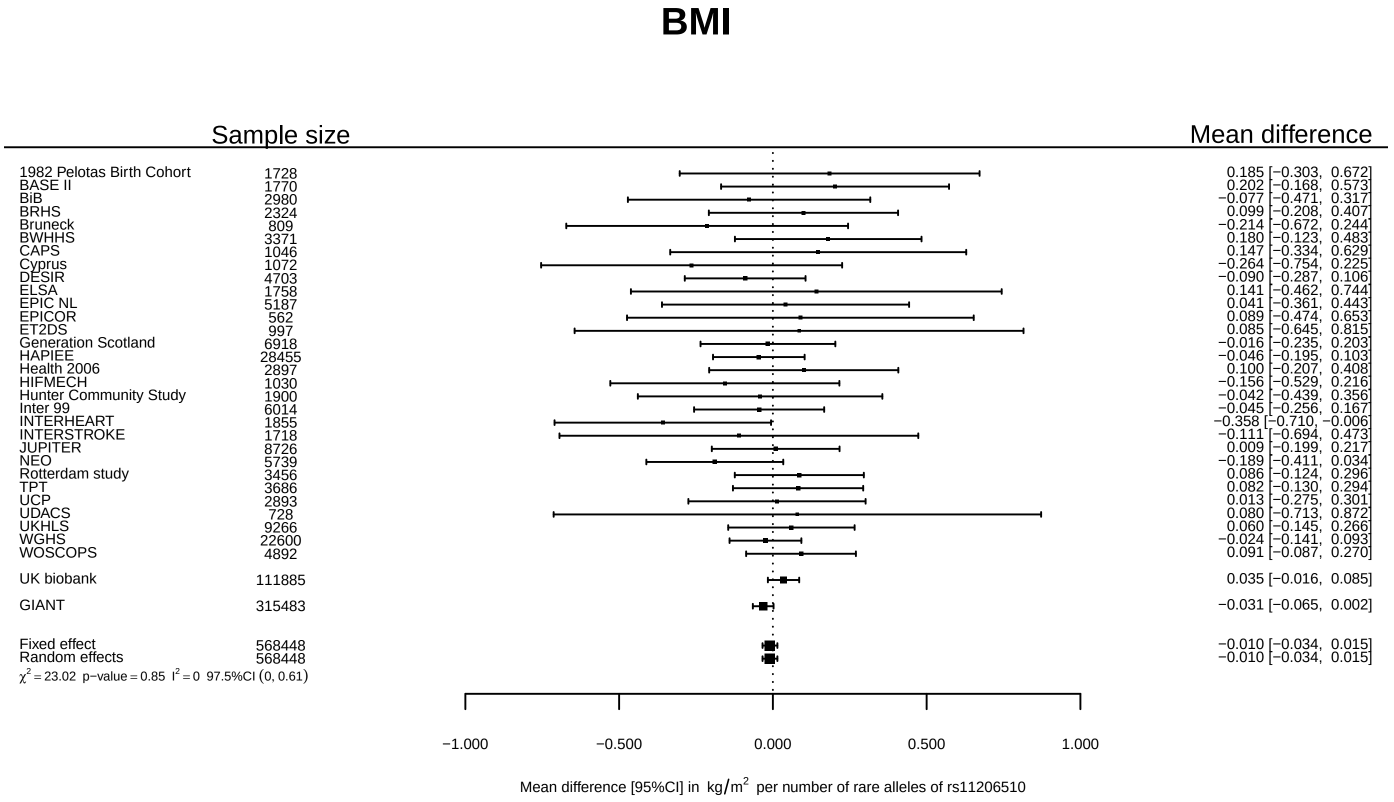

Figure 7: Mean difference of PCSK9 SNP rs11206510 on BMI (kg/m^2) per number of rare alleles, with 95% confidence interval (CI). Results are pooled using a fixed and random effects model, irrespective of observed between study heterogeneity; measured as a two sided Q–test (χ^2) and the I^2 with one–sided 97.5% CI.

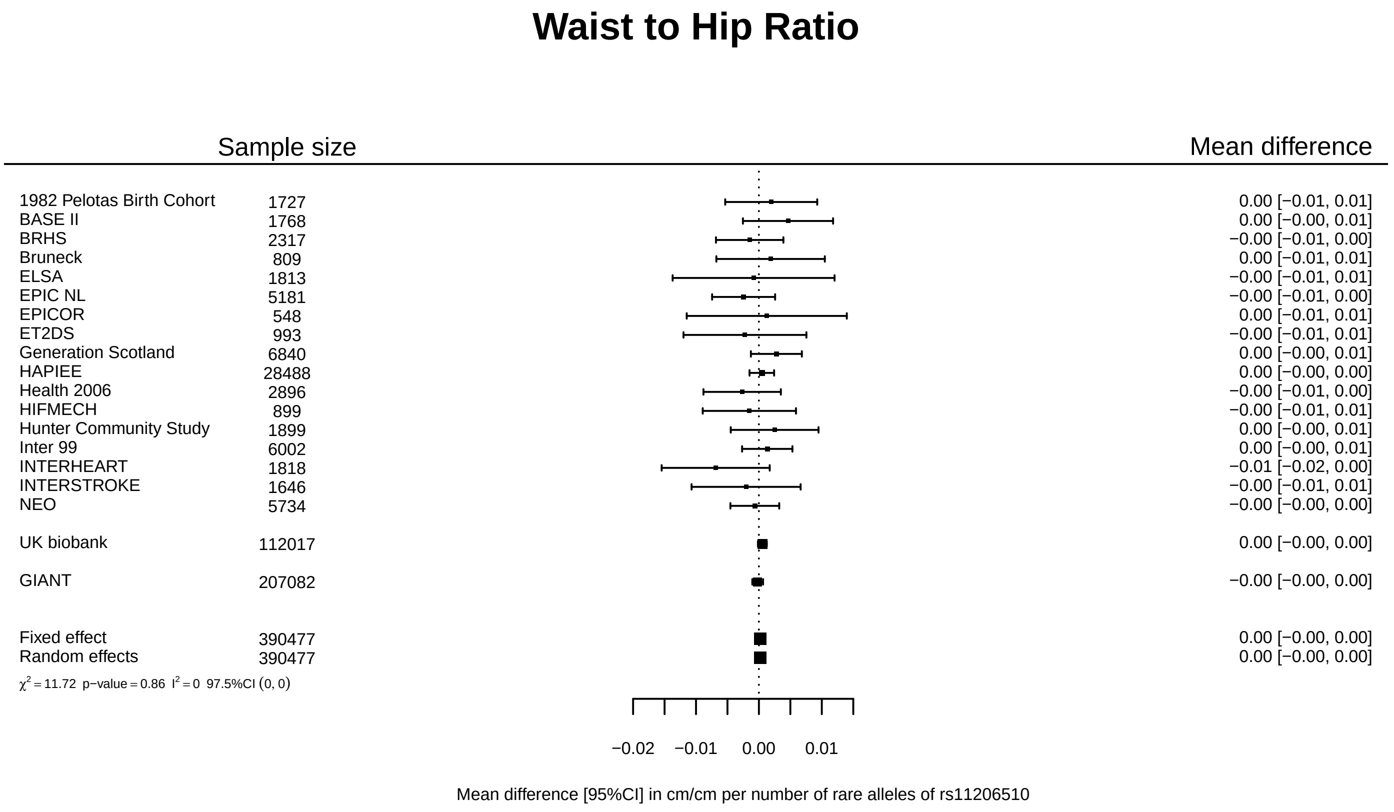

Figure 8: Mean difference of PCSK9 SNP rs11206510 on waist to hip ratio per number of rare alleles, with 95% confidence interval (CI). Results are pooled using a fixed and random effects model, irrespective of observed between study heterogeneity; measured as a two sided Q–test (χ^2) and the I^2 with one–sided 97.5% CI.

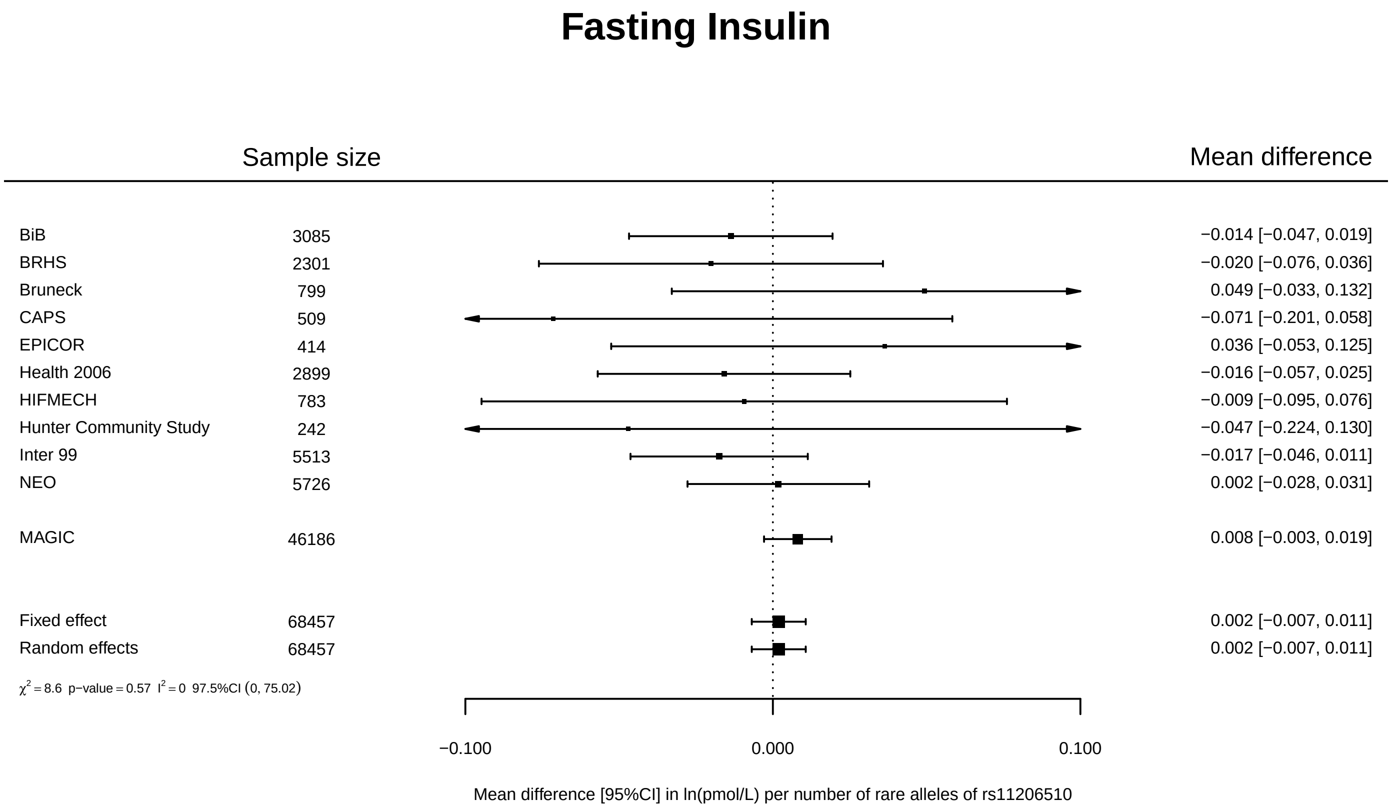

Figure 9: Mean difference of PCSK9 SNP rs11206510 on fasting insulin $ln(pmol/L)$ per number of rare alleles, with 95% confidence interval (CI). Results are pooled using a fixed and random effects model, irrespective of observed between study heterogeneity; measured as a two sided Q–test (χ^2) and the I^2 with one–sided 97.5% CI.

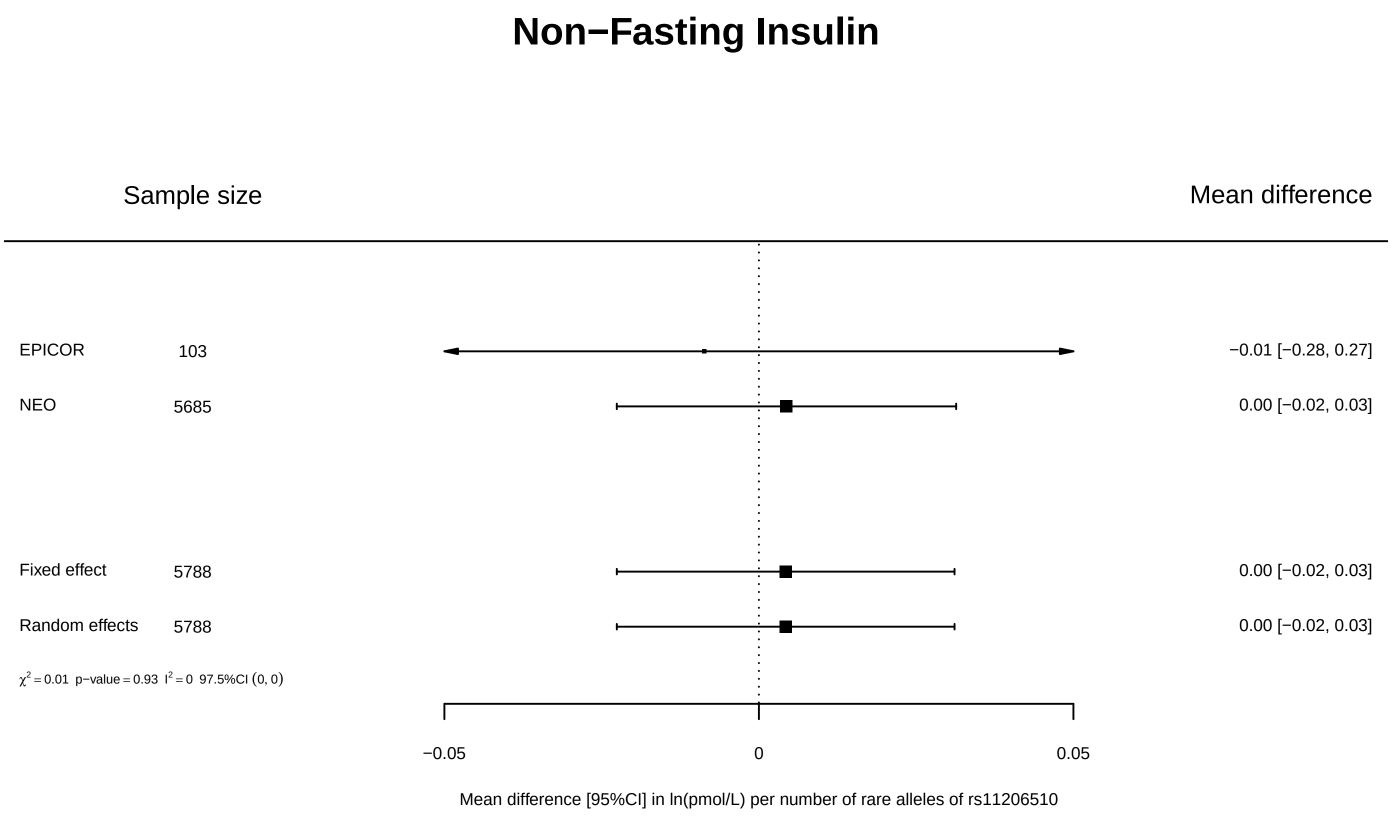

Figure 10: Mean difference of PCSK9 SNP rs11206510 on non–fasting insulin $ln(pmol/L)$ per number of rare alleles, with 95% confidence interval (CI). Results are pooled using a fixed and random effects model, irrespective of observed between study heterogeneity; measured as a two sided Q–test (χ^2) and the I^2 with one–sided 97.5% CI.

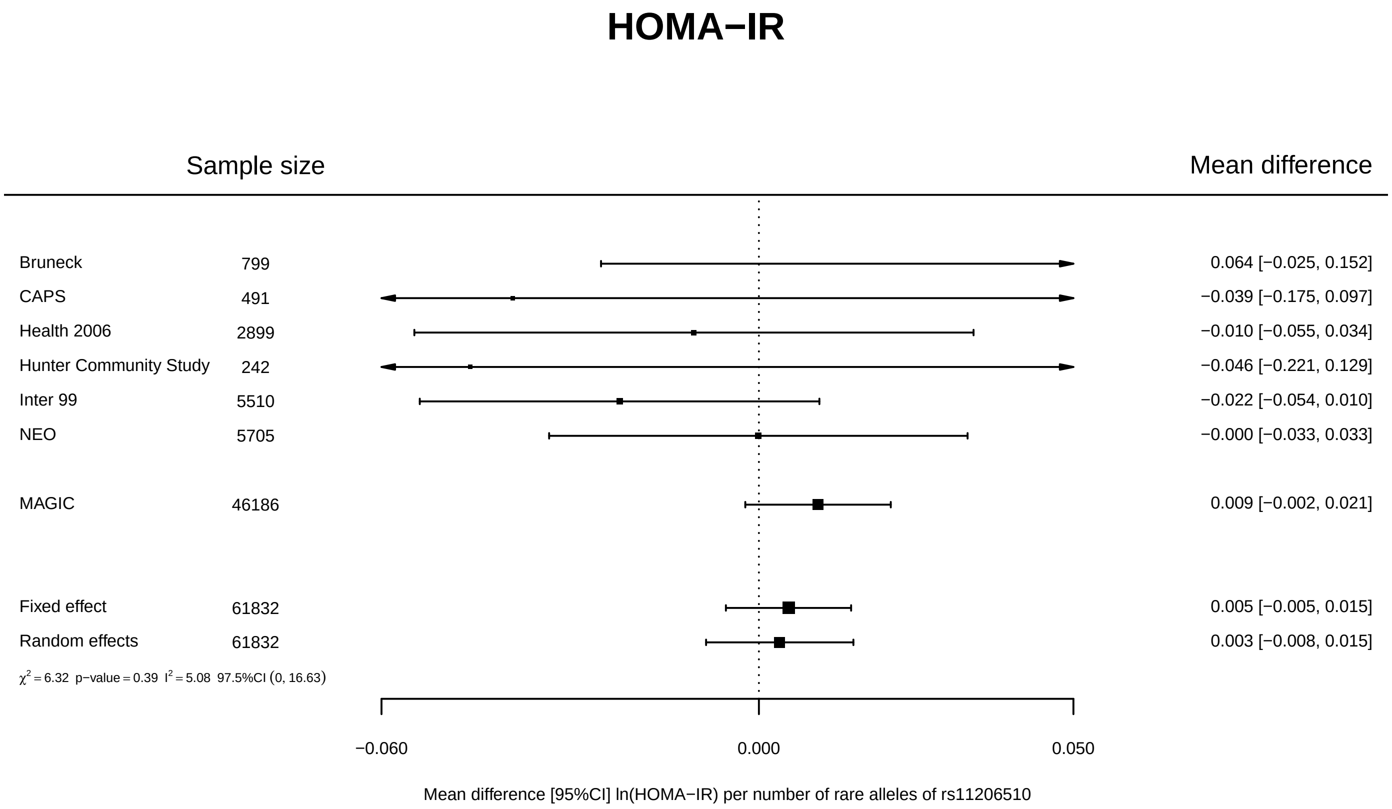

Figure 11: Mean difference of PCSK9 SNP rs11206510 on $ln(HOMA\check{\ }IR)$ per number of rare alleles, with 95% confidence interval (CI). Results are pooled using a fixed and random effects model, irrespective of observed between study heterogeneity; measured as a two sided Q–test (χ^2) and the I^2 with one–sided 97.5% CI.

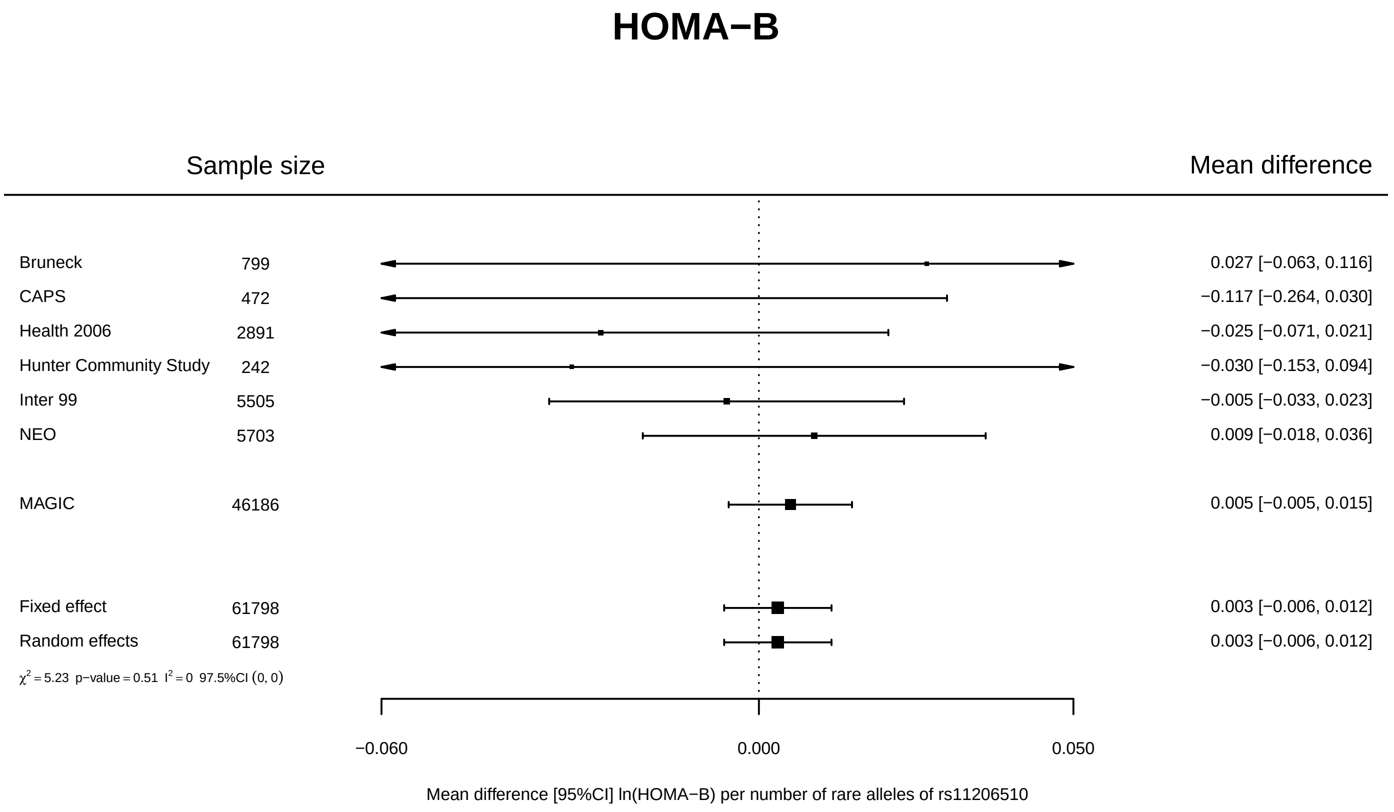

Figure 12: Mean difference of PCSK9 SNP rs11206510 on $ln(HOMA\breve{}\beta)$ per number of rare alleles, with 95% confidence interval (CI). Results are pooled using a fixed and random effects model, irrespective of observed between study heterogeneity; measured as a two sided Q–test (χ^2) and the I^2 with one–sided 97.5% CI.

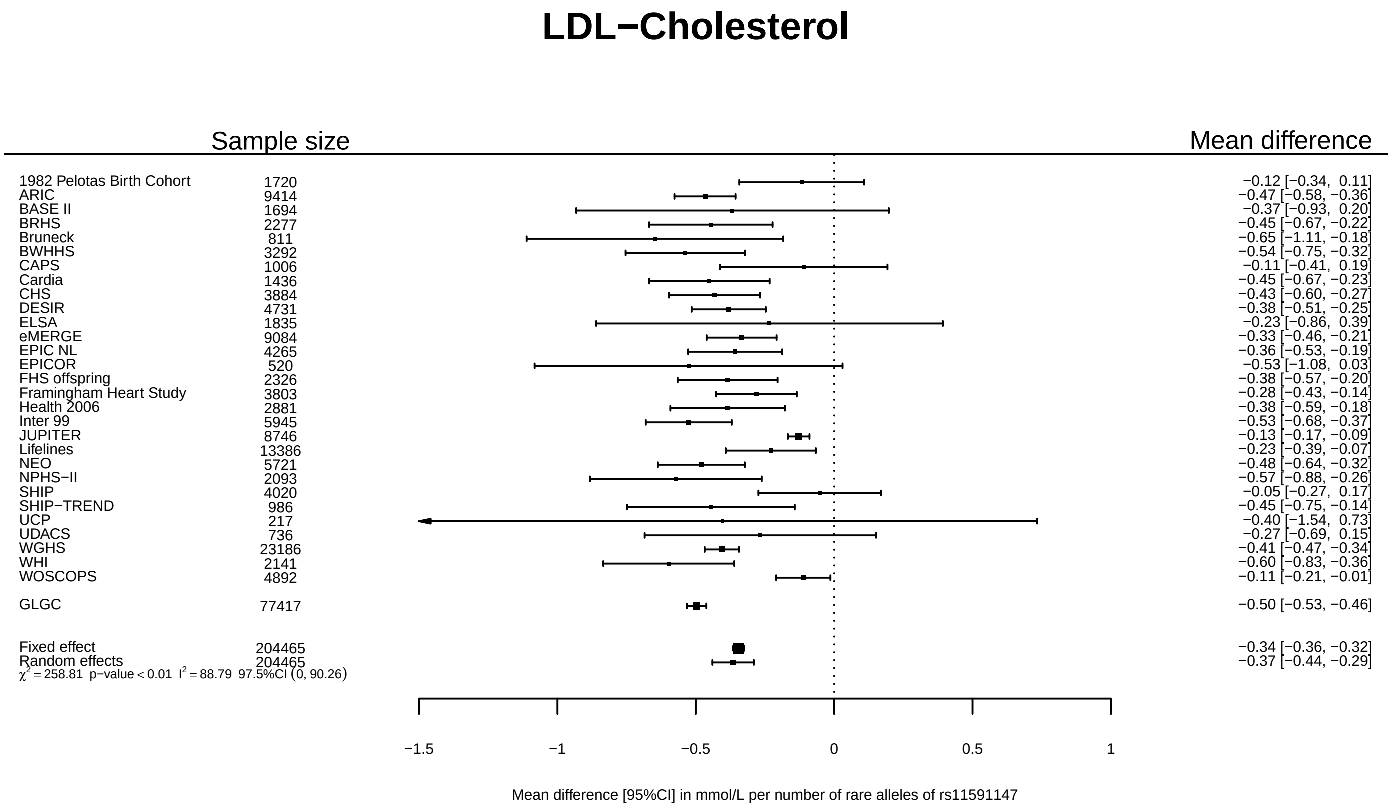

Figure 13: Mean difference of PCSK9 SNP rs11591147 on LDL–C (mmol/L) per number of rare alleles, with 95% confidence interval (CI). Results are pooled using a fixed and random effects model, irrespective of observed between study heterogeneity; measured as a two sided Q–test (χ^2) and the I^2 with one–sided 97.5% CI.

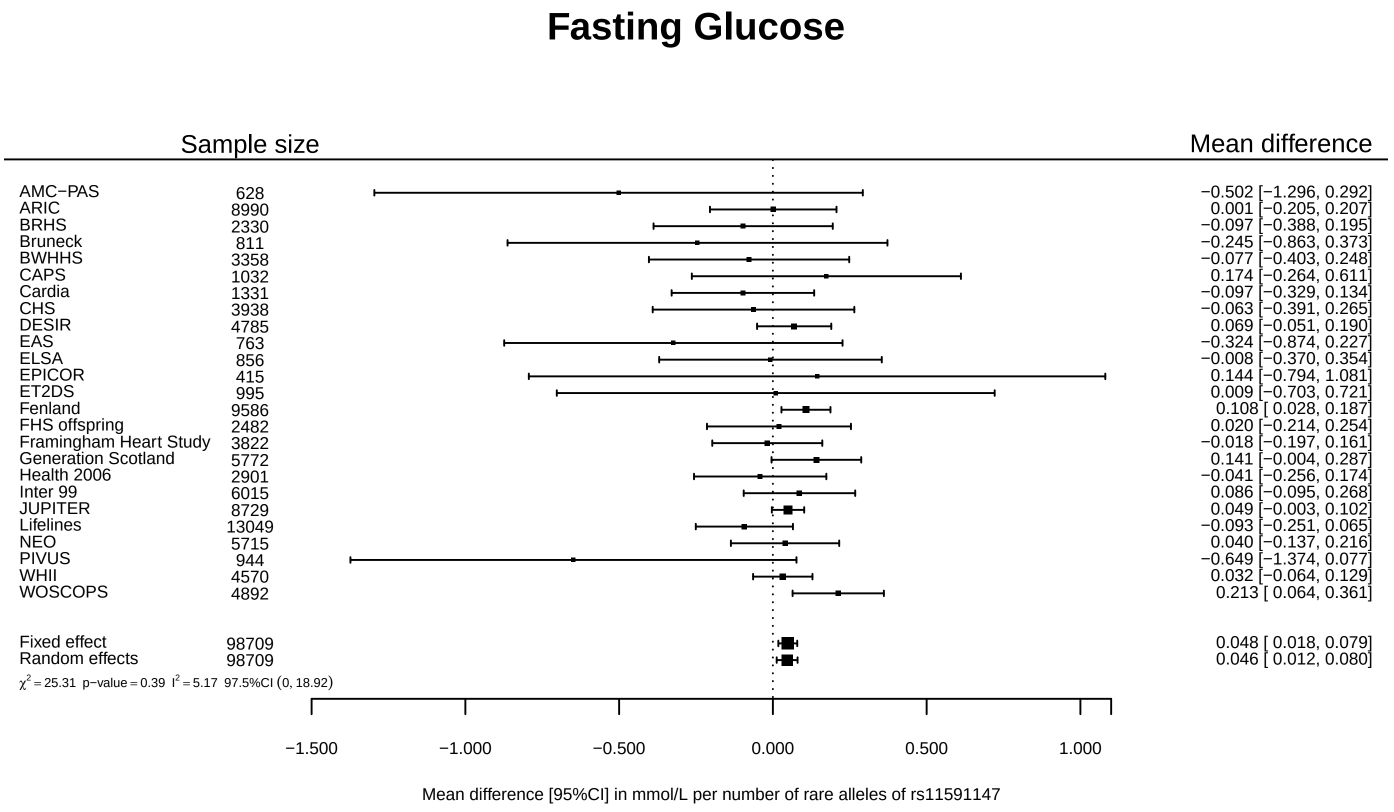

Figure 14: Mean difference of PCSK9 SNP rs11591147 on fasting glucose (mmol/L) per number of rare alleles, with 95% confidence interval (CI). Results are pooled using a fixed and random effects model, irrespective of observed between study heterogeneity; measured as a two sided Q–test (χ^2) and the I^2 with one–sided 97.5% CI.

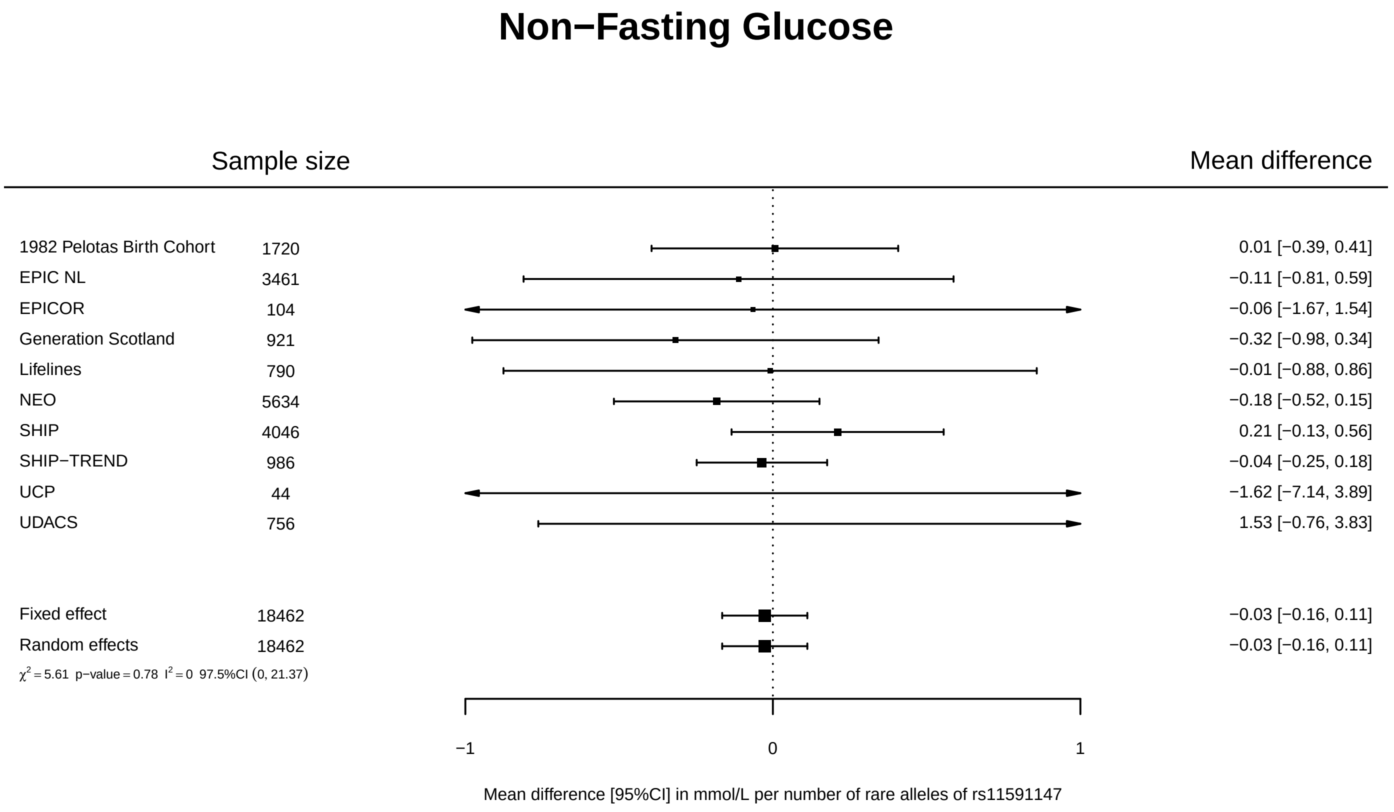

Figure 15: Mean difference of PCSK9 SNP rs11591147 on non–fasting glucose (mmol/L) per number of rare alleles, with 95% confidence interval (CI). Results are pooled using a fixed and random effects model, irrespective of observed between study heterogeneity; measured as a two sided Q–test (χ^2) and the I^2 with one–sided 97.5% CI.

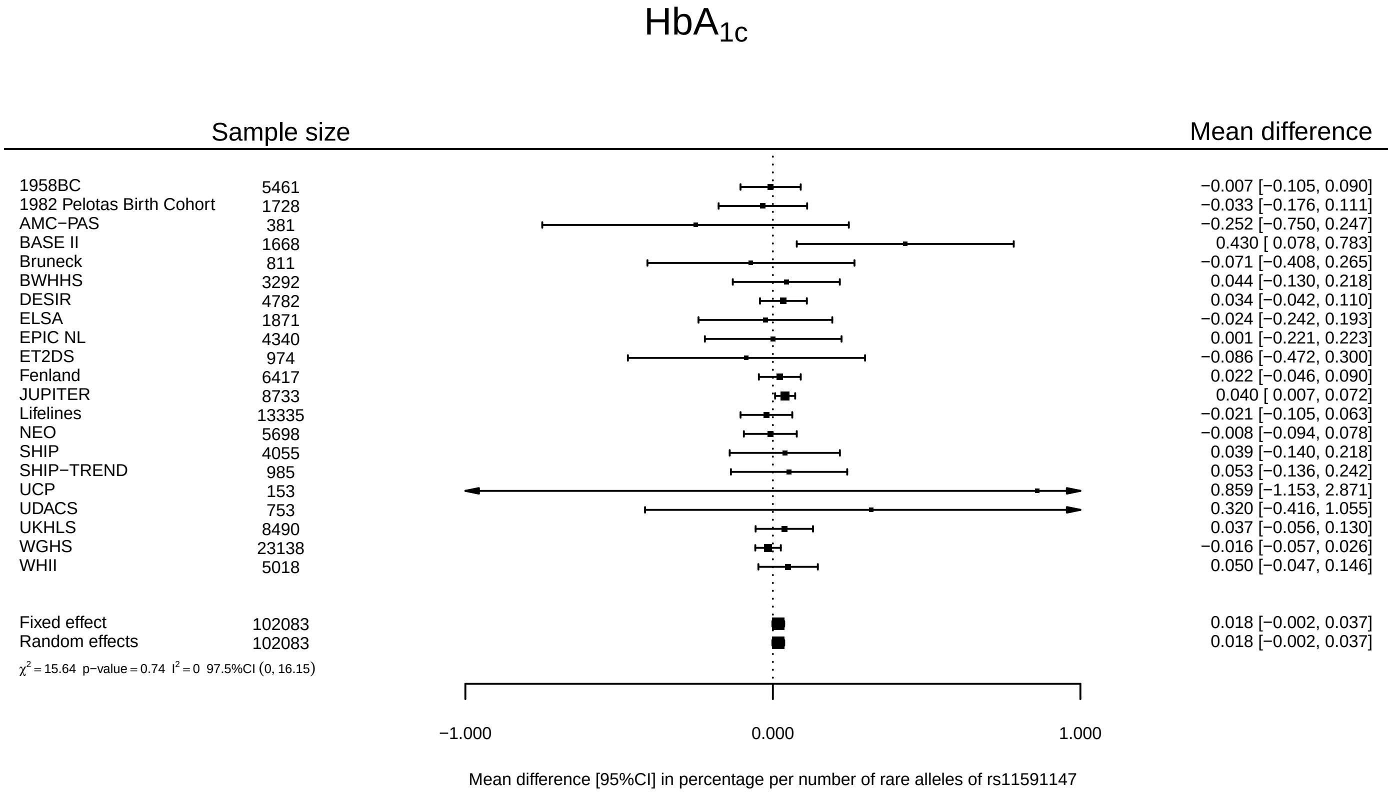

Figure 16: Mean difference of PCSK9 SNP rs11591147 on HbA_{1c} (percentage) per number of rare alleles, with 95% confidence interval (CI). Results are pooled using a fixed and random effects model, irrespective of observed between study heterogeneity; measured as a two sided Q–test (χ^2) and the I^2 with one–sided 97.5% CI.

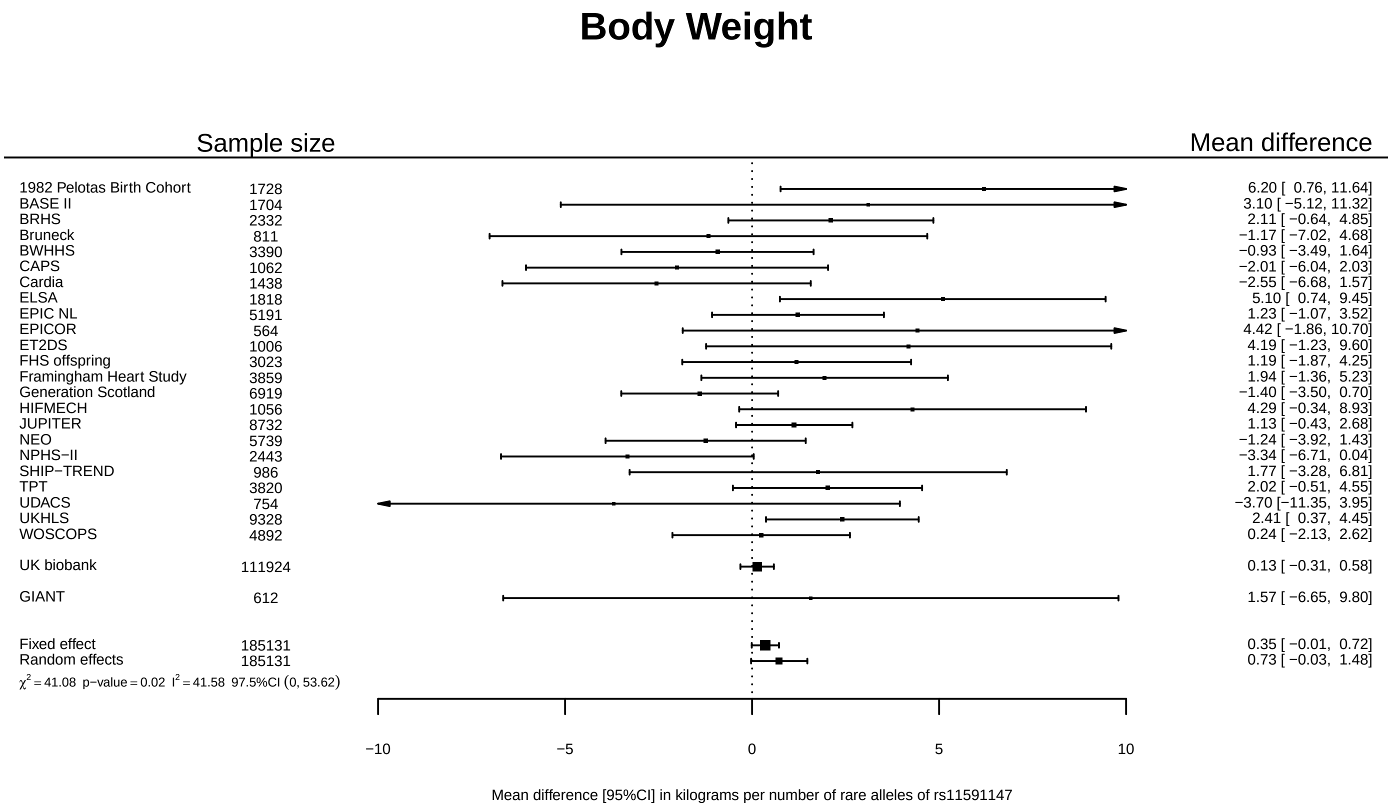

Figure 17: Mean difference of PCSK9 SNP rs11591147 on body weight (kilogram) per number of rare alleles, with 95% confidence interval (CI). Results are pooled using a fixed and random effects model, irrespective of observed between study heterogeneity; measured as a two sided Q–test (χ^2) and the I^2 with one–sided 97.5% CI.

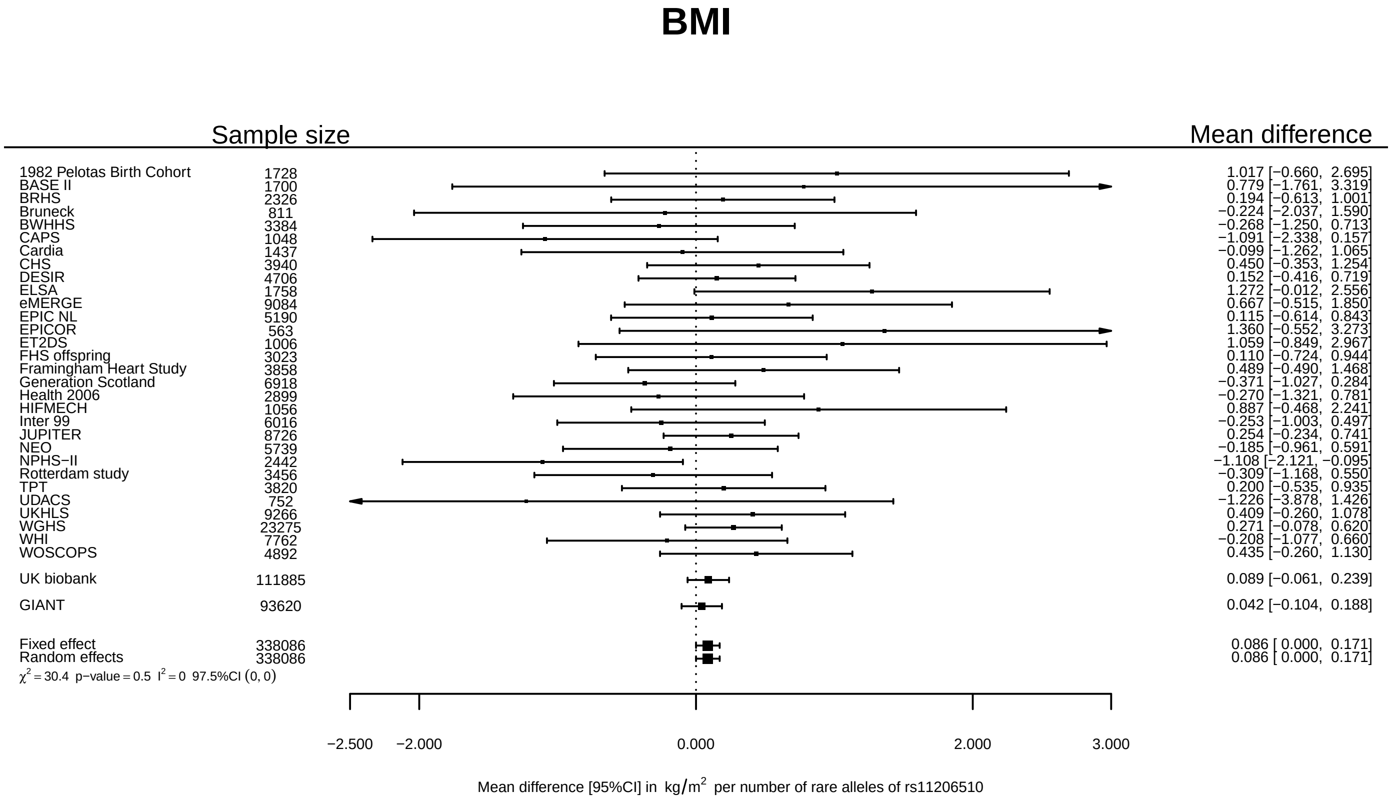

Figure 18: Mean difference of PCSK9 SNP rs11591147 on BMI (kg/m^2) per number of rare alleles, with 95% confidence interval (CI). Results are pooled using a fixed and random effects model, irrespective of observed between study heterogeneity; measured as a two sided Q–test (χ^2) and the I^2 with one–sided 97.5% CI.

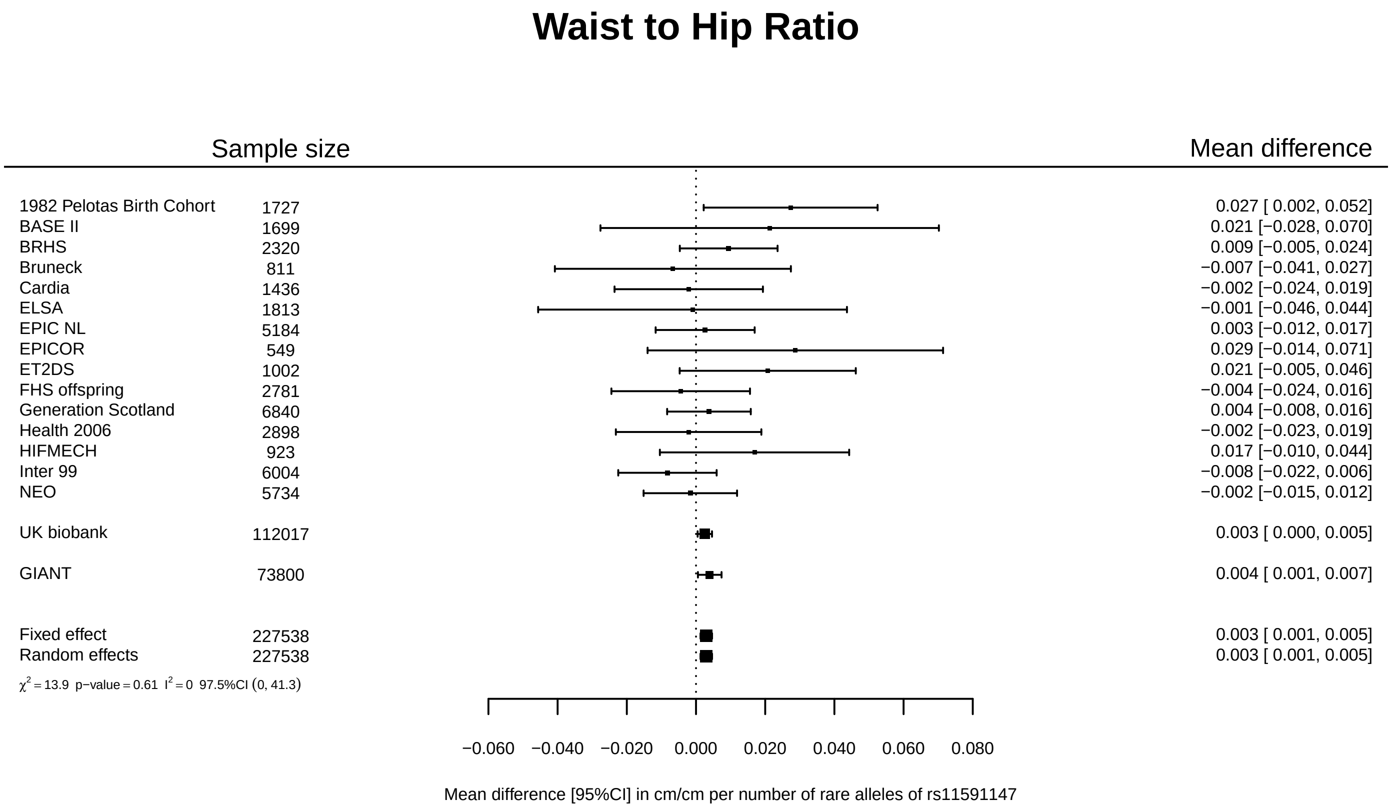

Figure 19: Mean difference of PCSK9 SNP rs11591147 on waist to hip ratio per number of rare alleles, with 95% confidence interval (CI). Results are pooled using a fixed and random effects model, irrespective of observed between study heterogeneity; measured as a two sided Q–test (χ^2) and the I^2 with one–sided 97.5% CI.

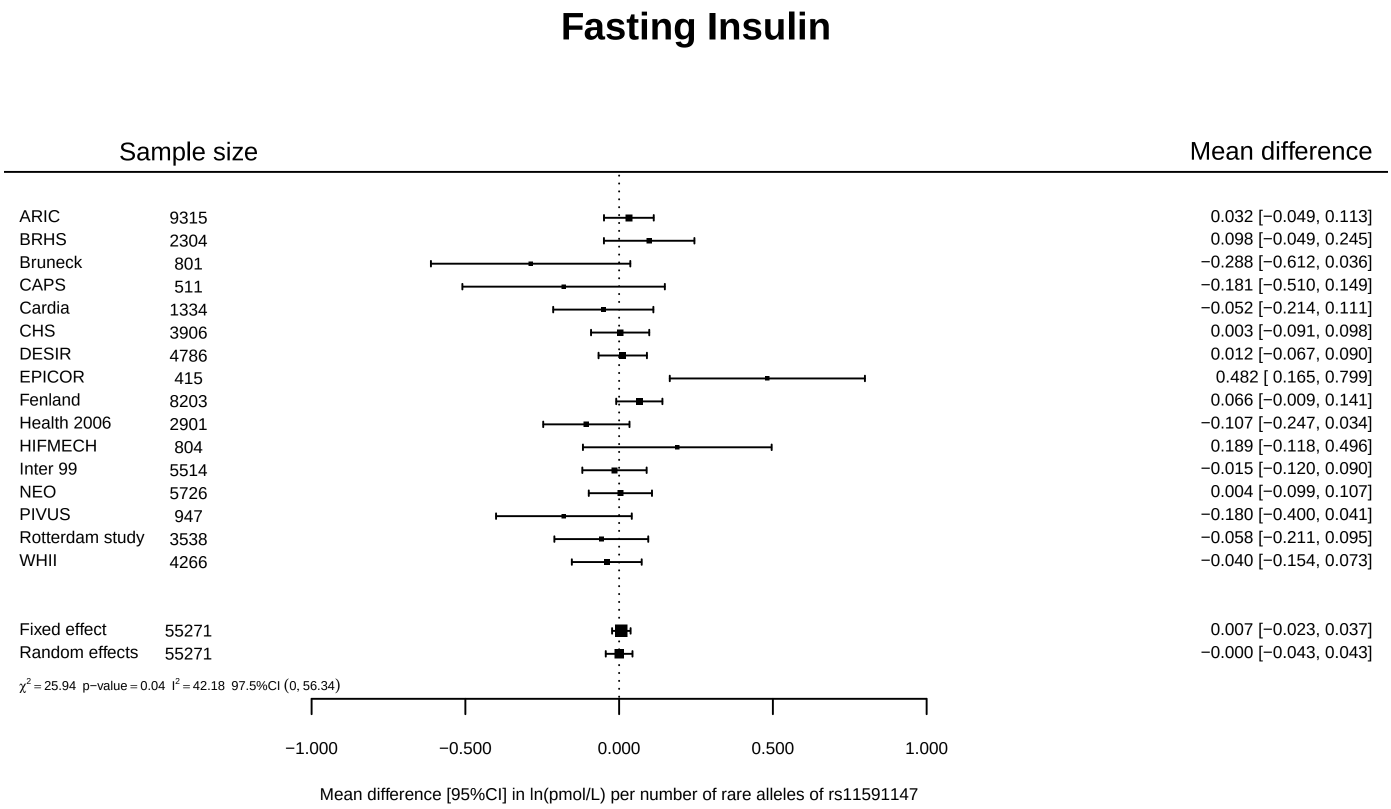

Figure 20: Mean difference of PCSK9 SNP rs11591147 on fasting insulin $ln(pmol/L)$ per number of rare alleles, with 95% confidence interval (CI). Results are pooled using a fixed and random effects model, irrespective of observed between study heterogeneity; measured as a two sided Q–test (χ^2) and the I^2 with one–sided 97.5% CI.

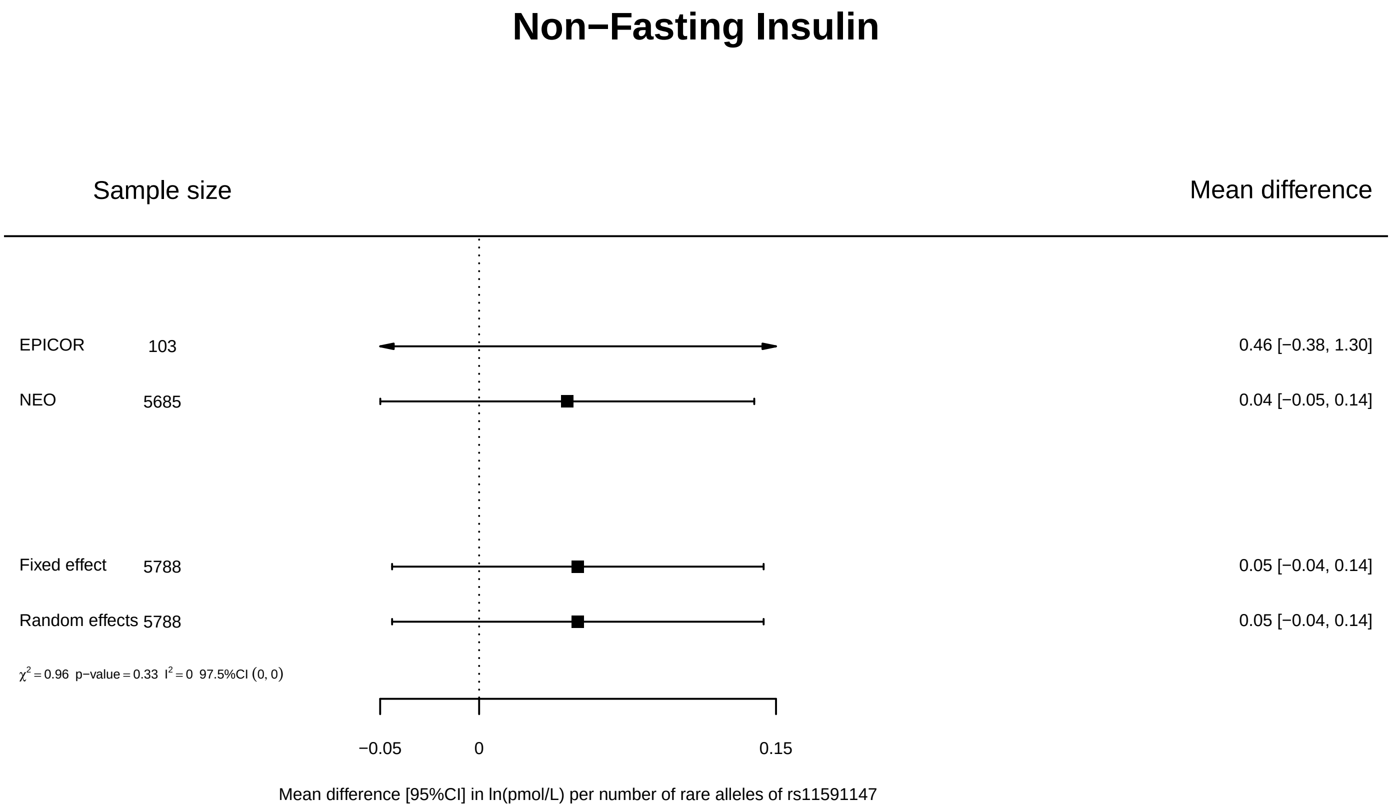

Figure 21: Mean difference of PCSK9 SNP rs11591147 on non–fasting insulin $ln(pmol/L)$ per number of rare alleles, with 95% confidence interval (CI). Results are pooled using a fixed and random effects model, irrespective of observed between study heterogeneity; measured as a two sided Q–test (χ^2) and the I^2 with one–sided 97.5% CI.

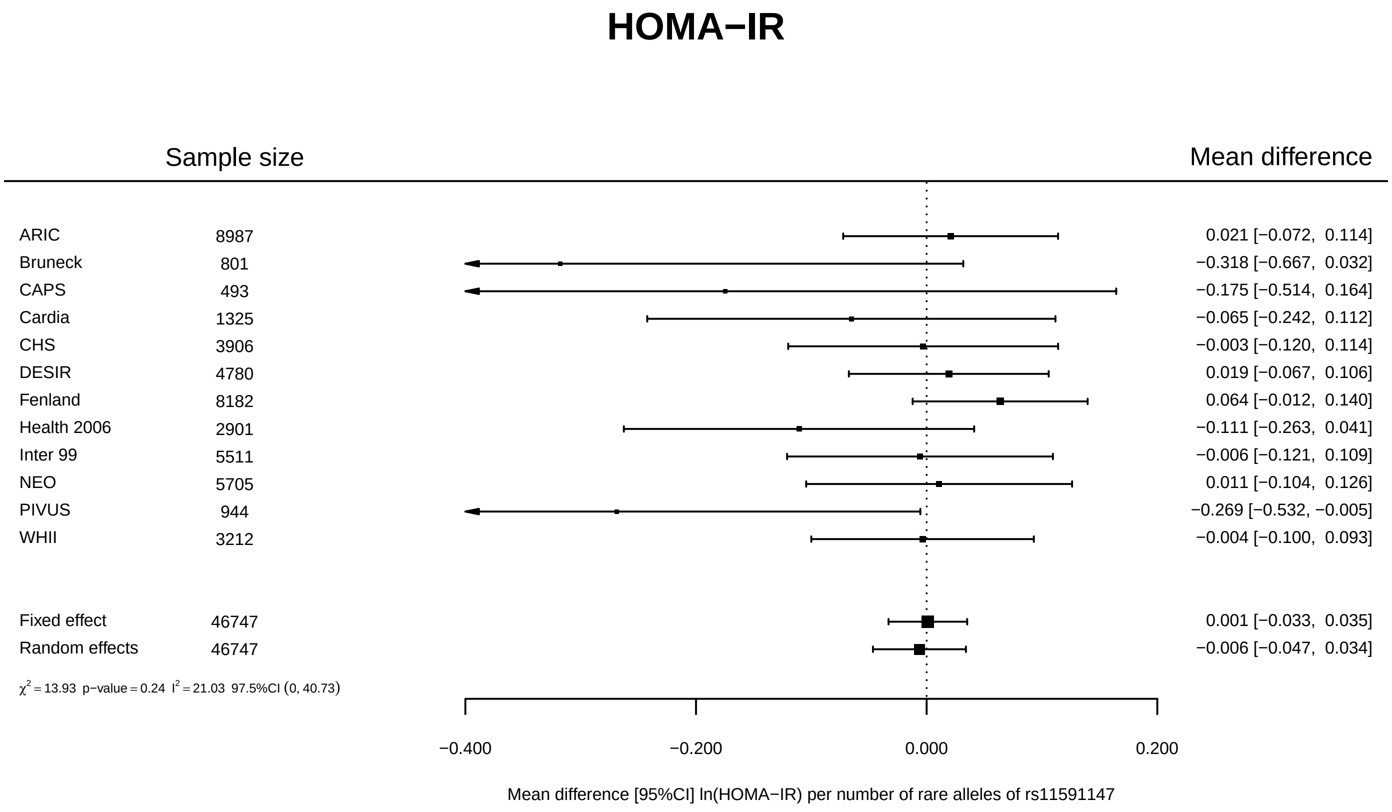

Figure 22: Mean difference of PCSK9 SNP rs11591147 on $ln(HOMA\check{}IR)$ per number of rare alleles, with 95% confidence interval (CI). Results are pooled using a fixed and random effects model, irrespective of observed between study heterogeneity; measured as a two sided Q–test (χ^2) and the I^2 with one–sided 97.5% CI.

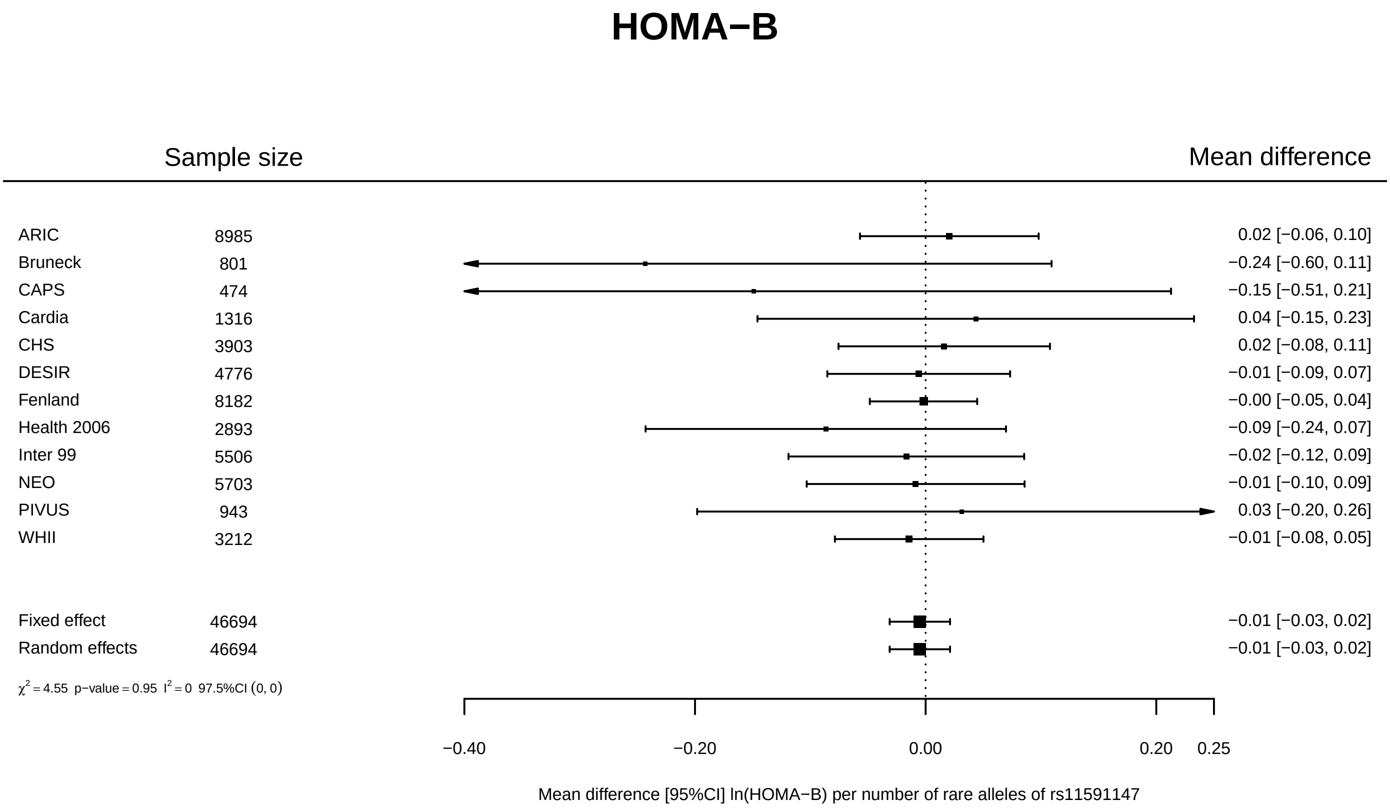

Figure 23: Mean difference of PCSK9 SNP rs11591147 on $ln(HOMA\breve{\ }\beta)$ per number of rare alleles, with 95% confidence interval (CI). Results are pooled using a fixed and random effects model, irrespective of observed between study heterogeneity; measured as a two sided Q–test (χ^2) and the I^2 with one–sided 97.5% CI.

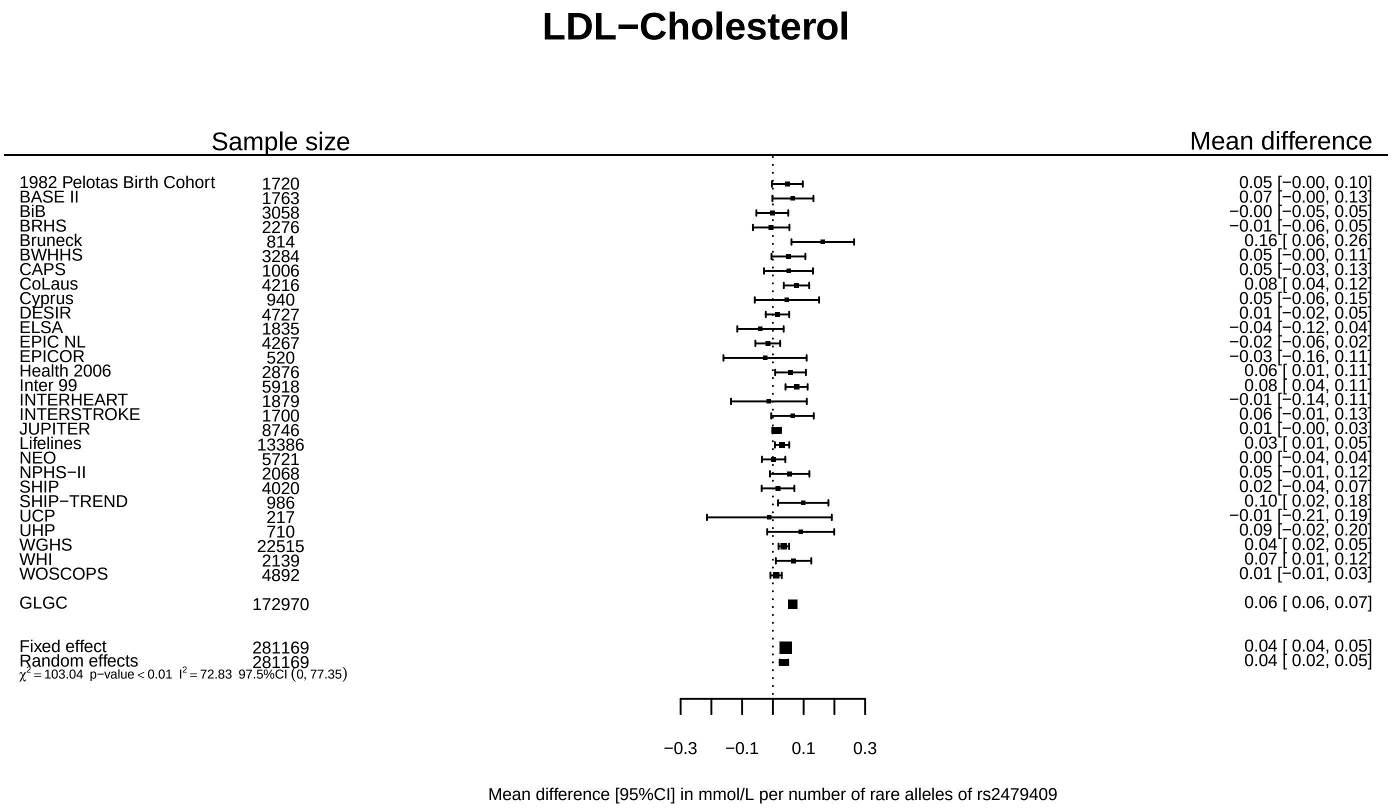

Figure 24: Mean difference of PCSK9 SNP rs2479409 on LDL–C (mmol/L) per number of rare alleles, with 95% confidence interval (CI). Results are pooled using a fixed and random effects model, irrespective of observed between study heterogeneity; measured as a two sided Q–test (χ^2) and the I^2 with one–sided 97.5% CI.

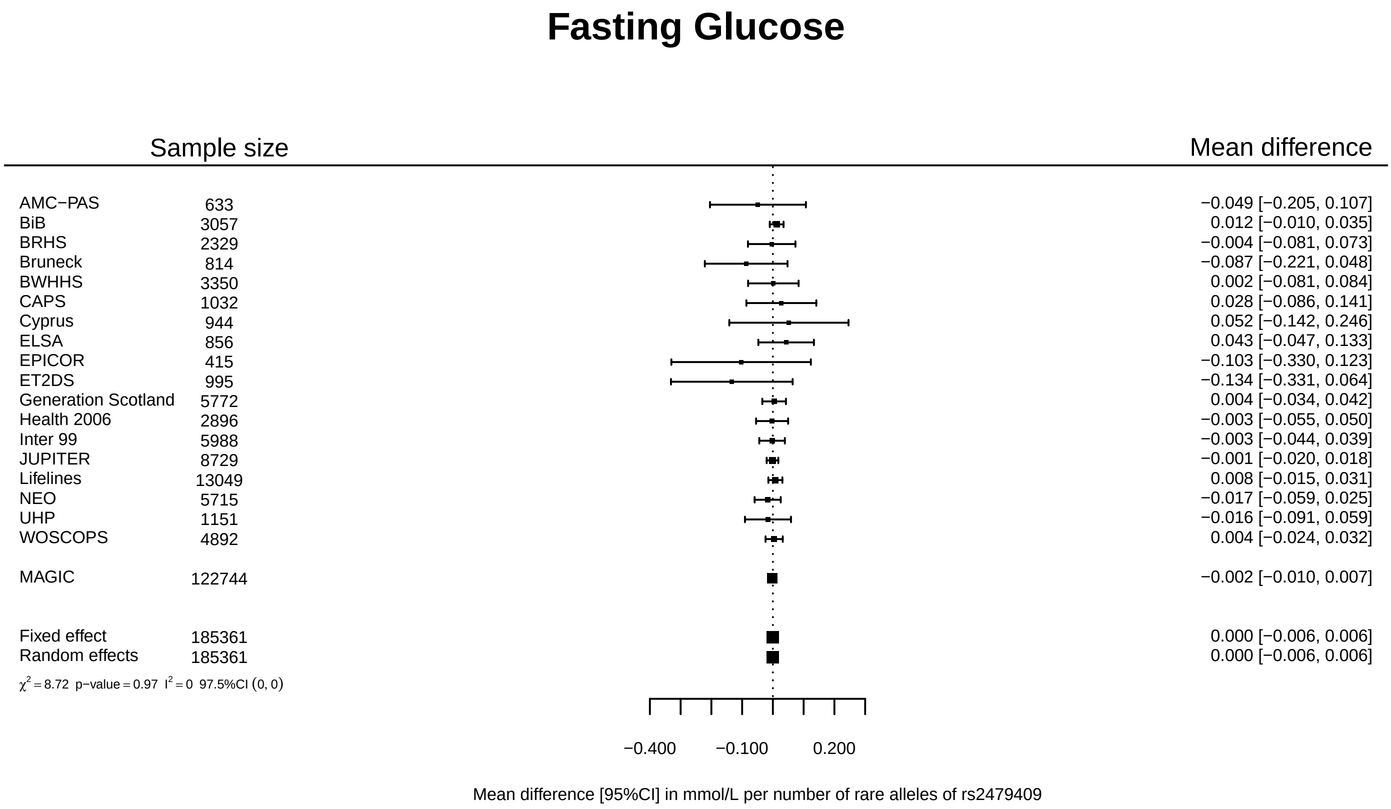

Figure 25: Mean difference of PCSK9 SNP rs2479409 on fasting glucose (mmol/L) per number of rare alleles, with 95% confidence interval (CI). Results are pooled using a fixed and random effects model, irrespective of observed between study heterogeneity; measured as a two sided Q–test (χ^2) and the I^2 with one–sided 97.5% CI.

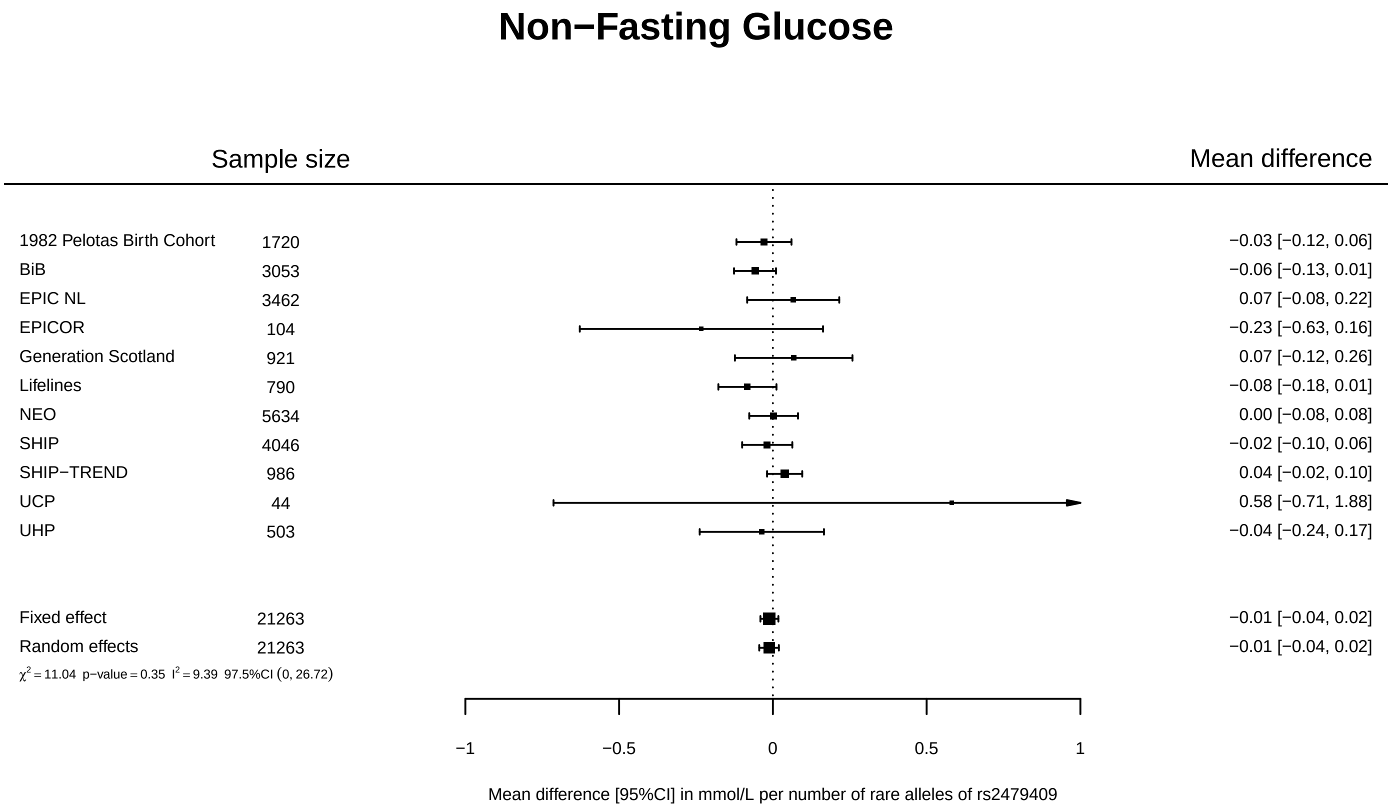

Figure 26: Mean difference of PCSK9 SNP rs2479409 on non–fasting glucose (mmol/L) per number of rare alleles, with 95% confidence interval (CI). Results are pooled using a fixed and random effects model, irrespective of observed between study heterogeneity; measured as a two sided Q–test (χ^2) and the I^2 with one–sided 97.5% CI.

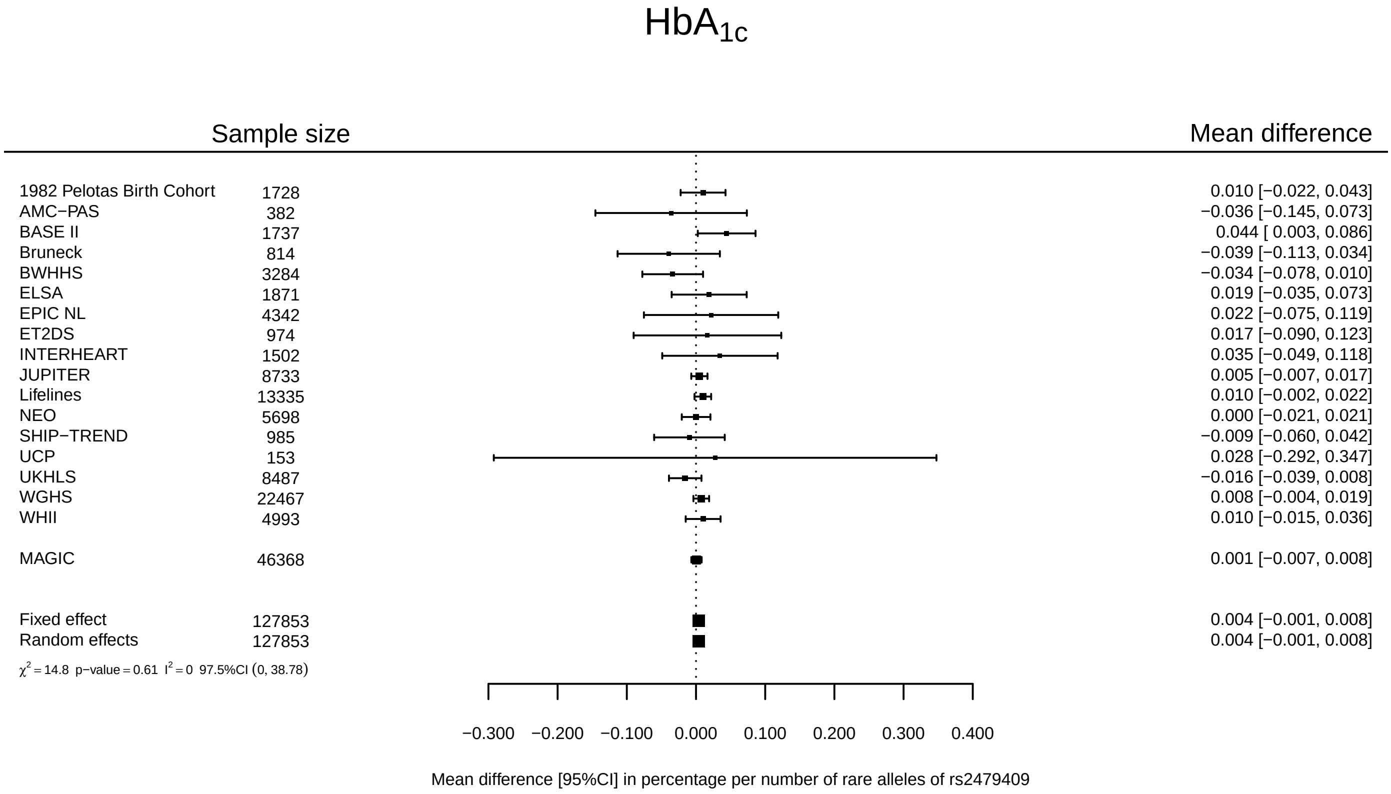

Figure 27: Mean difference of PCSK9 SNP rs2479409 on HbA_{1c} (percentage) per number of rare alleles, with 95% confidence interval (CI). Results are pooled using a fixed and random effects model, irrespective of observed between study heterogeneity; measured as a two sided Q–test (χ^2) and the I^2 with one–sided 97.5% CI.

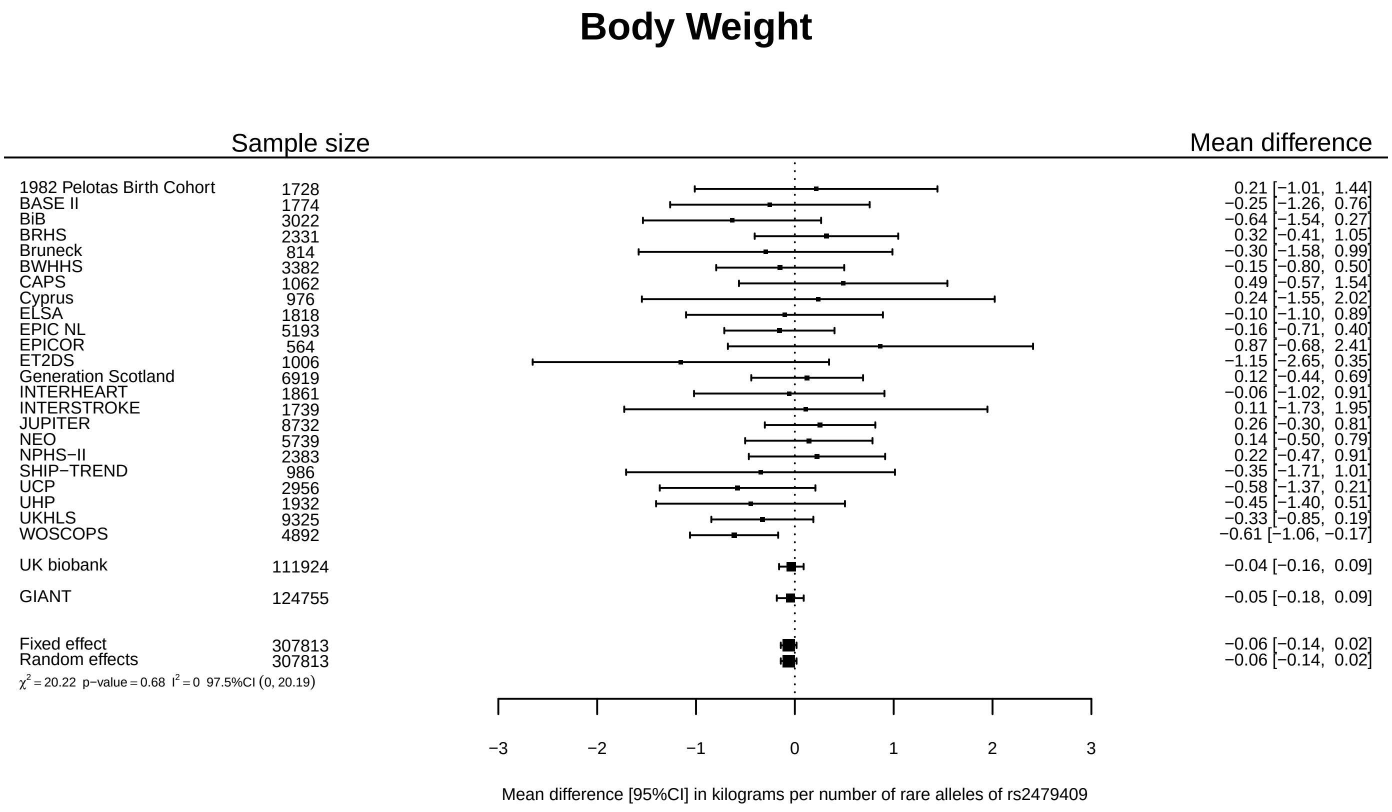

Figure 28: Mean difference of PCSK9 SNP rs2479409 on body weight (kilogram) per number of rare alleles, with 95% confidence interval (CI). Results are pooled using a fixed and random effects model, irrespective of observed between study heterogeneity; measured as a two sided Q–test (χ^2) and the I^2 with one–sided 97.5% CI.

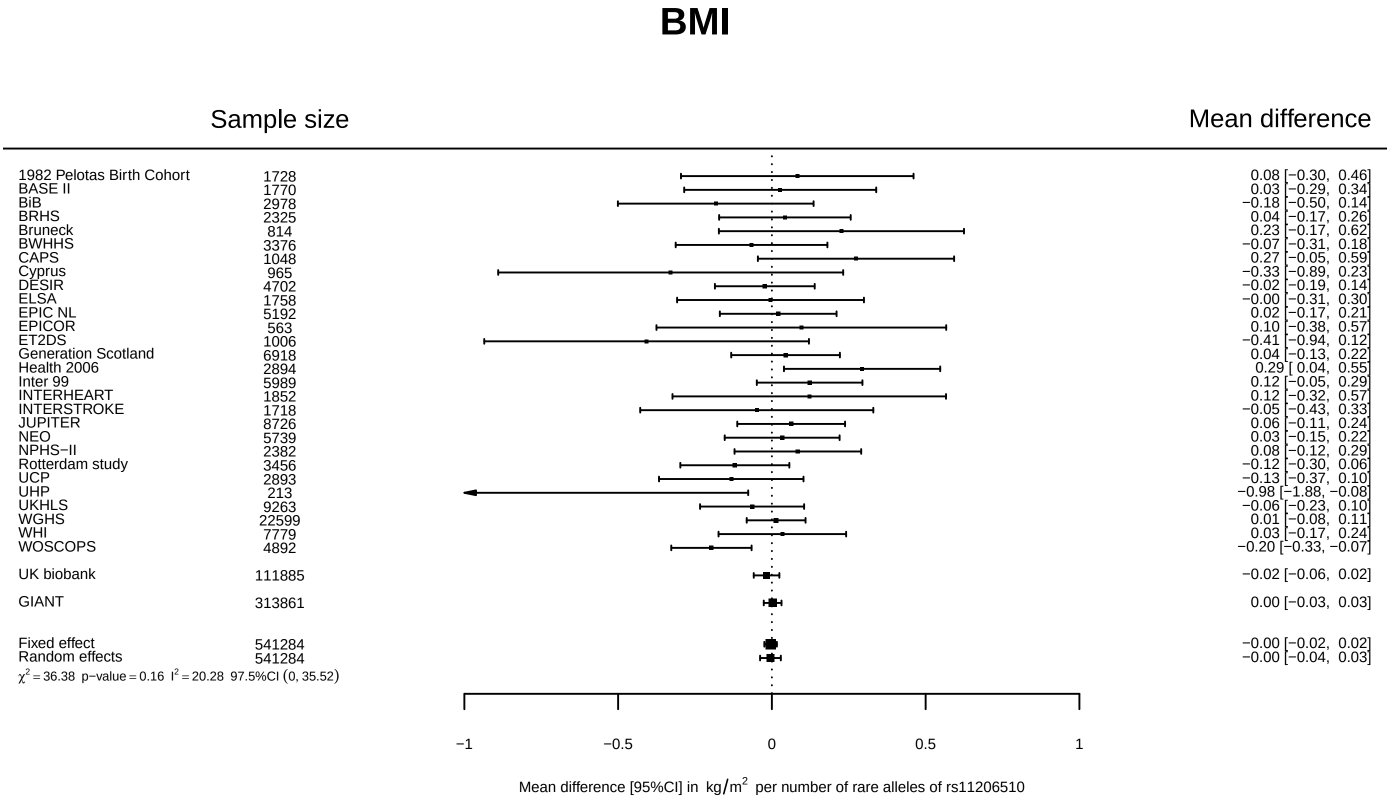

Figure 29: Mean difference of PCSK9 SNP rs2479409 on BMI (kg/m^2) per number of rare alleles, with 95% confidence interval (CI). Results are pooled using a fixed and random effects model, irrespective of observed between study heterogeneity; measured as a two sided Q–test (χ^2) and the I^2 with one–sided 97.5% CI.

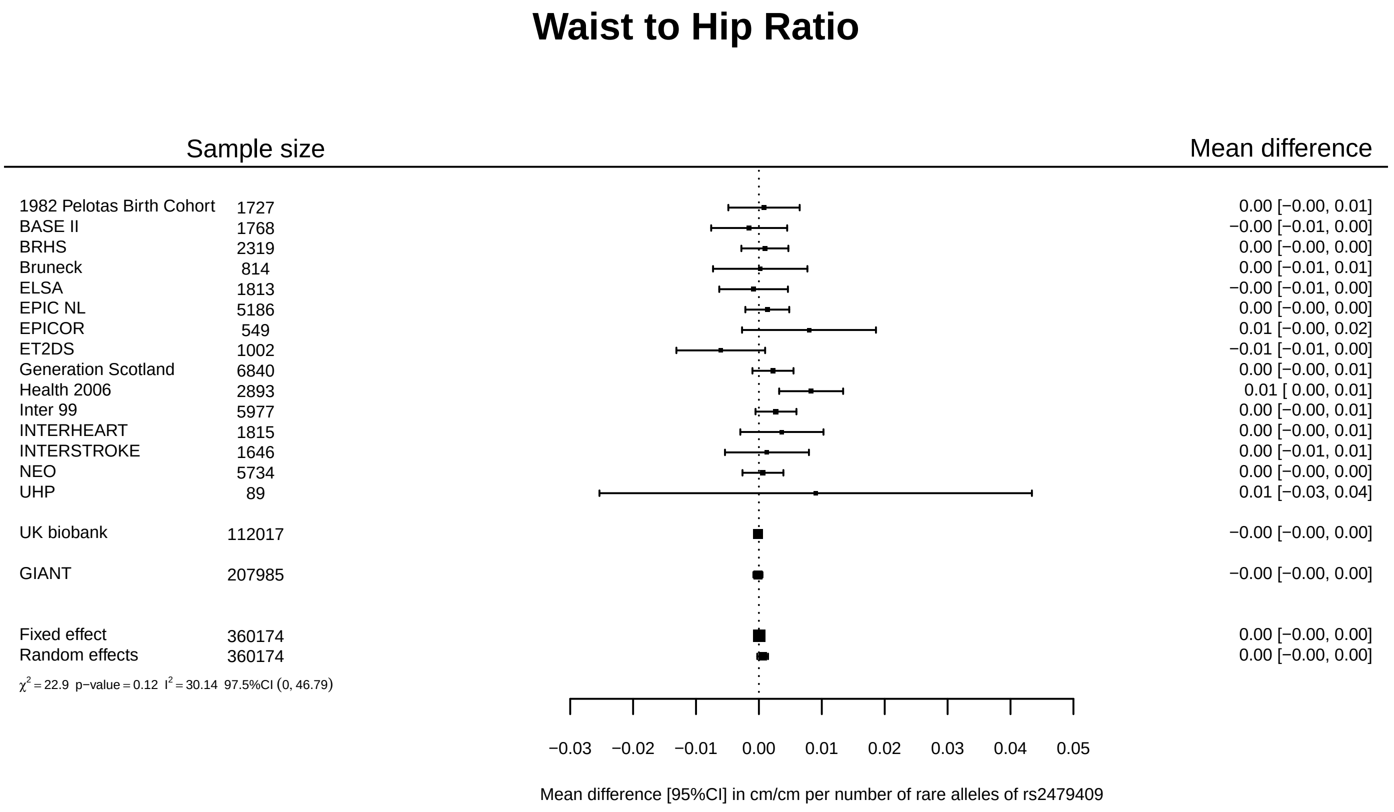

Figure 30: Mean difference of PCSK9 SNP rs2479409 on waist to hip ratio per number of rare alleles, with 95% confidence interval (CI). Results are pooled using a fixed and random effects model, irrespective of observed between study heterogeneity; measured as a two sided Q–test (χ^2) and the I^2 with one–sided 97.5% CI.

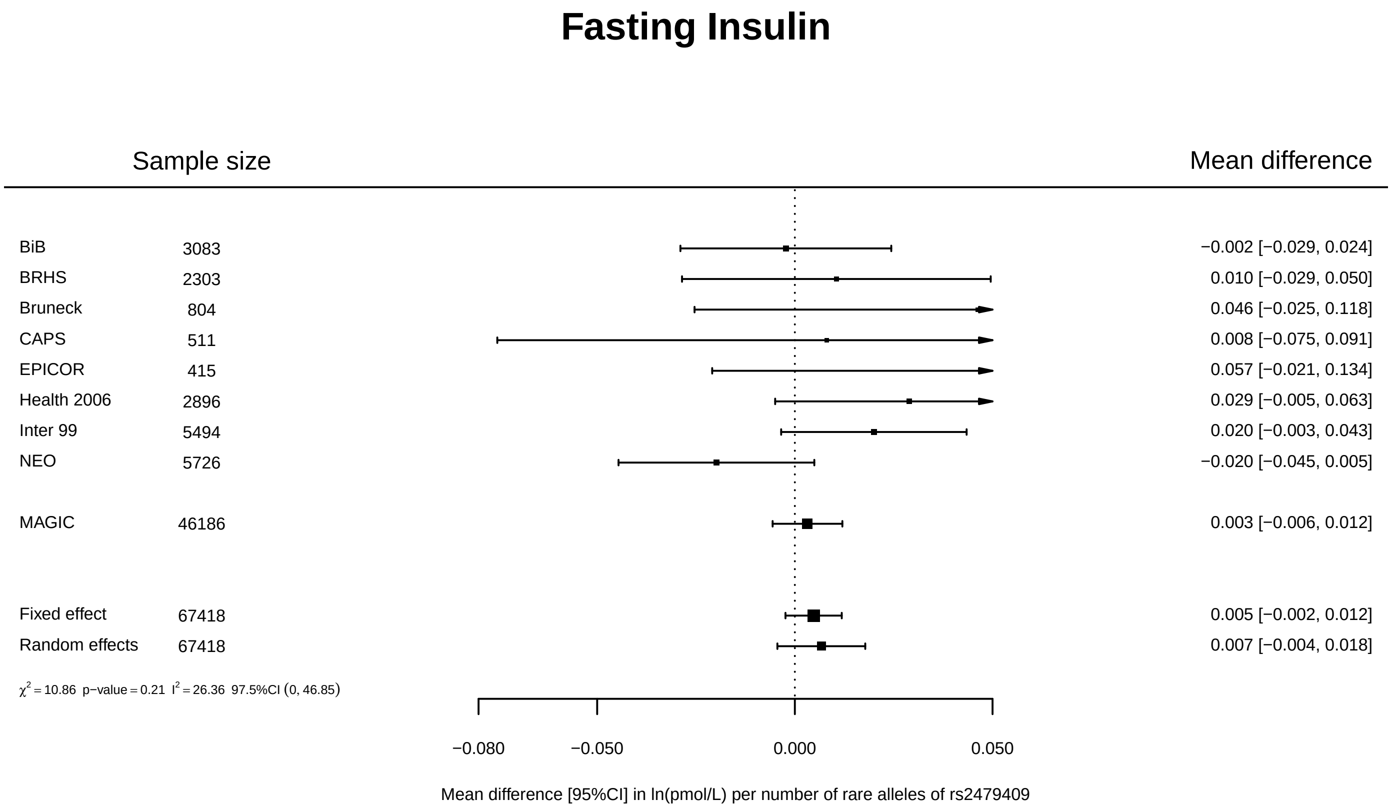

Figure 31: Mean difference of PCSK9 SNP rs2479409 on fasting insulin $ln(pmol/L)$ per number of rare alleles, with 95% confidence interval (CI). Results are pooled using a fixed and random effects model, irrespective of observed between study heterogeneity; measured as a two sided Q–test (χ^2) and the I^2 with one–sided 97.5% CI.

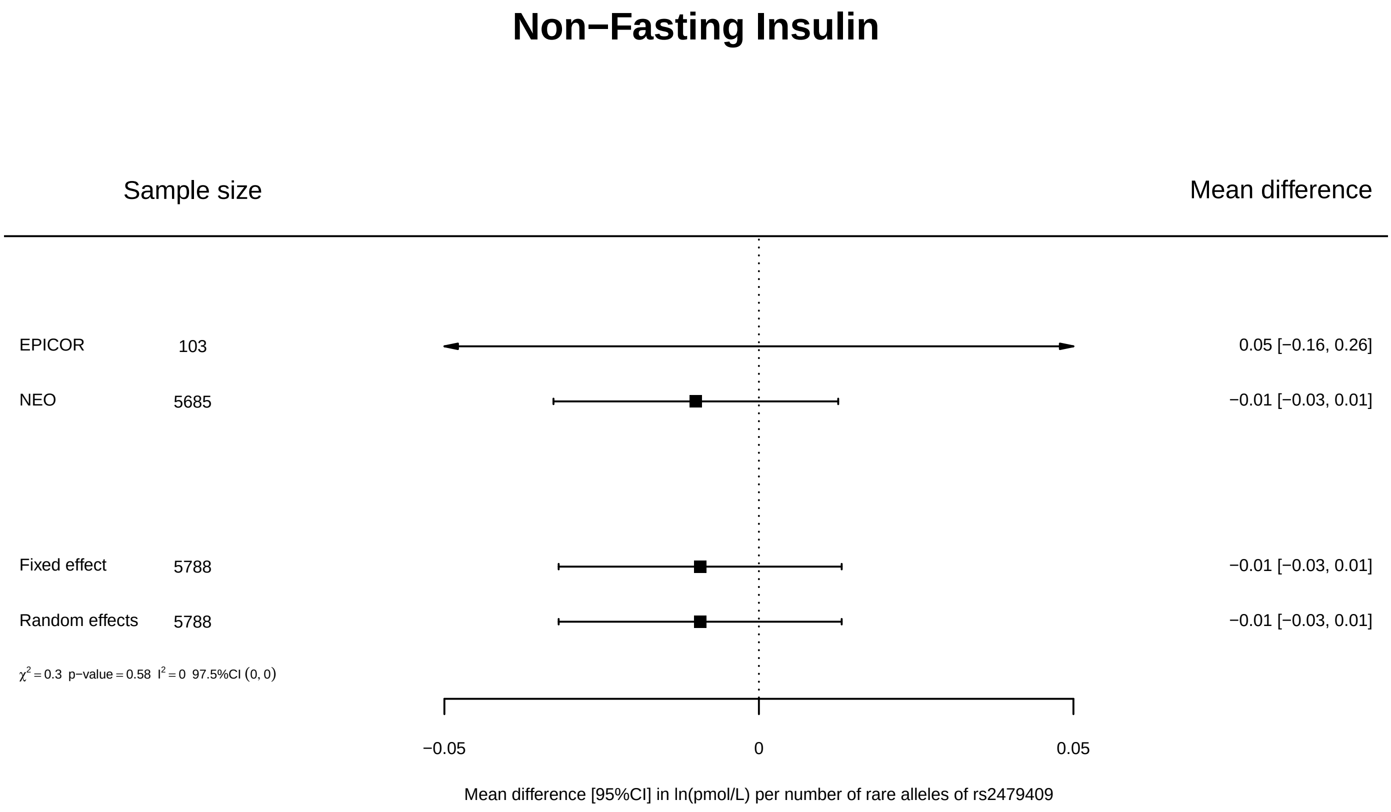

Figure 32: Mean difference of PCSK9 SNP rs2479409 on non–fasting insulin $ln(pmol/L)$ per number of rare alleles, with 95% confidence interval (CI). Results are pooled using a fixed and random effects model, irrespective of observed between study heterogeneity; measured as a two sided Q–test (χ^2) and the I^2 with one–sided 97.5% CI.

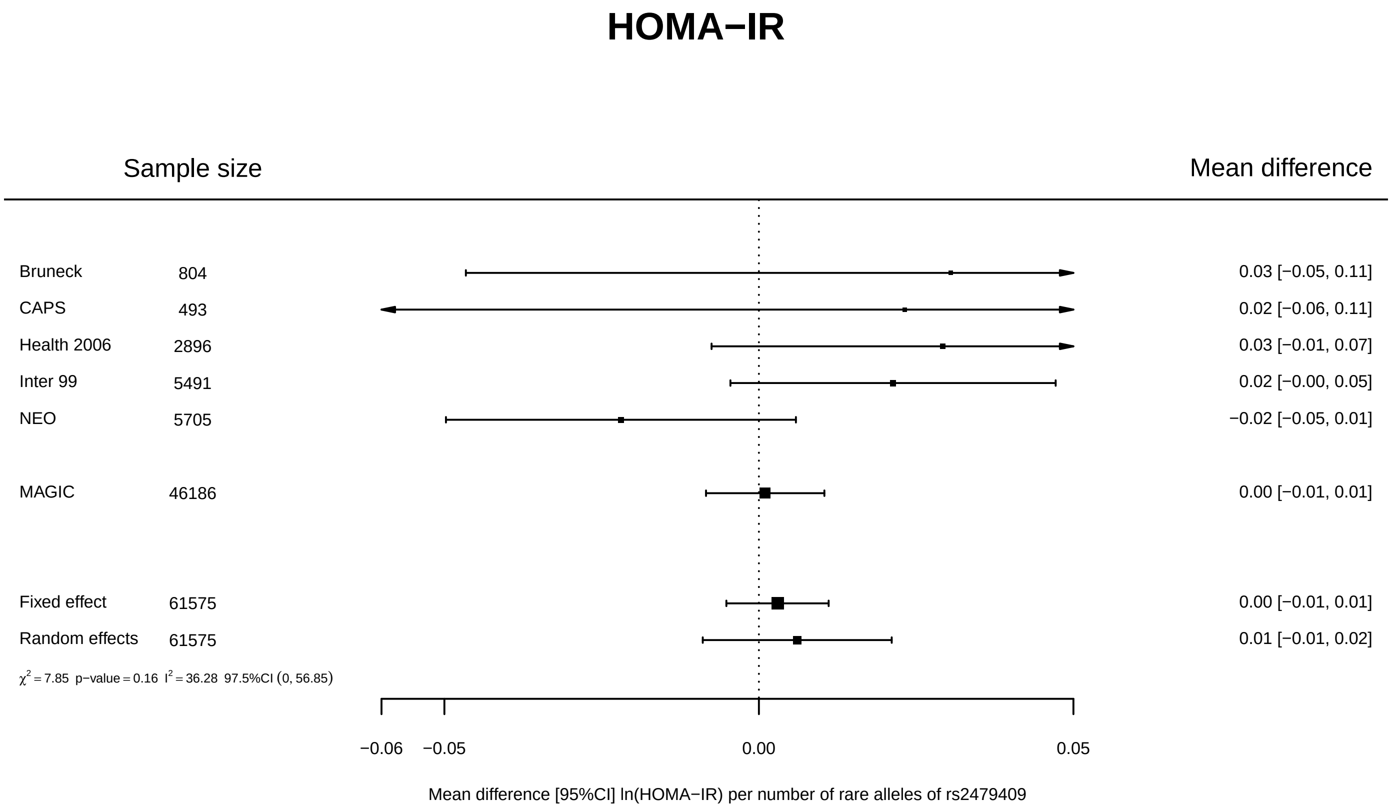

Figure 33: Mean difference of PCSK9 SNP rs2479409 on $ln(HOMA\check{}IR)$ per number of rare alleles, with 95% confidence interval (CI). Results are pooled using a fixed and random effects model, irrespective of observed between study heterogeneity; measured as a two sided Q–test (χ^2) and the I^2 with one–sided 97.5% CI.

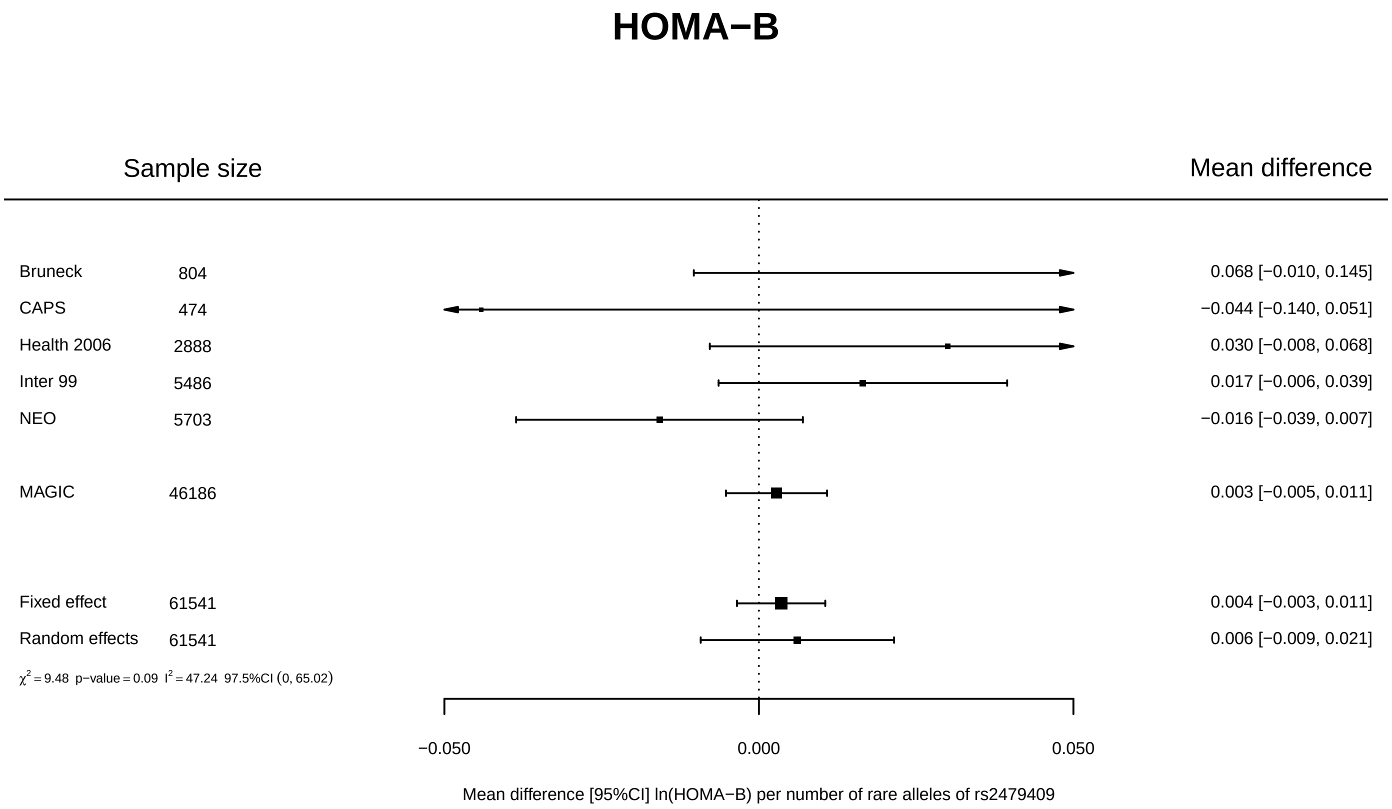

Figure 34: Mean difference of PCSK9 SNP rs2479409 on $ln(HOMA\breve{}\beta)$ per number of rare alleles, with 95% confidence interval (CI). Results are pooled using a fixed and random effects model, irrespective of observed between study heterogeneity; measured as a two sided Q–test (χ^2) and the I^2 with one–sided 97.5% CI.

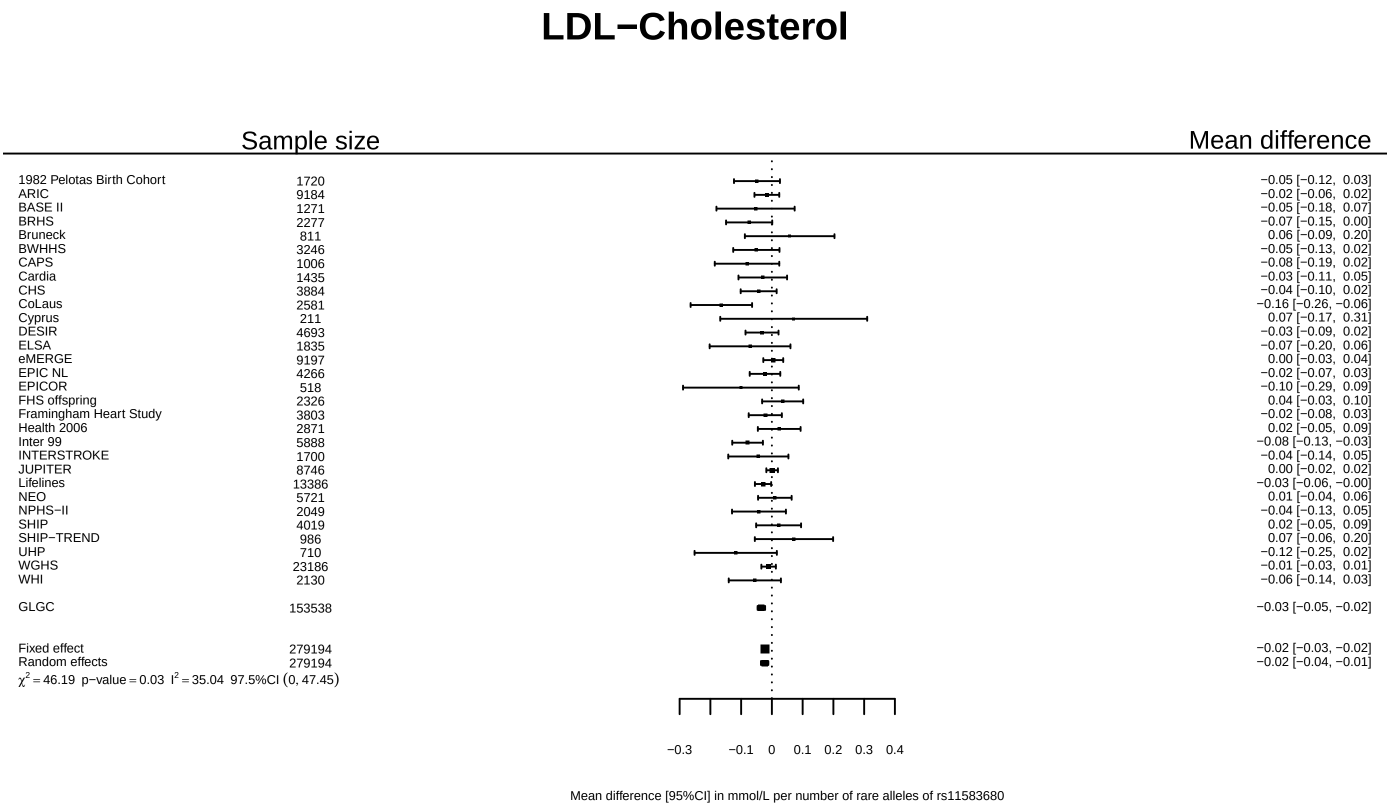

Figure 35: Mean difference of PCSK9 SNP rs11583680 on LDL–C (mmol/L) per number of rare alleles, with 95% confidence interval (CI). Results are pooled using a fixed and random effects model, irrespective of observed between study heterogeneity; measured as a two sided Q–test (χ^2) and the I^2 with one–sided 97.5% CI.

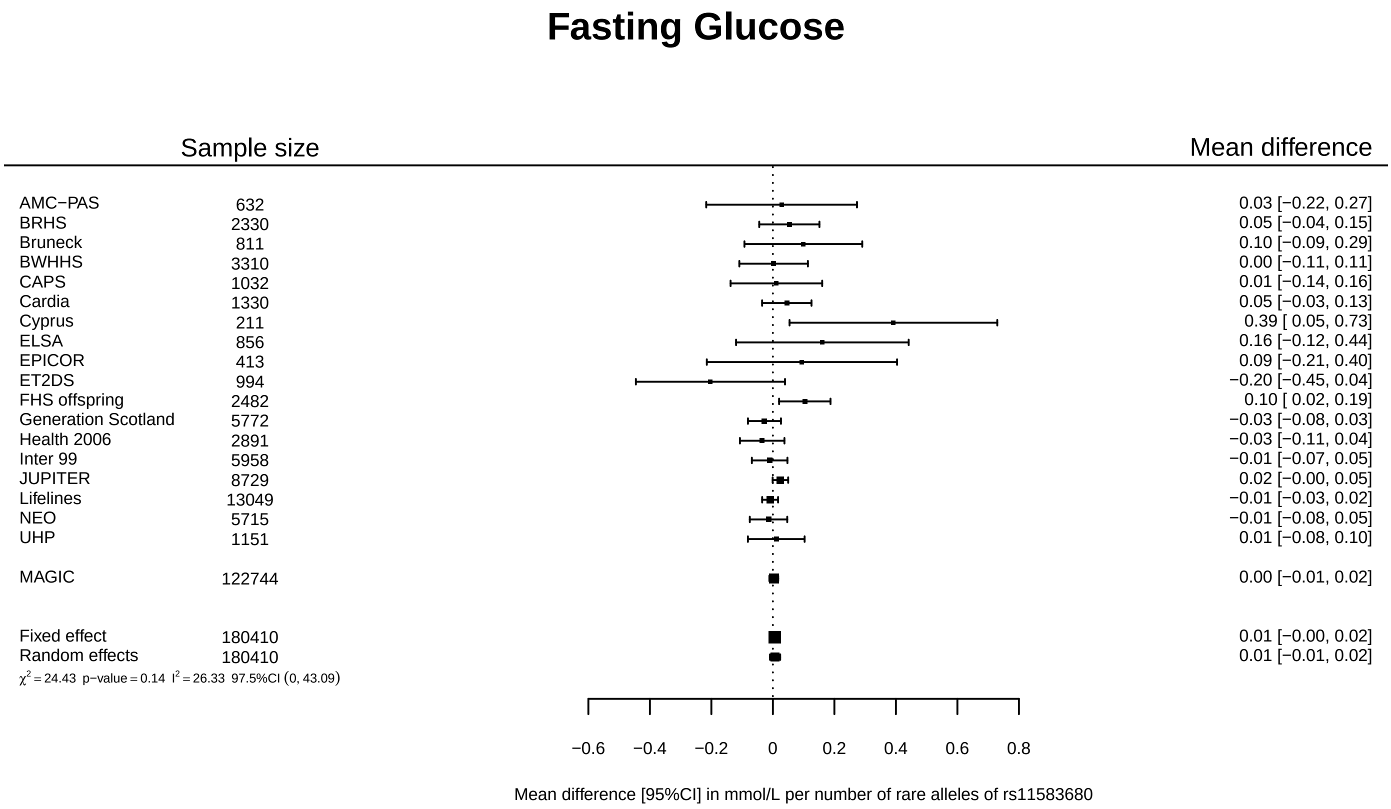

Figure 36: Mean difference of PCSK9 SNP rs11583680 on fasting glucose (mmol/L) per number of rare alleles, with 95% confidence interval (CI). Results are pooled using a fixed and random effects model, irrespective of observed between study heterogeneity; measured as a two sided Q–test (χ^2) and the I^2 with one–sided 97.5% CI.

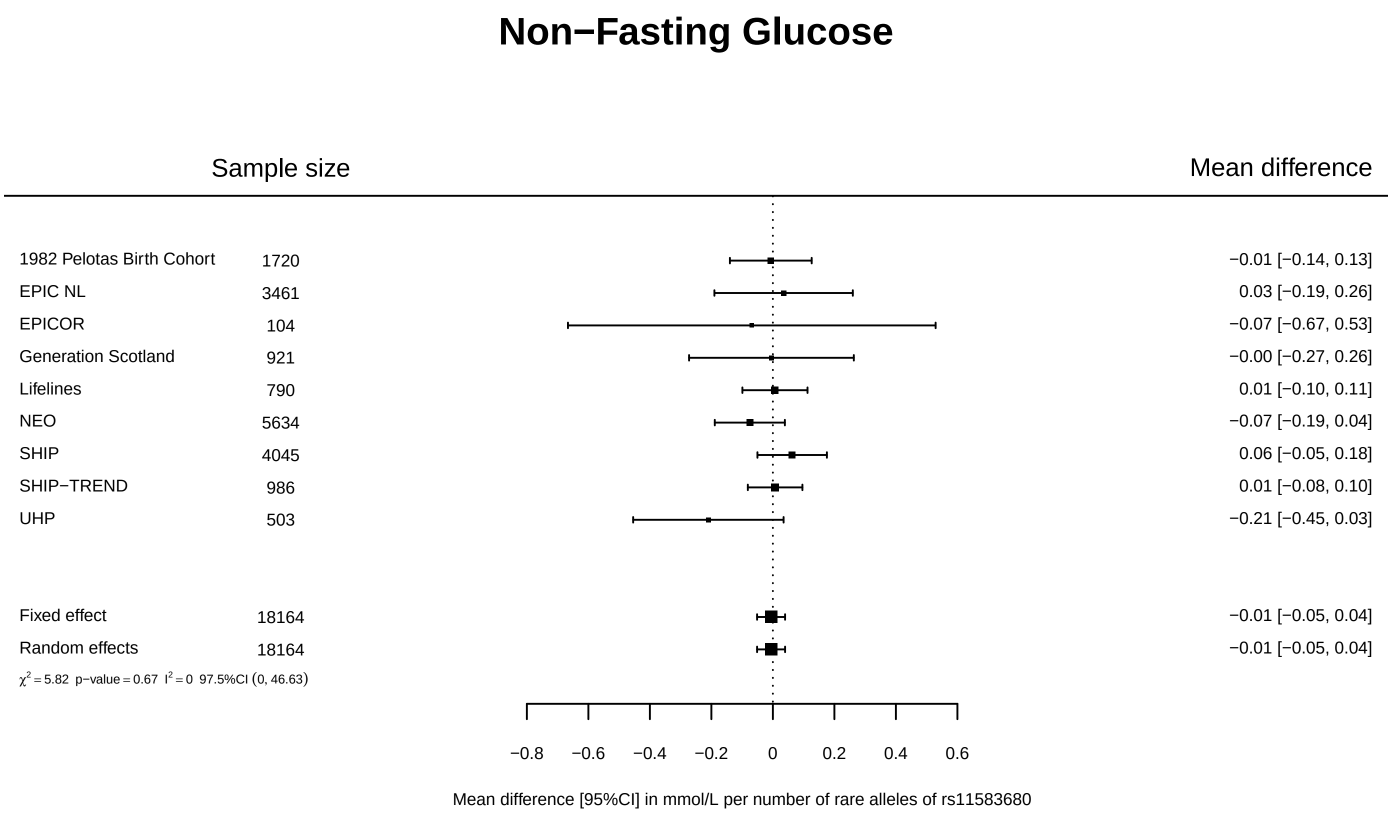

Figure 37: Mean difference of PCSK9 SNP rs11583680 on non–fasting glucose (mmol/L) per number of rare alleles, with 95% confidence interval (CI). Results are pooled using a fixed and random effects model, irrespective of observed between study heterogeneity; measured as a two sided Q–test (χ^2) and the I^2 with one–sided 97.5% CI.

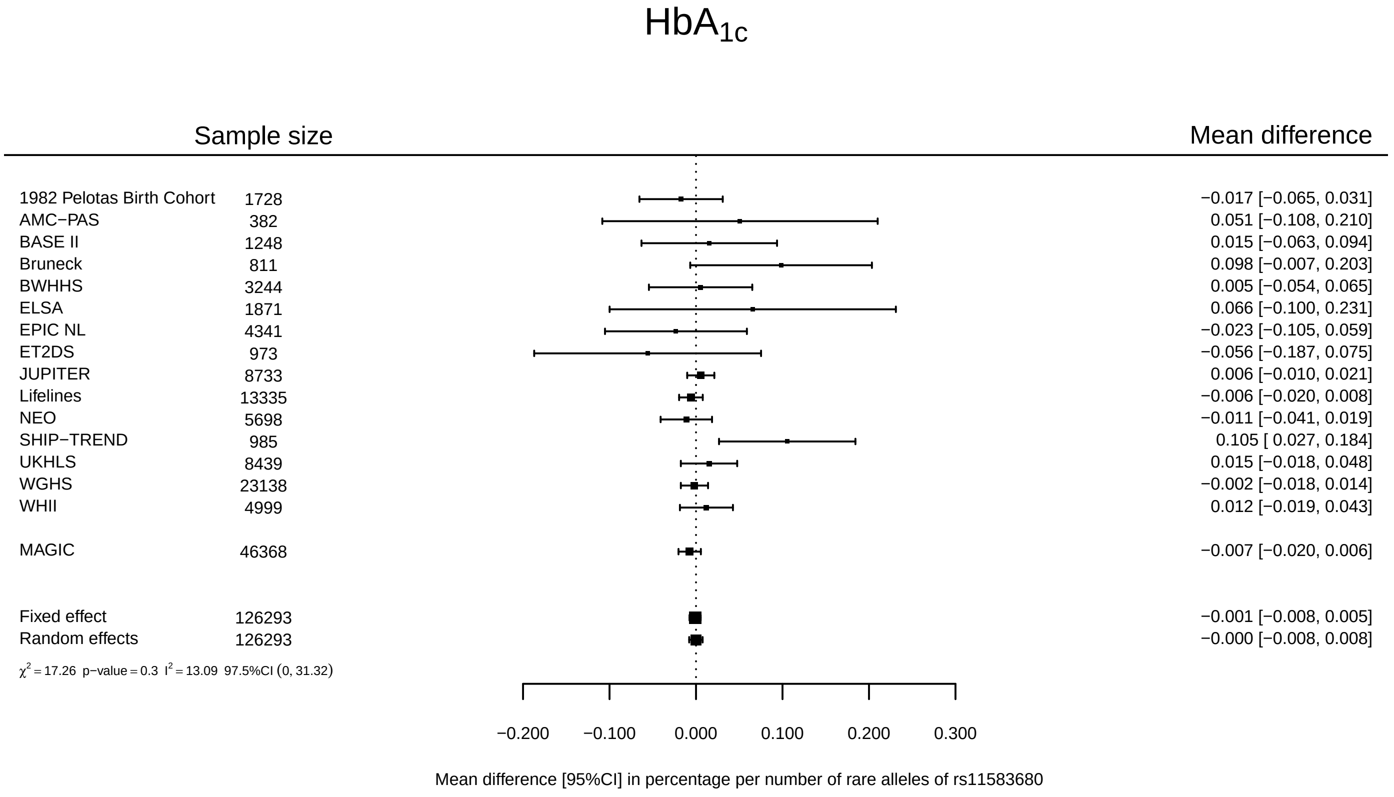

Figure 38: Mean difference of PCSK9 SNP rs11583680 on HbA_{1c} (percentage) per number of rare alleles, with 95% confidence interval (CI). Results are pooled using a fixed and random effects model, irrespective of observed between study heterogeneity; measured as a two sided Q–test (χ^2) and the I^2 with one–sided 97.5% CI.

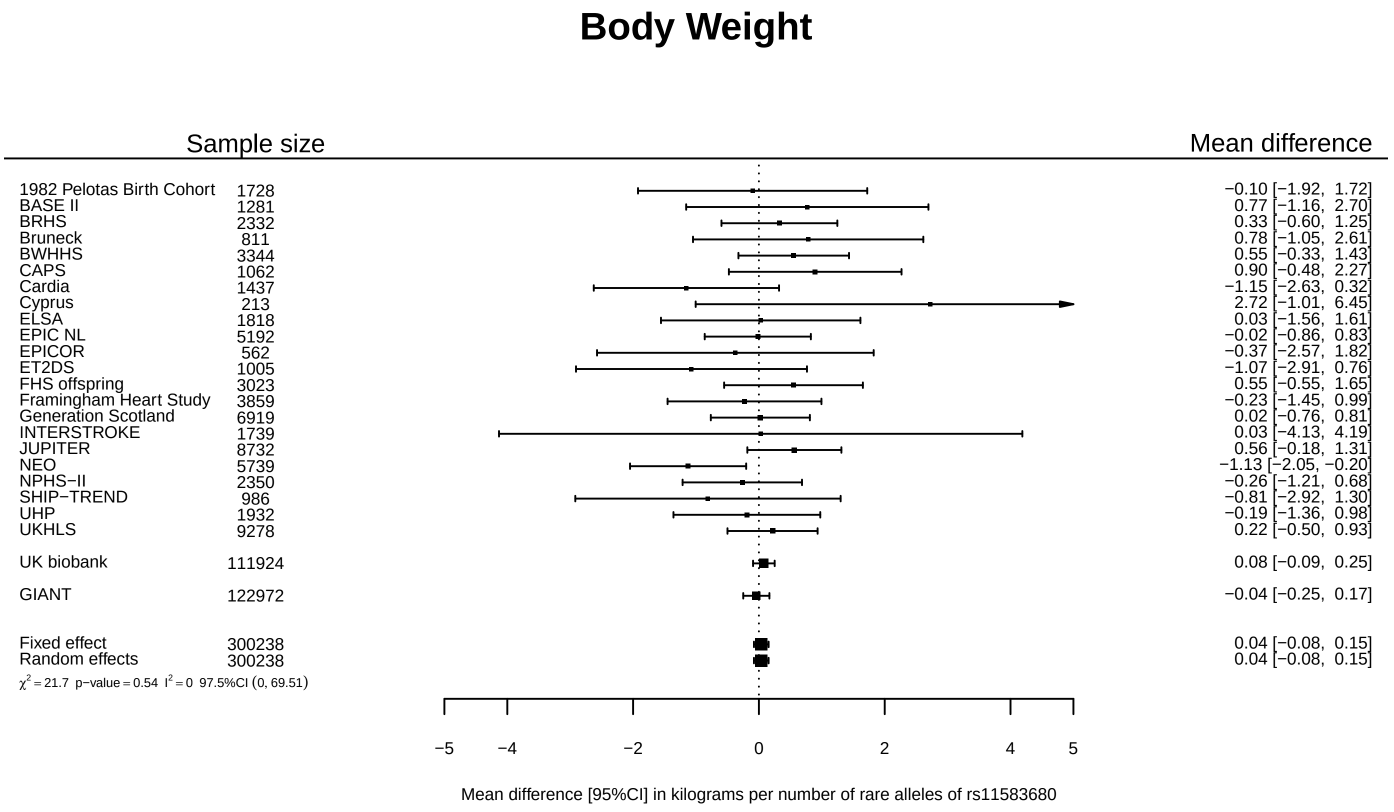

Figure 39: Mean difference of PCSK9 SNP rs11583680 on body weight (kilogram) per number of rare alleles, with 95% confidence interval (CI). Results are pooled using a fixed and random effects model, irrespective of observed between study heterogeneity; measured as a two sided Q–test (χ^2) and the I^2 with one–sided 97.5% CI.

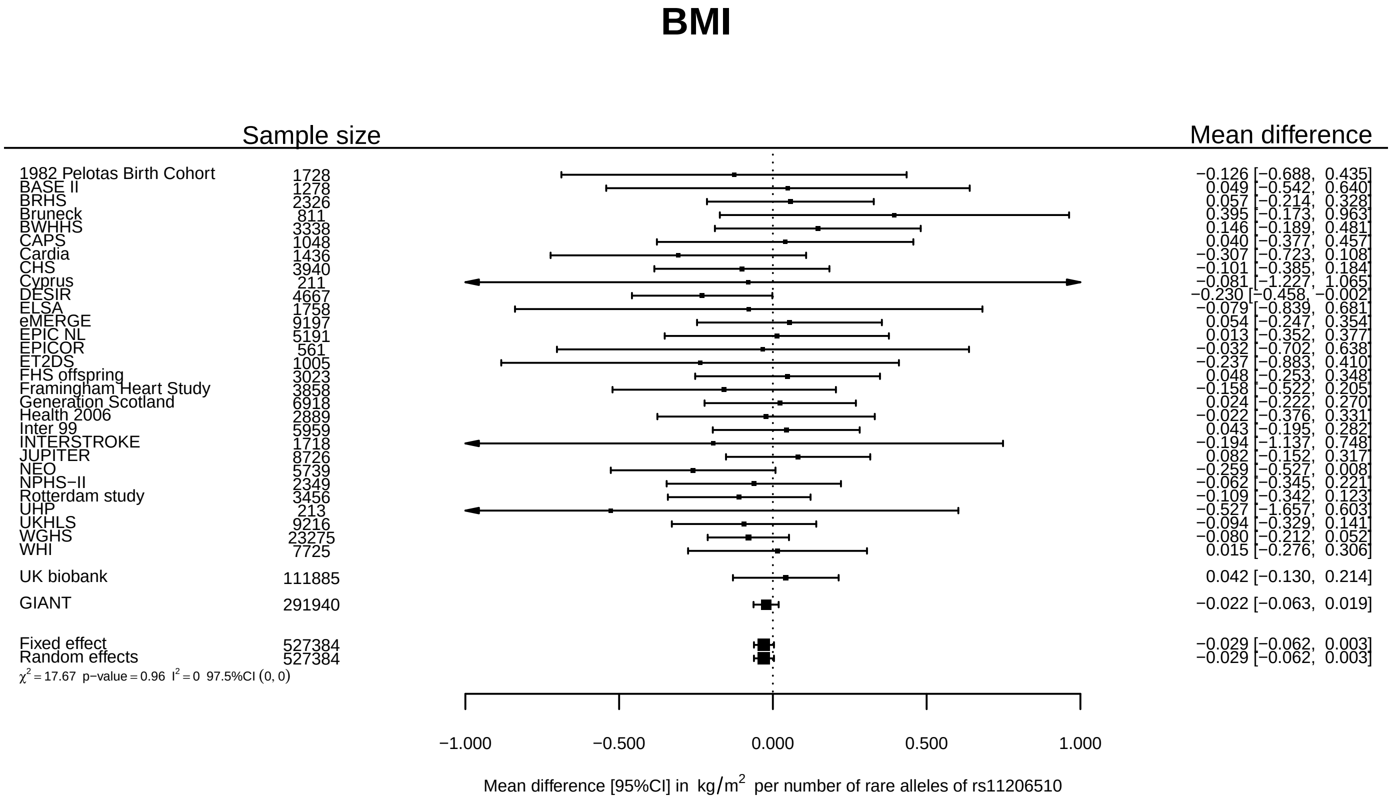

Figure 40: Mean difference of PCSK9 SNP rs11583680 on BMI (kg/m^2) per number of rare alleles, with 95% confidence interval (CI). Results are pooled using a fixed and random effects model, irrespective of observed between study heterogeneity; measured as a two sided Q–test (χ^2) and the I^2 with one–sided 97.5% CI.

Waist to Hip Ratio

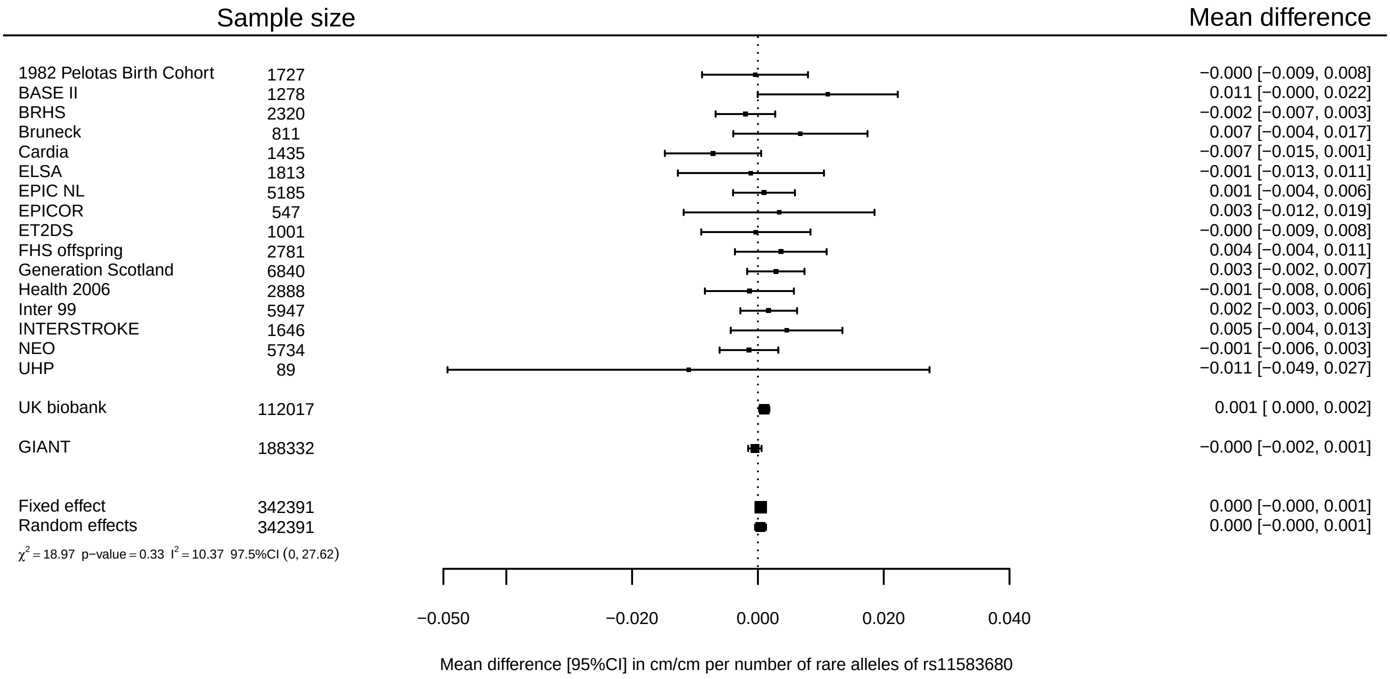

Figure 41: Mean difference of PCSK9 SNP rs11583680 on waist to hip ratio per number of rare alleles, with 95% confidence interval (CI). Results are pooled using a fixed and random effects model, irrespective of observed between study heterogeneity; measured as a two sided Q–test (χ^2) and the I^2 with one–sided 97.5% CI.

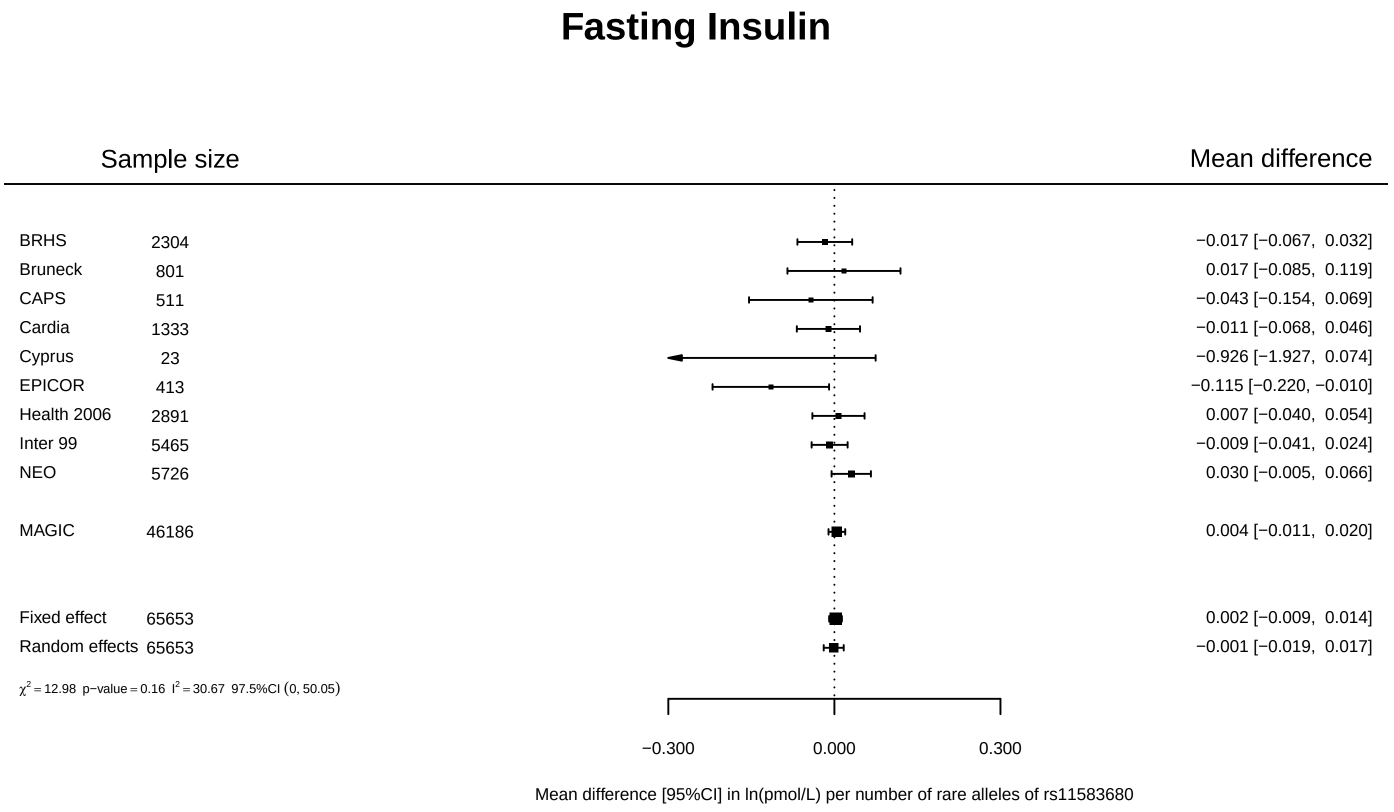

Figure 42: Mean difference of PCSK9 SNP rs11583680 on fasting insulin $ln(pmol/L)$ per number of rare alleles, with 95% confidence interval (CI). Results are pooled using a fixed and random effects model, irrespective of observed between study heterogeneity; measured as a two sided Q–test (χ^2) and the I^2 with one–sided 97.5% CI.

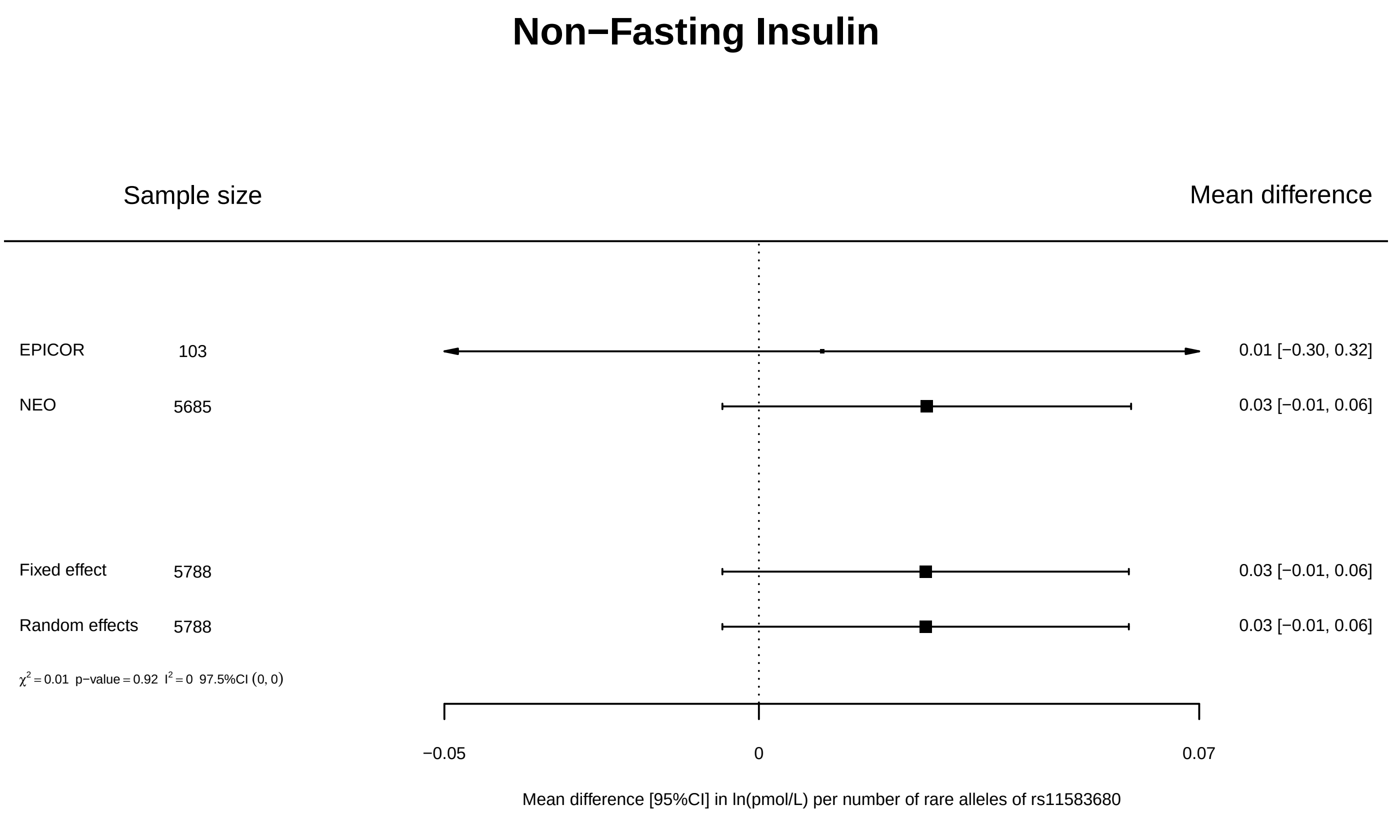

Figure 43: Mean difference of PCSK9 SNP rs11583680 on non–fasting insulin $ln(pmol/L)$ per number of rare alleles, with 95% confidence interval (CI). Results are pooled using a fixed and random effects model, irrespective of observed between study heterogeneity; measured as a two sided Q–test (χ^2) and the I^2 with one–sided 97.5% CI.

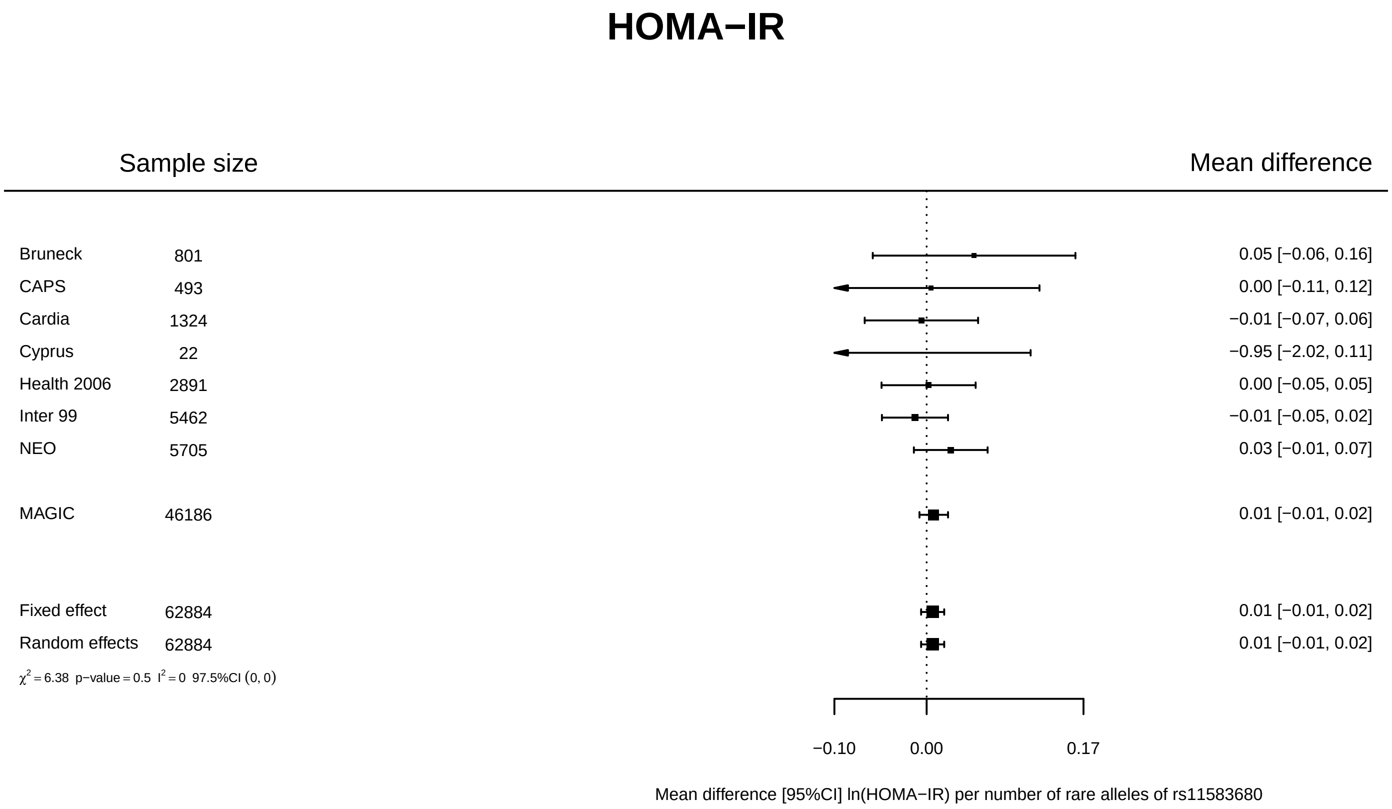

Figure 44: Mean difference of PCSK9 SNP rs11583680 on $ln(HOMA\check{}IR)$ per number of rare alleles, with 95% confidence interval (CI). Results are pooled using a fixed and random effects model, irrespective of observed between study heterogeneity; measured as a two sided Q–test (χ^2) and the I^2 with one–sided 97.5% CI.

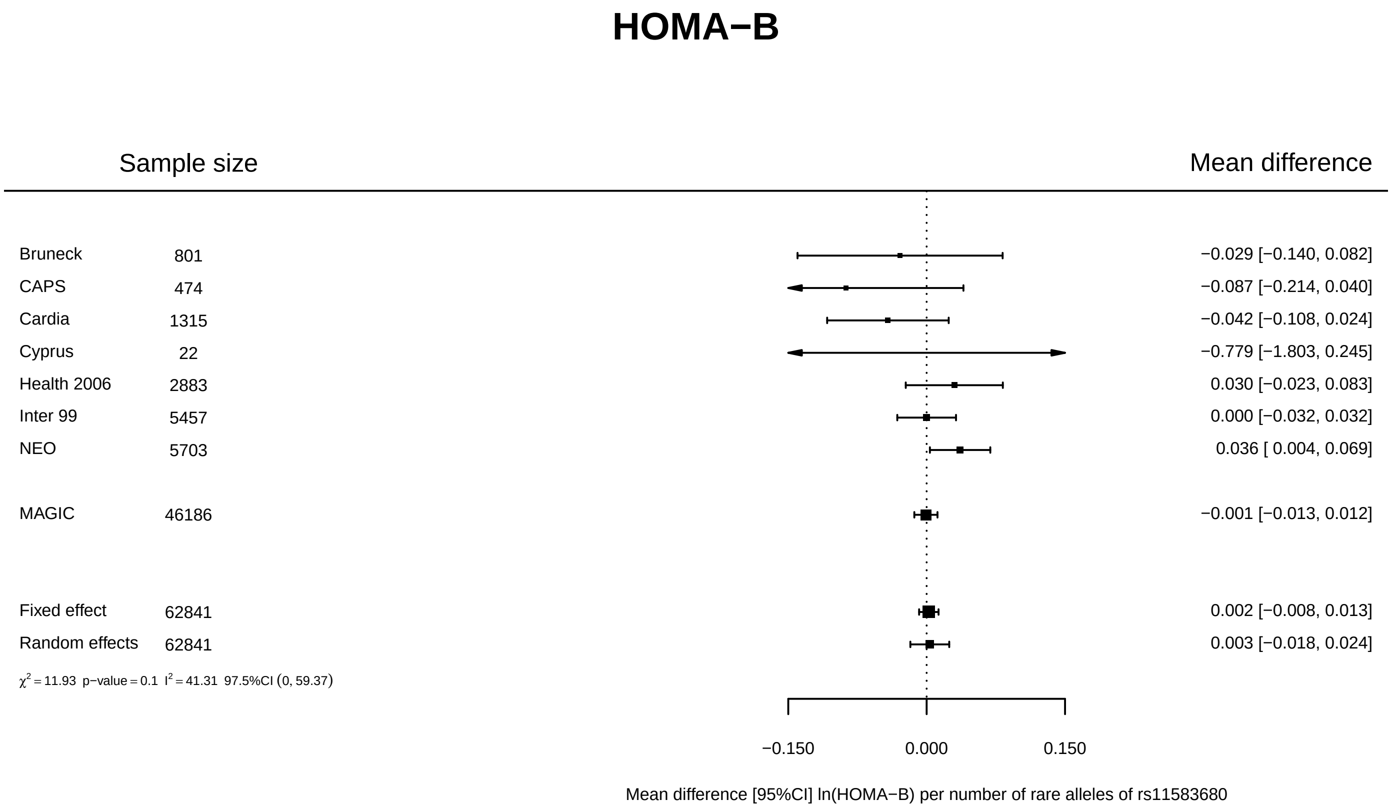

Figure 45: Mean difference of PCSK9 SNP rs11583680 on $ln(HOMA\breve{}\beta)$ per number of rare alleles, with 95% confidence interval (CI). Results are pooled using a fixed and random effects model, irrespective of observed between study heterogeneity; measured as a two sided Q–test (χ^2) and the I^2 with one–sided 97.5% CI.

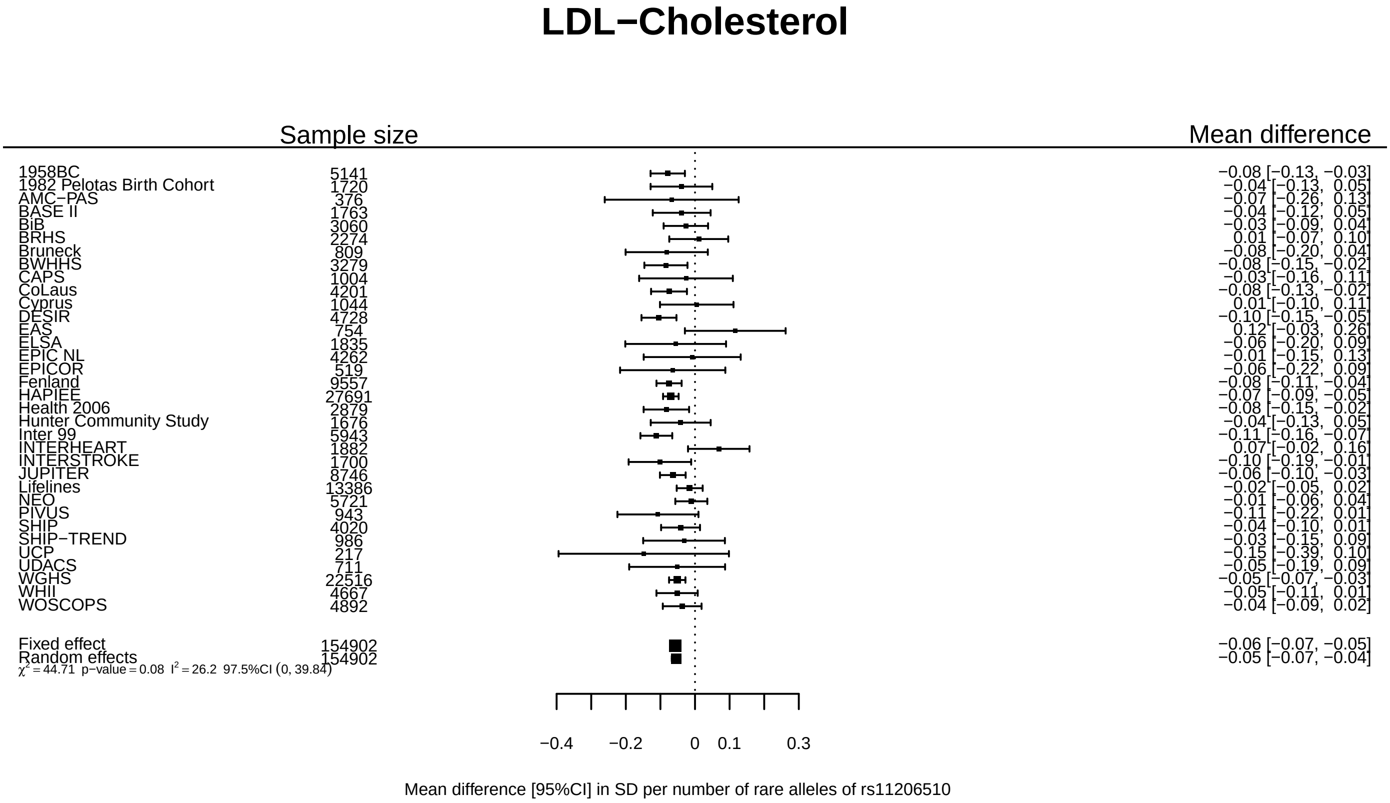

Figure 46: Mean difference of PCSK9 SNP rs11206510 on LDL–C (SD) per number of rare alleles, with 95% confidence interval (CI). Results are pooled using a fixed and random effects model, irrespective of observed between study heterogeneity; measured as a two sided Q–test (χ^2) and the I^2 with one–sided 97.5% CI.

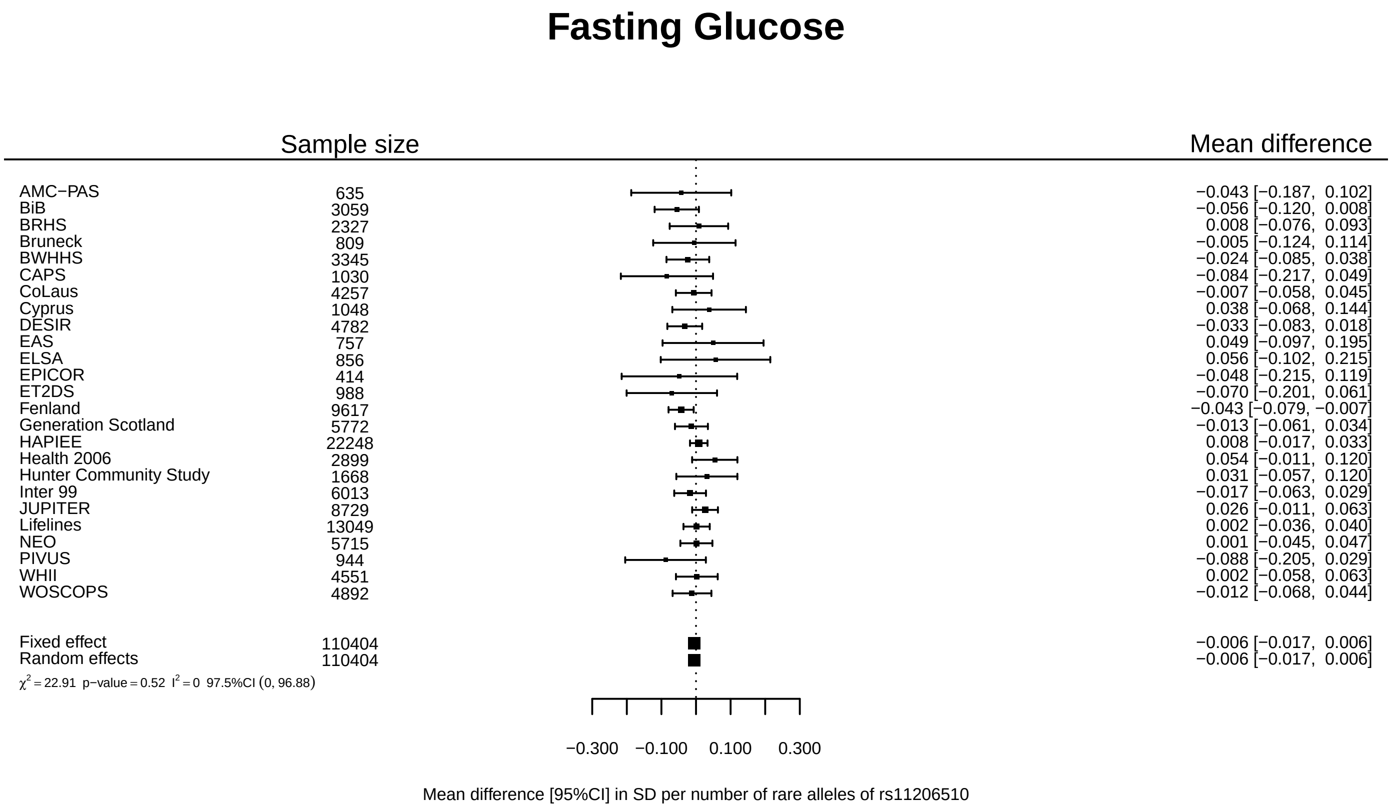

Figure 47: Mean difference of PCSK9 SNP rs11206510 on fasting glucose (SD) per number of rare alleles, with 95% confidence interval (CI). Results are pooled using a fixed and random effects model, irrespective of observed between study heterogeneity; measured as a two sided Q–test (χ^2) and the I^2 with one–sided 97.5% CI.

Non−Fasting Glucose

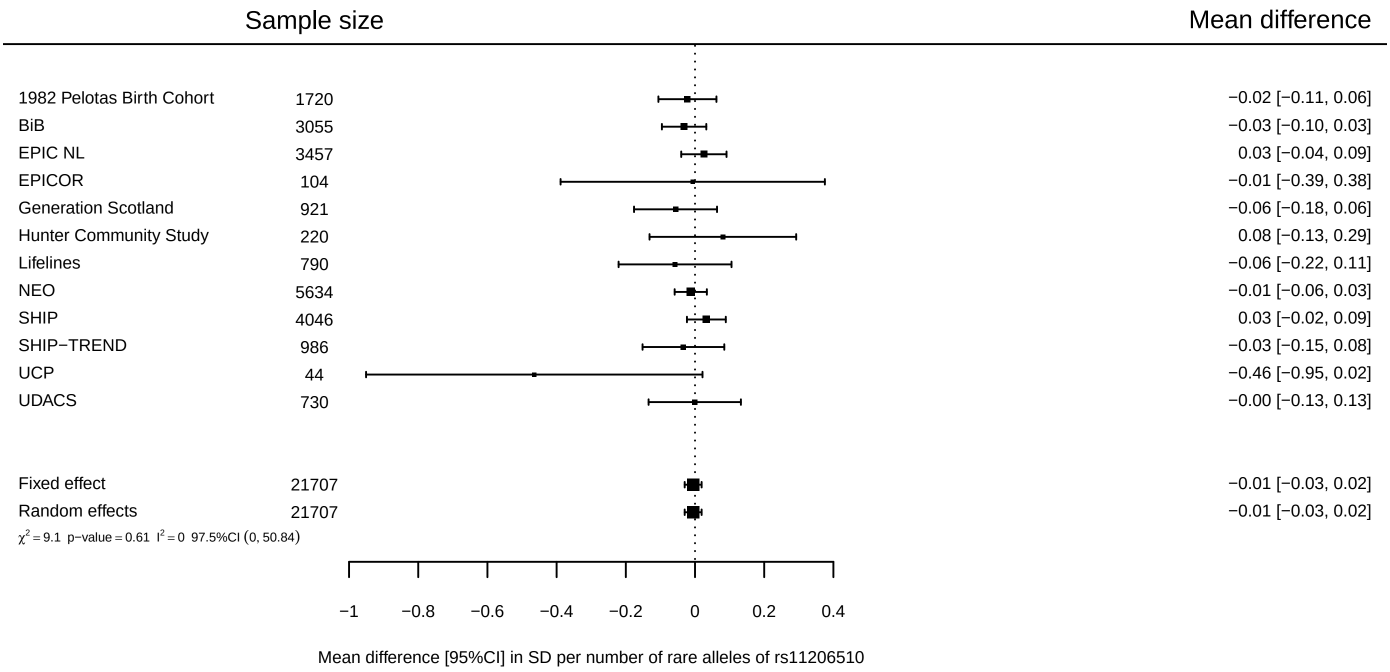

Figure 48: Mean difference of PCSK9 SNP rs11206510 on non–fasting glucose (SD) per number of rare alleles, with 95% confidence interval (CI). Results are pooled using a fixed and random effects model, irrespective of observed between study heterogeneity; measured as a two sided Q–test (χ^2) and the I^2 with one–sided 97.5% CI.

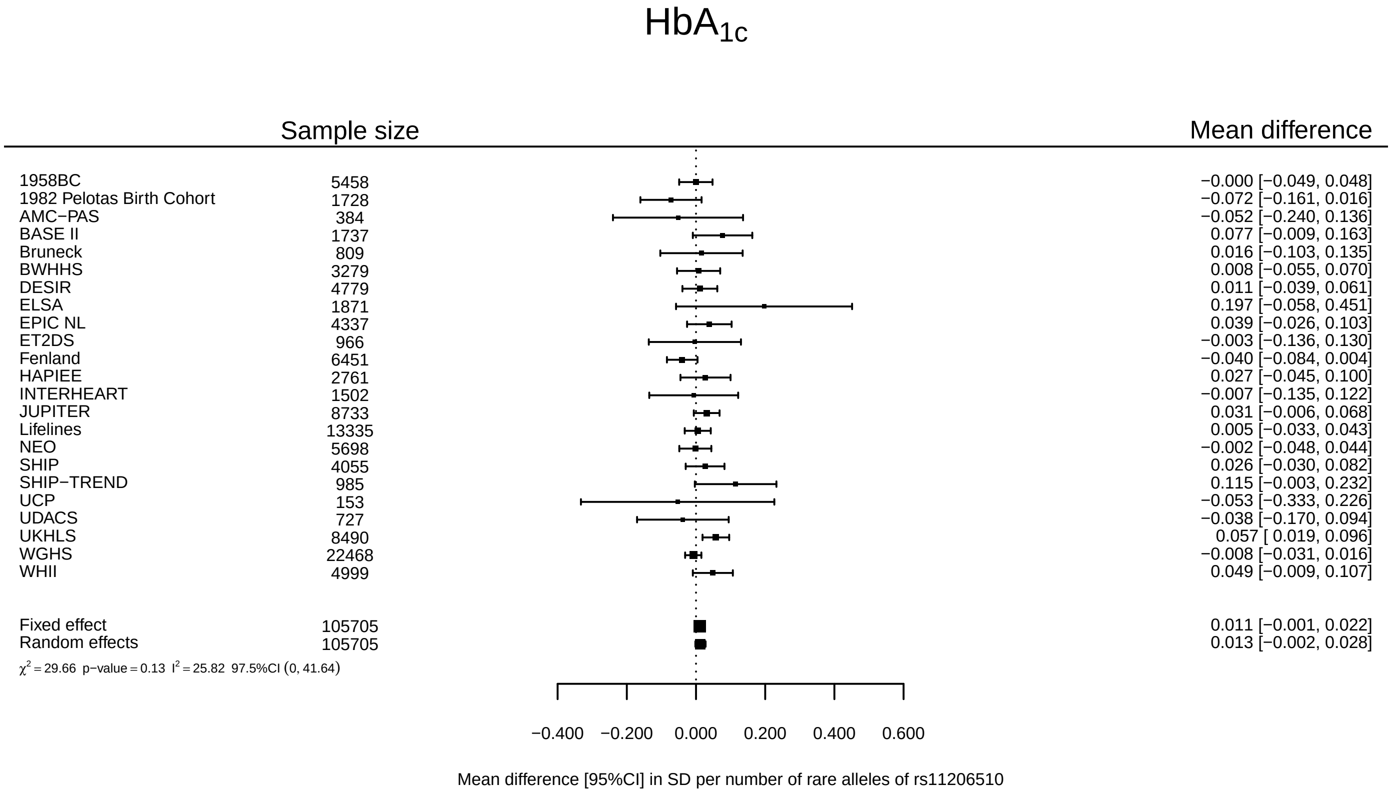

Figure 49: Mean difference of PCSK9 SNP rs11206510 on HbA_{1c} (SD) per number of rare alleles, with 95% confidence interval (CI). Results are pooled using a fixed and random effects model, irrespective of observed between study heterogeneity; measured as a two sided Q–test (χ^2) and the I^2 with one–sided 97.5% CI.

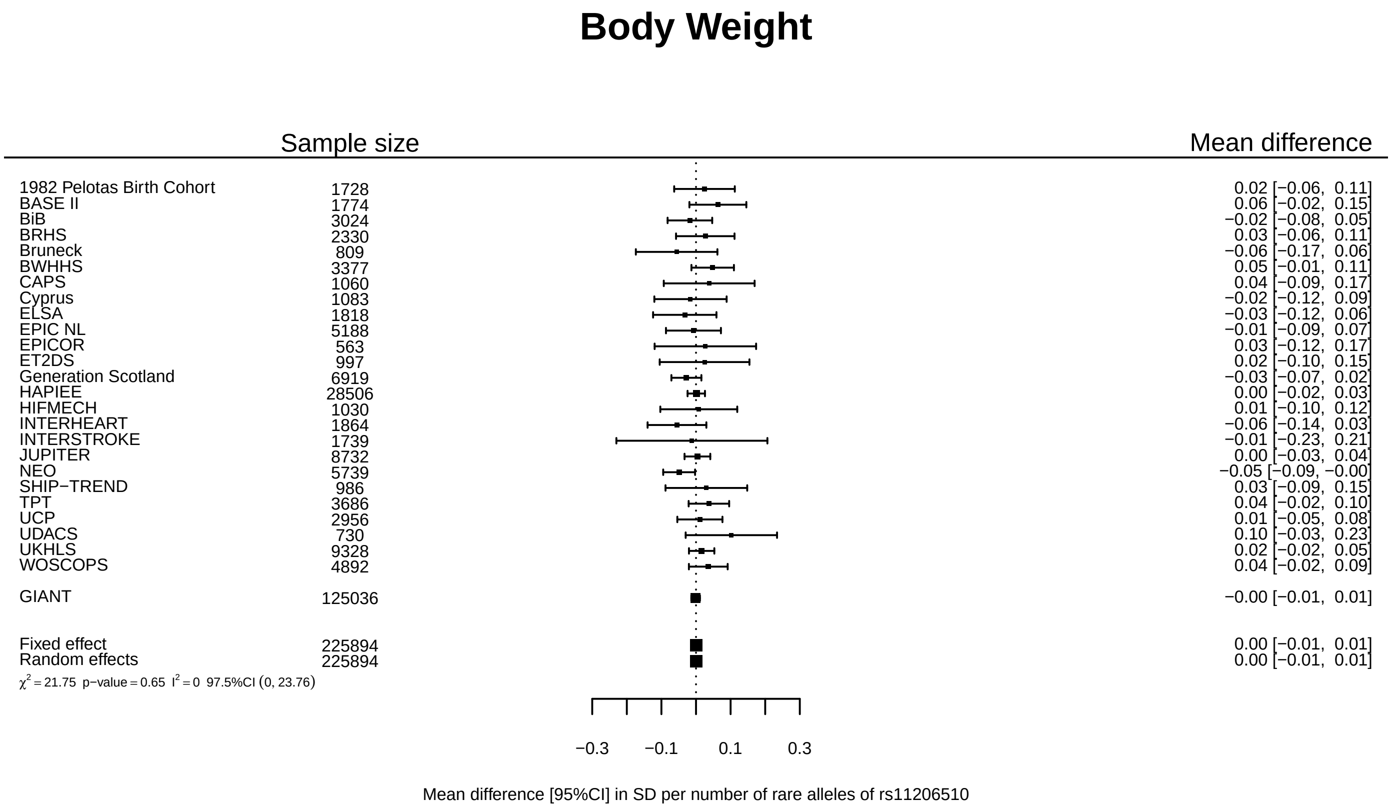

Figure 50: Mean difference of PCSK9 SNP rs11206510 on body weight (SD) per number of rare alleles, with 95% confidence interval (CI). Results are pooled using a fixed and random effects model, irrespective of observed between study heterogeneity; measured as a two sided Q–test (χ^2) and the I^2 with one–sided 97.5% CI.

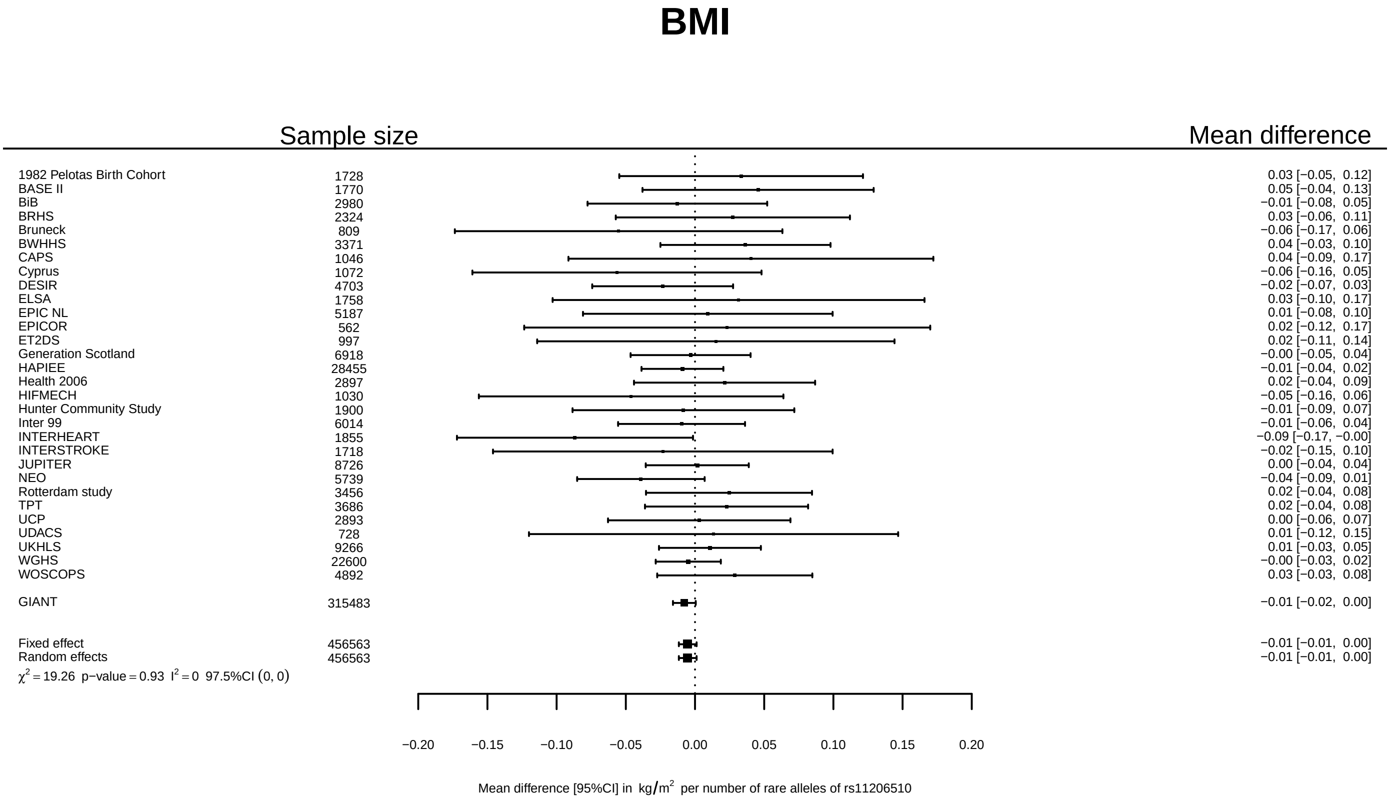

Figure 51: Mean difference of PCSK9 SNP rs11206510 on BMI (SD) per number of rare alleles, with 95% confidence interval (CI). Results are pooled using a fixed and random effects model, irrespective of observed between study heterogeneity; measured as a two sided Q–test (χ^2) and the I^2 with one–sided 97.5% CI.

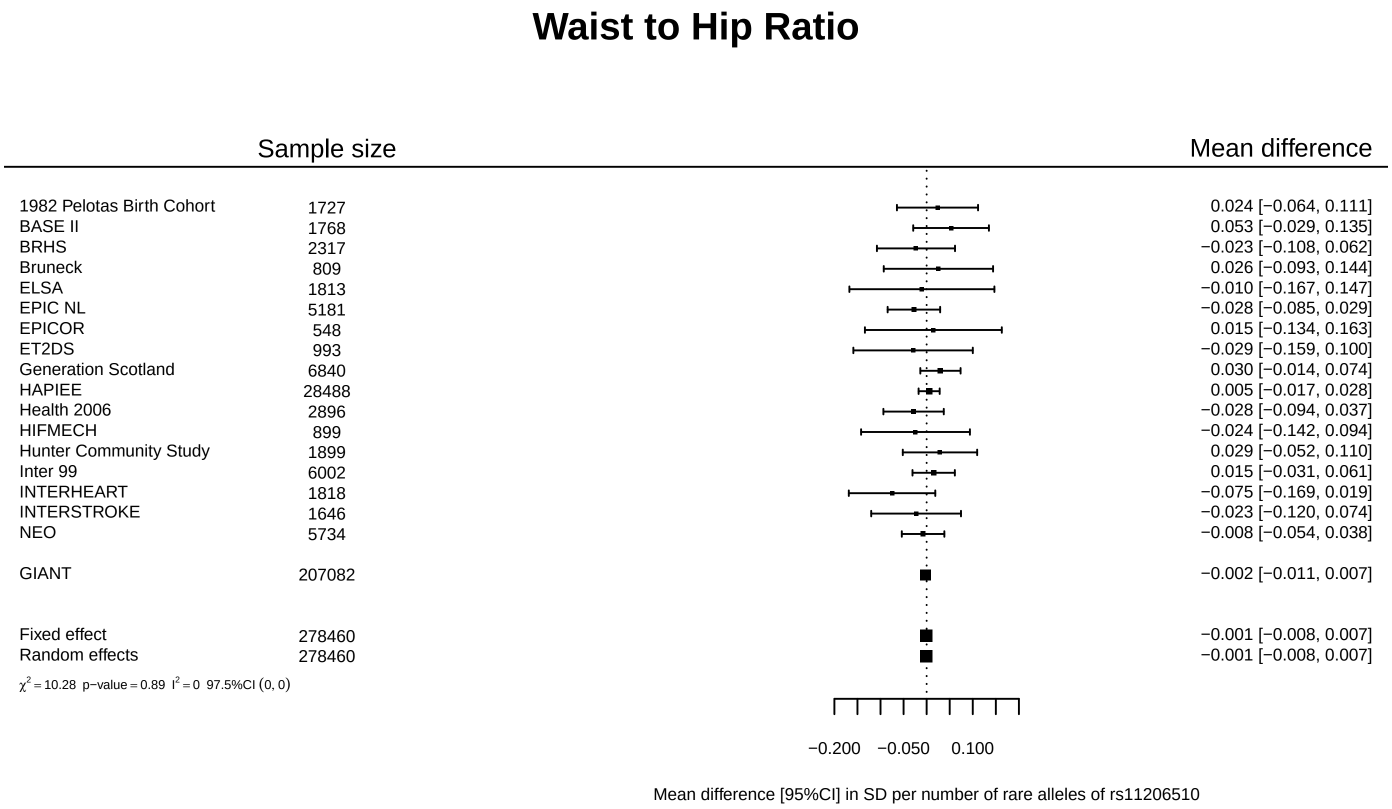

Figure 52: Mean difference of PCSK9 SNP rs11206510 on waist to hip ratio (SD) per number of rare alleles, with 95% confidence interval (CI). Results are pooled using a fixed and random effects model, irrespective of observed between study heterogeneity; measured as a two sided Q–test (χ^2) and the I^2 with one–sided 97.5% CI.

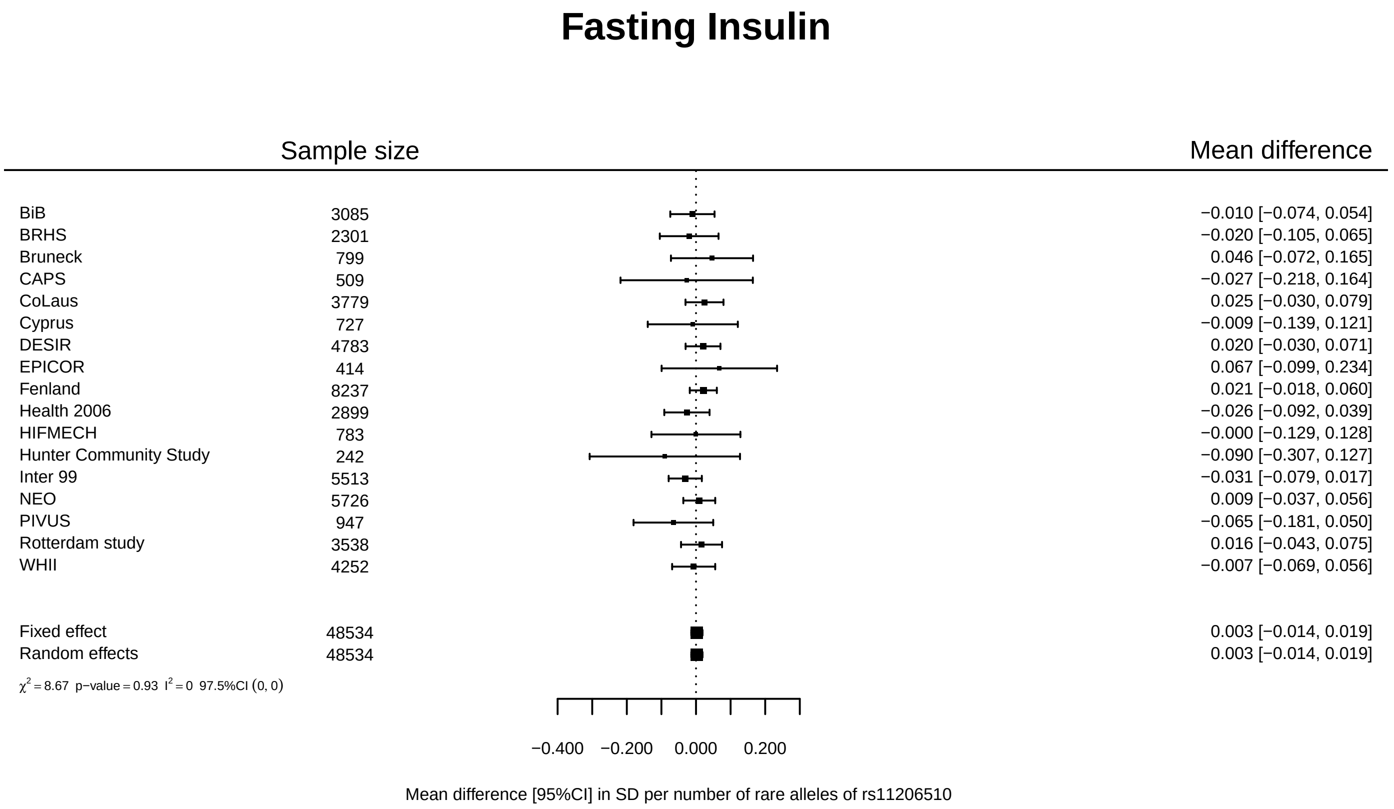

Figure 53: Mean difference of PCSK9 SNP rs11206510 on fasting insulin (SD) per number of rare alleles, with 95% confidence interval (CI). Results are pooled using a fixed and random effects model, irrespective of observed between study heterogeneity; measured as a two sided Q–test (χ^2) and the I^2 with one–sided 97.5% CI. Note the outcome was standardized based on the SD of the natural logarithm.

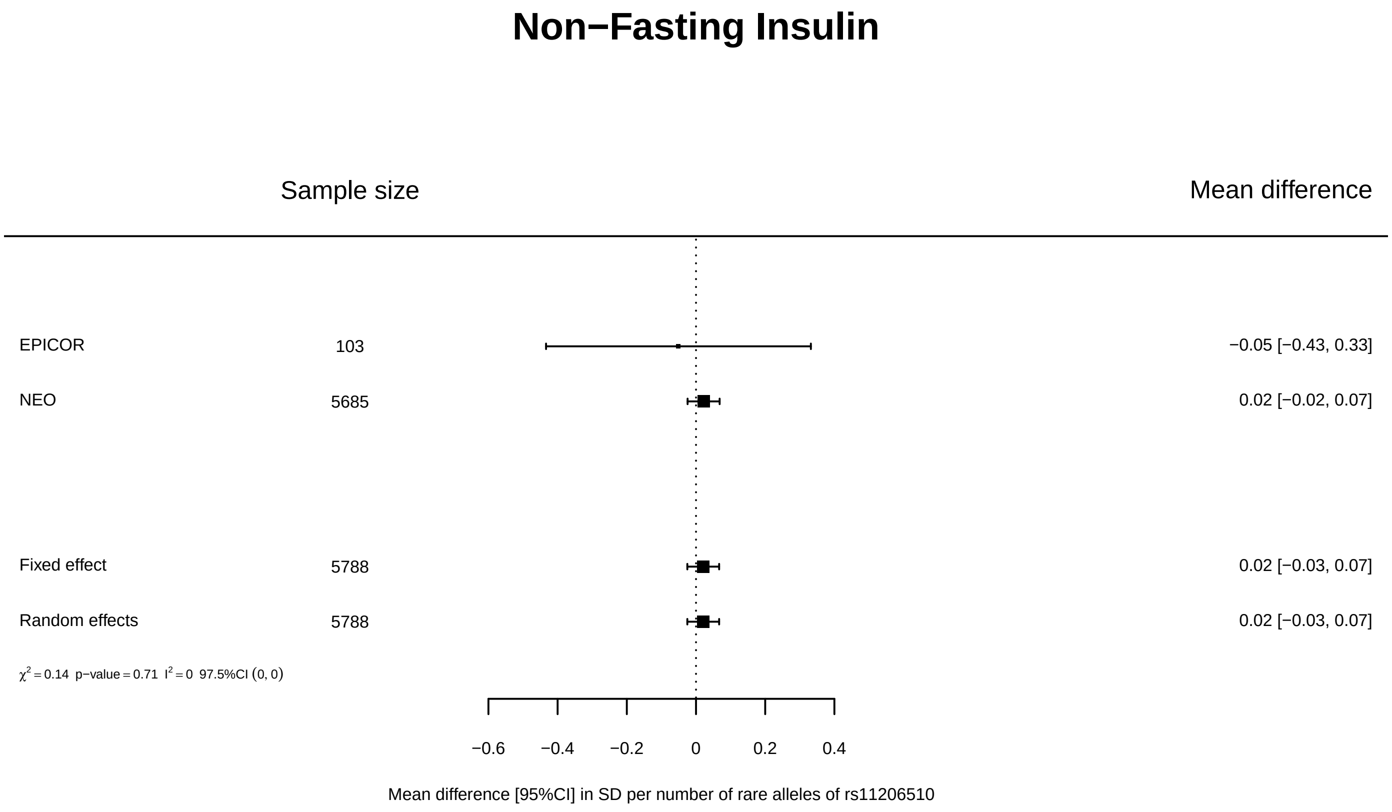

Figure 54: Mean difference of PCSK9 SNP rs11206510 on non–fasting insulin (SD) per number of rare alleles, with 95% confidence interval (CI). Results are pooled using a fixed and random effects model, irrespective of observed between study heterogeneity; measured as a two sided Q–test (χ^2) and the I^2 with one–sided 97.5% CI. Note the outcome was standardized based on the SD of the natural logarithm

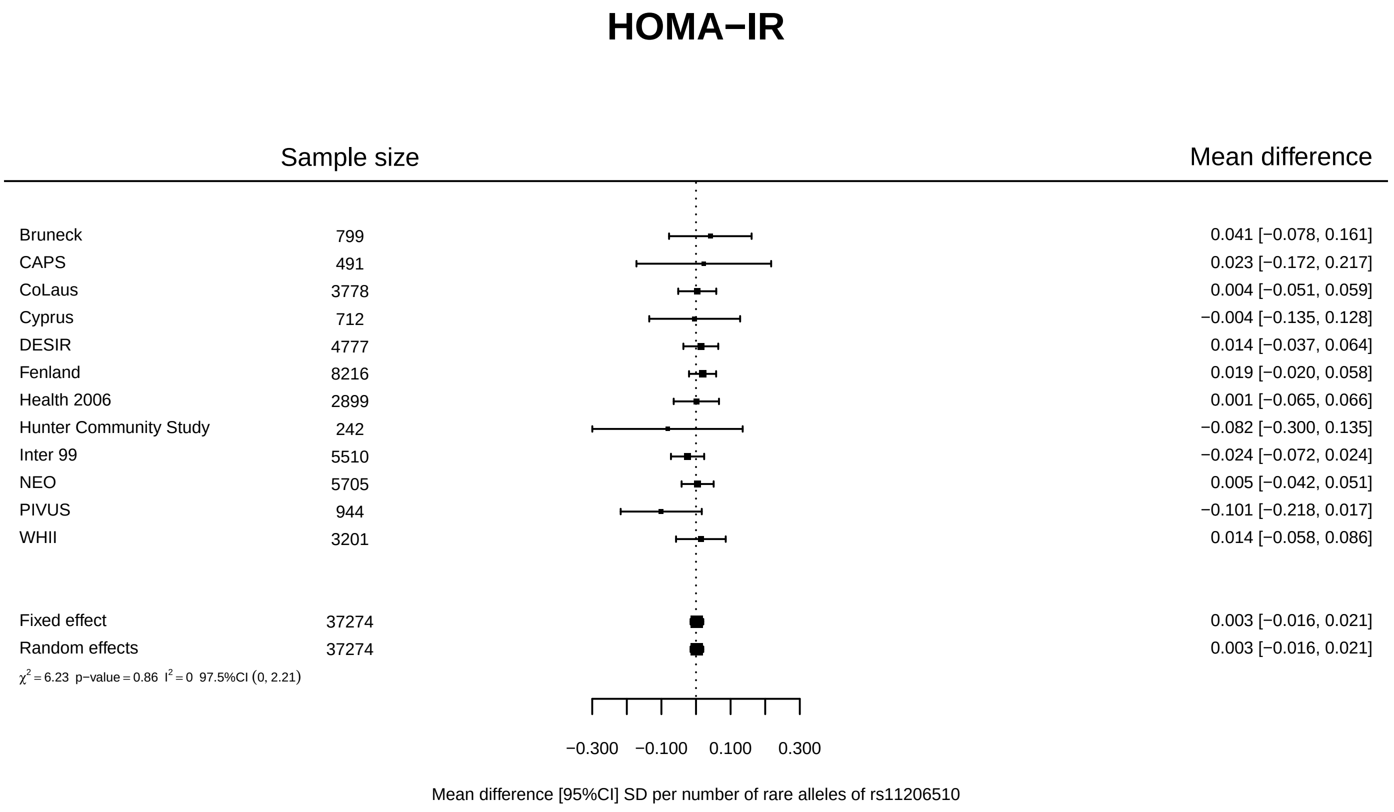

Figure 55: Mean difference of PCSK9 SNP rs11206510 on $HOMA\breve{\ }IR$ (SD) per number of rare alleles, with 95% confidence interval (CI). Results are pooled using a fixed and random effects model, irrespective of observed between study heterogeneity; measured as a two sided Q–test (χ^2) and the I^2 with one–sided 97.5% CI. Note the outcome was standardized based on the SD of the natural logarithm

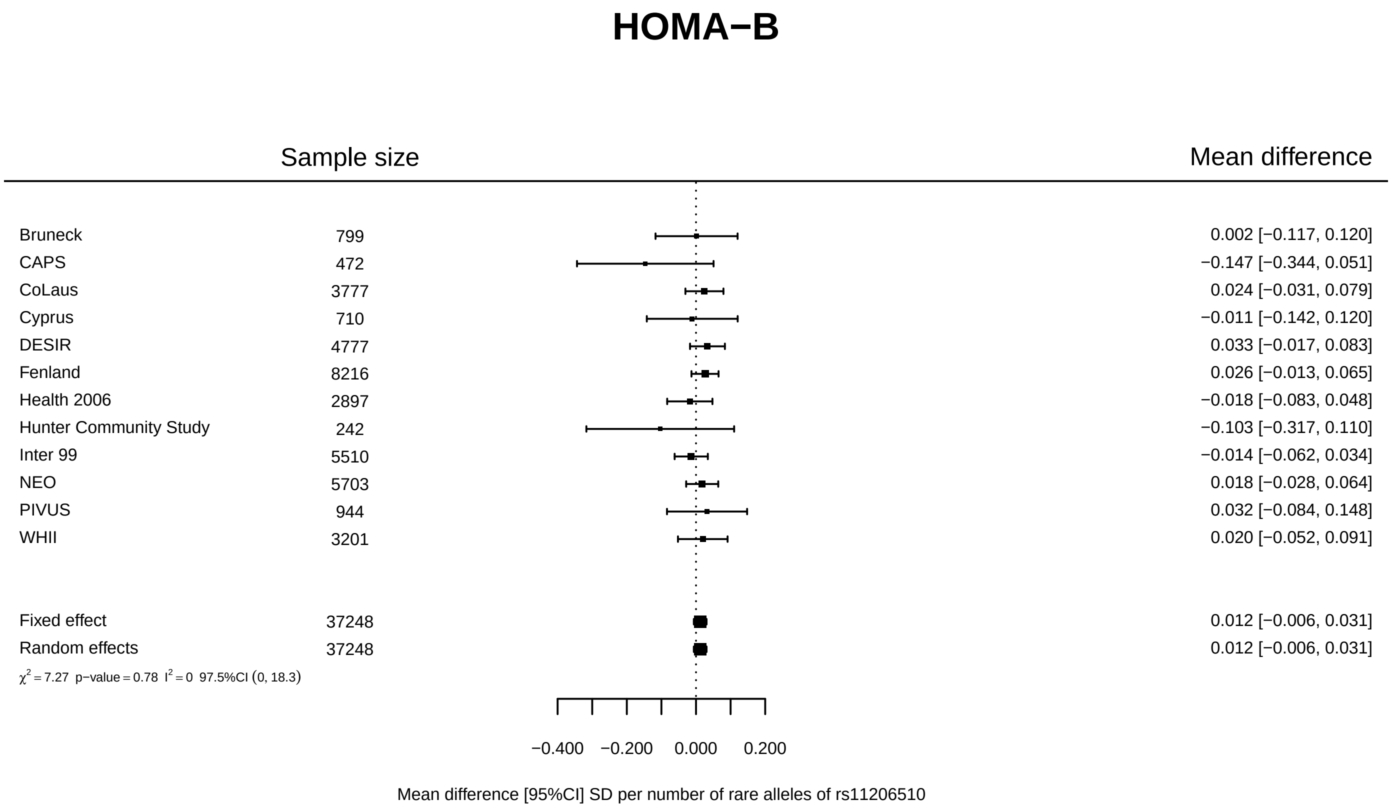

Figure 56: Mean difference of PCSK9 SNP rs11206510 on $HOMA\breve{}\beta$ (SD) per number of rare alleles, with 95% confidence interval (CI). Results are pooled using a fixed and random effects model, irrespective of observed between study heterogeneity; measured as a two sided Q–test (χ^2) and the I^2 with one–sided 97.5% CI. Note the outcome was standardized based on the SD of the natural logarithm

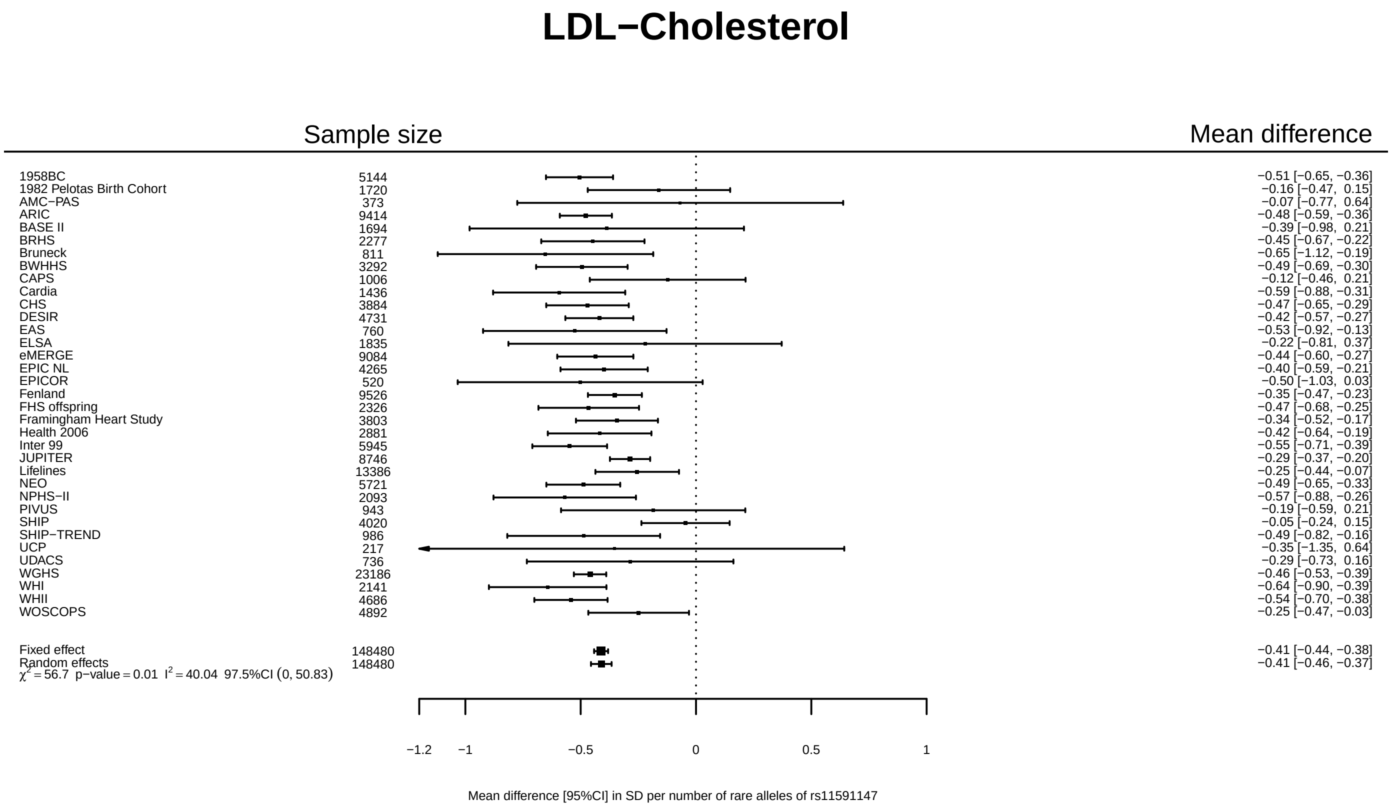

Figure 57: Mean difference of PCSK9 SNP rs11591147 on LDL–C (SD) per number of rare alleles, with 95% confidence interval (CI). Results are pooled using a fixed and random effects model, irrespective of observed between study heterogeneity; measured as a two sided Q–test (χ^2) and the I^2 with one–sided 97.5% CI.

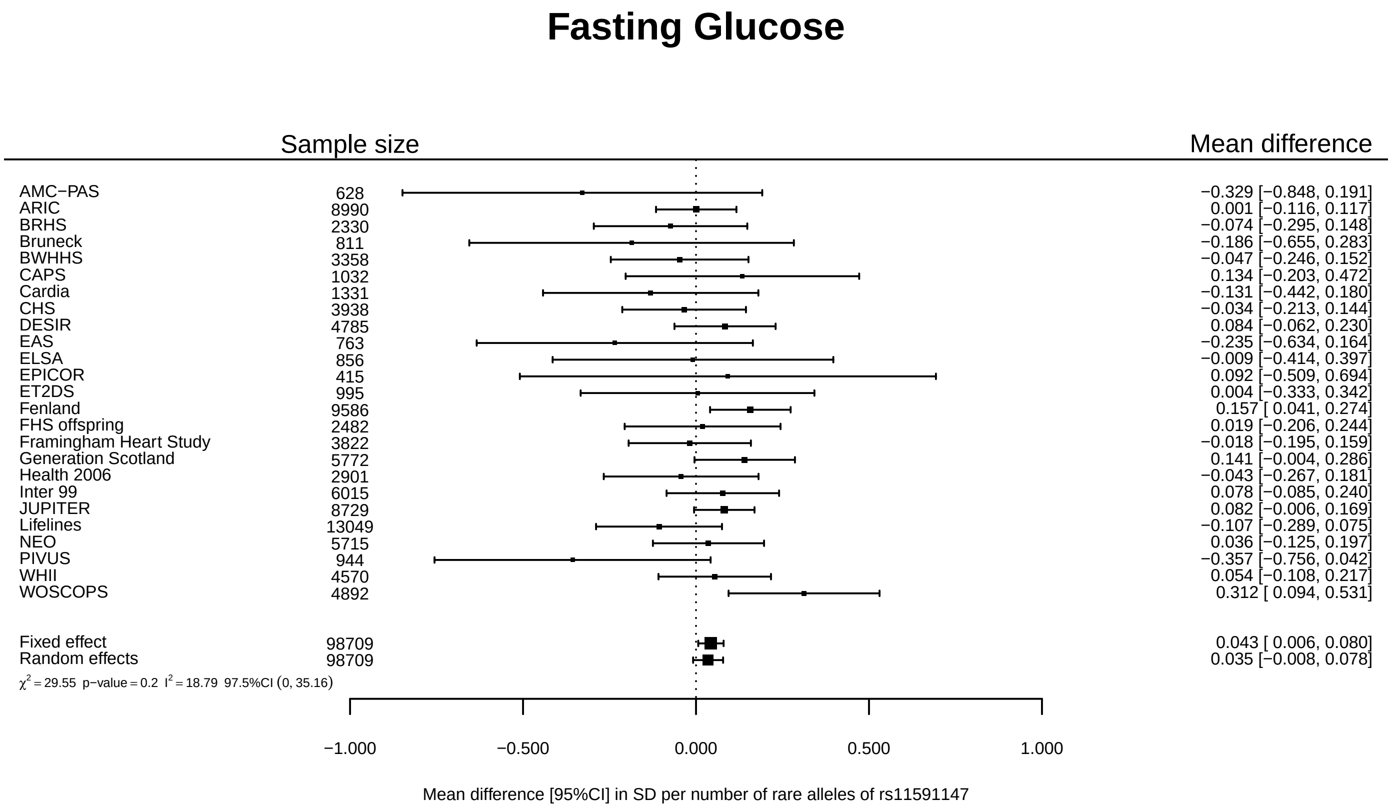

Figure 58: Mean difference of PCSK9 SNP rs11591147 on fasting glucose (SD) per number of rare alleles, with 95% confidence interval (CI). Results are pooled using a fixed and random effects model, irrespective of observed between study heterogeneity; measured as a two sided Q–test (χ^2) and the I^2 with one–sided 97.5% CI.

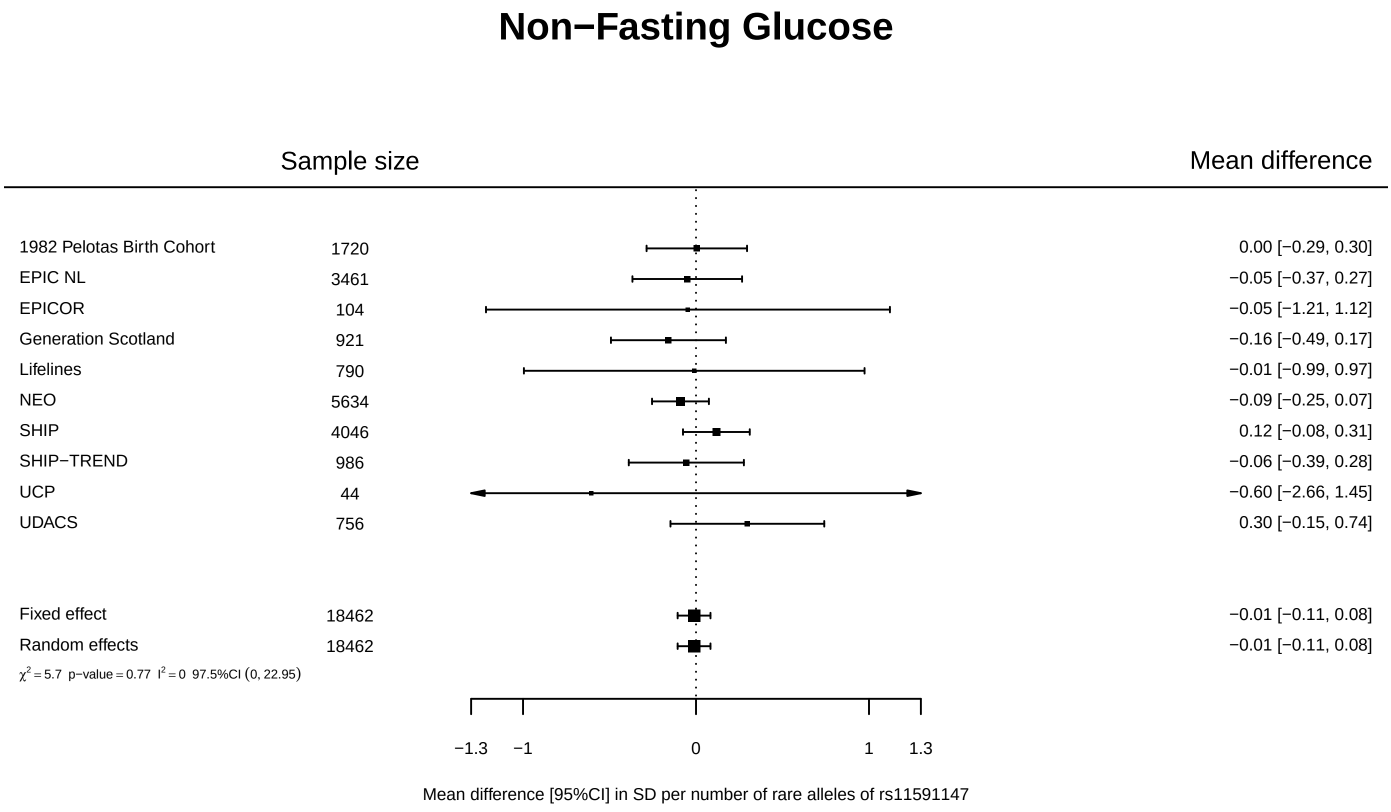

Figure 59: Mean difference of PCSK9 SNP rs11591147 on non–fasting glucose (SD) per number of rare alleles, with 95% confidence interval (CI). Results are pooled using a fixed and random effects model, irrespective of observed between study heterogeneity; measured as a two sided Q–test (χ^2) and the I^2 with one–sided 97.5% CI.

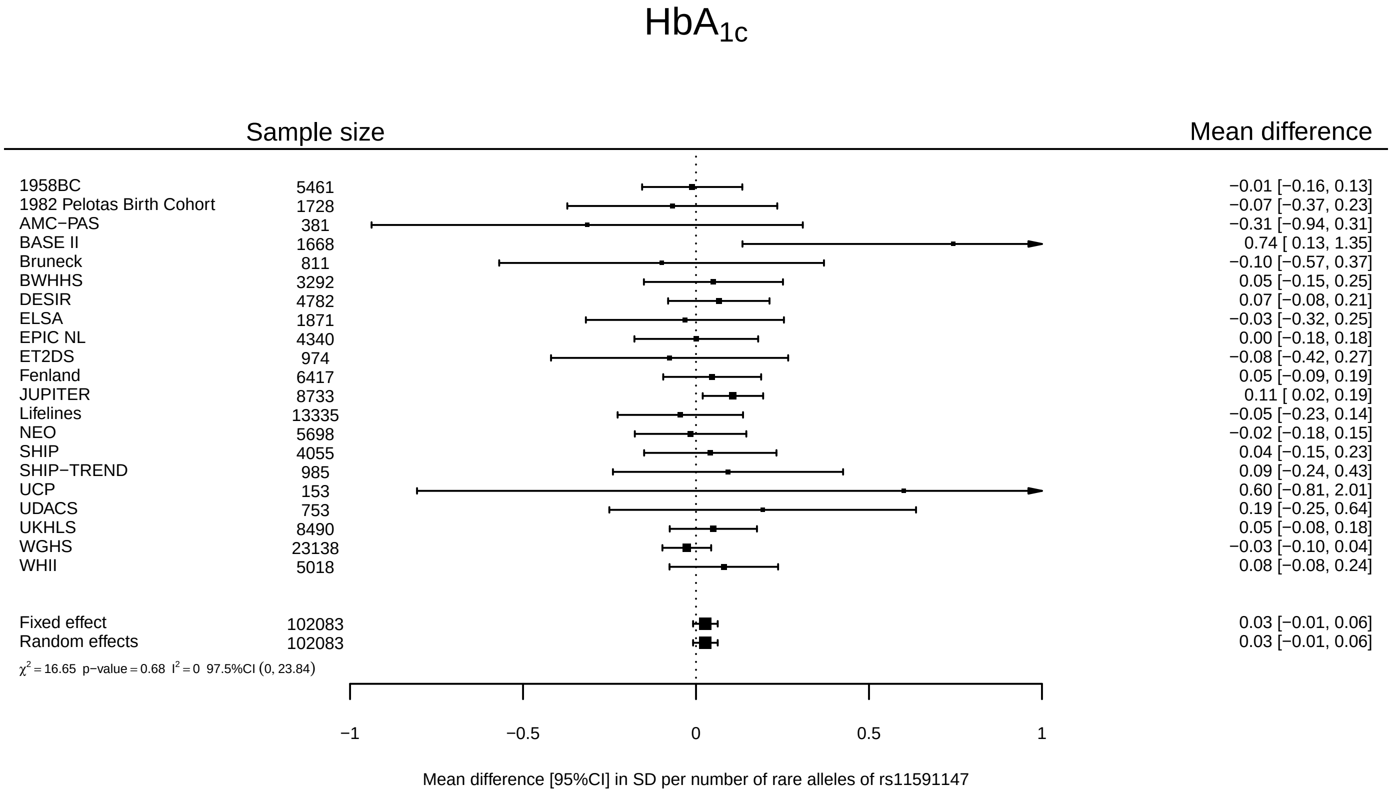

Figure 60: Mean difference of PCSK9 SNP rs11591147 on HbA_{1c} (SD) per number of rare alleles, with 95% confidence interval (CI). Results are pooled using a fixed and random effects model, irrespective of observed between study heterogeneity; measured as a two sided Q–test (χ^2) and the I^2 with one–sided 97.5% CI.

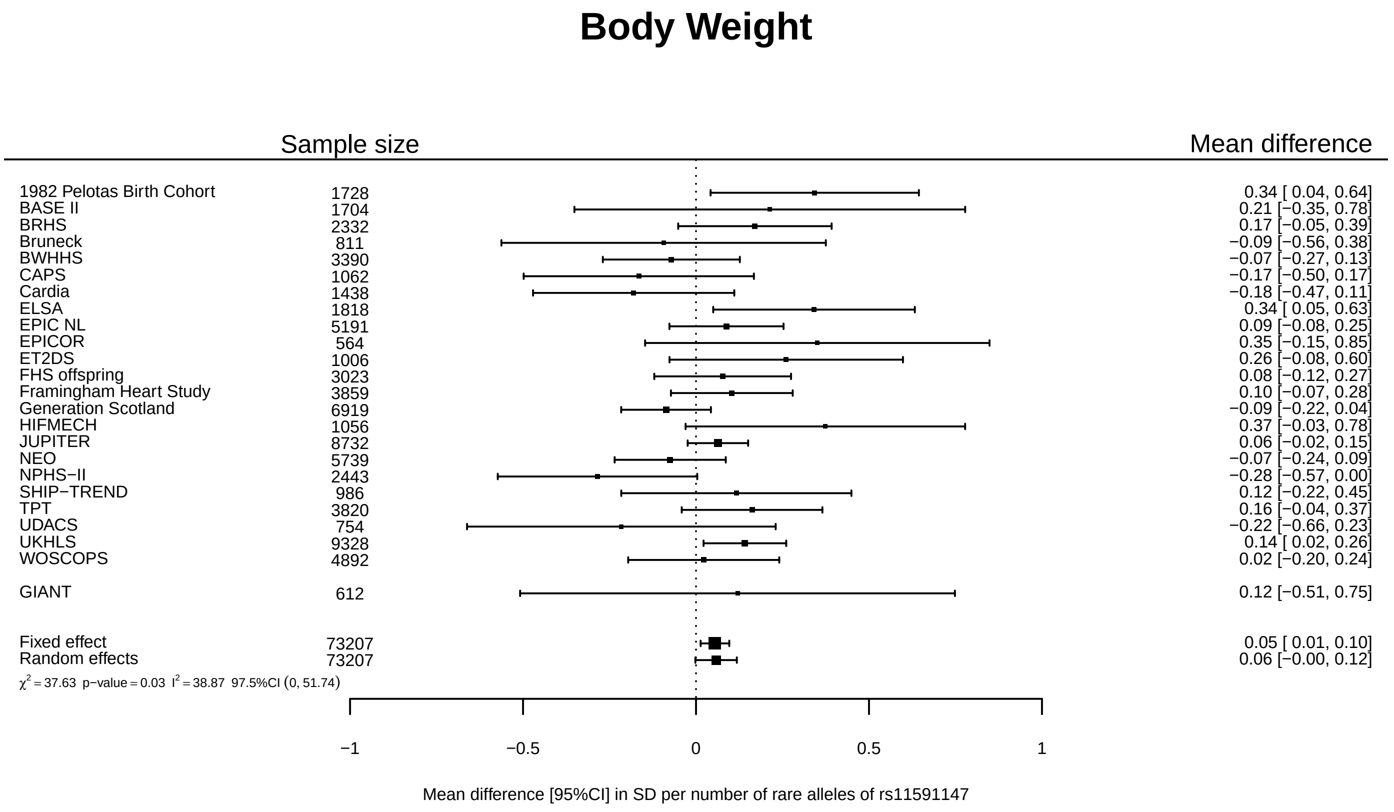

Figure 61: Mean difference of PCSK9 SNP rs11591147 on body weight (SD) per number of rare alleles, with 95% confidence interval (CI). Results are pooled using a fixed and random effects model, irrespective of observed between study heterogeneity; measured as a two sided Q–test (χ^2) and the I^2 with one–sided 97.5% CI.

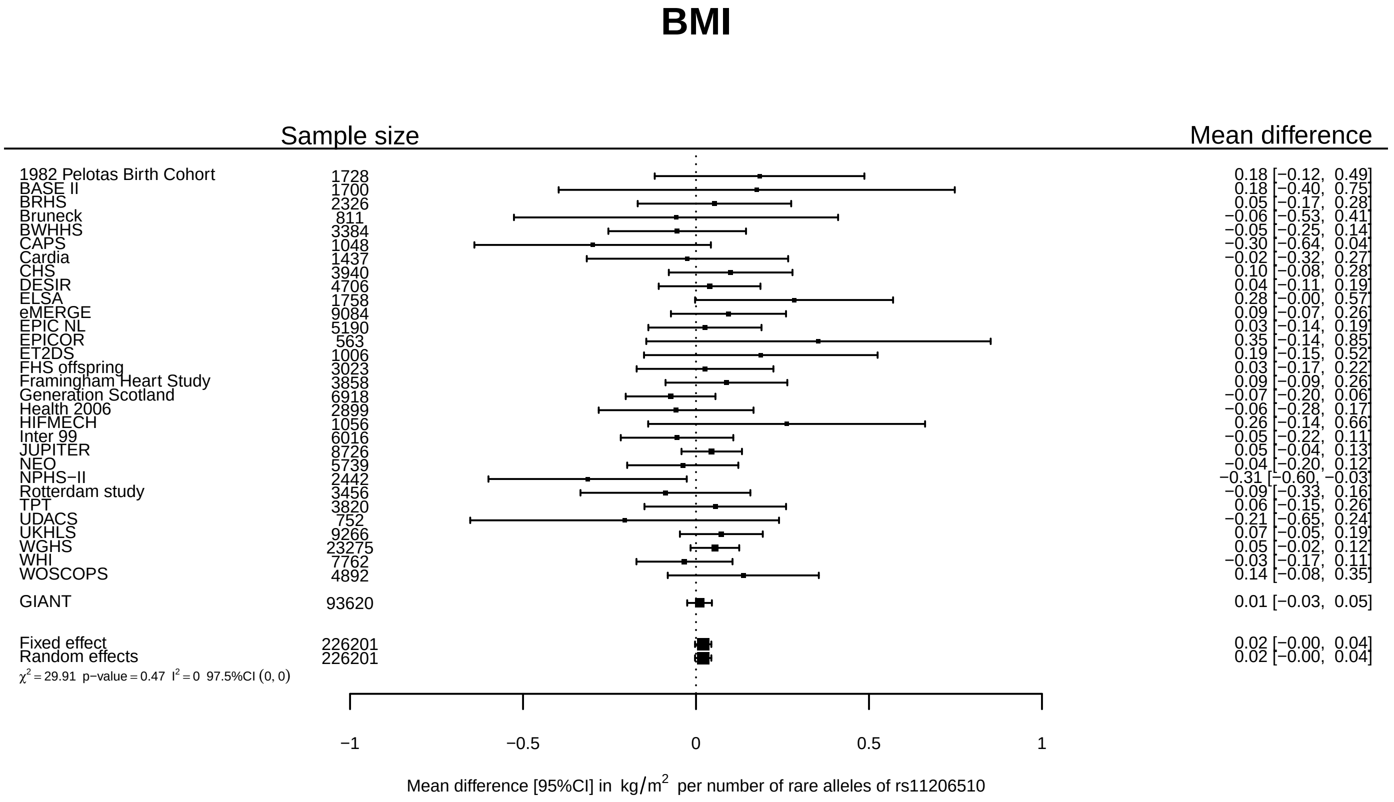

Figure 62: Mean difference of PCSK9 SNP rs11591147 on BMI (SD) per number of rare alleles, with 95% confidence interval (CI). Results are pooled using a fixed and random effects model, irrespective of observed between study heterogeneity; measured as a two sided Q–test (χ^2) and the I^2 with one–sided 97.5% CI.

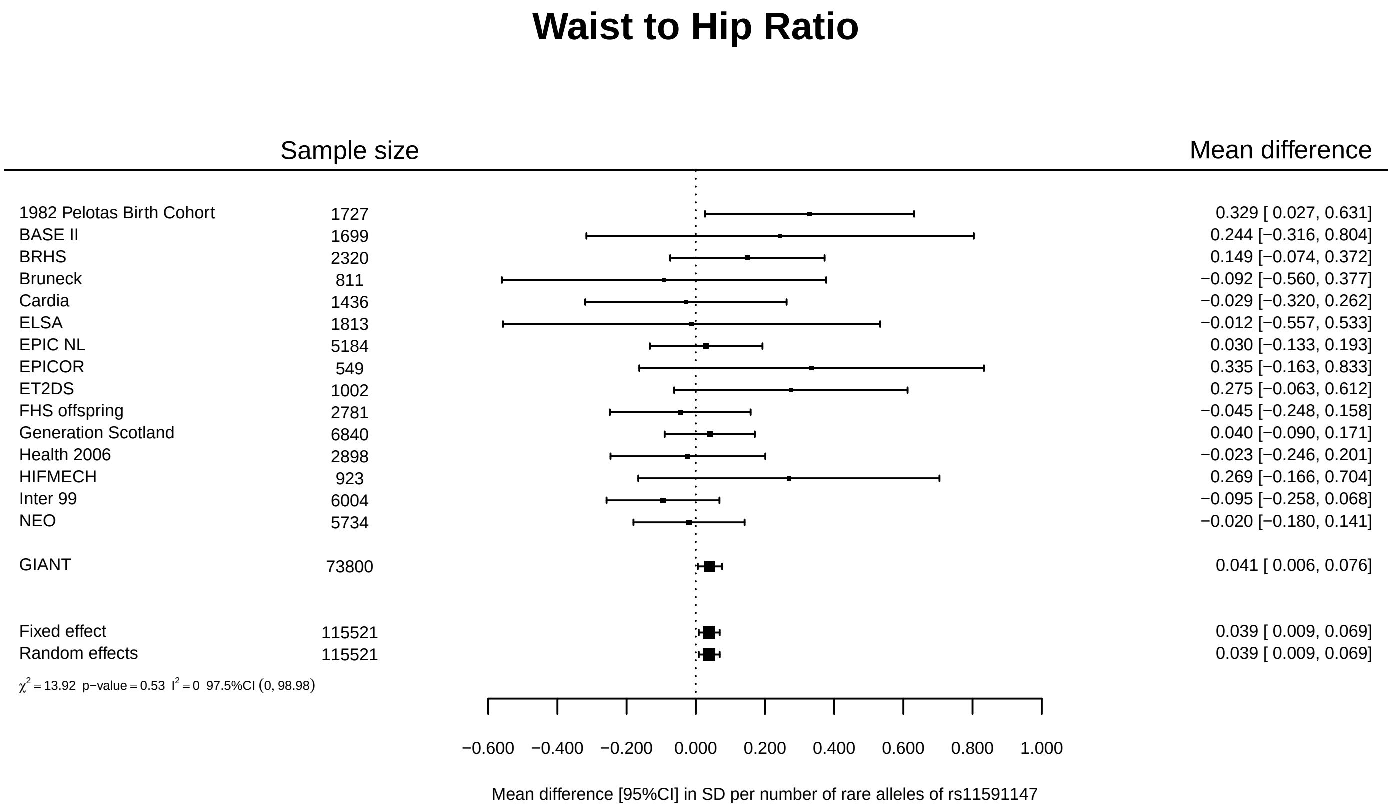

Figure 63: Mean difference of PCSK9 SNP rs11591147 on waist to hip ratio (SD) per number of rare alleles, with 95% confidence interval (CI). Results are pooled using a fixed and random effects model, irrespective of observed between study heterogeneity; measured as a two sided Q–test (χ^2) and the I^2 with one–sided 97.5% CI.

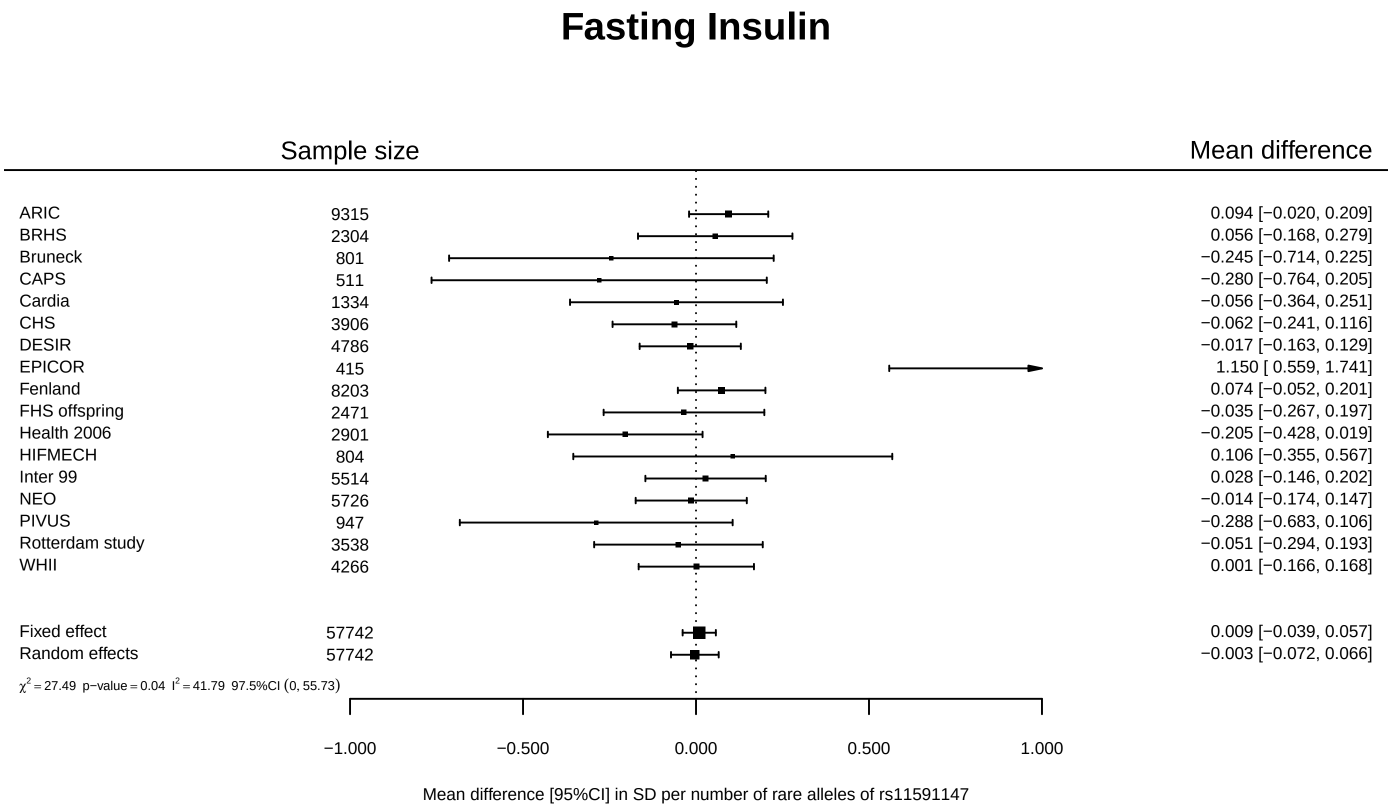

Figure 64: Mean difference of PCSK9 SNP rs11591147 on fasting insulin (SD) per number of rare alleles, with 95% confidence interval (CI). Results are pooled using a fixed and random effects model, irrespective of observed between study heterogeneity; measured as a two sided Q–test (χ^2) and the I^2 with one–sided 97.5% CI. Note the outcome was standardized based on the SD of the natural logarithm.

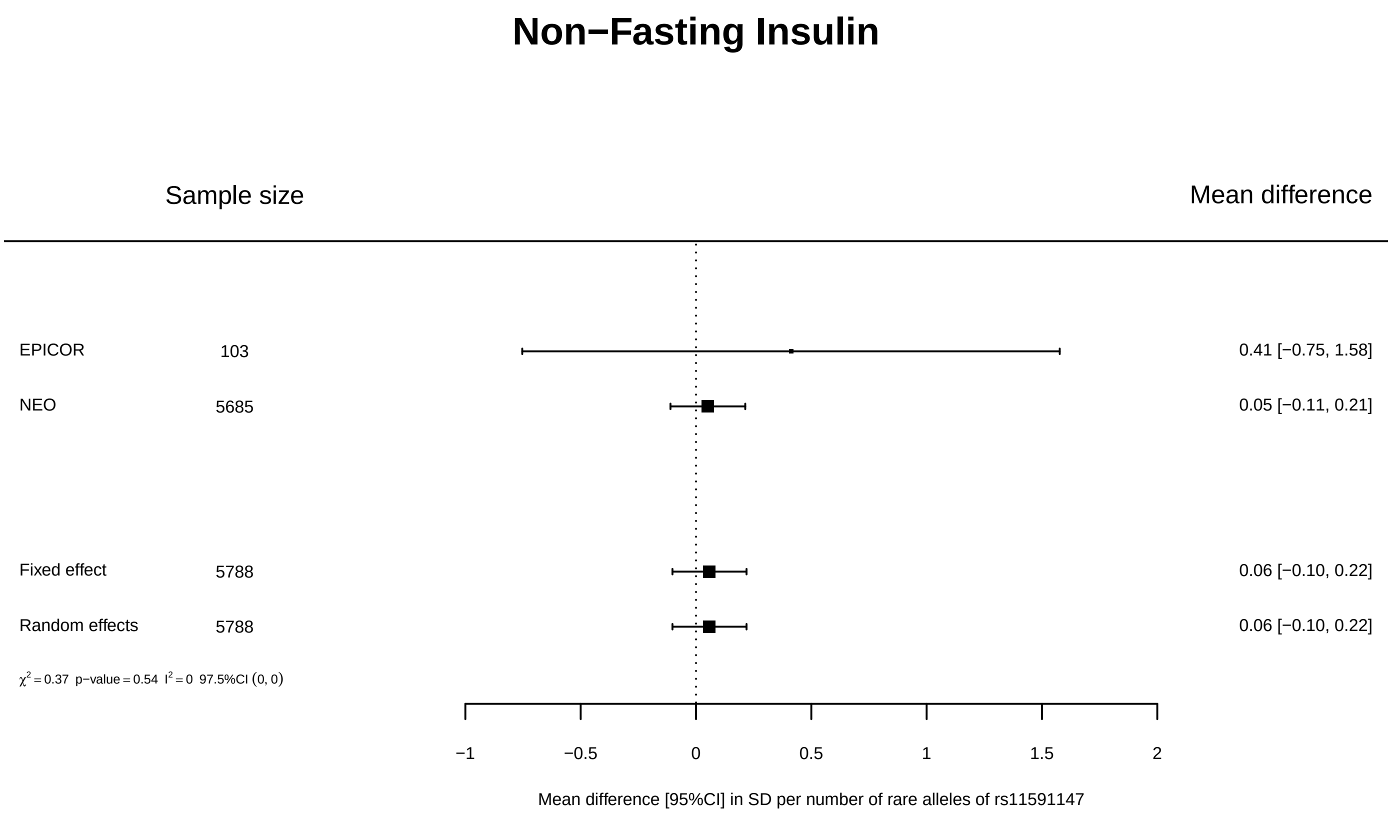

Figure 65: Mean difference of PCSK9 SNP rs11591147 on non–fasting insulin (SD) per number of rare alleles, with 95% confidence interval (CI). Results are pooled using a fixed and random effects model, irrespective of observed between study heterogeneity; measured as a two sided Q–test (χ^2) and the I^2 with one–sided 97.5% CI. Note the outcome was standardized based on the SD of the natural logarithm.

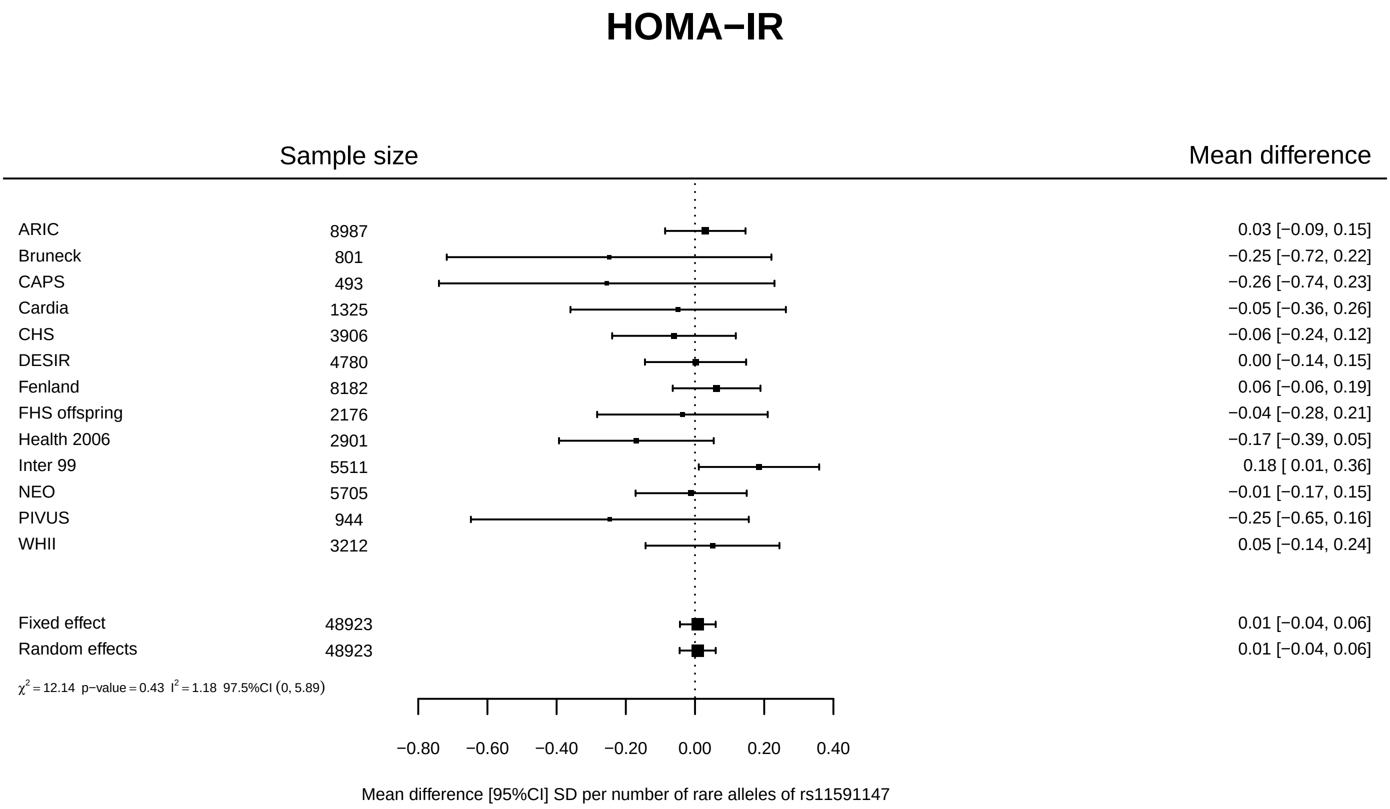

Figure 66: Mean difference of PCSK9 SNP rs11591147 on $HOMA\check{\ }IR$ (SD) per number of rare alleles, with 95% confidence interval (CI). Results are pooled using a fixed and random effects model, irrespective of observed between study heterogeneity; measured as a two sided Q–test (χ^2) and the I^2 with one–sided 97.5% CI. Note the outcome was standardized based on the SD of the natural logarithm.

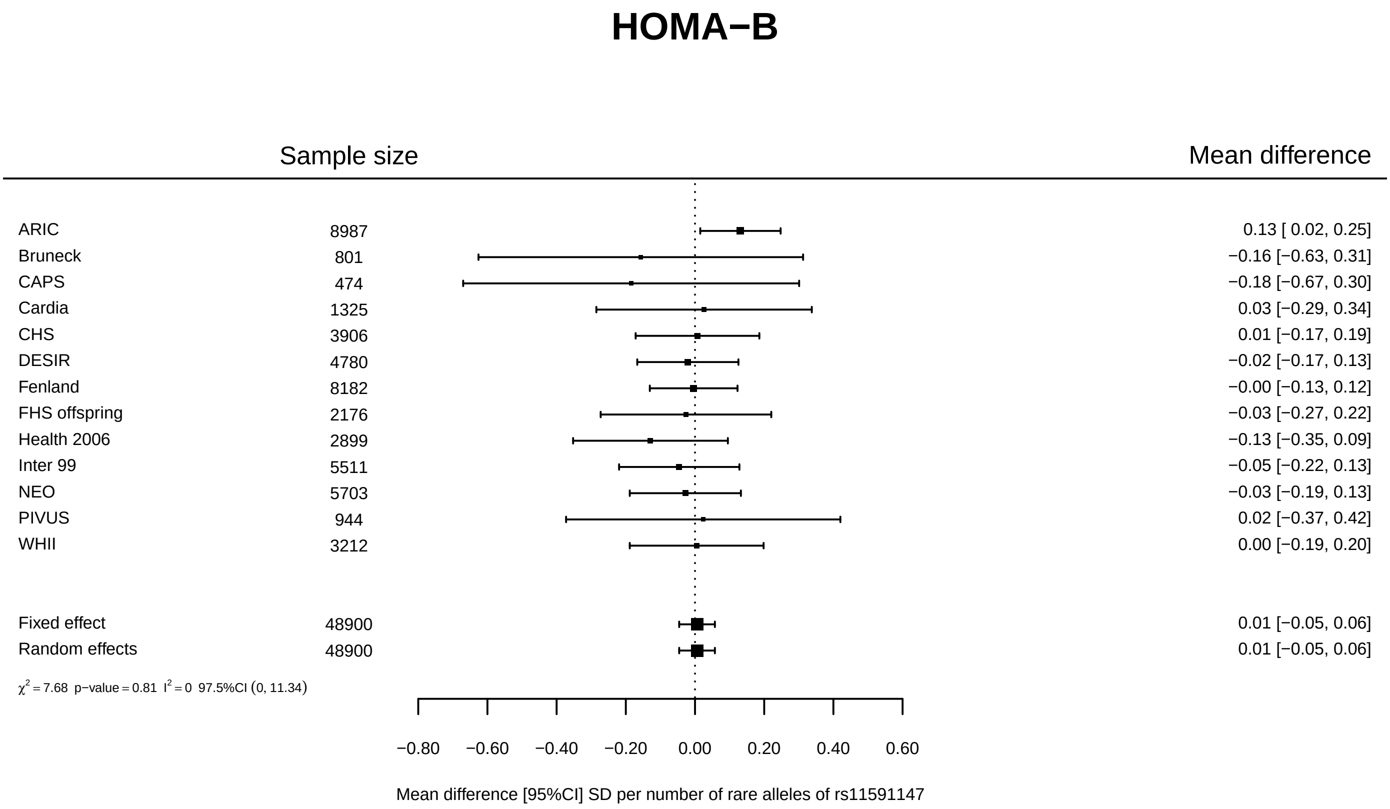

Figure 67: Mean difference of PCSK9 SNP rs11591147 on $HOMA\breve{}\beta$ (SD) per number of rare alleles, with 95% confidence interval (CI). Results are pooled using a fixed and random effects model, irrespective of observed between study heterogeneity; measured as a two sided Q–test (χ^2) and the I^2 with one–sided 97.5% CI. Note the outcome was standardized based on the SD of the natural logarithm.

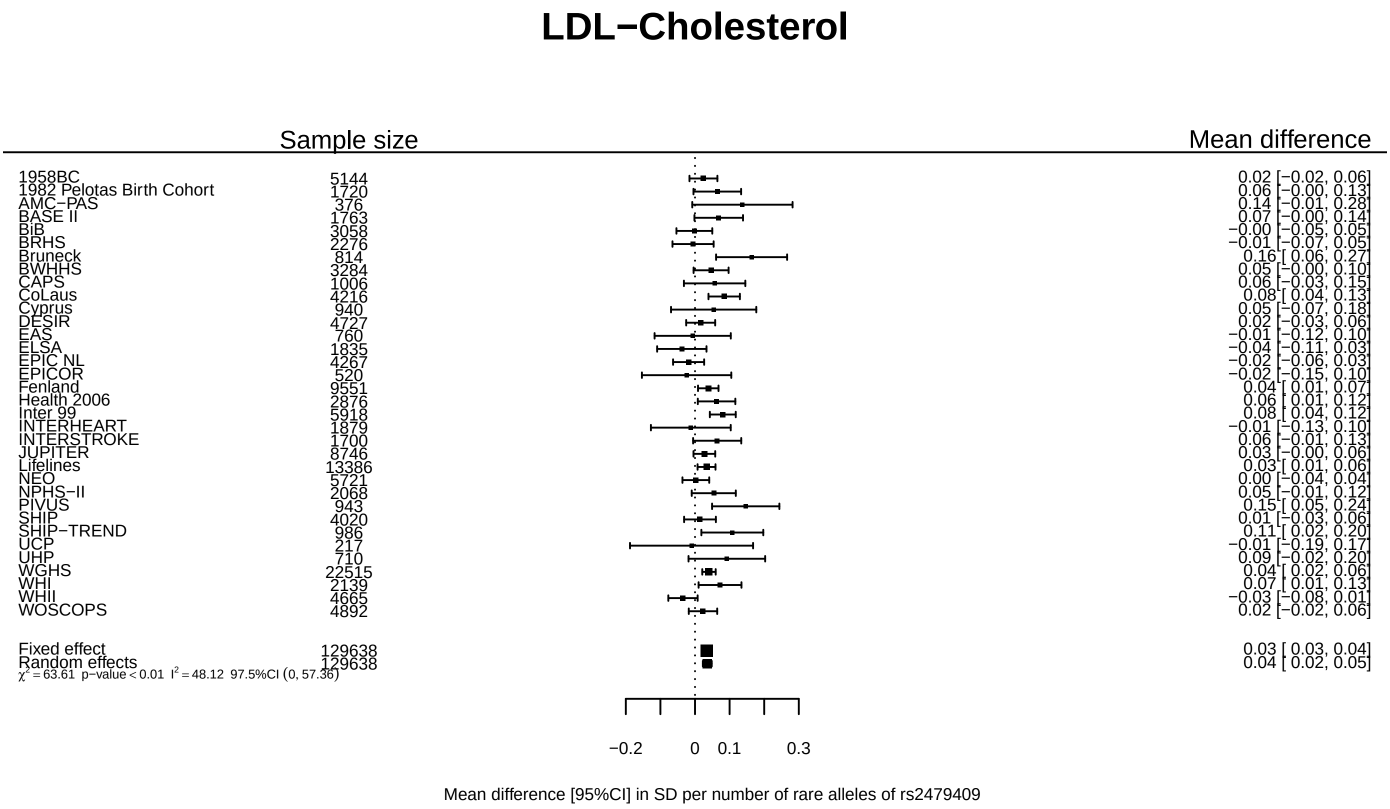

Figure 68: Mean difference of PCSK9 SNP rs2479409 on LDL–C (SD) per number of rare alleles, with 95% confidence interval (CI). Results are pooled using a fixed and random effects model, irrespective of observed between study heterogeneity; measured as a two sided Q–test (χ^2) and the I^2 with one–sided 97.5% CI.

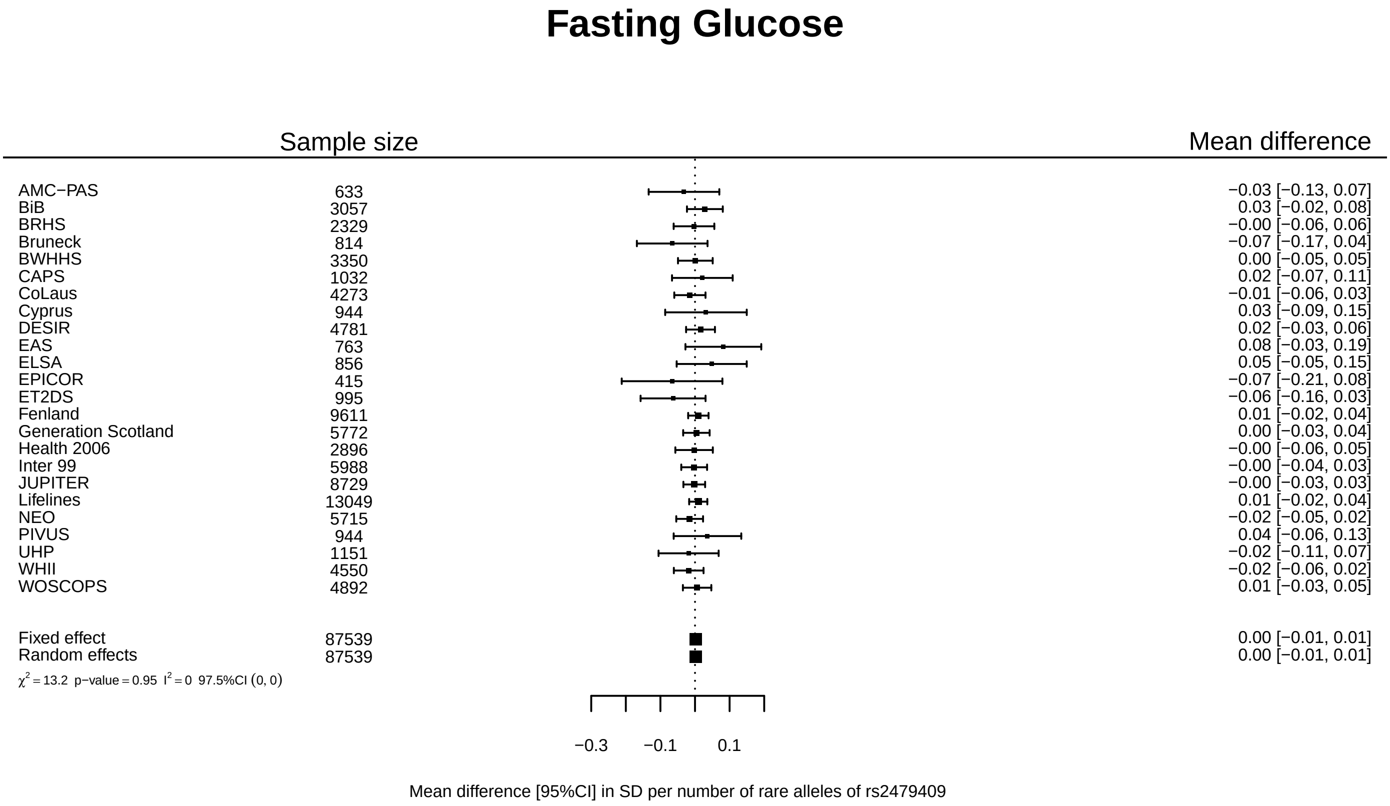

Figure 69: Mean difference of PCSK9 SNP rs2479409 on fasting glucose (SD) per number of rare alleles, with 95% confidence interval (CI). Results are pooled using a fixed and random effects model, irrespective of observed between study heterogeneity; measured as a two sided Q–test (χ^2) and the I^2 with one–sided 97.5% CI.

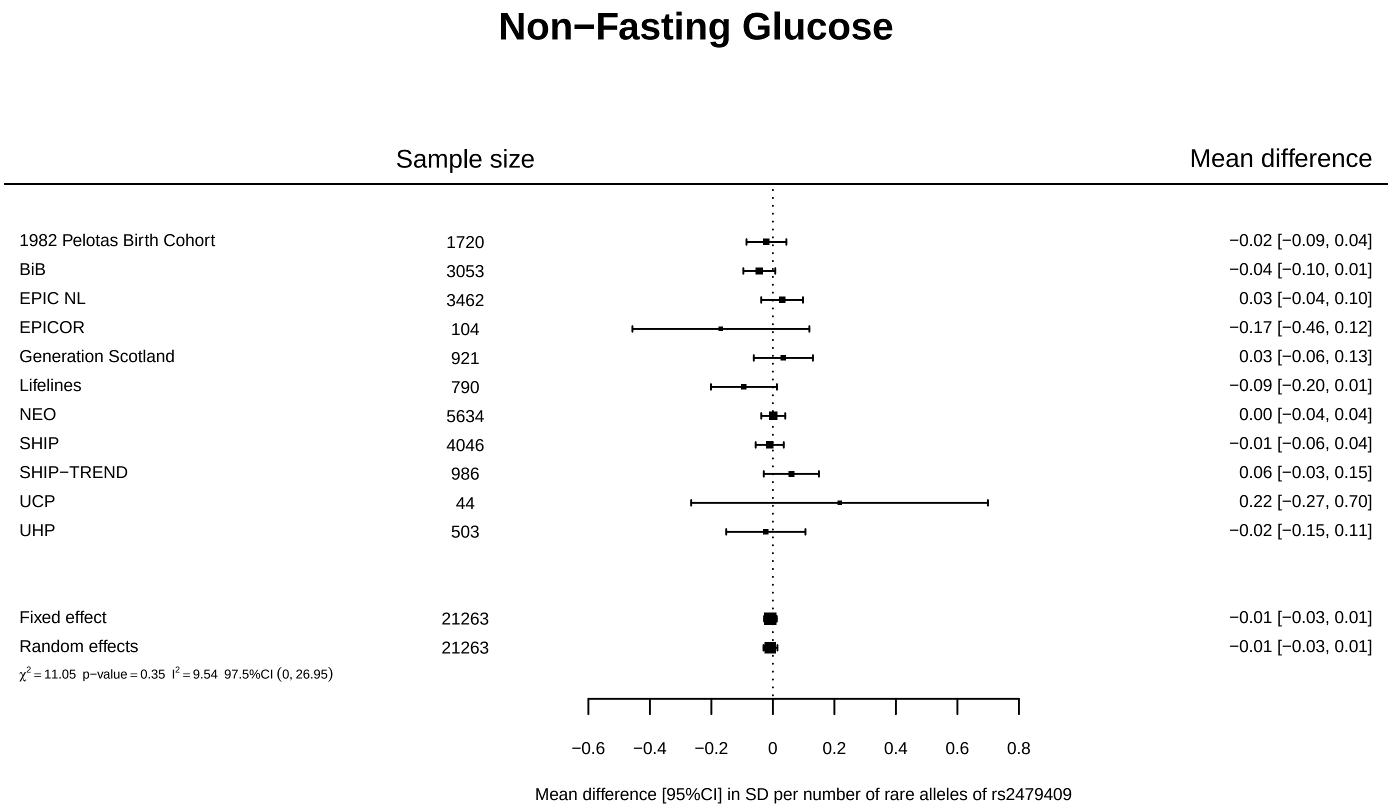

Figure 70: Mean difference of PCSK9 SNP rs2479409 on non–fasting glucose (SD) per number of rare alleles, with 95% confidence interval (CI). Results are pooled using a fixed and random effects model, irrespective of observed between study heterogeneity; measured as a two sided Q–test (χ^2) and the I^2 with one–sided 97.5% CI.

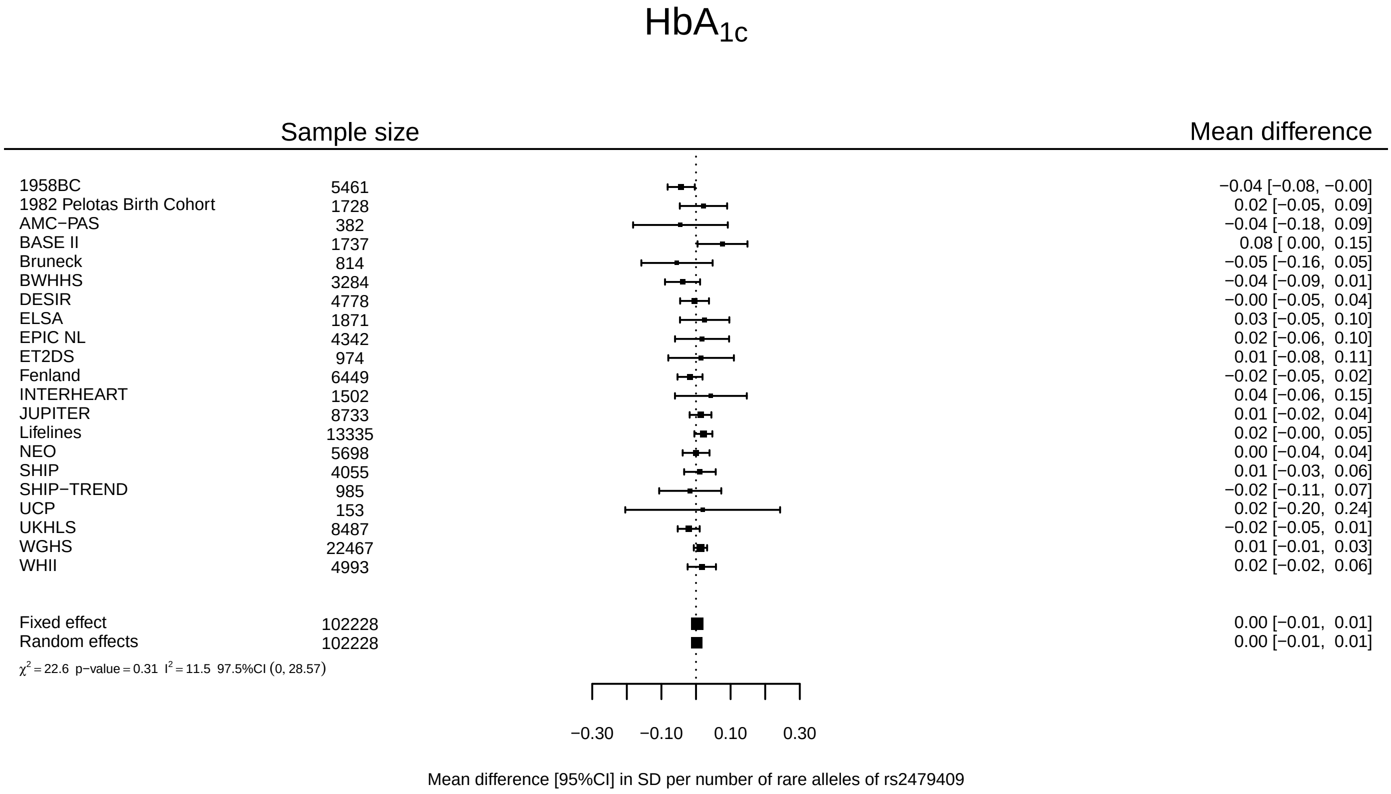

Figure 71: Mean difference of PCSK9 SNP rs2479409 on HbA_{1c} (SD) per number of rare alleles, with 95% confidence interval (CI). Results are pooled using a fixed and random effects model, irrespective of observed between study heterogeneity; measured as a two sided Q–test (χ^2) and the I^2 with one–sided 97.5% CI.

Body Weight

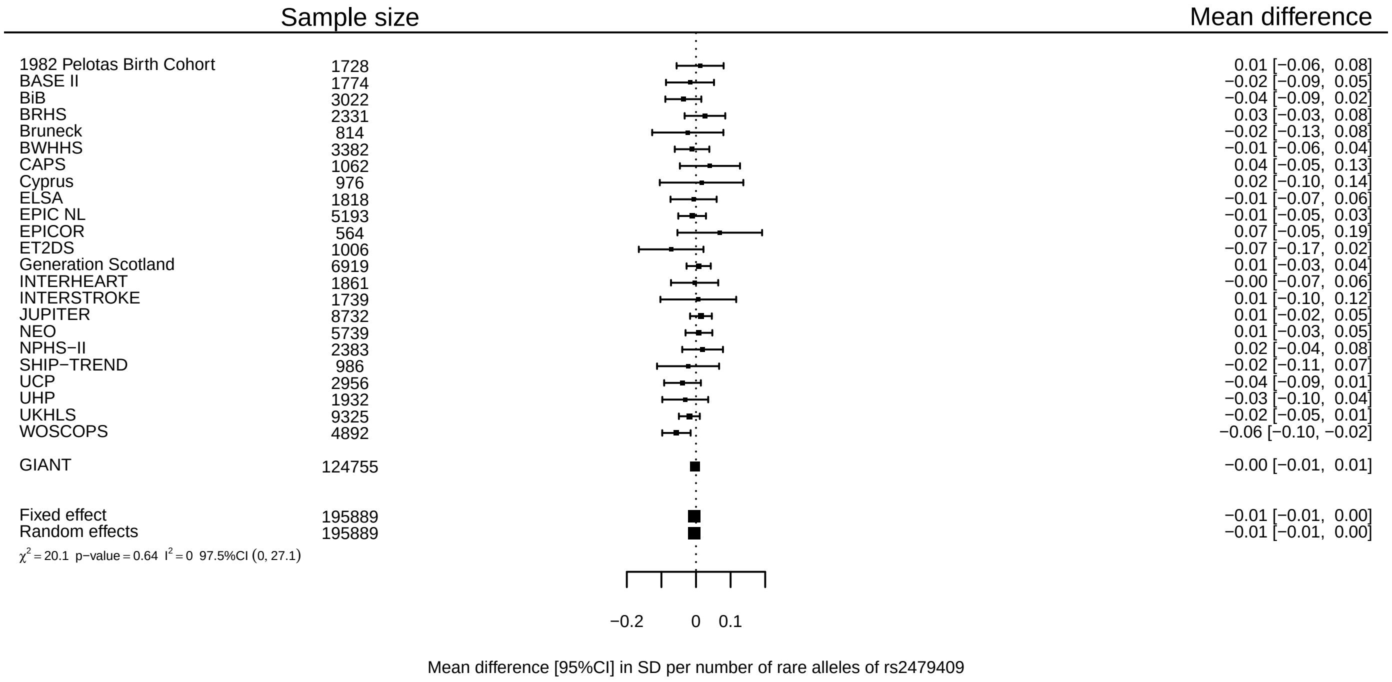

Figure 72: Mean difference of PCSK9 SNP rs2479409 on body weight (SD) per number of rare alleles, with 95% confidence interval (CI). Results are pooled using a fixed and random effects model, irrespective of observed between study heterogeneity; measured as a two sided Q–test (χ^2) and the I^2 with one–sided 97.5% CI.

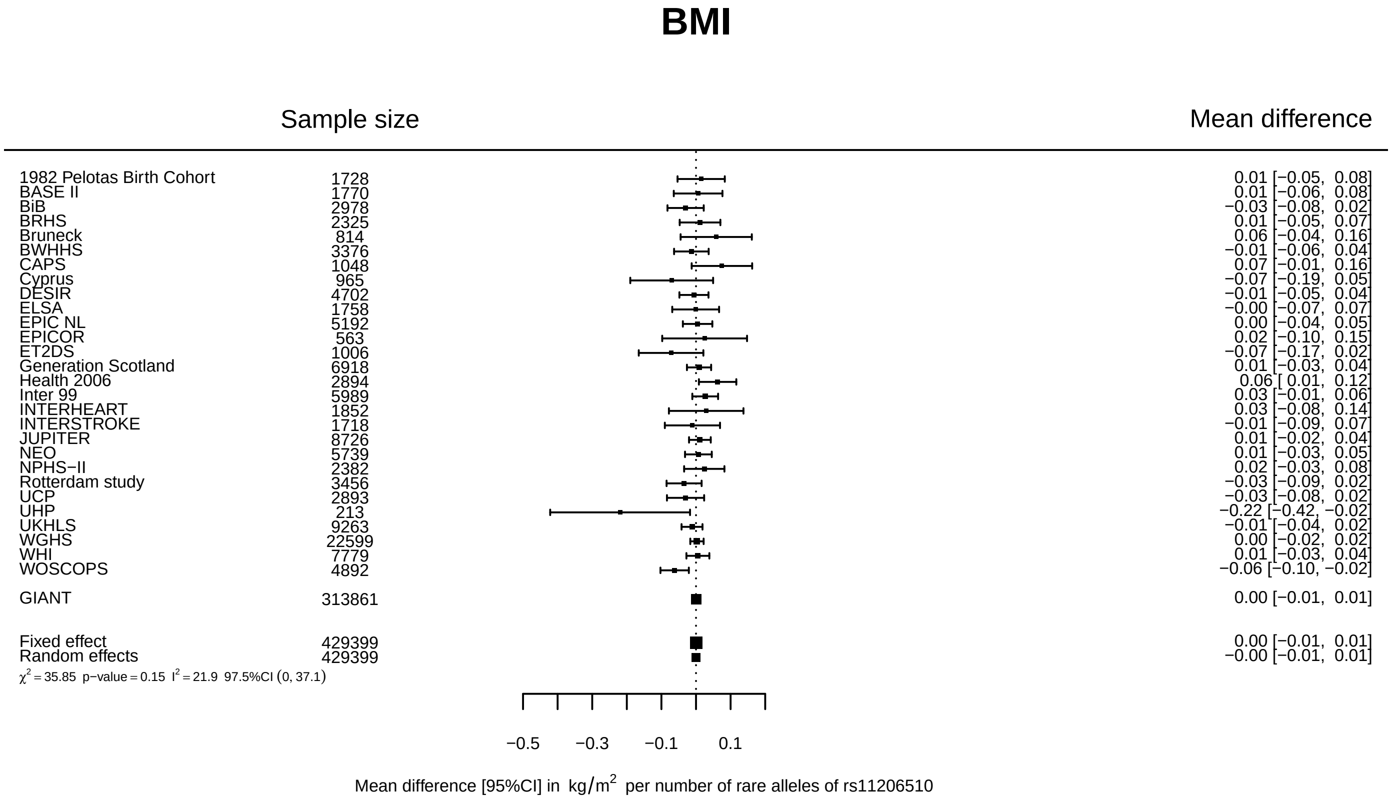

Figure 73: Mean difference of PCSK9 SNP rs2479409 on BMI (SD) per number of rare alleles, with 95% confidence interval (CI). Results are pooled using a fixed and random effects model, irrespective of observed between study heterogeneity; measured as a two sided Q–test (χ^2) and the I^2 with one–sided 97.5% CI.

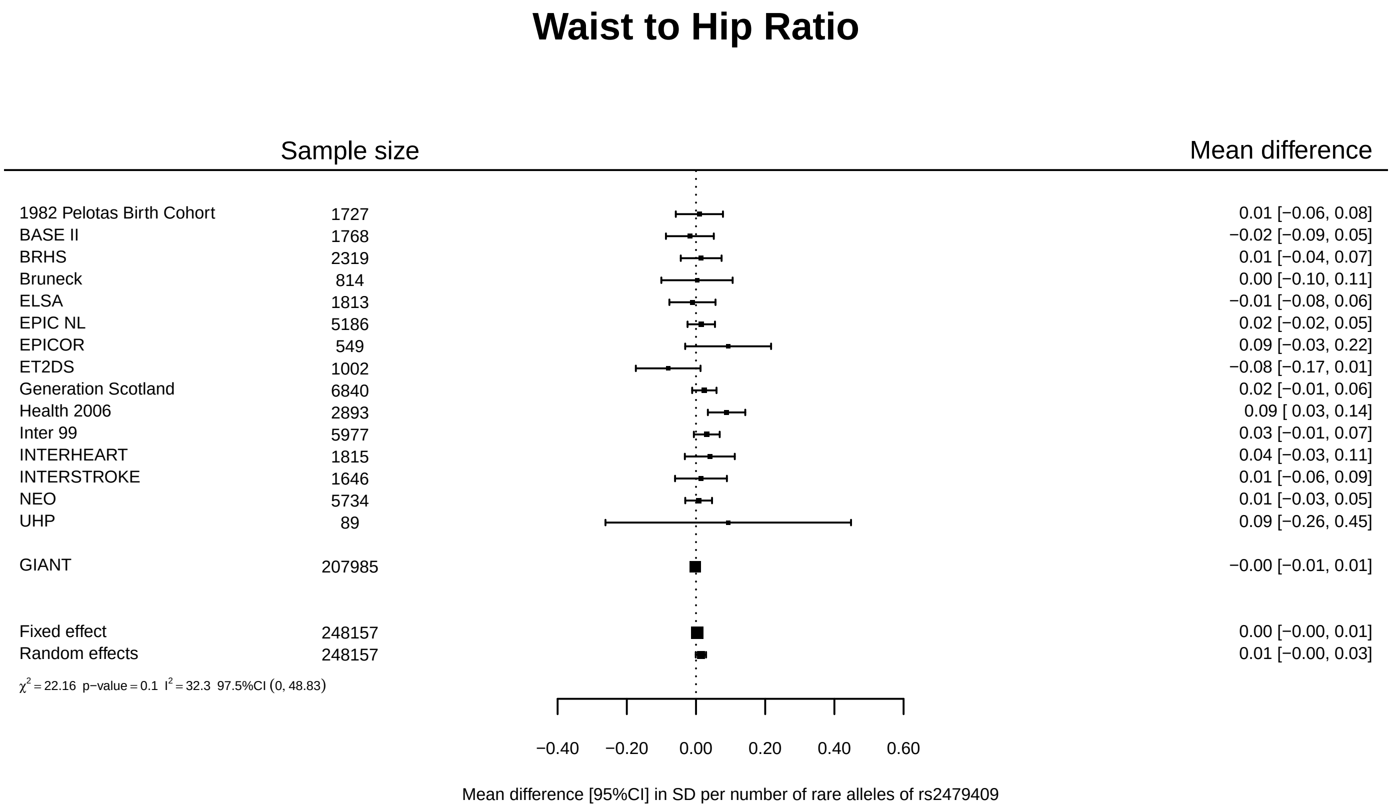

Figure 74: Mean difference of PCSK9 SNP rs2479409 on waist to hip ratio (SD) per number of rare alleles, with 95% confidence interval (CI). Results are pooled using a fixed and random effects model, irrespective of observed between study heterogeneity; measured as a two sided Q–test (χ^2) and the I^2 with one–sided 97.5% CI.

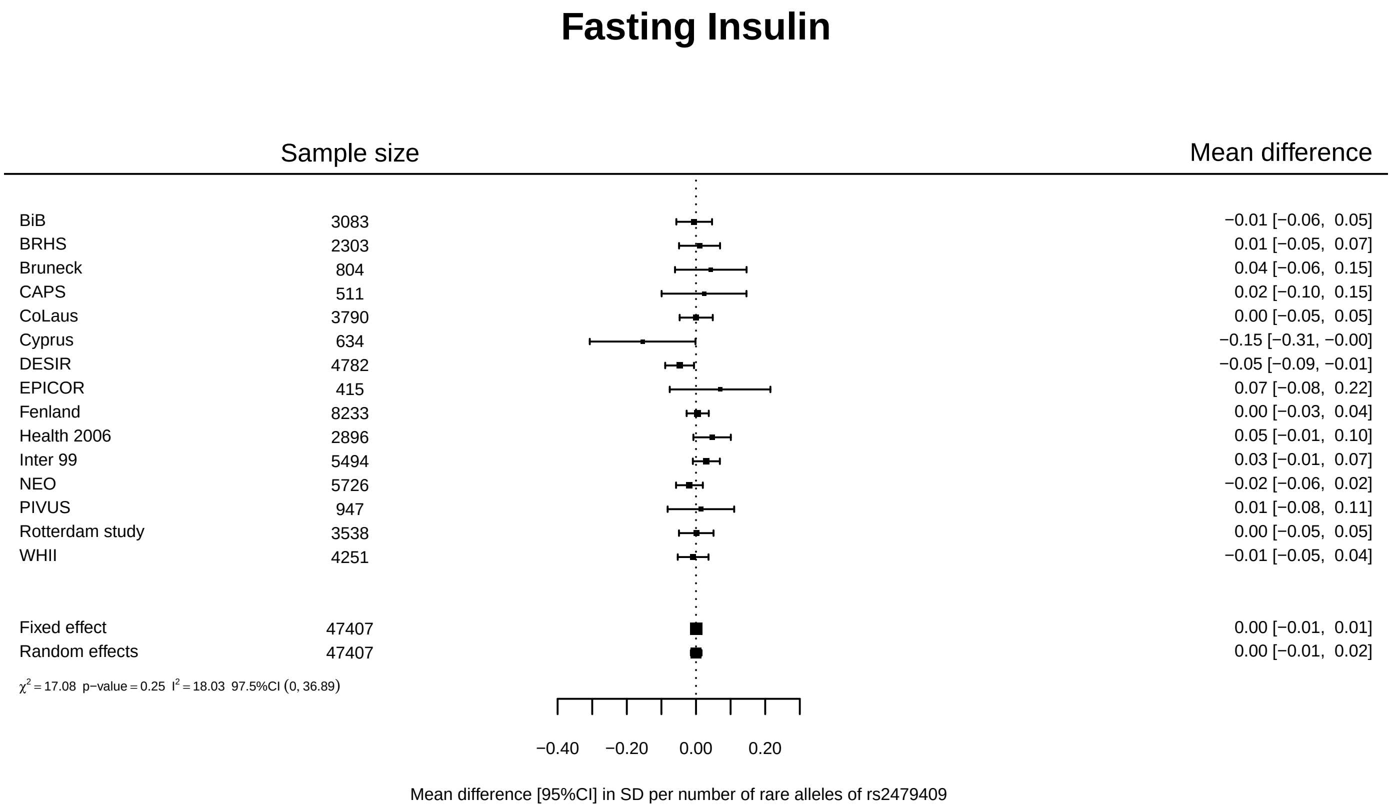

Figure 75: Mean difference of PCSK9 SNP rs2479409 on fasting insulin (SD) per number of rare alleles, with 95% confidence interval (CI). Results are pooled using a fixed and random effects model, irrespective of observed between study heterogeneity; measured as a two sided Q–test (χ^2) and the I^2 with one–sided 97.5% CI. Note the outcome was standardized based on the SD of the natural logarithm.

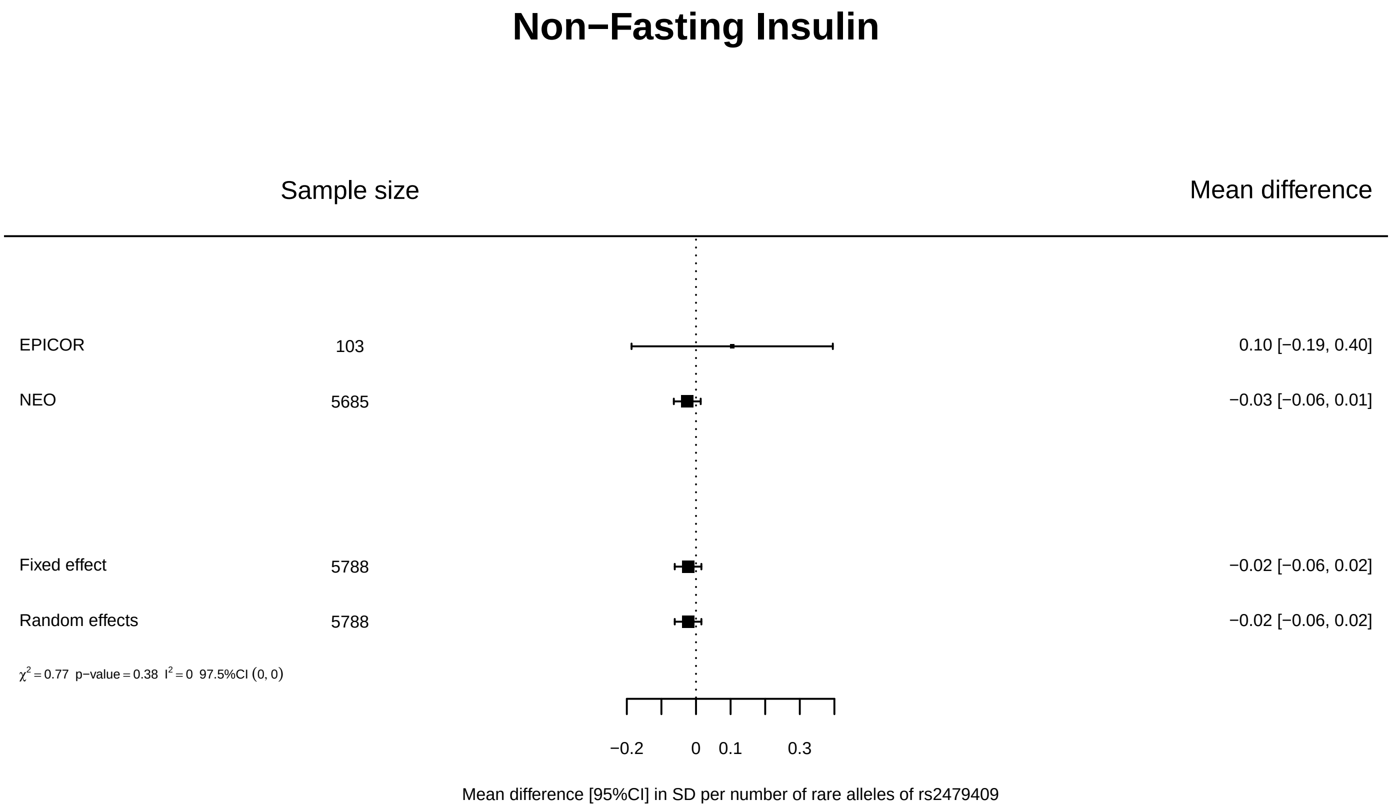

Figure 76: Mean difference of PCSK9 SNP rs2479409 on non–fasting insulin (SD) per number of rare alleles, with 95% confidence interval (CI). Results are pooled using a fixed and random effects model, irrespective of observed between study heterogeneity; measured as a two sided Q–test (χ^2) and the I^2 with one–sided 97.5% CI. Note the outcome was standardized based on the SD of the natural logarithm.

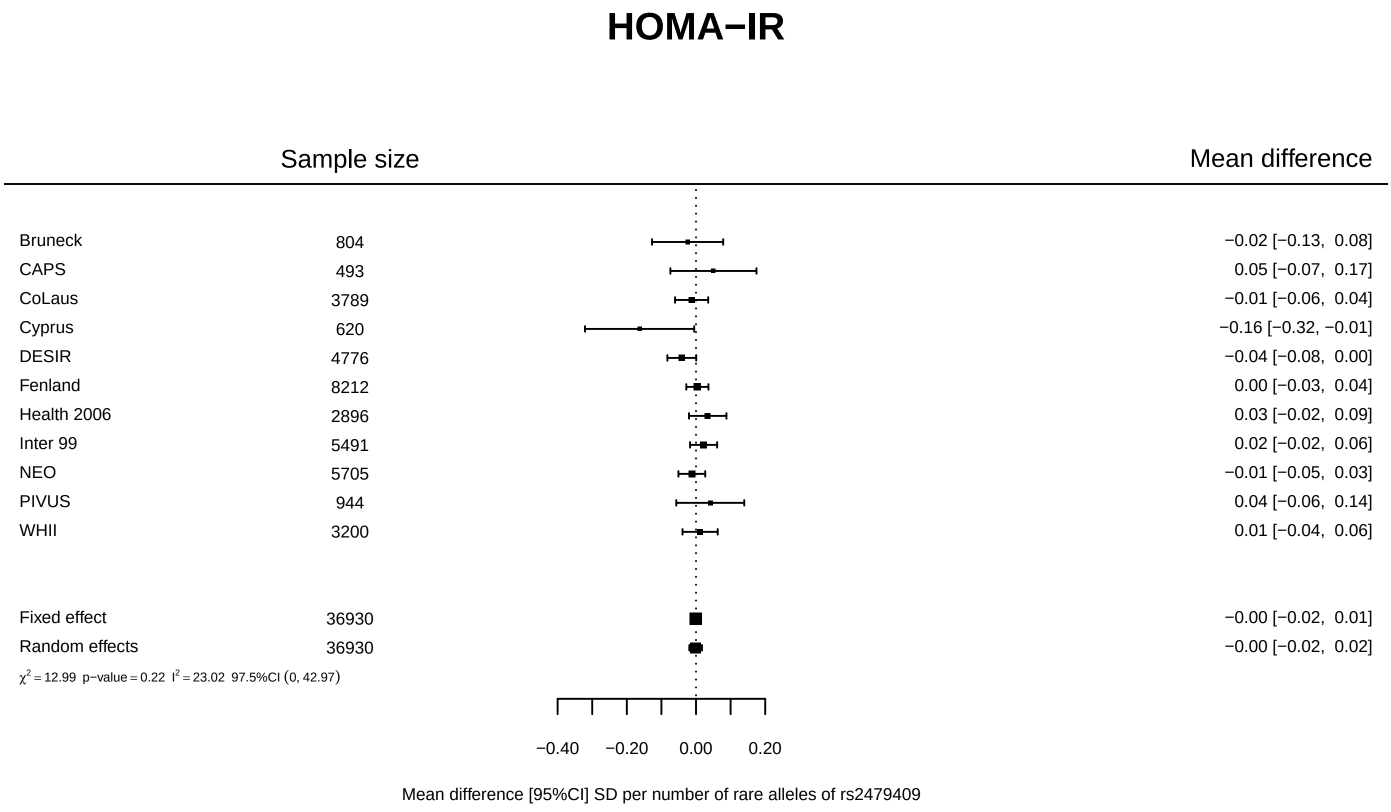

Figure 77: Mean difference of PCSK9 SNP rs2479409 on $HOMA\breve{}IR$ (SD) per number of rare alleles, with 95% confidence interval (CI). Results are pooled using a fixed and random effects model, irrespective of observed between study heterogeneity; measured as a two sided Q–test (χ^2) and the I^2 with one–sided 97.5% CI. Note the outcome was standardized based on the SD of the natural logarithm.

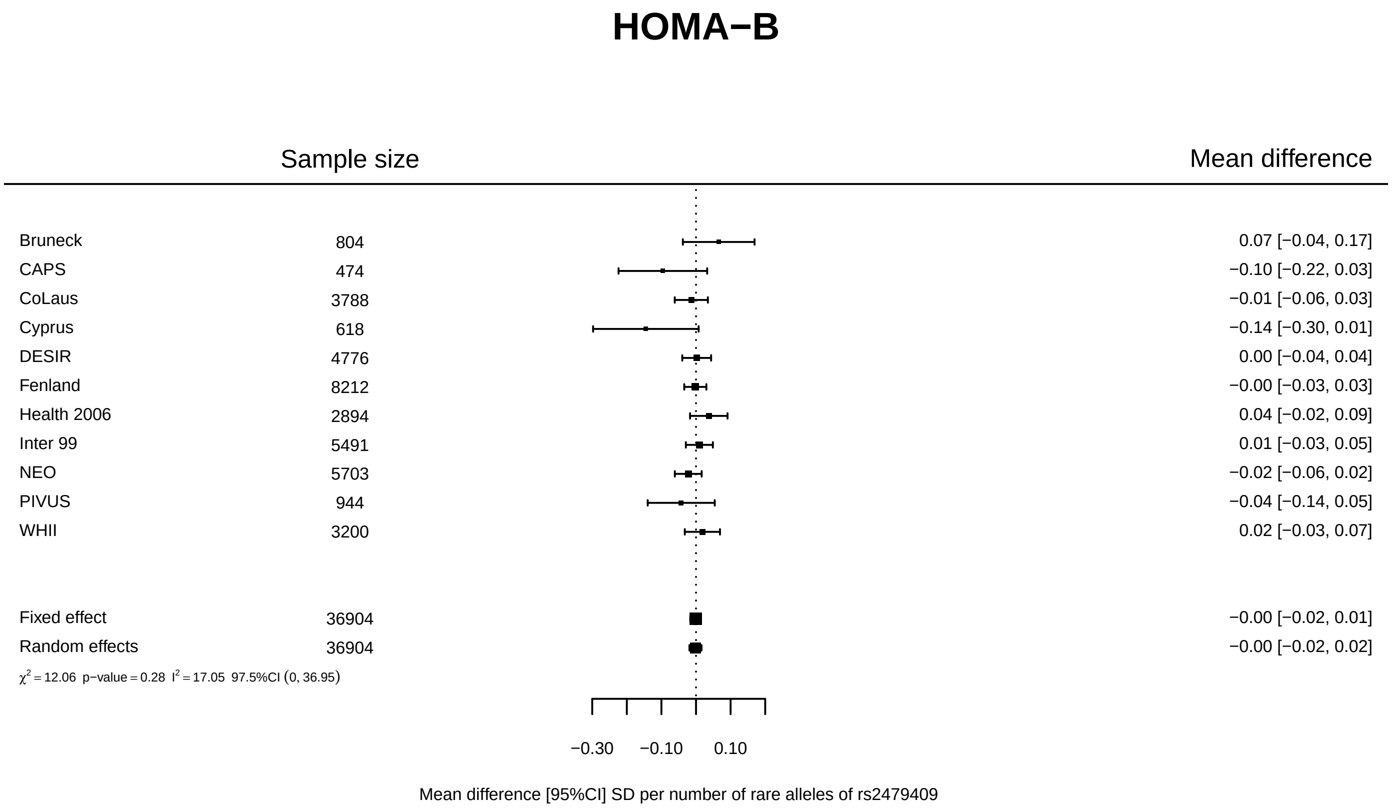

Figure 78: Mean difference of PCSK9 SNP rs2479409 on $HOMA\breve{}\beta$ (SD) per number of rare alleles, with 95% confidence interval (CI). Results are pooled using a fixed and random effects model, irrespective of observed between study heterogeneity; measured as a two sided Q–test (χ^2) and the I^2 with one–sided 97.5% CI. Note the outcome was standardized based on the SD of the natural logarithm.

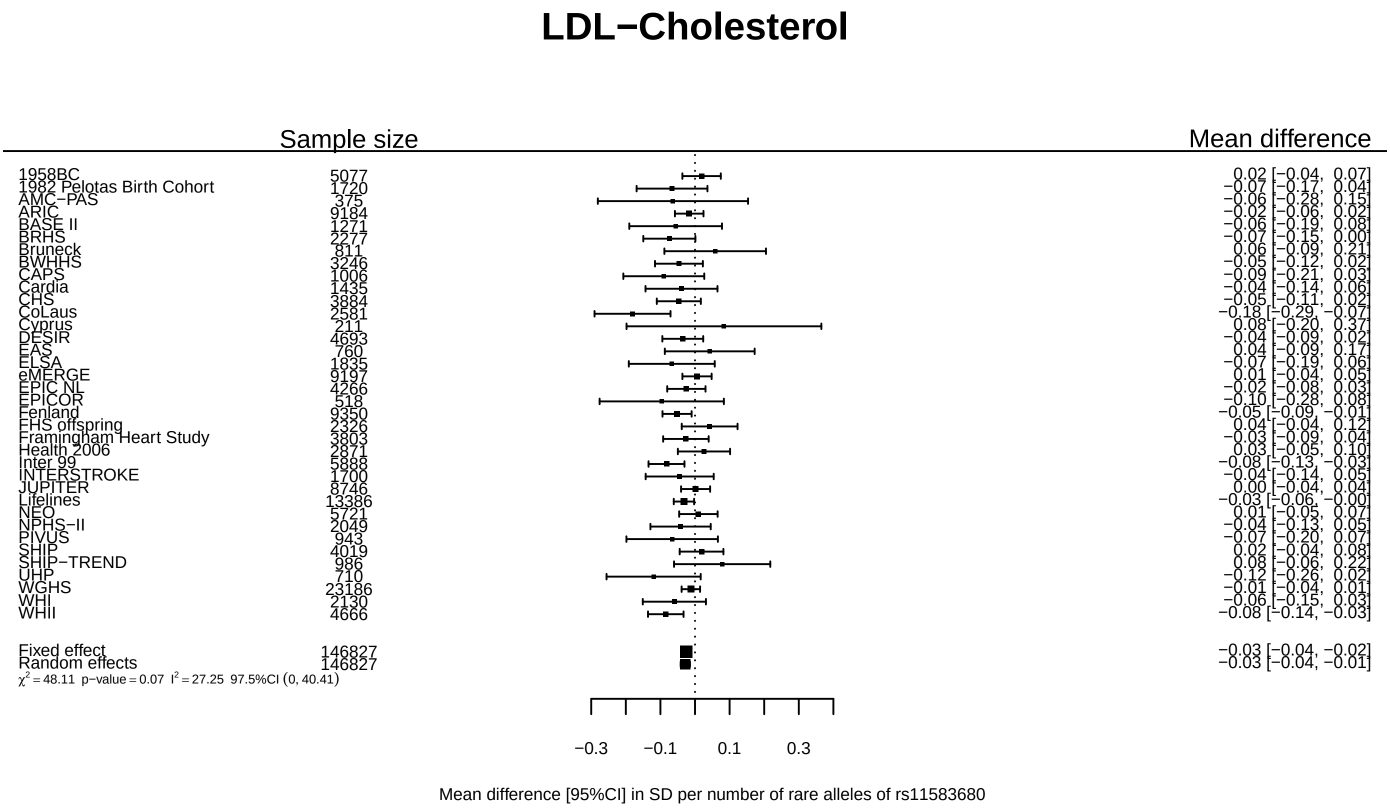

Figure 79: Mean difference of PCSK9 SNP rs11583680 on LDL–C (SD) per number of rare alleles, with 95% confidence interval (CI). Results are pooled using a fixed and random effects model, irrespective of observed between study heterogeneity; measured as a two sided Q–test (χ^2) and the I^2 with one–sided 97.5% CI.

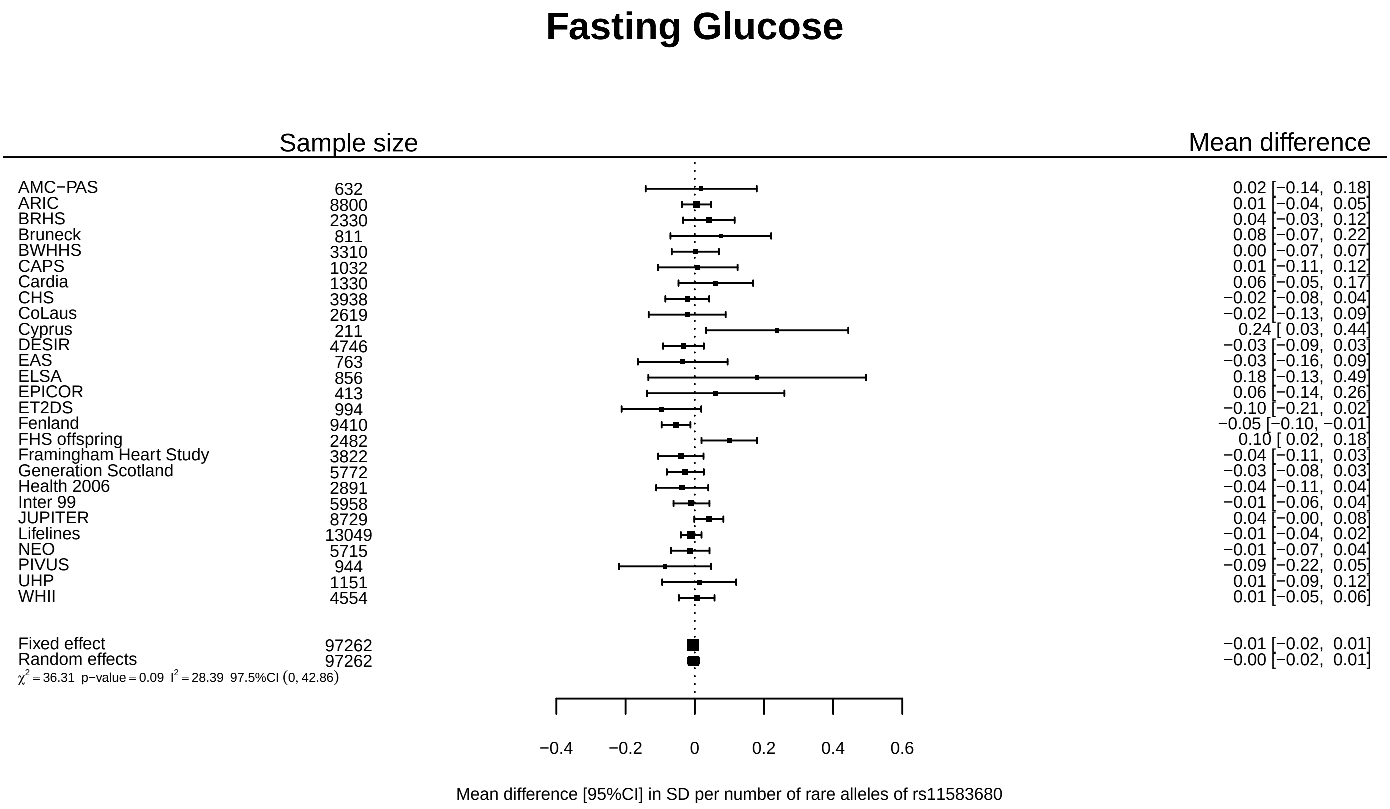

Figure 80: Mean difference of PCSK9 SNP rs11583680 on fasting glucose (SD) per number of rare alleles, with 95% confidence interval (CI). Results are pooled using a fixed and random effects model, irrespective of observed between study heterogeneity; measured as a two sided Q–test (χ^2) and the I^2 with one–sided 97.5% CI.

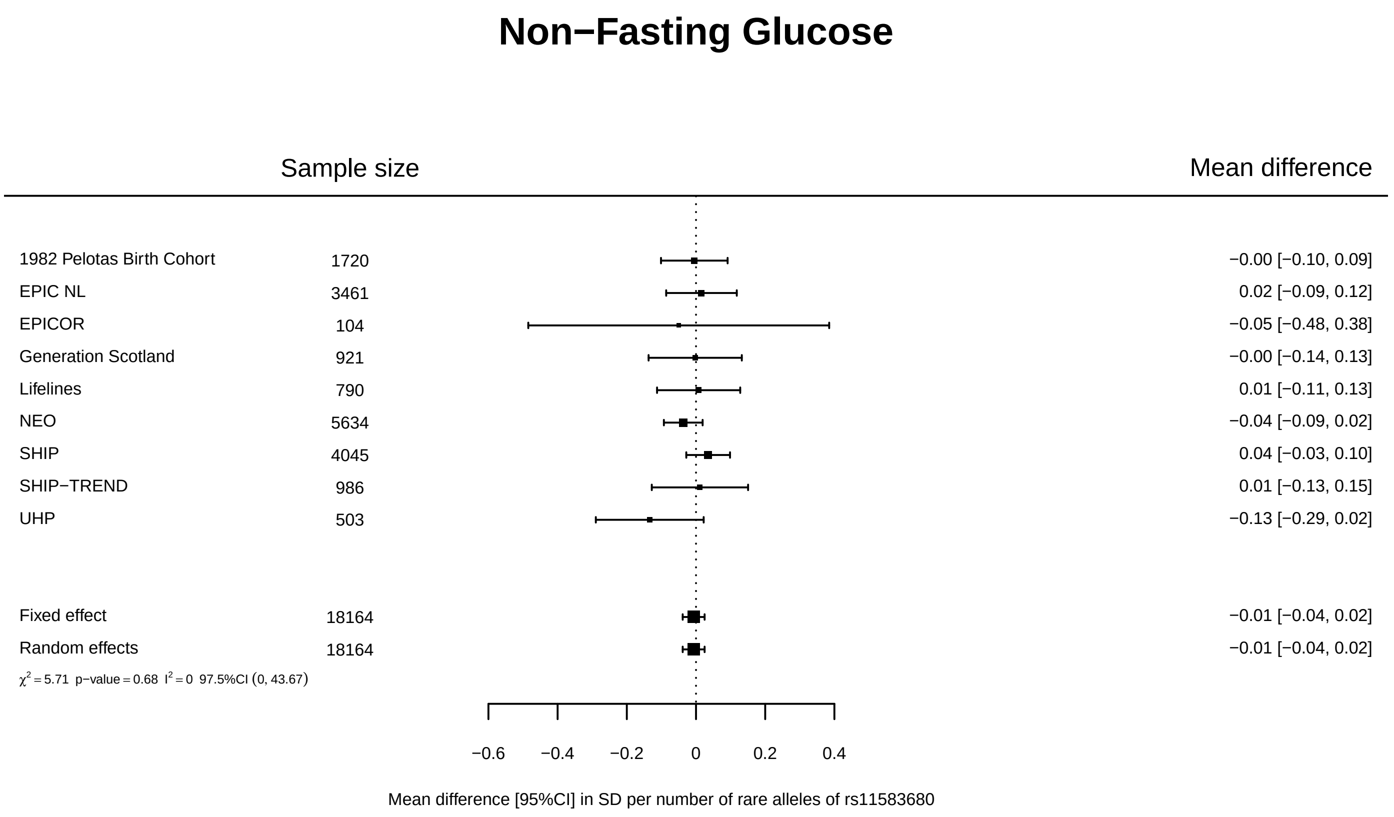

Figure 81: Mean difference of PCSK9 SNP rs11583680 on non–fasting glucose (SD) per number of rare alleles, with 95% confidence interval (CI). Results are pooled using a fixed and random effects model, irrespective of observed between study heterogeneity; measured as a two sided Q–test (χ^2) and the I^2 with one–sided 97.5% CI.

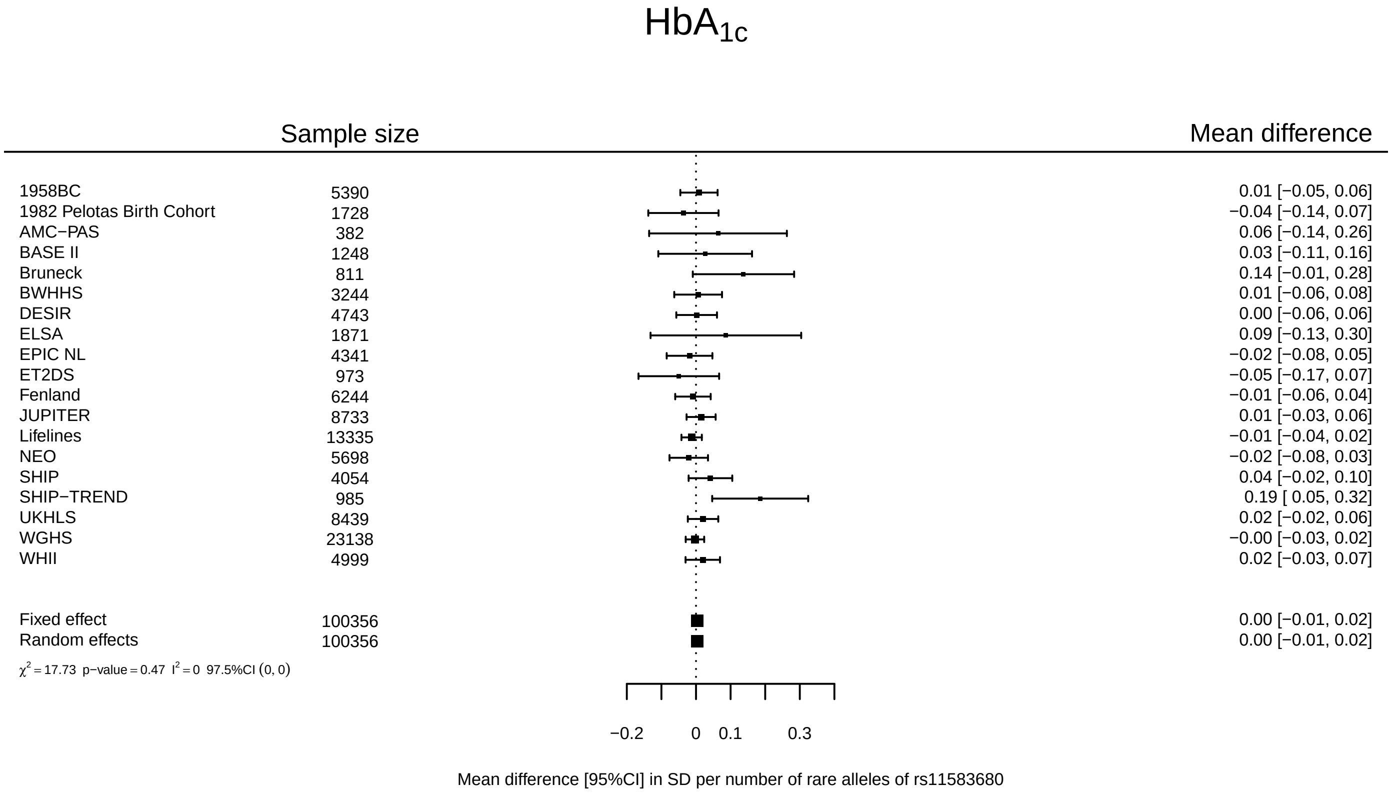

Figure 82: Mean difference of PCSK9 SNP rs11583680 on HbA_{1c} (SD) per number of rare alleles, with 95% confidence interval (CI). Results are pooled using a fixed and random effects model, irrespective of observed between study heterogeneity; measured as a two sided Q–test (χ^2) and the I^2 with one–sided 97.5% CI.

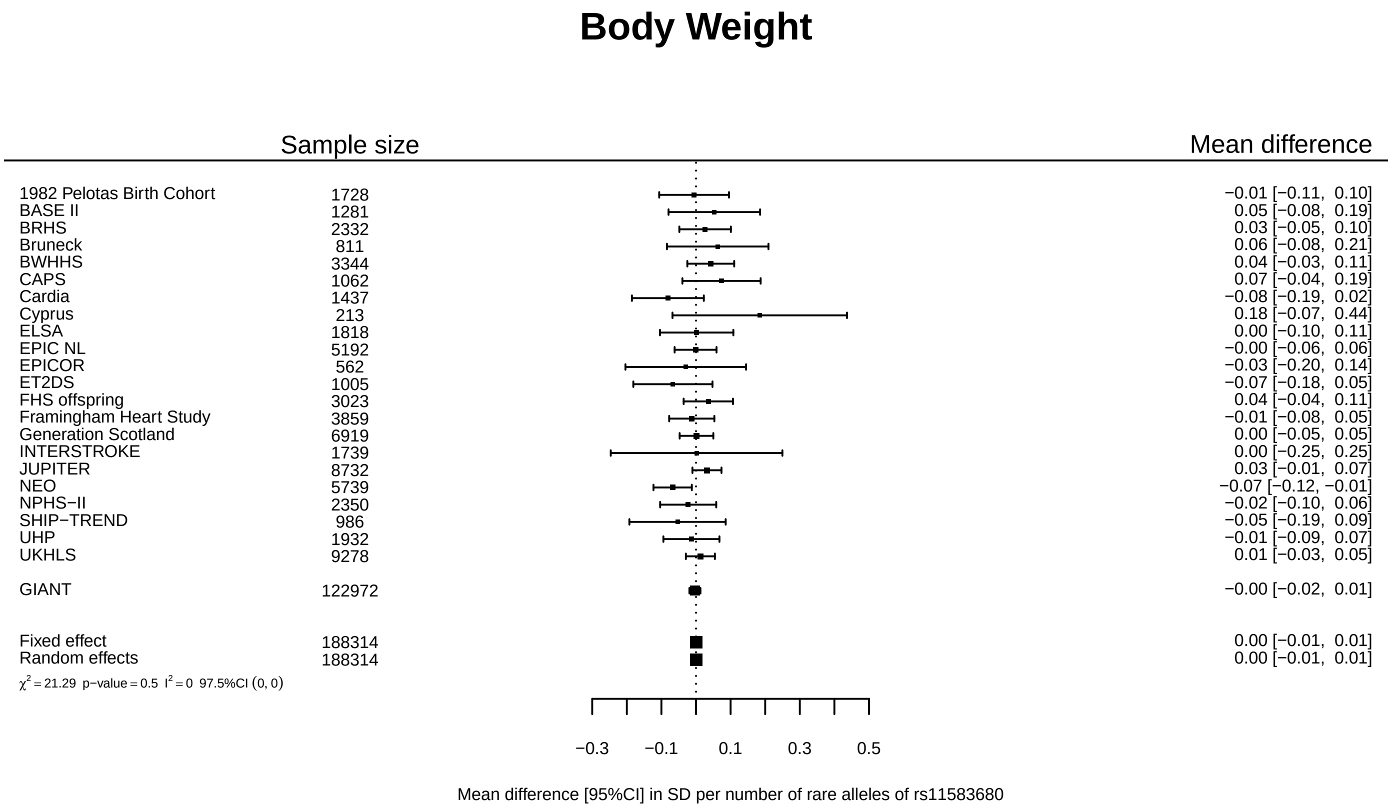

Figure 83: Mean difference of PCSK9 SNP rs11583680 on body weight (SD) per number of rare alleles, with 95% confidence interval (CI). Results are pooled using a fixed and random effects model, irrespective of observed between study heterogeneity; measured as a two sided Q–test (χ^2) and the I^2 with one–sided 97.5% CI.

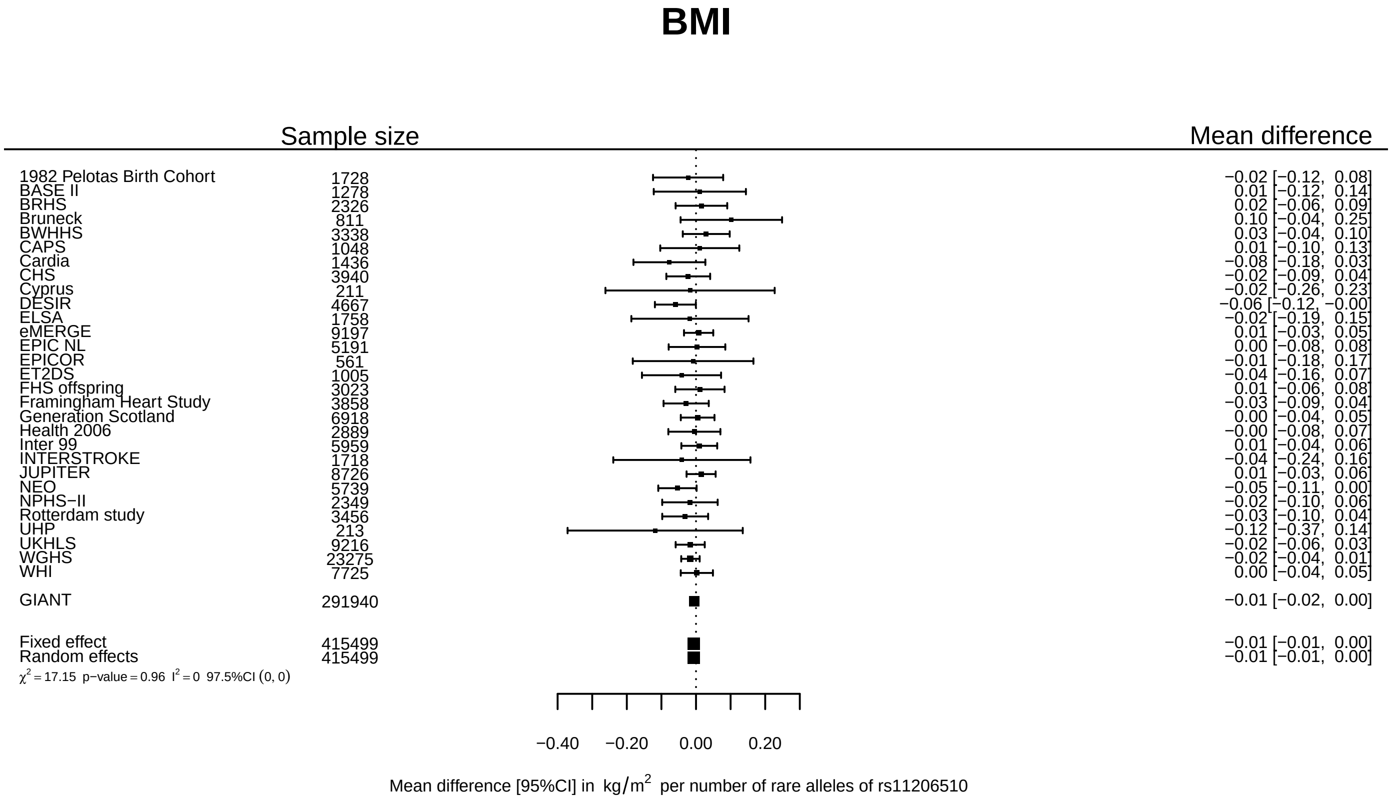

Figure 84: Mean difference of PCSK9 SNP rs11583680 on BMI (SD) per number of rare alleles, with 95% confidence interval (CI). Results are pooled using a fixed and random effects model, irrespective of observed between study heterogeneity; measured as a two sided Q–test (χ^2) and the I^2 with one–sided 97.5% CI.

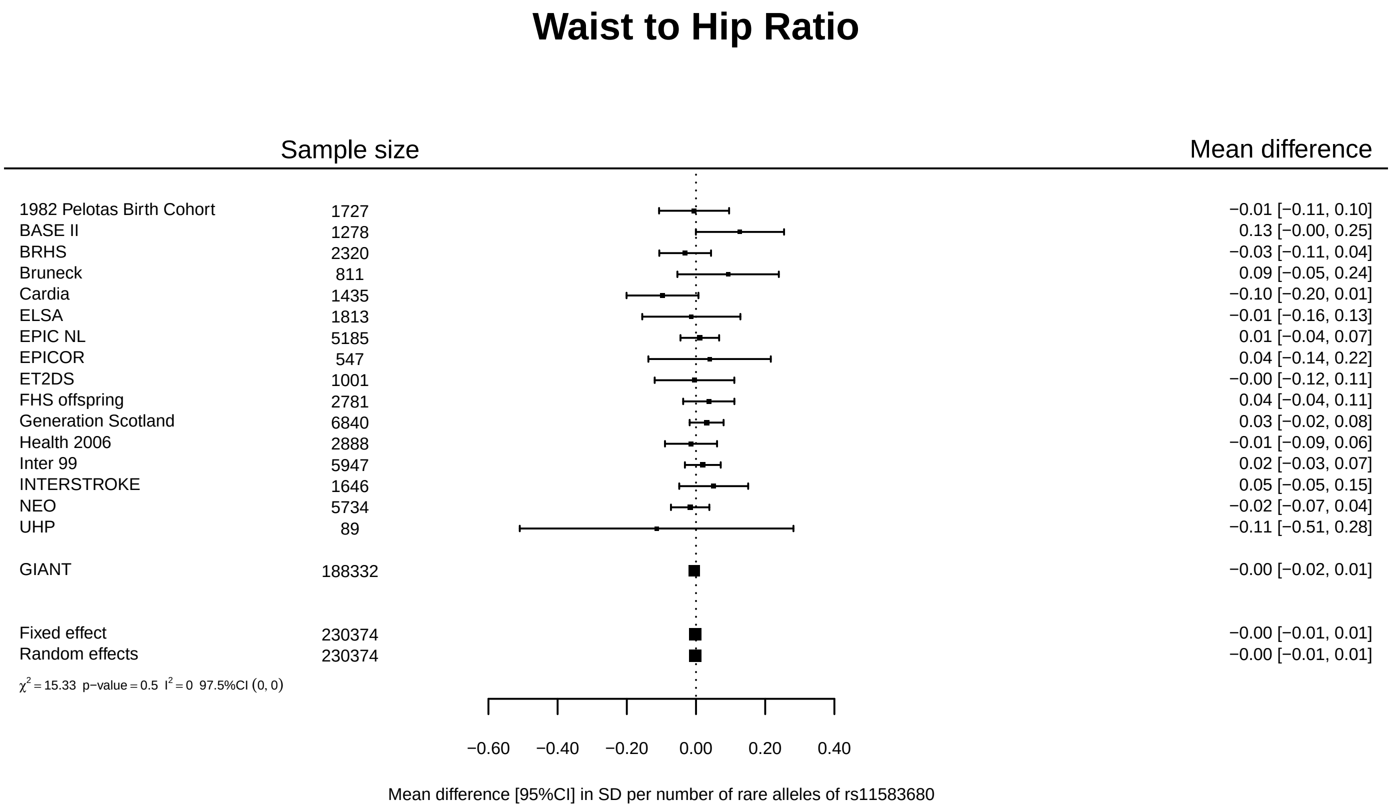

Figure 85: Mean difference of PCSK9 SNP rs11583680 on waist to hip ratio (SD) per number of rare alleles, with 95% confidence interval (CI). Results are pooled using a fixed and random effects model, irrespective of observed between study heterogeneity; measured as a two sided Q–test (χ^2) and the I^2 with one–sided 97.5% CI.

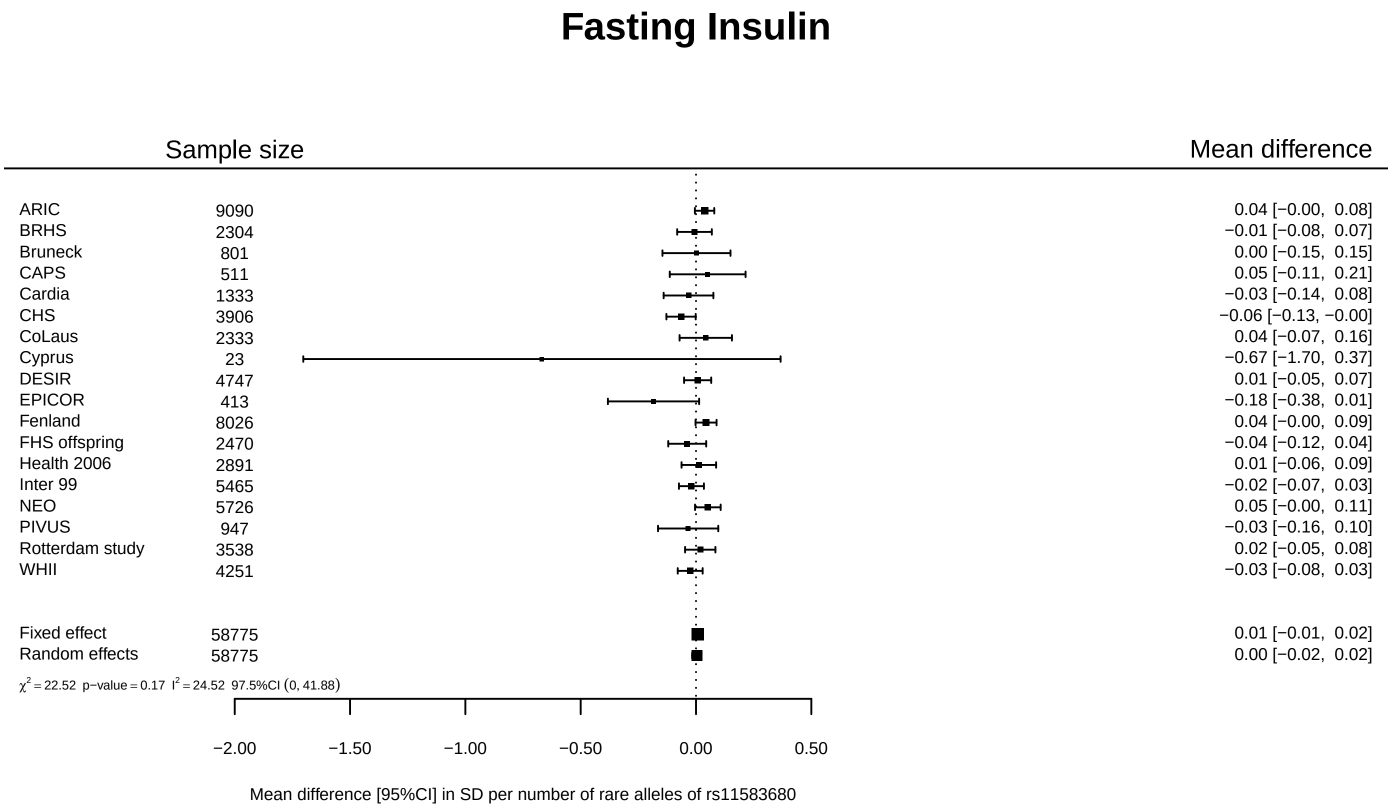

Figure 86: Mean difference of PCSK9 SNP rs11583680 on fasting insulin (SD) per number of rare alleles, with 95% confidence interval (CI). Results are pooled using a fixed and random effects model, irrespective of observed between study heterogeneity; measured as a two sided Q–test (χ^2) and the I^2 with one–sided 97.5% CI. Note the outcome was standardized based on the SD of the natural logarithm.

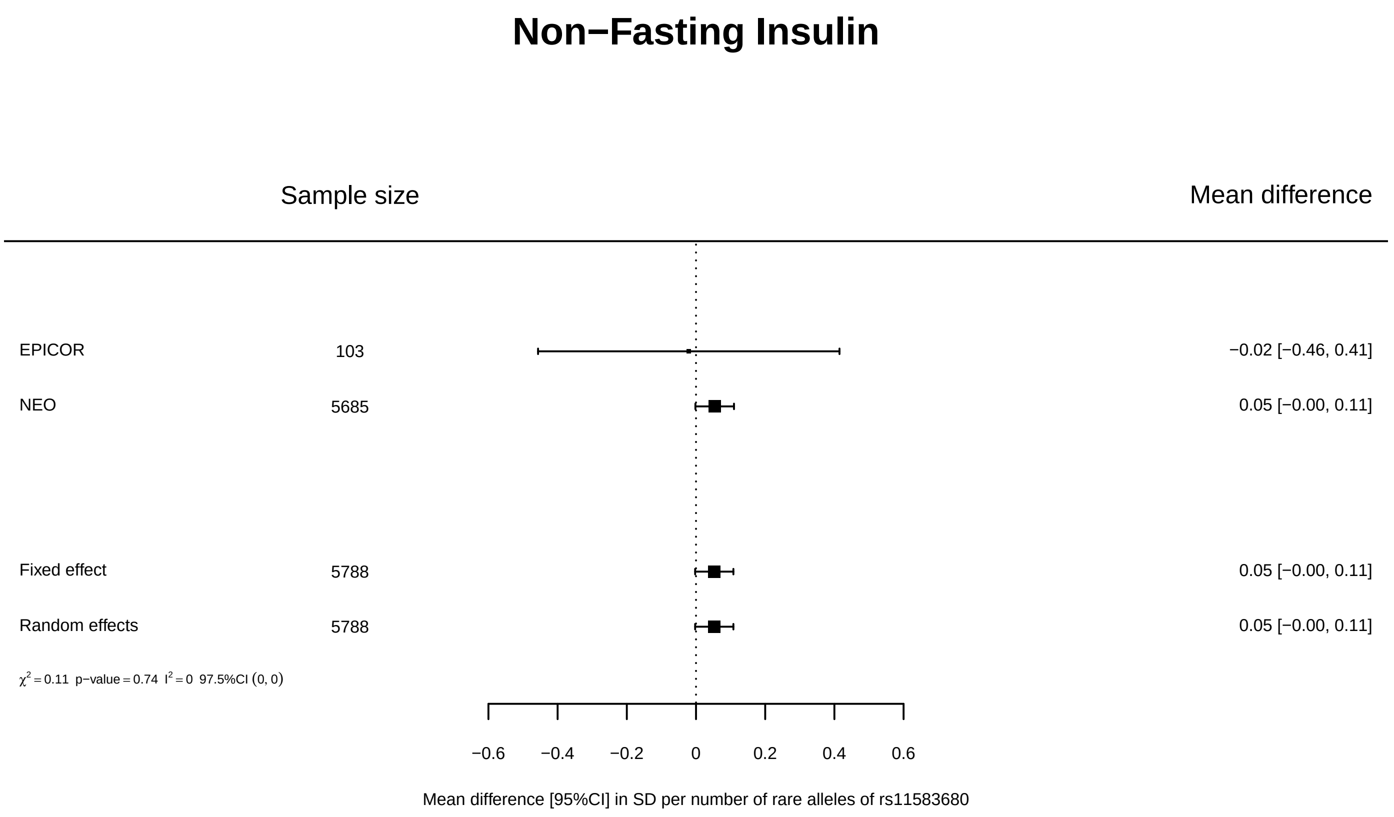

Figure 87: Mean difference of PCSK9 SNP rs11583680 on non–fasting insulin (SD) per number of rare alleles, with 95% confidence interval (CI). Results are pooled using a fixed and random effects model, irrespective of observed between study heterogeneity; measured as a two sided Q–test (χ^2) and the I^2 with one–sided 97.5% CI. Note the outcome was standardized based on the SD of the natural logarithm.

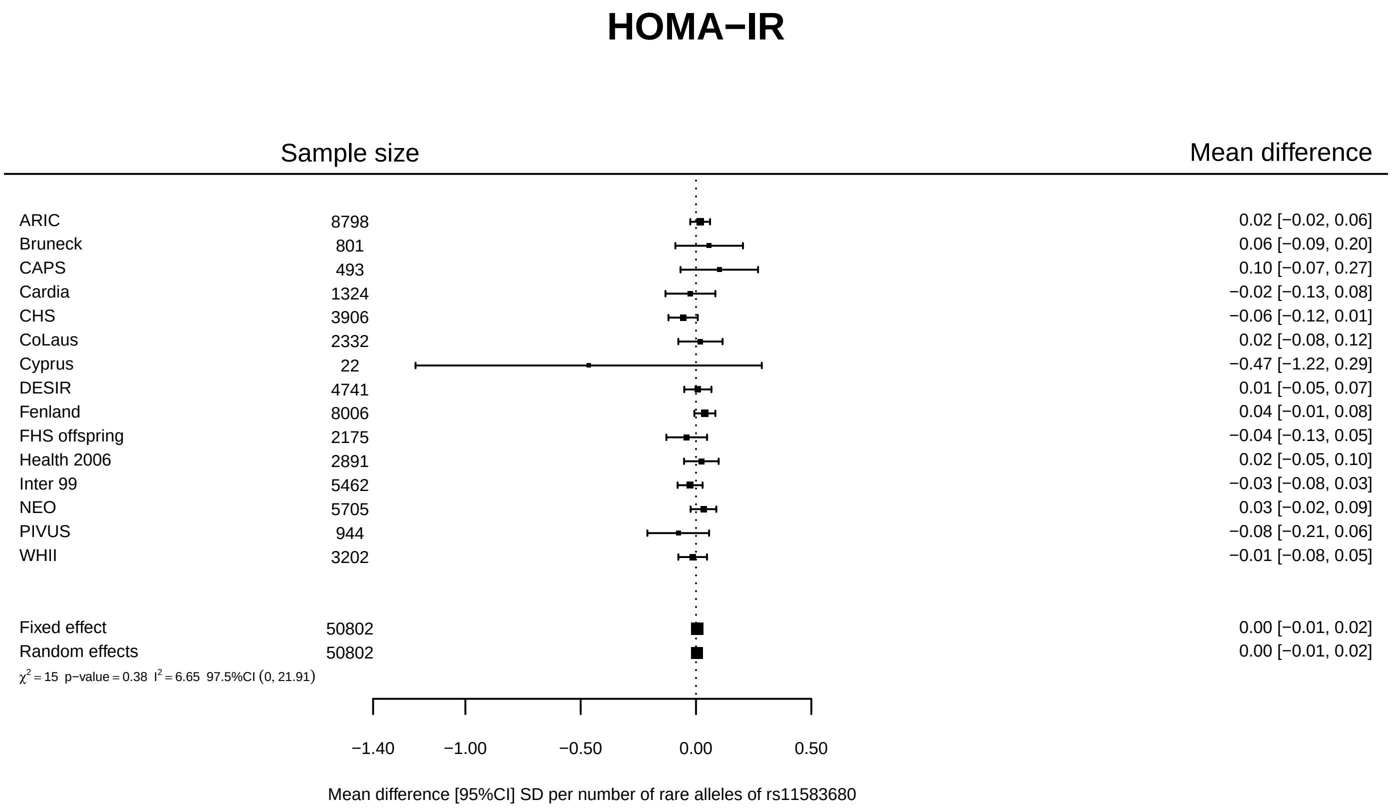

Figure 88: Mean difference of PCSK9 SNP rs11583680 on $HOMA\check{\ }IR$ (SD) per number of rare alleles, with 95% confidence interval (CI). Results are pooled using a fixed and random effects model, irrespective of observed between study heterogeneity; measured as a two sided Q–test (χ^2) and the I^2 with one–sided 97.5% CI. Note the outcome was standardized based on the SD of the natural logarithm.

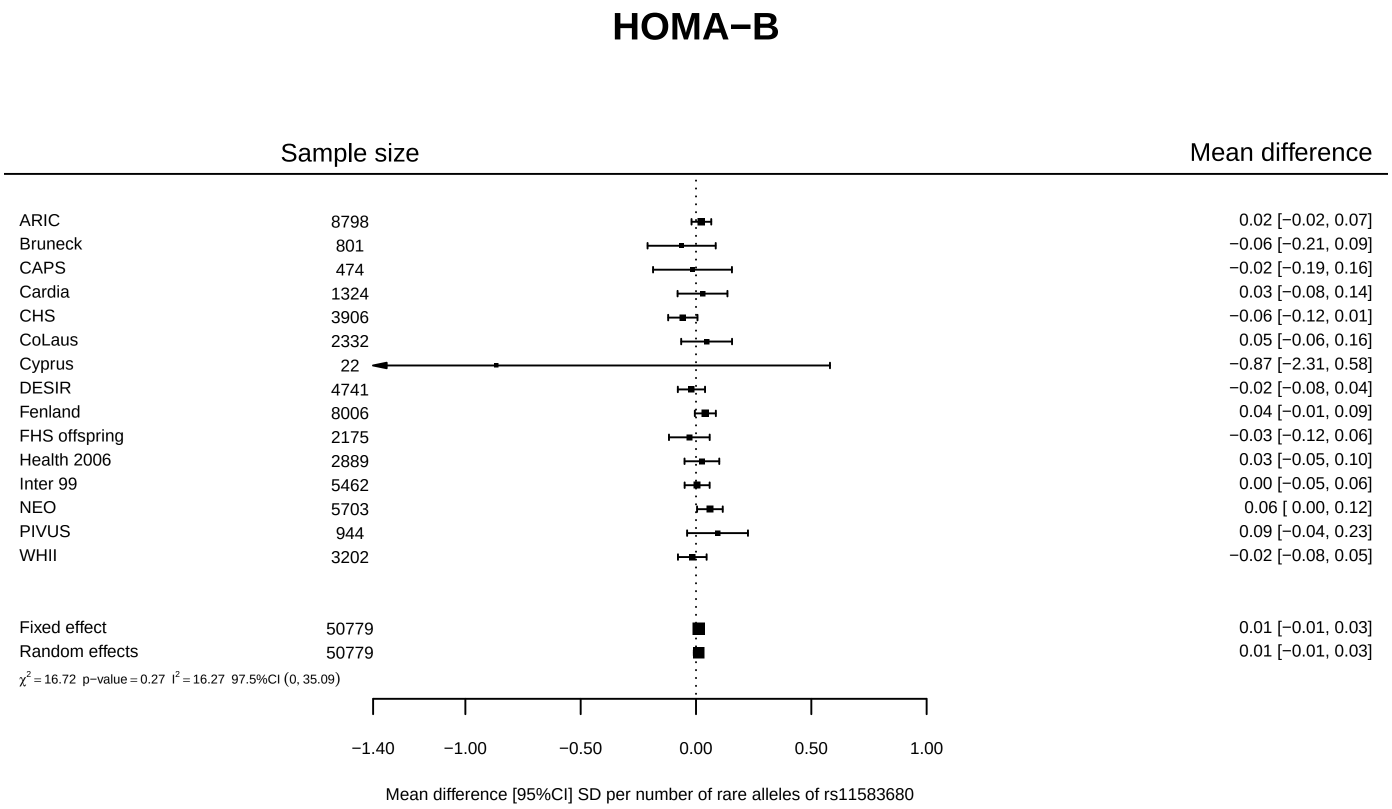

Figure 89: Mean difference of PCSK9 SNP rs11583680 on $HOMA\check{}\beta$ (SD) per number of rare alleles, with 95% confidence interval (CI). Results are pooled using a fixed and random effects model, irrespective of observed between study heterogeneity; measured as a two sided Q–test (χ^2) and the I^2 with one–sided 97.5% CI. Note the outcome was standardized based on the SD of the natural logarithm.

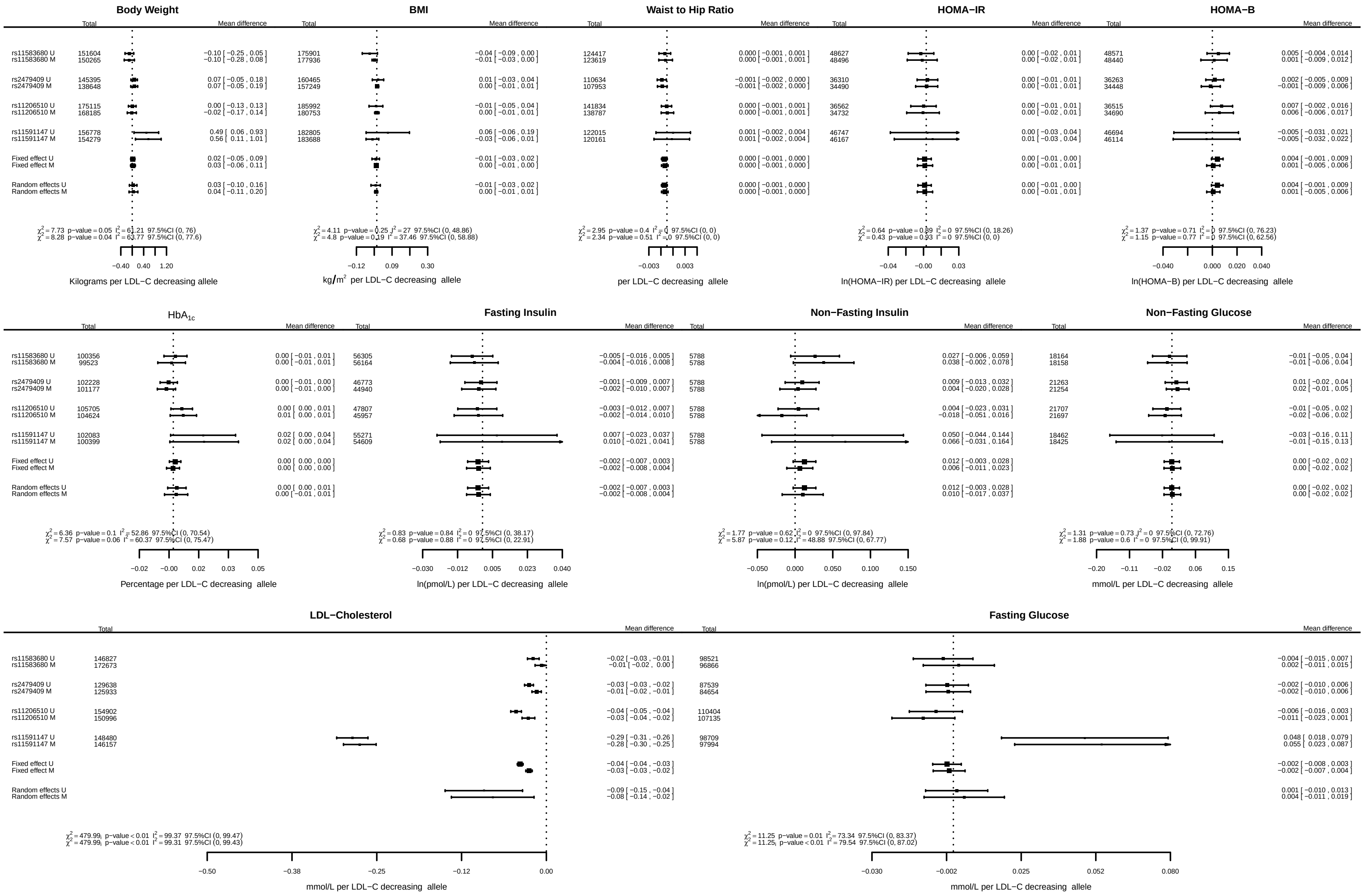

Figure 90: Multivariable (M) association accounting for between SNP correlation, compared to univariable (U) associations assuming no between SNP correlation. Effect estimates in mean difference per LDL–C decreasing allele, with 95% confidence interval (CI). Results are pooled using a fixed and random effects model, irrespective of observed between study heterogeneity; measured as a two sided Q–test (χ^2) and the I^2 with one–sided 97.5% CI. Note that similar multivariable repository data were unavailable, and to increase comparability this data was also excluded from the univariable associations.

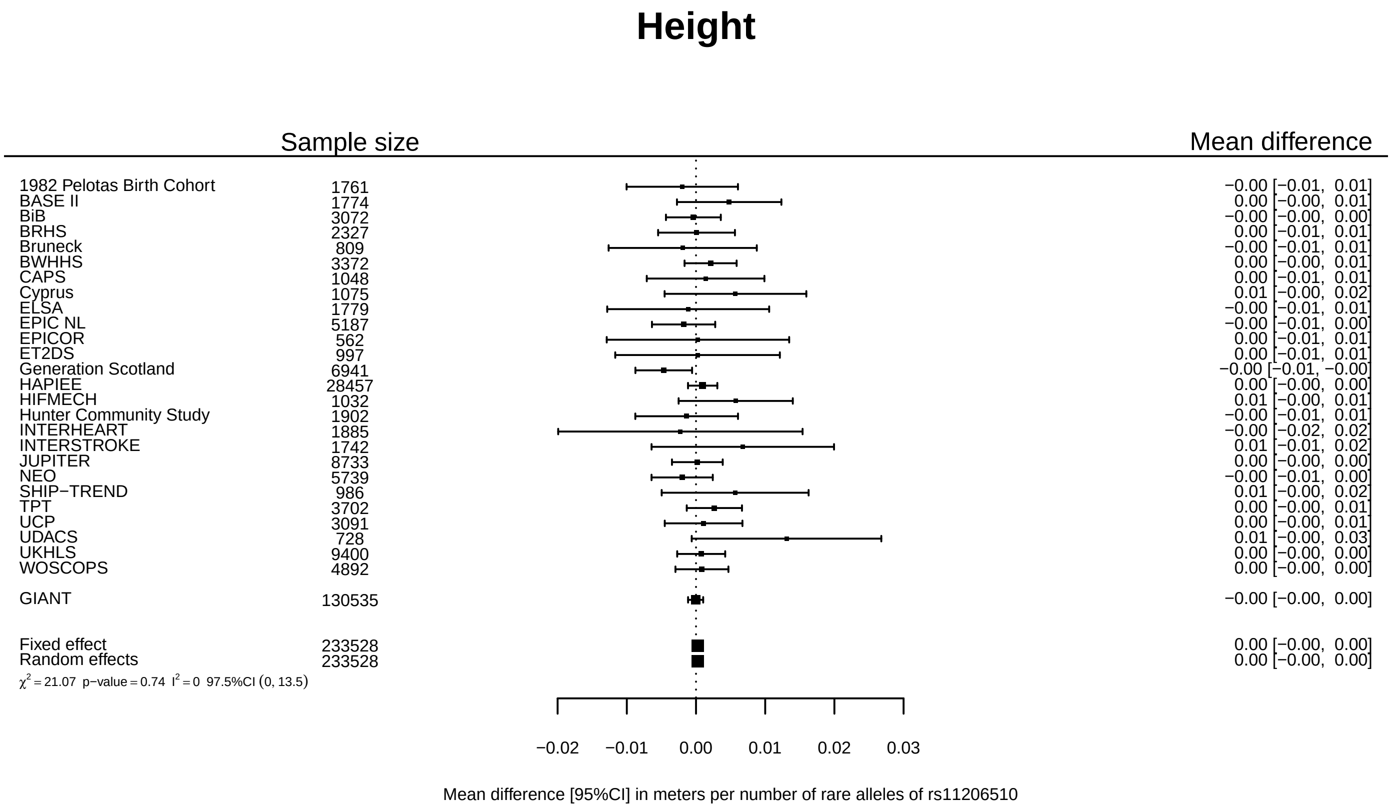

Figure 91: Mean difference of PCSK9 SNP rs11206510 on height (meter) per number of rare alleles, with 95% confidence interval (CI). Results are pooled using a fixed and random effects model, irrespective of observed between study heterogeneity; measured as a two sided Q–test (χ^2) and the I^2 with one–sided 97.5% CI.

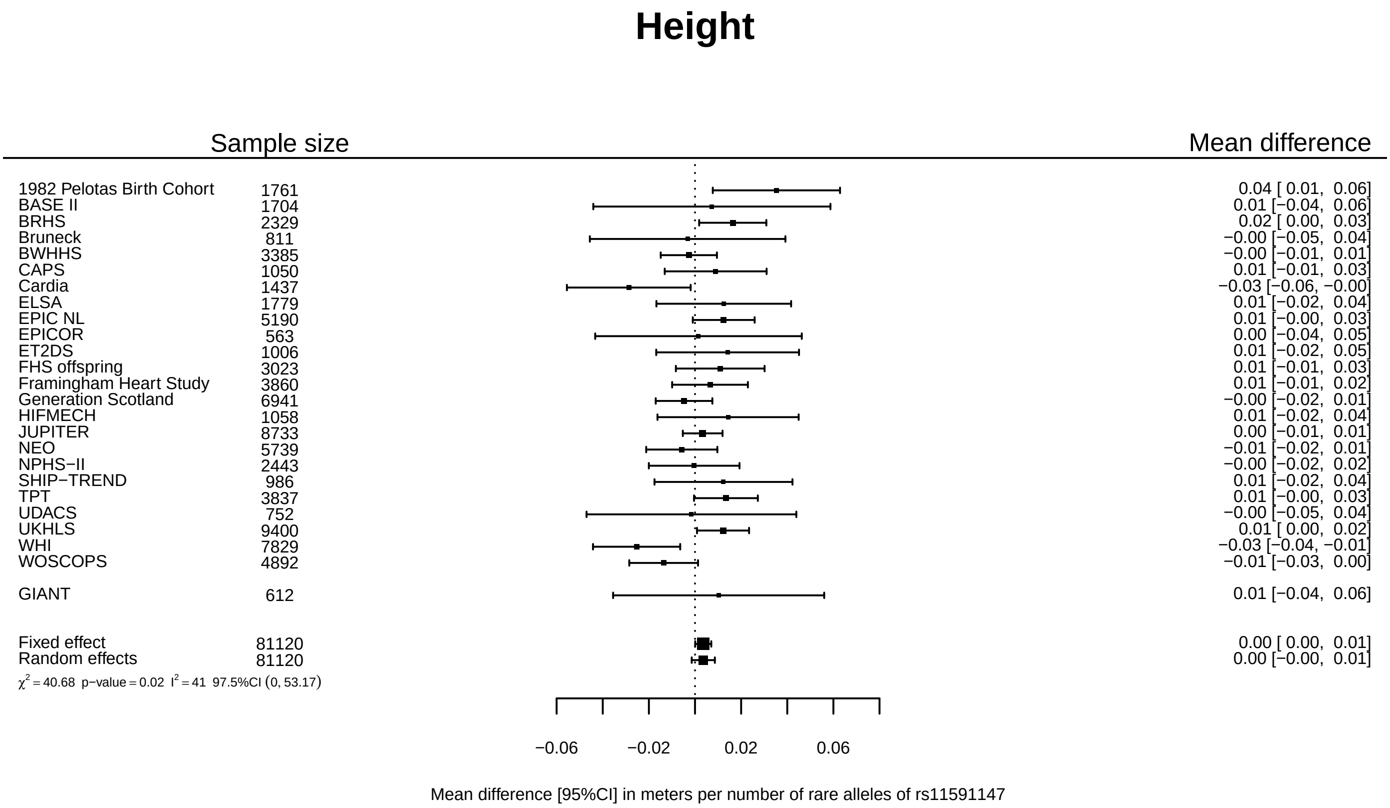

Figure 92: Mean difference of PCSK9 SNP rs11591147 on height (meter) per number of rare alleles, with 95% confidence interval (CI). Results are pooled using a fixed and random effects model, irrespective of observed between study heterogeneity; measured as a two sided Q–test (χ^2) and the I^2 with one–sided 97.5% CI.

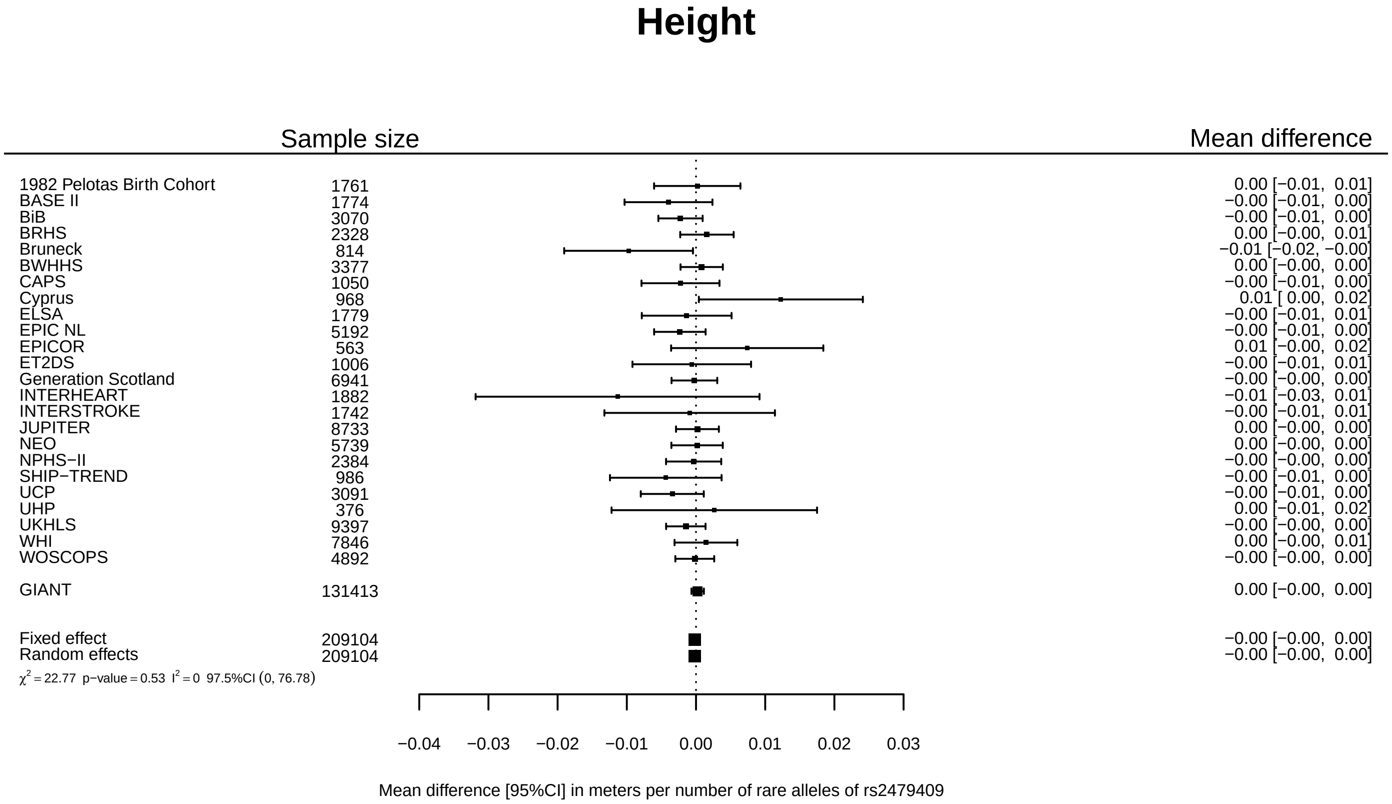

Figure 93: Mean difference of PCSK9 SNP rs2479409 on height (meter) per number of rare alleles, with 95% confidence interval (CI). Results are pooled using a fixed and random effects model, irrespective of observed between study heterogeneity; measured as a two sided Q–test (χ^2) and the I^2 with one–sided 97.5% CI.

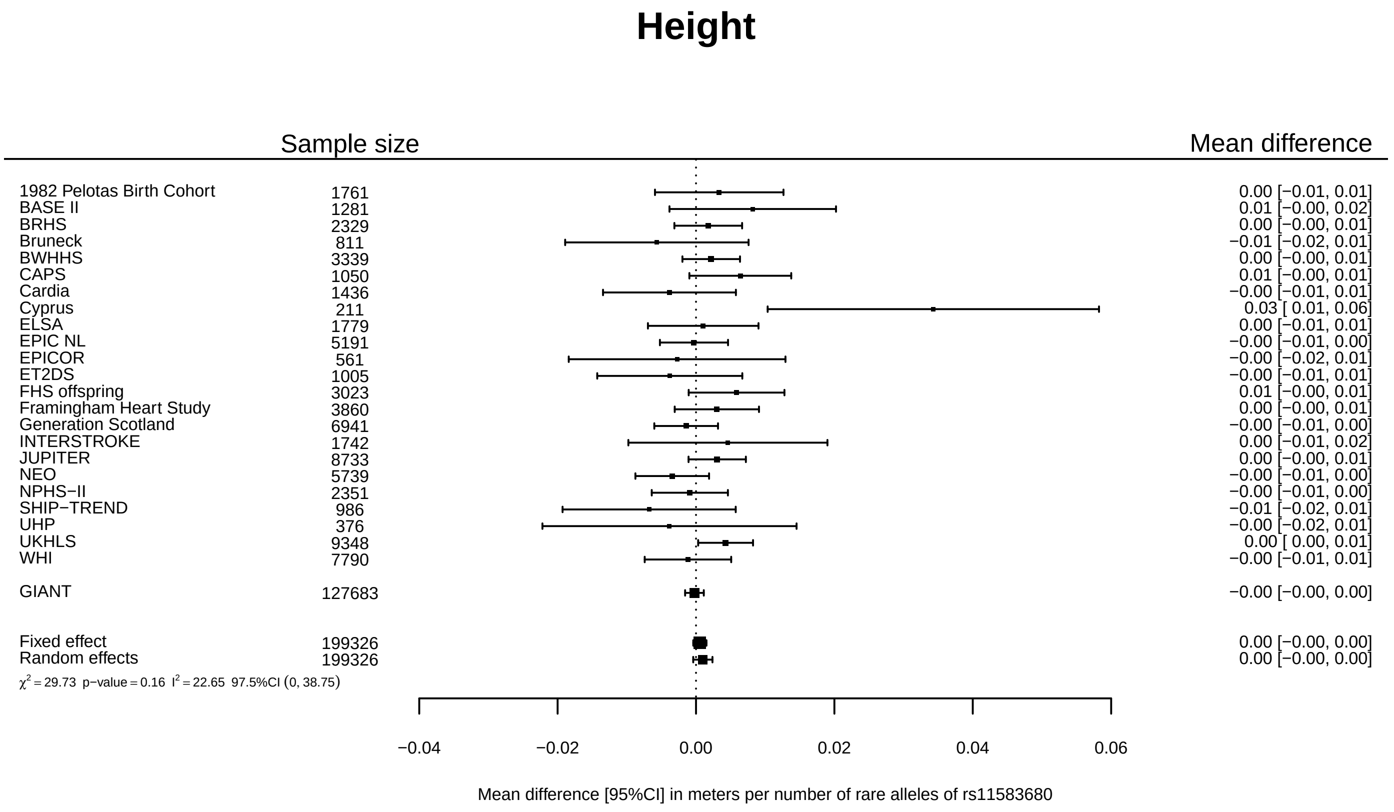

Figure 94: Mean difference of PCSK9 SNP rs11583680 on height (meter) per number of rare alleles, with 95% confidence interval (CI). Results are pooled using a fixed and random effects model, irrespective of observed between study heterogeneity; measured as a two sided Q–test (χ^2) and the I^2 with one–sided 97.5% CI.

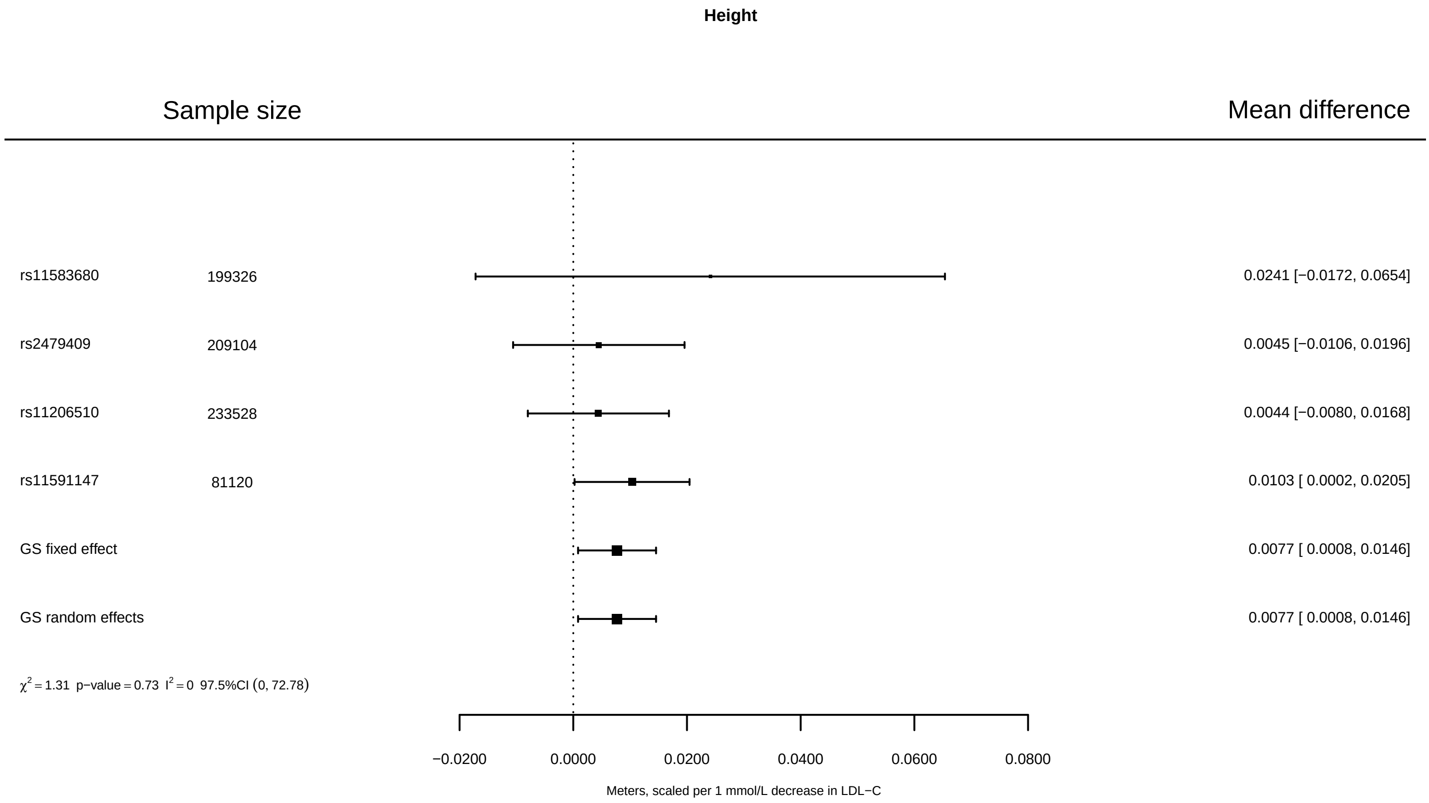

Figure 95: Mean difference of the 4 PCSK9 SNP on height (meter) scaled to have a 1 mmol/L decrease in LDL–C, with 95% confidence interval (CI). Results are pooled using a fixed and random effects genetic score, irrespective of observed between study heterogeneity; measured as a two sided Q–test (χ^2) and the I^2 with one–sided 97.5% CI.

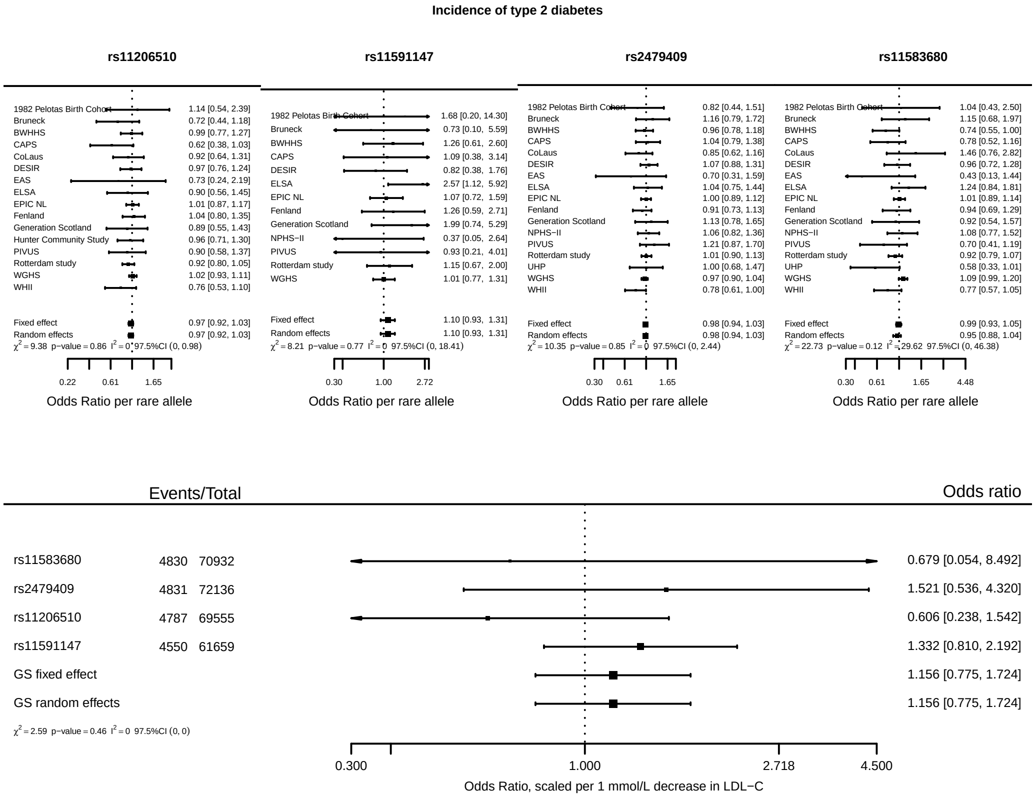

Figure 96: Associations of PCSK9 with T2DM incidence as odds ratio's (OR) with 95% confidence interval (CI). Results are pooled using a fixed and random effects model, irrespective of observed between study heterogeneity; measured as a two sided Q–test (χ^2) and the I^2 with one–sided 97.5% CI.

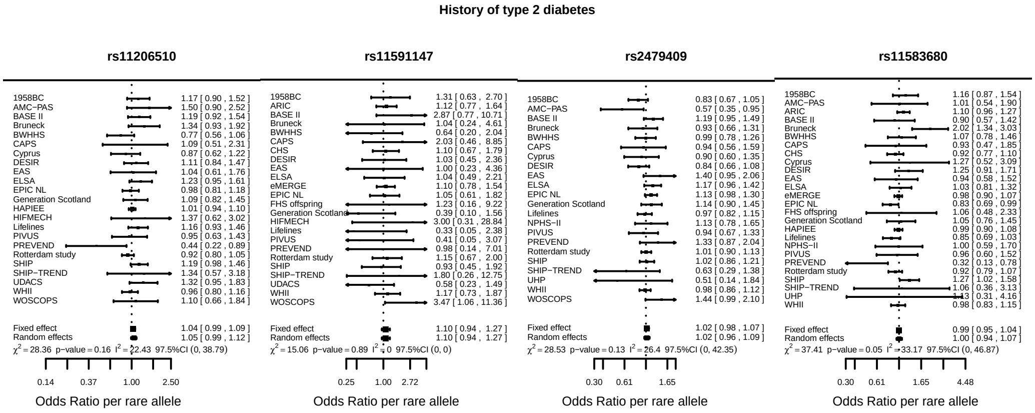

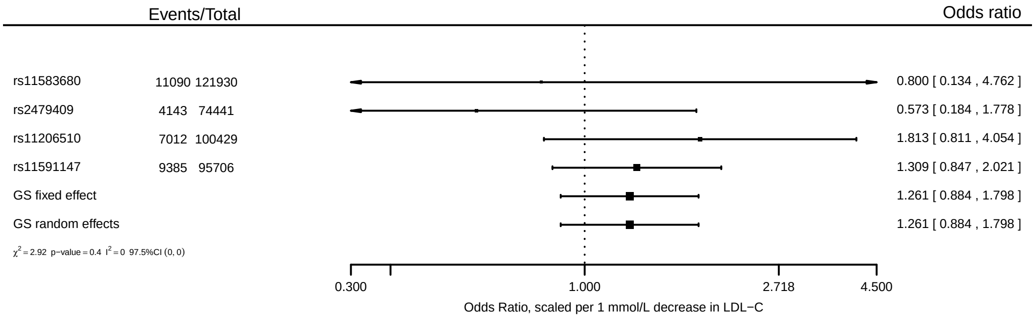

Figure 97: Associations of PCSK9 with T2DM history as odds ratio's (OR) with 95% confidence interval (CI). Results are pooled using a fixed and random effects model, irrespective of observed between study heterogeneity; measured as a two sided Q–test (χ^2) and the I^2 with one–sided 97.5% CI.

Appendix Tables

Appendix Table 1. SNP Selection

SNP	MAF	LDL-C (mmol/L) mean difference	CAD odds ratio	CADD score#	Functionality
rs11583680	0.14	-0.05 ($9*10^{9}$)	0.98 (0.48)	8.94	Non-synonymous
rs11591147	0.01	-0.50 ($8*10^{-145}$)	0.70 ($4*10^{-4}$)**	11.50	Non-synonymous
rs2479409	0.33	0.06 ($2*10^{-53}$)	1.04 (0.011)	4.94	Upstream
rs11206510	0.15	-0.08 ($2*10^{-55}$)	0.92 ($4*10^{-4}$)	NA	Intergenic

* all esimates are per minor allele, p-values are reported between brackets, the reported associations were extracted from the GLGC and CARDIoGRAMplusC4D consortia. ** based on Benn et.al., 2010 (30).

# see http://cadd.gs.washington.edu/ for an explanation on CADD scores

Appedix Table 2. Genetics

Study	rs11206510 HWE p- value	rs11206510 HWE alpha	rs11206510 Common	rs11206510 Het	rs11206510 Rare	rs11206510 MAF	rs11591147 HWE p-value	rs11591147 HWE alpha	rs11591147 Common	rs11591147 Het	rs11591147 Rare	rs11591147 MAF	rs2479409 HWE p-value	rs2479409 HWE alpha	rs2479409 Common	rs2479409 Het	rs2479409 Rare	rs2479409 MAF	rs11583680 HWE p-value	rs11583680 HWE alpha	rs11583680 Common	rs11583680 Het	rs11583680 Rare	rs11583680 MAF	Genotyping call rate	Imputation Quality
1958BC	9.015E-01	2.778E-03	3688	1679	189	0.185	1.855E-01	3.968E-04	5365	194	0	0.02	9.917E-01	1.667E-02	2326	2540	693	0.35	1.002E-01	3.546E-04	4056	1305	125	0.14	rs11583680, rs11591147, rs2479409, rs11206510: 0.9868705, 1, 1, 0.9994604	
1982 Pelotas Birth Cohort	4.982E-01	6.667E-04	1216	532	52	0.177	7.463E-01	1.250E-03	1615	26	0	0.01	3.851E-01	5.102E-04	718	824	258	0.37	3.553E-01	4.902E-04	1321	330	16	0.11	rs2479409 (Call rate=99.9%); rs11206510 (Call rate>99.9%)	rs11583680 (Imputed - INFO metric=0.97); rs11591147 (Imputed - INFO metric=0.843);
AMC-PAS	4.853E-01	6.329E-04	440	175	21	0.171	8.091E-01	1.429E-03	617	12	0	0.01	2.650E-01	4.425E-04	269	278	87	0.36	9.747E-01	8.333E-03	470	151	12	0.14	call rate: rs11583680=0.9946, rs11591147=0.9878, rs2479409=0.9973 ,rs11206510=1.0000	
ARIC	NA	NA	0	0	0	NA	7.510E-01	1.316E-03	9281	304	3	0.02	NA	NA	0	0	0	NA	4.842E-02	3.401E-04	6948	2200	205	0.14	call rates > 0.99	
BASE II	5.145E-01	7.143E-04	1178	551	71	0.193	8.849E-01	2.174E-03	1718	12	0	0.00	7.165E-01	1.136E-03	776	817	207	0.34	8.866E-01	2.273E-03	1065	221	12	0.09	call rates > 0.98	
BiB	9.400E-01	4.545E-03	2056	951	109	0.188	NA	NA	0	0	0	NA	2.471E-01	4.310E-04	1300	1401	413	0.36	NA	NA	0	0	0	NA	call rates > 0.99	
BRHS	8.746E-01	2.000E-03	1754	544	41	0.134	3.944E-01	5.208E-04	2261	81	0	0.02	1.746E-03	3.165E-04	1053	983	305	0.34	5.039E-01	7.042E-04	1552	715	75	0.18	call rates > 0.99	
Bruneck	3.087E-01	4.673E-04	515	255	39	0.206	7.493E-01	1.282E-03	793	18	0	0.01	4.902E-01	6.410E-04	338	380	96	0.35	9.885E-01	1.250E-02	619	179	13	0.13	rs2479409 (0.99), rs11206510 (0.98), rs11583680 (0.99), rs11591147 (0.99)	
BWHHS	4.135E-01	5.319E-04	2234	1053	113	0.188	7.511E-01	1.351E-03	3314	98	1	0.01	4.726E-01	6.024E-04	1485	1513	407	0.34	5.428E-01	7.353E-04	2465	825	75	0.14	call rates > 0.99	
CAPS	9.048E-01	2.941E-03	822	226	15	0.120	5.748E-01	7.937E-04	1029	36	0	0.02	1.040E-02	3.226E-04	488	438	139	0.34	9.823E-01	1.000E-02	732	302	31	0.17	call rates > 0.99	
Cardia	NA	NA	0	0	0	NA	5.206E-01	7.246E-04	1395	48	0	0.02	NA	NA	0	0	0	NA	6.065E-01	8.621E-04	1045	368	29	0.15	call rates > 0.99	
CHS	NA	NA	0	0	0	NA	2.466E-01	4.274E-04	3834	116	2	0.02	NA	NA	0	0	0	NA	1.044E-01	3.571E-04	2951	914	87	0.14	call rates > 0.99	
CoLaus	2.584E-01	4.386E-04	2648	1404	206	0.213	NA	NA	0	0	0	NA	2.867E-01	4.587E-04	1847	1949	478	0.34	1.692E-03	3.145E-04	2264	354	2	0.07	call rates > 0.99	rs11583680 r-squared: 0.4
Cyprus	NA	NA	NA	NA	NA	NA	NA	NA	NA	NA	NA	NA	NA	NA	NA	NA	NA	NA	NA	NA	NA	NA	NA	NA	call rate 99%	
DESIR	2.130E-01	4.065E-04	3033	1573	182	0.202	5.487E-01	7.463E-04	4607	183	1	0.02	6.302E-01	9.259E-04	1981	2185	621	0.36	2.749E-01	4.505E-04	3519	1151	82	0.14	call rates ≥95%	
EAS	5.223E-03	3.185E-04	525	224	9	0.160	6.457E-01	9.804E-04	739	25	0	0.02	3.776E-01	5.051E-04	350	342	72	0.32	5.825E-01	8.333E-04	500	239	25	0.19	call rates > 0.99	
ELSA	6.314E-01	9.434E-04	1362	482	39	0.149	4.531E-01	5.814E-04	1819	64	0	0.02	1.798E-02	3.289E-04	828	806	249	0.35	8.765E-01	2.083E-03	1224	587	72	0.19	call rates > 0.99	
eMERGE	NA	NA	0	0	0	NA	4.528E-01	5.747E-04	8942	142	0	0.01	NA	NA	0	0	0	NA	4.437E-01	5.618E-04	6847	2187	163	0.14		
EPIC NL	8.563E-01	1.724E-03	3805	1275	109	0.144	9.245E-01	3.571E-03	5042	149	1	0.01	6.578E-01	1.000E-03	2308	2298	588	0.33	3.583E-01	5.000E-04	3375	1610	208	0.20	call rates 0.999615	
EPICOR	7.539E-01	1.389E-03	358	185	22	0.203	7.330E-01	1.220E-03	550	16	0	0.01	9.361E-01	4.167E-03	241	256	69	0.35	8.367E-01	1.515E-03	428	126	10	0.13	call rate: rs11583680=0.9964, rs11591147=1, rs2479409=1 ,rs11206510=0.9982	
ET2DS	8.147E-01	1.471E-03	748	233	17	0.134	5.746E-01	7.813E-04	972	35	0	0.02	8.701E-01	1.923E-03	454	446	107	0.33	3.395E-01	4.762E-04	663	313	30	0.19	call rates > 0.99	
Fenland	1.936E-01	4.000E-04	6322	3000	326	0.189	1.306E-01	3.623E-04	9325	292	0	0.02	1.386E-01	3.759E-04	4148	4302	1192	0.35	9.679E-01	7.143E-03	7073	2197	170	0.13	rs11206510 99.96%; rs2479409 99.88%; rs11591147 99.96%; rs11583680 99.87%	all genotyped
FHS offspring	NA	NA	0	0	0	NA	3.551E-01	4.854E-04	2923	100	0	0.02	NA	NA	0	0	0	NA	1.382E-01	3.731E-04	2246	708	69	0.14	call rates > 0.99	
Framingham Heart Study	NA	NA	0	0	0	NA	3.503E-02	3.356E-04	3743	116	3	0.02	NA	NA	0	0	0	NA	9.215E-01	3.125E-03	2891	900	71	0.13	call rates > 0.99	
Generation Scotland	4.247E-01	0.000537634	4717	2017	230	0.177843194	1.746E-02	3.268E-04	6743	216	5	0.02	9.240E-01	3.333E-03	851	3172	2941	0.65	1.744E-01	3.846E-04	5162	1683	119	0.14	call rate > 98%	
HAPIEE	8.394E-01	1.613E-03	20398	7450	674	0.154	NA	NA	0	0	0	NA	NA	NA	0	0	0	NA	4.787E-10	3.106E-04	22450	5531	475	0.11		
Health 2006	6.256E-01	9.091E-04	1909	882	108	0.189	4.572E-01	5.882E-04	2822	79	0	0.01	9.593E-01	5.556E-03	1238	1310	348	0.35	1.045E-01	3.597E-04	2122	722	47	0.14	call rate: rs11583680=0.9949, rs11591147=1.0000, rs2479409=0.9998 ,rs11206510=0.9999	
HIFMECH	4.383E-01	5.495E-04	692	304	39	0.185	7.094E-01	1.064E-03	1037	24	0	0.01	NA	NA	0	0	0	NA	NA	NA	0	0	0	NA	call rate rs11583680: 96%, rs11206510: 94%	
Hunter Community Study	8.290E-02	0.00034965	1290	540	73	0.180241724	NA	NA	0	0	0	NA	NA	NA	0	0	0	NA	NA	NA	0	0	0	NA		r-squared = 0.89.
Inter 99	9.919E-01	2.500E-02	3979	1829	210	0.187	3.309E-01	4.717E-04	5871	149	0	0.01	3.192E-02	3.333E-04	2629	2624	740	0.34	7.189E-01	1.163E-03	4421	1423	119	0.14	call rate: rs11583680=0.9949, rs11591147=1.0000, rs2479409=0.9998 ,rs11206510=0.9999	
INTERHEART	4.946E-01	6.494E-04	1276	555	54	0.176	NA	NA	0	0	0	NA	4.258E-01	5.435E-04	800	866	216	0.34	NA	NA	0	0	0	NA	rs11583680, rs11591147, rs2479409, rs11206510: NA,NA,0.9995,0.9979	
INTERSTROKE	7.973E-02	3.472E-04	1174	525	43	0.175	NA	NA	0	0	0	NA	3.538E-01	4.808E-04	747	774	221	0.35	2.519E-01	4.348E-04	1323	384	35	0.13	rs2479409 (genotyped;r2=1;callrate=1),rs11206510 (genotyped;r2=1;callrate=1)	rs11583680 (imputed; r2=0.996; callrate=1)
JUPITER	7.153E-01	1.111E-03	5604	2803	342	0.199	9.015E-01	2.632E-03	8239	502	8	0.03	7.323E-01	1.190E-03	3894	3875	980	0.33	4.096E-01	5.263E-04	6414	2143	192	0.14		
Lifelines	1.297E-02	0.000324675	10387	2851	157	0.118141097	6.086E-01	8.772E-04	13277	118	0	0.00	1.379E-01	3.704E-04	6209	5876	1310	0.32	8.911E-01	2.381E-03	8350	4455	590	0.21	call rates > 0.99	
NEO	4.834E-01	6.250E-04	3677	1844	218	0.199	9.945E-01	5.000E-02	5589	149	1	0.01	9.430E-01	5.000E-03	2503	2576	660	0.34	1.534E-01	3.817E-04	1934	289	16	0.07	call rates > 0.99	rs11583680 imputed, R2=0.866
NPHS-II	NA	NA	0	0	0	NA	6.899E-02	3.448E-04	2401	42	1	0.01	2.824E-01	4.545E-04	1044	1050	291	0.34	5.600E-01	7.692E-04	1740	562	50	0.14	call rate rs11583680: 98%, rs2479409: 99% rs11591147: 96%	
PIVUS	1.846E-01	0.000393701	650	262	35	0.175290391	6.806E-01	1.020E-03	922	25	0	0.01	4.828E-01	6.173E-04	419	429	99	0.33	5.032E-01	6.757E-04	715	213	19	0.13	call rates 1,00	
PREVEND	2.369E-01	0.000423729	1046	139	2	0.060235889	9.266E-01	3.846E-03	2384	9	0	0.00	8.338E-04	3.125E-04	134	198	33	0.36	1.318E-01	3.650E-04	507	68	0	0.06	call rates 1,00	rs11206510 (Imputed INFO=0.822) rs2479409 (Imputed INFO=0.784) rs11591147 (Imputed INFO=0.571) rs11583680 (Imputed INFO=0.668)
Rotterdam study	1.940E-01	4.032E-04	2358	1048	134	0.186	5.756E-01	8.065E-04	3474	66	0	0.01	6.898E-01	1.042E-03	1605	1565	370	0.33	2.324E-02	3.311E-04	2627	826	87	0.14	call rates > 0.99, expect rs2479409 = 0.95.	
SHIP	4.975E-01	6.579E-04	2678	1255	137	0.188	3.866E-01	5.155E-04	3961	109	0	0.01	5.763E-01	8.197E-04	1757	1822	491	0.34	8.631E-01	1.852E-03	3017	975	77	0.14	rs11206510 (1.00), rs2479409 (1.00)	r2 = 1.00 for rs11591147 and rs2479409, r2=0.67 for rs11591147, r2=0.78 for rs11583680
SHIP-TREND	3.565E-01	4.950E-04	667	293	26	0.175	1.840E-01	3.906E-04	953	32	1	0.02	3.666E-02	3.378E-04	431	419	136	0.35	2.246E-01	4.167E-04	761	215	10	0.12	rs11206510 (1.00), rs2479409 (1.00), rs11591147 (1.00), rs11583680 (1.00)	r2 = 1.00 for rs11591147, rs2479409, rs11206510 and rs11583680
TPT	5.038E-01	6.944E-04	2495	1081	126	0.180	4.424E-01	5.556E-04	3743	94	0	0.01	NA	NA	0	0	0	NA	NA	NA	0	0	0	NA	call rate rs11583680: 96%, rs11206510: 90%	
UCP	8.970E-01	2.500E-03	2096	972	111	0.188	6.448E-01	9.615E-04	3089	88	1	0.01	2.185E-01	4.132E-04	1352	1418	409	0.35	NA	NA	0	0	0	NA	call rate: rs11206510 = 0.9997; rs11591147 = 0.9991; rs2479409 = 0.9997	
UDACS	5.037E-01	6.849E-04	485	225	22	0.184	7.130E-01	1.087E-03	739	20	0	0.01	NA	NA	0	0	0	NA	NA	NA	0	0	0	NA	call rate: rs11591147=0.98 ,rs11206510=0.95	
UHP	NA	NA	0	0	0	NA	NA	NA	0	0	0	NA	9.628E-01	6.250E-03	831	884	234	0.35	9.825E-02	3.521E-04	1250	636	63	0.20	call rates > 99%	
UKB	7.101E-01	NA	74019	34199	3979	0.188	7.993E-01	NA	108212	3951	34	0.02	7.776E-01	NA	47526	50960	13711	0.35	9.103E-01	NA	83292	26762	2143	0.14	call rates 100%	
UKHLS	1.433E-01	3.788E-04	6364	2822	345	0.184	4.594E-01	5.952E-04	9253	277	1	0.01	6.022E-01	8.475E-04	4017	4357	1154	0.35	6.204E-01	8.929E-04	7060	2246	171	0.14	call rates ≥ 99%	
WGHS	1.379E-01	3.676E-04	15013	6787	818	0.186	1.777E-01	3.876E-04	22520	764	10	0.02	4.473E-01	5.682E-04	9646	10209	2762	0.35	8.377E-01	1.563E-03	17466	5405	423	0.13		
WHII	3.054E-01	4.630E-04	3771	1193	83	0.135	4.821E-01	6.098E-04	4910	154	2	0.02	8.103E-03	3.205E-04	2282	2157	602	0.33	2.164E-01	4.098E-04	3357	1502	188	0.19	call rates > 0.99	
Women's Health Initiative (WHI)	NA	NA	0	0	0	NA	2.313E-01	4.202E-04	7620	209	0	0.01	2.686E-01	4.464E-04	3283	3619	944	0.35	8.566E-01	1.786E-03	5847	1806	137	0.13		
WOSCOPS	5.316E-02	3.425E-04	3504	1293	95	0.152	5.544E-01	7.576E-04	4810	82	0	0.01	8.534E-01	1.667E-03	1963	2267	662	0.37	NA	NA	0	0	0	NA	call rates 100%	

Footnote: The Hardy-Weinberg goodness of fit tests are corrected for multiple testing using a bonferroni holm procedure to preserve a family-wise error rate (FEWER) of 0.05 or less; the corrected alpha's are presented in the "HWE alpha" columns.

Appendix Table 3. Baseline data

Study	Number of subjects	Mean Age	SD Age	Number of subjects with an observed age	Proportion Male	Number of subjects with gender observed	Mean LDL-C	SD LDL-C	Number of subjects with an observed LDL-C	Mean Fasting glucose	SD Fasting glucose	Number of subjects with an observed glucose	Mean HbA1c	SD HbA1c	Number of subjects with an observed HbA1c	Proportion History of T2D	Number of subjects with T2D history observed	Proportion Incidence of T2D	Number of subjects with T2D incidence observed
1958BC	5559	NA	NA	0	0.45	5559	5144	0.90	5144	NA	NA	0	5.26	0.68	5461	0.03	5505	NA	NA
1982 Pelotas Birth Cohort	1859	30.18	0.34	1859	0.47	1859	1777	0.73	1777	NA	NA	0	5.09	0.47	1785	NA	NA	0.01	1859
AMC-PAS	636	43.17	5.22	636	0.76	636	376	1.22	376	5.59	1.53	635	5.60	0.80	384	0.07	636	NA	NA
ARIC	9588	54.28	5.69	9588	0.46	9588	9414	0.98	9414	6.05	1.77	8990	NA	NA	0	0.09	9570	NA	NA
BASE II	2170	59.10	17.15	2170	0.48	2170	2115	0.95	2115	5.17	1.05	2098	5.48	0.58	2083	0.10	2118	NA	NA
BiB	3117	26.72	6.06	3117	0.00	3117	3061	1.00	3061	4.41	0.43	3060	NA	NA	0	NA	0	NA	NA
BRHS	2342	68.91	5.62	2342	1.00	2342	2277	1.00	2277	5.56	1.31	2330	NA	NA	0	NA	NA	NA	NA
Bruneck	816	62.65	11.11	816	0.49	816	816	0.99	816	5.66	1.32	816	5.50	0.72	816	0.11	816	0.07	816
BWHHS	3413	68.86	5.51	3413	0.00	3413	3292	1.09	3292	6.06	1.64	3358	5.01	0.87	3292	0.05	3413	0.07	3231
CAPS	1065	56.77	4.46	1065	1.00	1065	1006	0.90	1006	5.38	1.30	1032	NA	NA	0	0.03	1065	0.10	1065
Cardia	1443	25.58	3.37	1443	0.47	1443	1436	0.76	1436	4.62	0.74	1331	NA	NA	0	NA	NA	NA	NA
CHS	3952	72.77	5.60	3952	0.44	3952	3884	0.92	3884	6.09	1.83	3938	NA	NA	0	0.15	3938	NA	NA
CoLaus	4274	53.40	10.70	4274	0.47	4274	4216	0.91	4216	5.55	1.17	4273	NA	NA	0	NA	NA	0.02	3992
Cyprus	1102	59.28	10.80	1102	0.48	1102	1051	0.85	1051	5.61	1.64	1055	NA	NA	0	0.11	1102	NA	0
DESIR	4791	46.64	10.07	764	0.49	4791	4731	0.91	4731	5.36	0.83	4785	5.47	0.52	4782	0.03	4695	0.05	4157
EAS	764	64.51	5.64	1883	0.48	764	760	1.24	760	5.78	1.38	763	NA	NA	0	0.08	764	0.02	764
ELSA	1883	73.69	9.44	9266	0.53	1883	1835	1.06	1835	5.07	0.89	856	5.68	0.76	1871	0.11	1883	0.04	1883
eMERGE	9266	37.90	14.40	5194	0.49	9266	9266	0.77	9266	NA	NA	0	NA	NA	0	0.37	9266	NA	NA
EPIC NL	5194	54.06	10.11	566	0.22	5194	4267	0.90	4267	NA	NA	0	5.91	1.24	4342	0.08	5194	0.27	2575
EPICOR	566	52.93	7.38	1007	0.64	566	520	1.05	520	5.64	1.56	415	NA	NA	0	0.00	566	NA	NA
ET2DS	1007	67.89	4.23	9653	0.51	1007	0	NA	0	7.55	2.11	995	7.39	1.13	974	1.00	1007	0.00	1007
Fenland	9653	48.67	7.36	3862	0.53	9653	9562	0.91	9562	4.84	0.68	9622	5.54	0.48	6453	NA	NA	0.02	9653
FHS offspring	3283	34.83	9.73	3283	0.46	3283	2527	0.83	2527	5.16	1.04	2700	NA	NA	0	0.01	3148	NA	NA
Framingham Heart Study	3862	40.14	8.81	34876	0.53	3862	3803	0.82	3803	5.28	1.01	3822	NA	NA	0	NA	NA	NA	NA
Generation Scotland	6964	57.12	10.69	2901	0.42	6964	0	NA	0	4.90	1.00	5772	NA	NA	0	0.02	6964	0.01	6964
HAPIEE	34876	58.61	7.28	1106	0.47	34876	31160	1.04	31160	5.73	2.21	23872	5.25	0.75	3069	0.08	34876	NA	NA
Health 2006	2901	49.57	12.89	6022	0.45	2901	2881	0.92	2881	5.25	0.96	2901	NA	NA	0	NA	NA	0.04	2901
HIFMECH	1106	51.67	5.44	1886	1.00	1106	0	NA	0	NA	NA	0	NA	NA	0	0.05	1096	NA	NA
Hunter Community Study	3017	66.26	7.80	1742	0.47	3017	2094	0.90	2094	5.14	1.22	2083	NA	NA	0	NA	NA	0.09	2506
Inter 99	6022	46.23	7.91	8749	0.49	6022	5947	0.96	5947	5.61	1.11	6017	NA	NA	0	NA	NA	0.02	6022
INTERHEART	1886	60.45	11.90	4791	0.71	1886	1883	1.07	1883	NA	NA	0	5.92	0.80	1503	NA	NA	NA	NA
INTERSTROKE	1742	66.87	12.52	5739	0.55	1742	1700	1.00	1700	NA	NA	0	NA	NA	0	NA	NA	NA	NA
JUPITER	8749	66.10	7.77	3012	0.68	8749	8746	0.45	8746	5.30	0.60	8729	5.63	0.37	8733	NA	NA	NA	NA
Lifelines	13395	48.13	11.44	4070	0.42	13395	13386	0.90	13386	5.09	0.87	13049	5.57	0.46	13335	0.03	13338	NA	NA
NEO	5739	55.97	5.95	986	0.48	5739	5721	0.98	5721	5.72	1.10	5715	5.45	0.53	5698	NA	NA	NA	NA
NPHS-II	3012	56.07	3.49	5085	1.00	3012	2508	1.01	2508	NA	NA	0	NA	NA	0	0.02	3012	0.06	2937
PIVUS	1014	70.15	0.15	3179	0.50	1014	1009	0.88	1009	6.03	1.82	1011	NA	NA	0	0.09	1014	0.09	1014
PREVEND	3649	49.62	12.49	790	0.52	3649	0	NA	0	NA	NA	0	NA	NA	0	0.02	3649	NA	NA
Rotterdam study	3540	66.09	7.23	9531	0.59	3540	0	NA	0	NA	NA	0	NA	NA	0	0.25	3513	0.25	3513
SHIP	4070	49.76	16.27	23294	0.49	4070	4020	1.16	4020	NA	NA	0	5.45	0.94	4055	0.08	4056	NA	NA
SHIP-TREND	986	50.13	13.69	5067	0.44	986	986	0.91	986	NA	NA	0	5.19	0.57	985	0.02	985	NA	NA
TPT	5085	57.51	6.77	7882	1.00	5085	0	NA	0	NA	NA	0	NA	NA	0	NA	NA	NA	NA
UCP	3179	63.27	10.01	4892	0.72	3179	217	1.14	217	7.26	3.09	175	7.20	1.43	153	NA	NA	NA	NA
UDACS	790	62.46	13.73	790	0.61	790	759	0.93	759	NA	NA	0	7.92	1.66	778	0.75	790	NA	NA
UHP	1949	40.26	13.47	1949	0.47	1949	710	0.98	710	5.10	0.86	1151	NA	NA	NA	0.00	1949	0.03	1949
UKB	111966	56.91	7.93	111966	0.47	111966	NA	NA	NA	NA	NA	NA	NA	NA	NA	0.05	111966	NA	NA
UKHLS	9531	52.87	16.81	9531	0.44	9531	0	NA	0	NA	NA	0	5.64	0.74	8490	NA	NA	NA	NA
WGHS	23294	54.70	7.12	23294	0.00	23294	23186	0.88	23186	NA	NA	0	5.08	0.59	23138	NA	NA	0.08	22718
WHII	5067	44.38	5.94	5067	0.74	5067	2150	1.01	2150	5.22	0.59	0	5.30	0.62	0	0.11	5067	0.04	4484
Women's Health Initiative (WHI)	7882	67.98	6.58	7882	0.00	7882	4686	0.93	4686	NA	NA	4570	NA	NA	5019	NA	NA	NA	NA
WOSCOPS	4892	55.12	5.52	4892	1.00	4892	4892	0.45	4892	4.77	0.68	4892	NA	NA	0	0.01	4892	NA	NA

Appendix Table 4. Non-additivitiy

	rs11206510	rs11591147	rs2479409	rs11583680
p-values for non-additivity of the association with LDL-C	0.016	0.204	0.044	0.655
p-values for non-additivity of the association with Fasting Glucose	0.791	0.817	0.744	0.269
p-values for non-additivity of the association with Non-Fasting Glucose	0.632	0.898	0.918	0.241
p-values for non-additivity of the association with HbA1c	0.168	0.974	0.306	0.689
p-values for non-additivity of the association with Body weight	0.506	0.644	0.048	0.978
p-values for non-additivity of the association with BMI	0.424	0.808	0.019	0.486
p-values for non-additivity of the association with Waist to hip ratio	0.981	0.678	0.255	0.961
p-values for non-additivity of the association with Fasting insulin	0.413	0.054	0.415	0.150
p-values for non-additivity of the association with Non-Fasting insulin	0.332	0.703	0.799	0.110
p-values for non-additivity of the association with HOMA-IR	0.977	0.078	0.347	0.425
p-values for non-additivity of the association with HOMA-B	0.982	0.531	0.628	0.260

Footnote: P values lower than 0.05 are highlighted red
